# Read-Across of Biotransformation Potential between Activated Sludge and the Terrestrial Environment: Toward Making It Practical and Plausible

**DOI:** 10.1021/acs.est.4c09306

**Published:** 2025-01-14

**Authors:** Claudia Coll, Claudio Screpanti, Jasmin Hafner, Kunyang Zhang, Kathrin Fenner

**Affiliations:** 1 Eawag, Swiss Federal Institute of Aquatic Science and Technology, Dübendorf 8600, Switzerland; 2 Soil Health Research Center, Biology Research, Syngenta Crop Protection AG, Schaffhauserstrasse 101, Stein CH-4332, Switzerland; 3 Department of Chemistry, University of Zürich, Zürich 8057, Switzerland

**Keywords:** Biotransformation, read-across, activated sludge, OECD 307, OECD 308

## Abstract

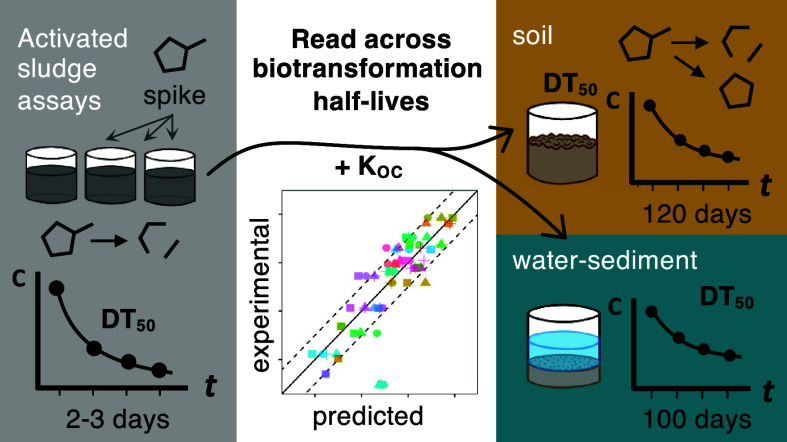

Recent emphasis on the development of safe-and-sustainable-by-design chemicals highlights the need for methods facilitating the early assessment of persistence. Activated sludge experiments have been proposed as a time- and resource-efficient way to predict half-lives in simulation studies. Here, this persistence “read-across” approach was developed to be more broadly and robustly applicable. We evaluated 21 previously used reference plant protection products (PPPs) for their broader applicability in calibrating regression and classification models for predicting half-lives in soil (DT50_OECD307_) and water-sediment systems (DT50_OECD308_) based on their half-life in sludge and the organic carbon–water partition coefficient *K*_OC_ as predictors. The calibrated regression models showed satisfactory predictions of DT50_OECD307_ for another 22 test PPPs. Performance was less satisfying for the prediction of DT50_OECD308_ for 46 active pharmaceutical ingredients (APIs), suggesting a need for expanding the set of calibration substances and more experimental *K*_OC_ values. The classification models mostly correctly classified persistent and non-persistent test compounds for both PPPs and APIs, which is relevant for early-stage screening of persistence. Transformation products of the reference compounds in activated sludge samples were consistent with the reported degradation pathways in soil, particularly with respect to major aerobic, enzyme-catalyzed transformation reactions. Overall, “reading across” biotransformation in environmental compartments such as soils or sediments from experiments with activated sludge outperformed three widely used *in silico* approaches for estimating half-lives and hence has immediate potential to support early assessment of biodegradability when aiming to develop chemicals that are safe and sustainable by design.

## Abstract 2

Efficient assessment of environmental persistence for the design of safe and sustainable chemicals may be achieved by read-across of biotransformation half-lives from activated sludge to soil and water-sediment systems.

## 1 Introduction

The EU commission and chemical industry are currently leading efforts to develop products that are “safe and sustainable by design” (SSbD). Designing chemicals for degradability is an important principle of green chemistry,^1^ as will persistence likely become a key criterion in the initial hazard assessment step of the tiered procedure suggested by the European Commission’s assessment framework for “safe and sustainable by design” chemicals and materials.^2^ Accordingly, there is a need to identify chemicals that could be potentially persistent in the environment in early phases of product development, and to further develop the chemicals that are unlikely to exceed persistence criteria according to current regulatory frameworks.^2−4^

Standard tests to assess ready biodegradability of chemicals exist (OECD 301 series), but the results cannot be directly extrapolated to environmental half-lives because they give only categorical yes/no answers and use unrealistically high concentrations of chemicals. Standard regulatory simulation studies, which aim to represent more environmentally relevant conditions (i.e., OECD 307, OECD 308, and OECD 309), are not adequate for early-stage testing because they are cost- and time-intensive and hence not high-throughput.^5^ Further compartment-specific and enhanced screening tests recently proposed seem to suffer from similar limitations and have been tested for very few chemicals only.^6,7^ More high-throughput, e.g., well plate-based, approaches have been tested with aquatic microbial communities and activated sludge inocula. These approaches, however, seem to have limited applicability because the tested compounds had to be restricted to phenol structures to achieve a colorimetric read-out and are focused on ready biodegradability outcomes.^8,9^

Previous research has shown that half-lives derived from mixture experiments with activated sludge can be used to predict primary biotransformation half-lives of plant protection products (PPPs) in soil as determined according to OECD 307 guidelines.^10^ It has therefore been suggested that such experiments could be a useful medium-throughput approach to predict persistence in soil because they can be used with mixtures of chemicals and provide biotransformation read-outs within a relatively short duration of 48 h thanks to increased bioavailability of the test substances and/or higher biomass relative to simulation study set-ups. Yet, practical and theoretical questions on the applicability of this so-called “read-across approach” remain open. First, the approach has only been tested for 52 commercially available PPPs, and, hence, its applicability to a wider range of chemicals and chemicals with different exposure histories in activated sludge remains unclear (question (i)). For instance, is the approach applicable to pharmaceuticals too, particularly if emerging processes such as adaptation to high concentrations in wastewater treatment plants (WWTP) might affect the fate of these compounds?^11^ Can biotransformation of speciating compounds and hence pH-dependent bioavailability be sufficiently captured? Second, the underlying rationale for read-across between different microbial communities to potentially work is not fully understood, and it therefore remains unknown if we can expect similar functions from microbial communities that widely differ in terms of taxonomic composition (question (ii)). Third, since these experiments rely on freshly sampled sludge, methods are required to obtain consistent results from experiments run at different seasons or with activated sludge obtained from different sources (question (iii)).

The aim of this paper is to address these open questions by testing and validating the practical application of the read-across approach for a wider set of compounds and including water-sediment total system half-lives as prediction endpoints. Similar to the method proposed in,^12^ a number of reference compounds were selected from the previously tested PPPs to calibrate the read-across approach, assuming that the relative persistence of chemicals is robust against the latent variability in biotransformation outcomes. We performed four biotransformation experiments between 2018 and 2021, including one with a different WWTP inoculum, to assess repeatability of the approach across seasons and to obtain a first proof-of-principle regarding transferability across WWTPs (question (iii)). We also broadened the scope of tested chemicals to include novel as well as ubiquitous active pharmaceutical ingredients (APIs), widened the experimental conditions to include a lower sludge pH level, and extended the models to predict DT50s from OECD 308 (question (i)). Finally, we investigated commonalities in biotransformation functions by comparing transformation products (TPs) in soil and activated sludge for the reference compounds (question (ii)).

## 2 Materials and Methods

### 2.1 Compound Selection

We used 21 reference compounds to calibrate the read-across models and for a detailed investigation of the transformation potential between activated sludge and soil microbiomes. With one exception (i.e., bromoxynil), these were chosen to be PPPs that were also included in our previous study.^10^ Other 26 PPPs and 46 APIs with high structural diversity were chosen as test compounds, i.e., for evaluation of the performance of the read-across models (Supporting Information (SI), section S1).

Several factors were considered to select the reference and test compounds used to build and evaluate the read-across models. Both reference and test compounds had to have at least one available regulatory DT50 value obtained from OECD 307 and/or OECD 308 guideline studies. In the case of OECD 308, this had to be a whole system DT50 value. Based on the experimental organic carbon–water partition coefficient (K_OC_) in soil obtained from an OECD 106 test, very hydrophobic compounds (i.e., log K_OC-OECD106_ > 5) and compounds with masses larger than 1000 Da were excluded because of their limited bioavailability. Experimental K_OC_ values (K_OC-OECD106_) were considered as the gold standard for considering differences in bioavailability between systems. However, additional experimental and *in silico* estimated partition coefficients were considered as alternatives: K_OC_ predictions^13^ provided by OPERA^14^ version 2.7, Chemaxon predictions for log P (Chemaxon, https://chemaxon.com/), and experimental log P values obtained from OECD 107 (SI Table S1).

### 2.2 Sludge Biotransformation Experiments and Analysis

In addition to the experiment in 2018 (2018-summer) reported in the previous publication, four more biotransformation experiments were conducted between 2020 and 2021 (2020-fall, 2021-winter, 2021-summer, 2021-fall). The experiments in 2020–2021 were performed by taking approximately 3 L of activated sludge from the nitrification reactor of the local wastewater treatment plant (WWTP) Neugut (Dübendorf, Switzerland). In the case of the experiment in 2021-summer, activated sludge was also collected from the Eawag pilot plant in the Eawag experimental hall (SI section S2.2). The activated sludge was used to prepare four different types of bottle incubations: 1) biotransformation incubations (BT) as main treatments to track biotransformation of chemicals, from which DegT50_sludge_ were derived, 2) abiotic controls (AB) to assess abiotic dissipation of chemicals in activated sludge supernatant, 3) sorption controls (SC) to assess sorption to sludge, and 4) unspiked controls (UC) to identify background levels. Each BT, AB, and SC bottle was spiked with a mixture of reference and test compounds at concentrations of 5–8 μg/L (SI Table S2). Controls were only used for qualitatively discerning dissipation processes other than biotransformation and to explain non-detects in treatments with high biomass.

Duplicate BT bottles were incubated with 50 mL of activated sludge at two sludge biomass levels and at two pH levels to better capture differences in degradation related to the extent of speciation of ionizable compounds and pH effects on microbial communities. High (∼7.5) and low (∼6) pH levels were created by gentle bubbling of air or a mix of air and CO_2_ gas (in a 50:1 L/min ratio), respectively, through all 10 incubation bottles of each pH setup. The two biomass levels according to total suspended solids (TSS) were high (HB, TSS of 5–13 g/L) and dilute (DB, TSS of 0.5–0.7 g/L). Hence, for the majority of experiments, the BT treatment consisted of eight incubations, eight concentration–time series, and eight DegT50_sludge_ values derived for each compound per experiment (for full details of the experimental design, see SI Section S2).

After 1 h of acclimation and pH stabilization, the reference and/or test mixes were spiked at starting concentrations of 5–8 μg/L of each reference or test substance (SI Table S2). Triplicate time point zero 1.5 mL samples were taken immediately after spiking, and subsequent samples were taken after 2, 4, 7, 15, 24, 30, and 48 h in the BT experiments. In summer- and fall-2021 experiments, additional samples were taken at 54 and 72 h but not further considered to obtain a homogeneous data set. Fewer time point samples were taken for controls (AB, SC, and UC; see SI Table S2). Internal standard mixes containing isotopically labeled versions of commercial chemicals were spiked to each sample to aid with the semi-quantification of reference and test compounds (SI Table S5).

### 2.3 Analysis by LC-HRMS, Semi-quantification, and DegT50_sludge_ Calculation

Samples from the biotransformation experiments were measured using reversed-phase liquid chromatography (LC) tandem high-resolution mass spectrometry (HRMS, Q-exactivePlus, Thermo Fisher Scientific). The method was adopted from Fenner et al.^10^ except that MS2 spectra were acquired with the top 5 data-dependent mode, using an inclusion list with masses of parent compounds and previously reported soil transformation products for the reference PPPs (extracted from enviPath Eawag-Soil data package^15^). The full details of the method can be found in SI Table S3. Concentrations of the parent compounds were semi-quantified using TraceFinder 4.1 EFS (Thermo Fisher Scientific).

The concentration–time series (SI Section S4) were log-transformed and linearly fitted assuming first-order kinetics using a least-squares regression standard approach (*lm* function in R software). The dissipation kinetics in BT were fit regardless of sorption behavior or abiotic dissipation (SI section S5.1). The kinetic constant *k* was determined as the slope of the regression. For each fit, a two-tailed *t* test was conducted to determine if *k* was significantly different from zero. DegT50_sludge_ values were only calculated from significant *k* values, using the formula DegT50_sludge_*=* ln(2)/k. This resulted in a maximum of eight DegT50_sludge_ values per compound and experiment.

To properly account for those cases where DegT50_sludge_ values could not be quantified, but the time series indicated very fast dissipation, very slow dissipation, or even increasing concentrations, we defined upper and lower reporting limits for the half-lives that could be determined in our biodegradation experiments. In those cases, the DegT50_sludge_ half-lives were then reported as censored values (SI Section S5.3), i.e., as below 1.2 h (lower reporting limit for left-censoring) or as above 315 or 473 h, depending on the duration of the experiment (upper reporting limit for right-censoring).

Mean sludge log DegT50_sludge_ were obtained for each reference and test compound using Bayesian inference as in.^16^ Bayesian inference was applied to use all available DegT50_sludge_ information, including censored values. The prior distribution of the mean was designed to cover the range of possible half-life values between the upper (473 h, or 1.3 log(days)) and lower (1.2 h, −1.3 log(days)) reporting limits. We therefore chose a normal distribution with a mean DegT50_sludge_ of 0 log(days) (1 day) representing the center of the half-life range and a standard deviation of 1.5 log(days). The standard deviation of the observed DegT50_sludge_ was assumed to be lognormally distributed, with a mean of 0.2 log(days), a standard deviation of 0.2 log(days), and a minimal value of 0.1 log(days), which reflects our assumption that the more homogeneous experimental conditions in sludge reactors as compared to OECD 307 studies used in^16^ lead to a smaller experimental variability. Given the observed data, we then updated our belief using Bayes’ theorem, which establishes that the posterior probability of the parameter given the data is proportional to the product of the likelihood of the data given the parameter and the prior probability of the parameter. The posterior distribution was once inferred for each compound and each experiment. Each posterior distribution was sampled to obtain the mean log DegT50_sludge_, as well as the 50th percentile interval (i.e., the 0.25 and 0.75 quantiles) to characterize its estimated experimental variability. The mean log DegT50_sludge_ for each experiment and compound were then used to build and test regression and classification models.

### 2.4 TP Screening Reference Substances

Samples of experiments from 2020 to 2021 were processed in *Compound Discoverer* v3.2 (ThermoFisher Scientific) according to the workflow detailed in ref. (17). The list of known soil TPs of reference compounds as extracted from Eawag-Soil^15^ was used as a suspect mass list (instead of enviPath predictions). Based on the MS1 and MS2 spectra, individual mass spectrometric features found in the sludge samples were assigned to structures of suspect TPs and assigned confidence levels^18^ by assessing the molecular formula, comparing the fragmentation patterns to the *mzCloud* reference database, and, in some cases, by comparison to the fragmentation of the parent reference compound.

Generalized reaction types (e.g., oxidations, hydrolyses) and transformed functional groups (e.g., esters, amines, etc.) associated with each TP were compiled to identify TPs and transformation reactions that are observed in both soil and the activated sludge experiments.

### 2.5 Regression Models

Read-across linear regression models were developed using half-life data for the reference chemicals and the data from activated sludge experiments from 2018-summer, 2020-fall, 2021-winter, and 2021-fall. The data of experiment 2021-summer was not used because it had a different experimental design targeted toward comparing two different activated sludges, i.e., only high biomass levels. For the linear regression models, the log-transformed geometric means of DT50_OECD307_‘s and DT50_OECD308_‘s were used as response variables, and the mean log DegT50_sludge_ from the activated sludge experiments as well as the log-transformed partition coefficients, determined either experimentally (log K_OC-OECD106_, log P_OECD107_) or predicted (log K_OC-OPERA_, log P_Chemaxon_), as predictor variables. Once outliers (residuals >2*RMSE) were removed, a final version of the reference model was fitted. The coefficient of determination (R^2^), the root mean squared error (RMSE), and the same quality metrics for a leave-one-out-cross validation (LOO–CV) were calculated to assess the performance of the models (the *caret* package functions in R software were used for calculations). To assess the predictive power of these reference models, we then applied them to predict soil and sediment half-lives for the PPP and API test sets (from experiments 2018-fall and 2021-fall, respectively) and assessed the accuracy of predictions by the squared Pearson correlation coefficient and RMSE of the predictions.

### 2.6 Classification Models

Classification models were built using a distance-weighted K-nearest neighbor algorithm based on Euclidean distance (*caret* package functions in R software) to classify compounds’ DegT50_sludge_’s into “persistent” or “non-persistent” according to a threshold. The thresholds chosen were 70 days (based on the median of the DT50_OECD307_ and DT50_OECD308_ of the reference group) and 100 days (threshold closer to persistence criteria for OECD 307 or 308, respectively). Three parameters of the reference compounds were considered in training the classification models: the mean and standard deviation of the posterior log DegT50_sludge_ sludge distribution and one partition coefficient (log K_OC-OECD 106_, log P_OECD 107_, logK_OC-OPERA_ or log P_Chemaxon_). The models trained on the reference compounds were then used to predict the DT50 classification of the PPP and API test sets from experiments 2018-fall and 2021-fall, respectively. A range of K-neighbors (K between 1 to 3) and K_OC_-specific coefficients to adjust the importance of the differences in log K_OC_ (0.1, 1, and 10) were used for prediction optimization. The accuracy, sensitivity, specificity, and precision of the predictions were calculated to assess model performance, defining “non-persistent” as the positive outcome. More details and equations can be found in SI section S7.1.

## 3 Results and Discussion

### 3.1 Relative Biotransformation of Reference Compounds Conserved across Experiments

Concentration–time series were obtained for the 21 reference compounds, but only 17 of them have DegT50_sludge_ in all experiments. Due to nondetection, DegT50_sludge_ of bromoxynil, dicamba, kresoxim-methyl, and topramezone could not be obtained in some experiments (for full information on time series, see Figures S4–S69 in Supporting Information). From each available concentration–time series in BT reactors, one DegT50_sludge_ value was calculated for reference compounds in each experiment (see SI Table S6 for individual DegT50_sludge_ for each experiment and incubation). Censored DegT50_sludge_ values were observed for all compounds in at least one BT reactor of each experiment, except for fenhexamide. Mean log DegT50_sludge_ derived from the individual DegT50_sludge_ values are reported in Table 1.

**Table 1:** Mean log DegT50_sludge_ (d) of Reference Compounds Obtained with Bayesian Inference for Each of the Experiments^a^

**reference compound**	**2018-summer**	**2020-fall**	**2021-winter**	**2021-summer-HB**	**2021-summer-HB-VH**	**2021-fall**
Azoxystrobin	0.23	0.64	0.98	0.38	0.17	0.84
Benzovindiflupyr	1.47	0.55	1.52	1.19	2.22	1.21
Bromoxynil	*na*	*na*	*na*	–0.92	–0.36	–0.43
Cyantraniliprole	0.75	2.18	1.00	2.12	1.31	1.55
Cyclaniliprole	1.14	2.11	2.11	2.16	2.16	2.23
Dicamba	0.78	*nd*	*nd*	0.77	2.17	1.13
Diuron	0.81	1.38	1.24	0.68	0.73	1.26
Fenhexamid	0.22	0.21	0.38	–0.17	0.03	0.52
Fenoxycarb	–1.99	–1.98	–2.02	–1.75	–1.86	–1.96
Fipronil	0.95	2.15	2.17	1.17	1.16	1.10
Florasulam	1.44	1.19	1.04	0.89	2.19	1.22
Fluopyram	1.22	2.15	1.49	1.26	2.15	1.45
Flupyradifurone	1.22	1.41	1.25	1.22	1.28	1.48
Imidacloprid	1.22	1.19	1.23	1.26	1.17	2.22
Isoproturon	0.71	0.74	0.93	0.17	0.34	1.08
Kresoxim-methyl	–1.10	*nd*	*nd*	*nd*	*nd*	–0.78
Mandipropamid	–0.51	–0.26	–0.11	–0.76	–0.49	–0.36
Mesotrione	1.22	0.90	0.85	0.23	0.82	0.74
Oxathiapiprolin	–0.37	–0.06	–0.14	–0.53	–0.36	–0.09
Terbuthylazine	1.23	1.31	2.10	1.32	1.21	1.46
Topramezone	0.81	1.49	2.17	1.03	2.14	*nd*

a HB- is indicated for experiment 2021-summer because it was performed with activated sludge at a high biomass level only. VH- indicates that the experiment was performed with activated sludge from the pilot WWTP in the Eawag experimental hall. *na*- the compound was not added. *nd*- the compound was not detected.

There is variation in the mean log DegT50_sludge_ of the reference compounds between experiments performed at different times, yet there is consistency in relative biodegradation behaviors. Five compounds (bromoxynil, fenoxycarb, kresoxim-methyl, mandipropamid, and oxathiapiprolin) have short mean log DegT50_sludge_ of below 1 day in all experiments and hence are consistently degraded fast. Benzovindiflupyr, cyclaniliprole, fluopyram, and terbuthylazine had the longest mean log DegT50_sludge_ (>13 days, with the exception of benzovindiflupyr in 2020-fall). Across all compounds, we found that despite the inter-experiment variation in degradation kinetics, there is an overall correlation between the mean log DegT50_sludge_ values of the reference compounds between experiments. Pairwise correlations between experiments are above 0.8 using Pearson (r) and above 0.7 using Spearman (ρ) rank correlation coefficients, except for the Spearman rank correlation between experiments 2018-summer/2020-fall and 2018-summer/2021-winter, which drops to 0.45 and 0.64, respectively (SI Figure S70).

The mean log DegT50_sludge_ values were generally well correlated (SI Figure S70,*r* = 0.86, ρ = 0.77) between the incubations using activated sludge from the two WWTPs (Neugut and Eawag experimental hall) in experiment 2021-summer. These data provide some first proof-of-principle that the approach might be robust toward using activated sludge sourced from different WWTPs (Section 2.2).

### 3.2 Linear Regression Models for Reference Compounds

**Figure 1:**
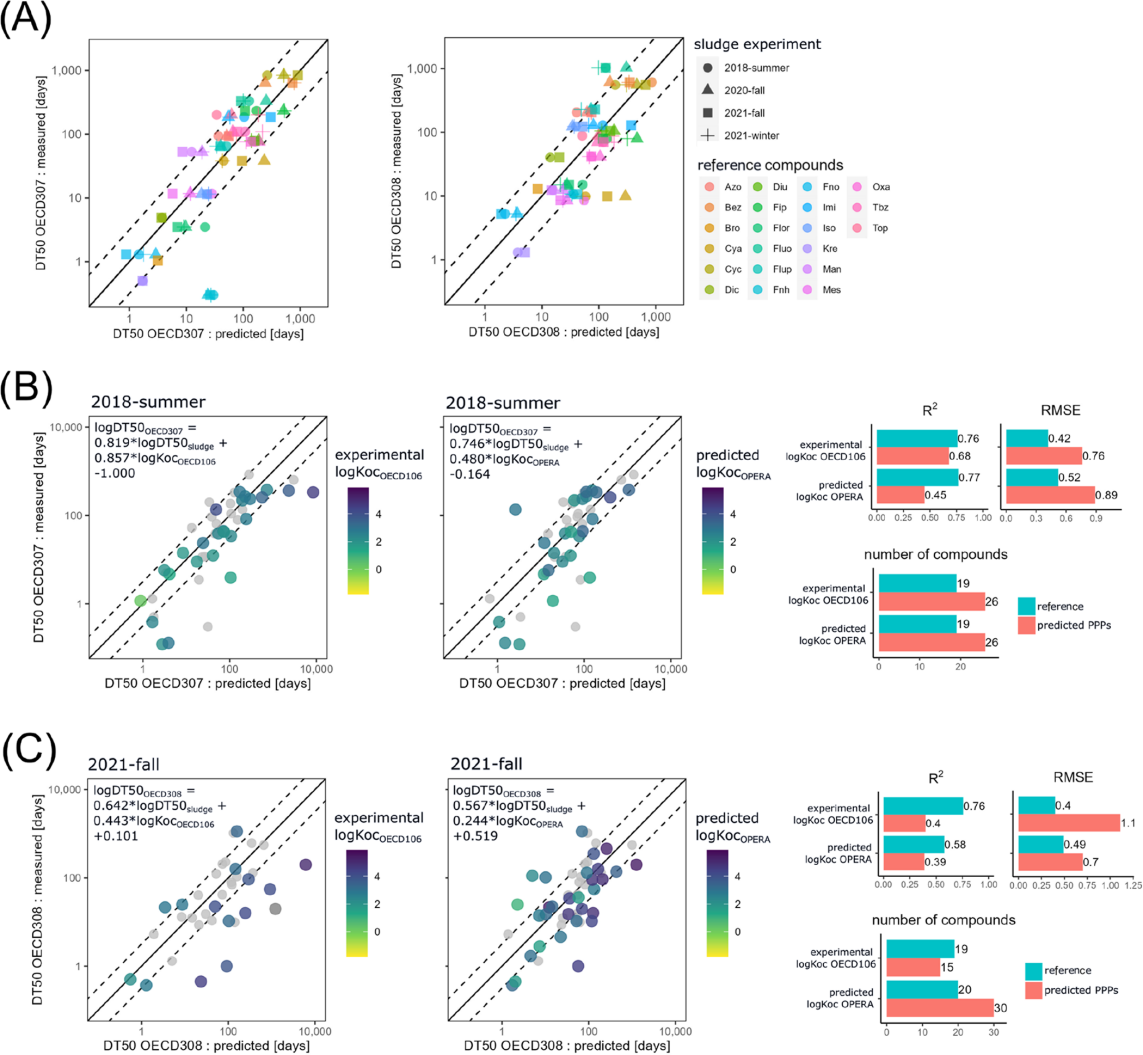
A) Regression models calibrated with reference data of experiment 2018-summer, 2020-fall, 2021-winter and 2021-fall to predict DT50_OECD307_ and DT50_OECD308_. B) Regression models with predicted vs measured DT50_OECD307_ of PPPs and model performance parameters (R^2^, RMSE, and number of compounds) C) Regression models with predicted vs measured D50_OECD308_ of APIs and model performance parameters (R^2^, RMSE and number of compounds). Gray data points show DT50s of calibration compounds.

Regression models for prediction of DT50_OECD307_ values in soil calibrated with the reference compound data show high correlations (R^2^ between 72 and 90%) and low error (RMSE between 0.29 and 0.42) when using the log K_OC-OECD106_ and the mean log DegT50_sludge_ of each experiment as predictors. Model coefficients for the mean log DegT50_sludge_ and for the log K_OC_ coefficient are both significant (Figure 1A). These fit parameters are also in good agreement with the values found in the previous analysis with the 2018-summer data set.^10^

Regression models for prediction of DT50_OECD308_ values in water-sediment systems yielded lower correlations (R^2^ between 52 and 76%) and slightly higher errors (RMSE between 0.39 and 0.48) than models for soil half-lives (SI Tables S9 and S10). Still, the coefficients for the log DegT50_sludge_ and log K_OC_ are both significant. The water-sediment systems are static and have layered aerobic/anaerobic conditions. In those systems, diffusion plays an important role in determining the DT50_OECD308_ of a test compound^19^ as might anaerobic transformation do for some compounds. In contrast, the activated sludge system is mostly aerobic and well-mixed.^19^ Therefore, the decrease of model performance indicators is likely related to the fact that activated sludge systems are not capturing diffusion and anaerobic degradation in water-sediment systems.

The use of the predicted log K_OC-OPERA_ instead of the experimental log K_OC-OECD106_ values yielded models with remarkably similar performance for OECD 307 and slightly lower performance for OECD 308 fits (SI Tables S9 and S10). However, using other partition coefficients as predictors decreased model quality in all cases. This is most likely due to the fact that *K*_OC-OPERA_ is trained on experimentally observed K_OC_ values and hence *de facto* considers speciation, while this is not the case with the log P values used alternatively.

### 3.3 Predictive Power of Regression Models for PPPs and APIs

**Figure 2:**
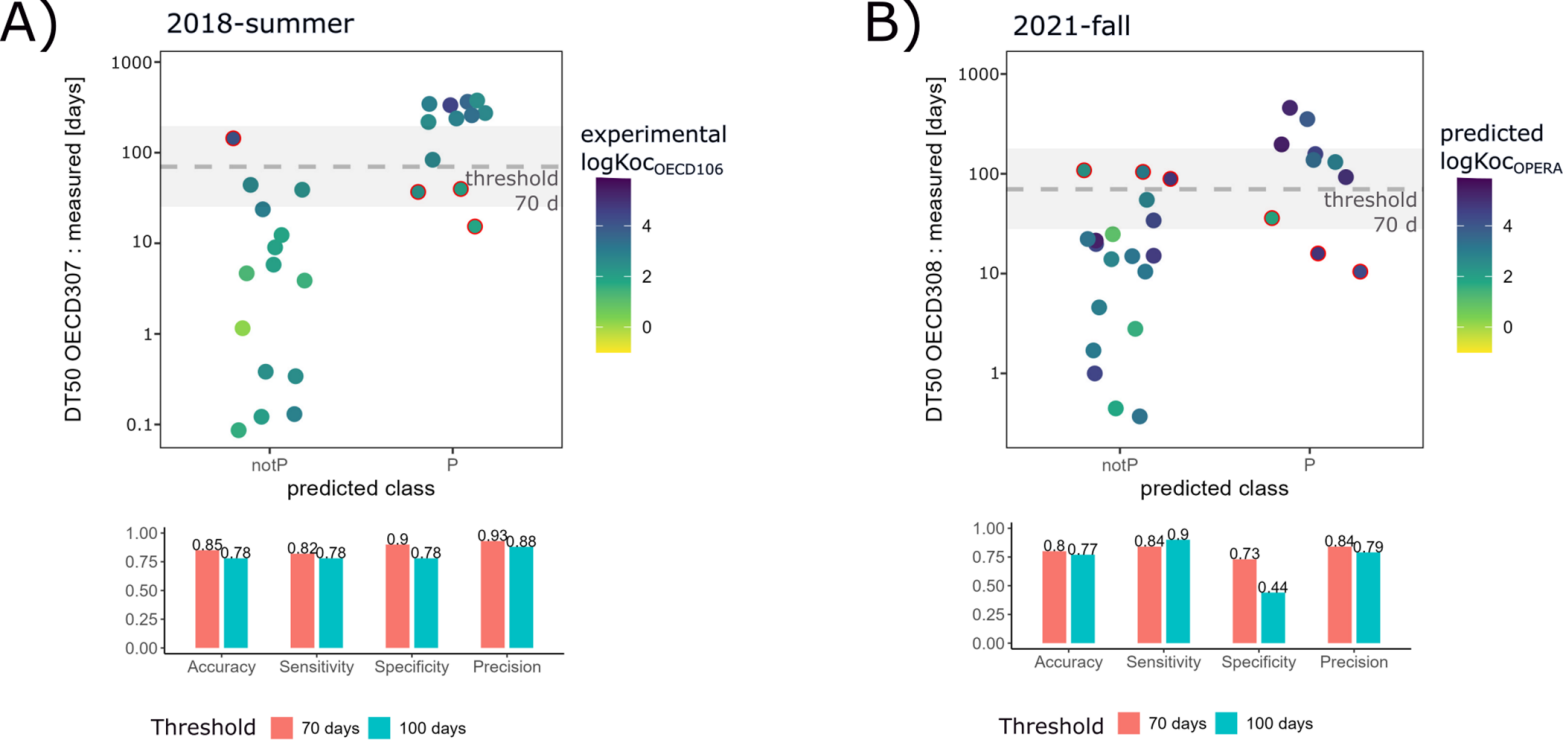
Results of classification models using 2-neighbors to classify A) test PPPs into persistent/non-persistent (P/notP) classes based on the DT50_OECD307_ (adjustment coefficient of 1 for log K_OC_), and B) APIs into persistent/non-persistent classes based on the DT50_OECD308_ (adjustment coefficient of 0.1 for log K_OC_). DT50 plots illustrate classification with threshold of 70 days. The misclassifications are marked with red lines in the plot. Model quality parameters (precision, specificity, sensitivity, and accuracy) are shown in the adjacent bar plots for thresholds of 70 days and 100 days. The gray area indicates ± one standard deviation around the threshold. The average standard deviation of DT50_OECD307_ has been reported to be 0.42 log(days),^16^ and was assumed to be the same for DT50_OECD308_.

The regression models calibrated on reference compound data from experiment 2018-summer were used to predict the soil DT50_OECD307_ of 26 PPPs (Figure 1B), and from experiment 2021-fall to predict the water-sediment DT50_OECD308_ of 46 APIs (Figure 1C). The model using the experimental K_OC-OECD106_ shows the highest correlation between predicted and measured soil DT50_OECD307_ for the 26 test PPPs (R^2^ of 68%). While this is only moderately lower than the correlation found for the reference compounds (76%), the RMSE of the predictions increases considerably (from 0.42 for the reference compounds to 0.76 for the test PPPs). As can be seen from Figure 2A, the model is mostly able to predict soil DT50_OECD307_ between 1 and 200 days but seems to overestimate the DT50s outside of this range (i.e., shorter than 1 day or longer than 200 days), which is expected. Most of the outliers of the different models built with the various partition coefficients (residuals >2*RMSE) have indeed DT50 below 1 day, including fenhexamid, sulfoxaflor, trinexapac-ethyl, and valifenalate (SI Table S9).

The prediction of DT50_OECD308_ for water-sediment systems was faced with the additional challenge of the general scarcity of experimentally determined K_OC-OECD106_’s for APIs (SI Table S1). The read-across model fitted with the K_OC-OECD106_ could only be applied to predict the DT50_OECD308_ of 15 APIs. The resulting predictions generally overestimate the DT50 and have only a weak correlation with the measured values (R^2^ = 40%, RMSE = 1.1, Figure 1C). The lower predictive power of this model could be due to the complexity in fate and transport processes of compounds in water-sediment systems as discussed in the previous section. An additional issue is that all seven API outliers in this model (i.e., atazanavir, atovaquone, ceritinib, clopidogrel, ezetimbe, mirtazepine, and panabinostat) have high log K_OC_ values between 3.2 and 6.1, a range which usually leads to lower bioavailability and hence often slower biotransformation of such chemicals (SI Table S1, Figure S73). Yet, five of these outliers (i.e atovaquone, clopidogrel, ezetimbe, mistazepine, and panabinostat) show rather fast degradation in water-sediment systems (DT50*s* < 60 days; SI Table S1), suggesting that the bioavailable fraction is biotransformed very quickly. Although compounds with similar combinations of properties, i.e., high log K_OC_ and low DT50_OECD308_, were included in the reference set (i.e., fenoxycarb and oxathiapiprolin; SI Table S1), the read-across model seemed to not be able to properly translate the behavior of these types of compounds from activated sludge to water-sediment systems (SI Figure S73).

Even though the regression models using the *K*_OC-OPERA_ had satisfactory performance for the prediction of soil and water-sediment DT50 of the reference compounds (SI Table S9 and Table S10), when applied to the test PPPs and APIs, the agreement between predicted and measured DT50s was clearly worse, i.e., R^2^ = 45%, RMSE = 0.89 for PPPs for OECD 307 (Figure 1B) and R^2^ = 39%, RMSE = 0.70 for APIs for OECD 308 (Figure 1C). It is worth noting, however, that the RMSE for DT50_OECD308_ of the test APIs was clearly better for the model trained with K_OC-OPERA_ (RMSE = 0.70) than for the one trained with K_OC-OECD106_ (RMSE = 1.1). This is only partly due to the larger number of test compounds (n= 30 with K_OC-OPERA_ versus n= 15 with K_OC-OECD106_), giving less weight to outliers. When only considering compounds that have both K_OC-OPERA_ and K_OC-OECD106_ (n= 14), the performance is still higher with *K*_OC-OPERA_ (RMSE = 0.80, R^2^=43%), the reason for this being unclear.

### 3.4 Identification of Non-persistent Compounds in Soil Using Classification Models

Quantitative prediction of half-lives is useful for exposure modeling purposes but in certain contexts, e.g., persistence assessment or alternatives assessment, a qualitative prediction might be sufficient.^20^ Therefore, and due to the partially low predictive power of the regression models, we also trained and explored classification models with our data. Classification models trained with the reference compound data of experiment 2018-summer were generally able to correctly classify the PPP test set into persistent and non-persistent. A threshold of 70 days for soil DT50_OECD307_ was found to be optimal across models. With this threshold, the best performance (accuracy, sensitivity, specificity, and precision) was achieved using the log K_OC-OECD106_ with an adjustment coefficient of 1, which gives the same importance to the difference between log K_OC_ values as it gives to the differences between the mean and standard deviation of log DegT50 sludge (performance metrics >0.75, Figure 2A). The sensitivity and the specificity of the models (around 0.82 and 0.9, respectively) points to a consistent classification of non-persistent compounds as well as persistent compounds for a large proportion of the compounds. It is worth noting that although the reference training set for classification is balanced relative to the 70-day threshold (i.e., even amount in persistent/not persistent categories), only 38% of the PPP test set is persistent according to this threshold, which in itself can influence the sensitivity and specificity values for predictions. The performance of models using the log P_OECD107_, log P_Chemaxon_, and the log K_OC-OPERA_ were less satisfactory, with low accuracies and sensitivities (SI Table S11).

The classification of test APIs into persistent/non-persistent with a threshold of 70 days based on DT50_OECD308_ also showed acceptable performance parameters (Figure 2B), comparable to the predictions of the PPP test set for DT50_OECD307_. There was better performance with a 0.1 adjustment coefficient for the log K_OC_, which means that the difference in K_OC_ has less importance than the difference in mean and standard deviation of the log DegT50 sludge for prediction of DT50_OECD308_ in water-sediment systems. Due to this, there is lower variation in the performance of the models for OECD 308 persistence prediction according to the choice of partition coefficient (SI Table S13).

The four performance parameters were highest using one neighbor for classification for prediction of both OECD 307 and OECD 308 DT50s (Tables S11 and S13). Here, we report the results of models using two neighbors because their performance was still adequate and because using one neighbor can lead to overfitting. Using three neighbors weakened the prediction performance and therefore increasing the size of the reference training set would likely be needed to use more neighbors. The models were slightly more robust when using a threshold of 70 days (i.e., the median of the DT50s of the training set) than a threshold of 100 days (Figure 2). Even though it would be desirable to increase the classification threshold to better approximate the regulatory persistence criteria of 120 days for OECD 307 and OECD 308, this causes an imbalance in the reference training set and the test sets (i.e., very few compounds would be classified as persistent) and a decline in the models’ classification power.

### 3.5 Comparable Spectrum of Aerobic Biotransformation Reactions of Reference Compounds between Sludge and Soil

The organisms, genes, and functions driving biotransformation of chemicals are unknown for the majority of PPPs and APIs and are generally difficult to identify. Therefore, it would be difficult to confirm if a specific biotransformation is due to the presence of the same organism/enzyme in sludge and soil. The screening of transformation products through LC-HRMS and grouping into general biotransformation functions might be a way to generate practical knowledge on the functional diversity that would allow for effective read-across.

We grouped common biotransformation reactions of the reference compounds in soil by relevant transformed or produced moieties or functional groups and compared if we observed the same reactions in our activated sludge experiments (Table 2 and SI Table S14). In general, there seems to be consistency between transformation reactions in aerobic soil and sludge for hydrolysis and oxidation of oxygen-containing groups (e.g., alcohols, esters, ethers, and ketones). Hydrolysis and oxidation of nitrogen-containing groups (e.g., nitriles, amines, amides) occur in both aerobic soil and activated sludge but are less readily available in sludge. For example, we found evidence of amide hydrolysis in activated sludge for only half of the reference compounds that show that transformation in soil. This observation might be related to the fact that many amidohydrolases exhibit high substrate specificities.^21−23^ The presence/absence of specific amidohydrolases in different microbial communities therefore can be expected to lead to more observable differences than it is the case for some of the known unspecific monooxygenases likely involved in oxidative processes.

**Table 2:** Comparison between Biotransformation Reactions Reported in Soil (Taken from the Eawag-Soil Package in EnviPath) and Observed in This Study’s Activated Sludge Experiment

		**Observed in**	
**Reaction type**	**Reference compounds**	**Soil**	**Sludge**
**Oxygen-containing groups**			
Hydrolysis of **ester**	Azoxystrobin	√	√
	Kresoxim-methyl	√	√
Oxidative **ether** cleavage/O-dealkylation Ar–O–Al and Al–O–Al	Azoxystrobin	√	√
	Cyantraniliprole	√	
	Dicamba	√	√
	Fenoxycarb	√	
	Florasulam	√	√
	Mandipropamid	√	√
Oxidative **ether** cleavage Ar–O–Ar	Azoxystrobin	√	√
	Fenoxycarb	√	√
Oxidation of **alcohol**	Fenoxycarb (CGA-294848)	√	
	Fluopyram (secondary alcohol)	√	
	Isoproturon (primary alcohol)	√	√
	Kresoxim-methyl (primary alcohol)	√	
	Oxathiapiprolin (primary alcohol)	√	√
Oxidative cleavage in α -position to **ketone**	Mesotrione (triketone)	√	√
	Topramezone	√	√
Cleavage of **C–C bond** in α -position to carboxylic acid	Azoxystrobin (alkene group)	√	
**C-**hydroxylation	Azoxystrobin (not located)	√	
	Benzovindiflupyr (aliphatic ring)	√	
	Dicamba (C-aromatic ring)	√	
	Fenoxycarb (C-aromatic ring)	√	√
	Fluopyram (secondary C)	√	√
	Imidacloprid (aliphatic ring)	√	√
	Isoproturon (primary and secondary C)	√√	√
	Kresoxim-methyl (primary C)	√	
	Oxathiapiprolin (primary C, pyrazole ring, and tertiary C, aliphatic ring)	√√	√
	Terbuthylazine (primary C)	√	√
	Topramezone (primary C, aromatic methyl))	√	
**Reductive C-**dehydroxylation	Bromoxynil (phenyl, anaerobic)	√	
Decarboxylation	Florasulam (triazole ring), topramezone (benzoic acid)	√√	
Dehydration	Imidacloprid (imidazol ring)	√	
**Nitrogen-containing groups**			
Reduction of nitro group	Imidacloprid (to nitroso)	√	√
	Mesotrione (to amine)	√	√
Cleavage of nitro group	Imidacloprid (from nitroguanidine derivative)	√	√
Hydrolysis of **nitrile**	Azoxystrobin	√	
	Bromoxynil	√	√
	Cyantraniliprole	√	
	Fipronil	√	
**Nitrile** reduction	Bromoxynil	√	
Hydrolysis of **amide**	Bromoxynil (primary amide)	√	
	Benzovindiflupyr (secondary amide)	√	
	Cyantraniliprole (primary amide)	√	
	Cyclaniliprole (primary amide)	√	
	Diuron (urea-secondary amide)	√	
	Fenhexamide (secondary amide)		√
	Fipronil (primary amide)	√	
	Mandipropamid (secondary amide)	√	√
	Oxathiapiprolin (tertiary amide)	√	√
Hydrolysis of **imine**	Imidacloprid	√	√
Hydrolysis of **carbamate**	Fenoxycarb	√	
Hydrolysis of **formimidate** derivative	Cyantraniliprole	√	
Hydrolytic cleavage **of C–N bond**	Fenoxycarb (α-position to **carbamate)**	√	
(Hydrolytic) cleavage of **thiazole** ring	Oxathiapiprolin	√	√
Oxidative *N*-dealkylation of **amine**	Benzovindiflupyr (tertiary amine)	√	
	Flupyradifurone (tertiary amine)	√	√
	Oxathiapiprolin (tertiary amine)	√	√
	Terbuthylazine (secondary amine)	√	√
Oxidative *N*-dealkylation of **amide**	Cyantraniliprole (secondary amide)	√	
	Cyclaniliprole (secondary amide)	√	
	Diuron (urea- tertiary and secondary amide)	√√	√
	Fluopyram (secondary amide)	√	√
	Isoproturon (urea- tertiary and secondary amide)	√√	√
Oxidative cleavage of **amide**	Fenoxycarb (α-position to carbamate)	√	
	Mandipropamid (secondary amide)		√
Oxidative cleavage of sulfanilamide moiety	Florasulam (sulfonamide)	√	
Oxidation of heterocyclic C–N rings	Imidacloprid (imidazol ring)	√	√
	Topramezone (pyrazole ring)	√	
	Florasulam (triazolo-pyrimidine moiety)	√	
Aldoxime to nitrile	Topramezone	√	
**S-containing groups**			
Oxidation of **sulfoxide** to sulfone	Fipronil	√	√
Reduction of **sulfoxide** to sulfide	Fipronil	√	
Sulfoxidation of pyridyl ring (anaerobic)	Fluopyram (dechlorination)	√	
**Others**			
Halogenation	Flupyradifurone (chlorination, lactone ring)	√	
Reductive dehalogenation (anaerobic)	Bromoxynil (debromination, phenyl ring)	√	
	Fenhexamid (dechlorination, phenyl ring)	√	
Hydrolytic dechlorination	Terbuthylazine (triazine ring)	√	√
Deasaturation	Cyantraniliprole (pyrazol ring)	√	
	Imidacloprid (imidazol ring)	√	√
Dimerization	Fenhexamid	√	
Cyclization	Mesotrione (only sediment)	√	
Reduction of double or triple bonds	Mandipropamid	√	
Intramolecular nucleophilic aromatic substitution	Cyantraniliprole (dehydrochlorination and dehydration)	√√	

There was clearly least agreement for a set of reactions observed in soil that are more typically observed under anaerobic conditions. For example, the products of reductive debromination of bromoxynil or the fipronil transformation products resulting from the reduction of the sulfoxide group to a thioether were not found in any of our experiments in activated sludge. While there could be anaerobic regions also in activated sludge flocs,^24,25^ they could have been disturbed by the stirring in the sludge reactors or were simply not prevalent enough to lead to observable amounts of the respective transformation products.

While anaerobic transformation reactions are thus most likely a blind spot when using activated sludge to predict biotransformation in soil or water-sediment systems, the overlap of aerobic biotransformation functions between soil and activated sludge is remarkable given the different composition and complexity of the two types of microbial communities.^26,27^ However, research in functional community profiles suggests that metabolic functions can indeed to some extent be decoupled from taxonomic composition.^28^ This is mostly explained by functional redundancy, i.e., the fact that several organisms are able to perform a given function, given the same environmental conditions.^29,30^ The concept of functional redundancy has mainly been described for broad functions (e.g., respiration, biomass production, nitrification), and it is currently not clear to what extent it can be transferred to biotransformation of diverse chemical contaminants. More research is needed to determine which contaminant biotransformation functions are redundant and if they are thermodynamically favorable across different environmental compartments and conditions. In our study, the activated sludge from the selected WWTP seems to be sufficiently functionally diverse to harbor many biotransformation reactions that have also been observed in soil. This seems to be supported by earlier findings asserting that nitrifying activated sludge above a sludge age of 7–10 days reaches saturation of functional diversity in terms of the richness of enzyme classes.^31^

Qualitatively, our findings suggest that the representation of the soil aerobic pathways in activated sludge are sufficient for read-across to provide satisfactory results for most compounds and to also identify key transformation products. However, we cannot exclude that for certain transformation reactions, their relative speed and hence also the relative abundance of the resulting transformation products could be very different in soil and activated sludge. Such seems to be the case for the hydrolysis of the nitrile groups, which takes place relatively readily in soil but not necessarily as fast as other transformation reactions in activated sludge. Soil transformation products indicating nitrile hydrolysis have been reported for azoxystrobin, bromoxynil, cyantraniliprole, and fipronil, but the corresponding product was only observed for bromoxynil in our sludge experiments (Table 2 and SI Table S14). More quantitative evaluations of the abundances of specific reaction-catalyzing enzymes or gene transcripts would be needed to shed more light on these aspects but were beyond the scope of this study.

### 3.6 Environmental Relevance

Assessing the fate of PPPs and APIs in soil and water-sediment systems is an arduous and complex task, but necessary to understand the environmental risk of these chemicals. Through several experiments over a period of three years, we have corroborated the potential of using a short, 2-days activated sludge assay to estimate biotransformation half-lives of PPPs and APIs in OECD 307 and OECD 308 standard tests through read-across.

We could show that the predictive power of the regression models was adequate for PPPs in soil, but less satisfactory for APIs in water-sediment systems. However, it is also important to reflect our findings in the light of existing alternatives for predicting environmental half-lives. We found that current *in silico* tools, i.e., models that predict environmental half-lives directly from molecular structures such as OPERA-BioHL,^14^ VEGA,^32^ and EPI Suite BIOWIN4,^33^ are not suitable to predict DT50s of PPPs and APIs studied here (R^2^ < 10%, Figures S71 and S72). While BIOWIN did not indicate any applicability domain, VEGA tagged the predictions as “low reliability,” and OPERA-BioHL is designed specifically for hydrocarbons and therefore low performance was expected. In this context, it is noteworthy that the Pearson and Spearman correlation coefficients of the comparison between experimental DT50 measured in OECD 307 and OECD 308 studies reach 0.80 and 0.83, respectively (SI Figure S72, n = 48), further supporting the notion that experimental biotransformation half-lives from another environmental compartment give better predictions than computational tools estimating persistence from structure alone. Overall, “reading across” biotransformation in environmental compartments such as soils or sediments from experiments with activated sludge outperformed three widely used *in silico* approaches for estimating half-lives, and hence has immediate potential to support early assessment of biodegradability when aiming to develop chemicals that are safe and sustainable by design.

Using a set of reference compounds to calibrate and train regression or classification models seems to be a key aspect of the robustness of the approach, as it buffers the impact of the inherent inter-experimental variability of DegT50_sludge_. We, however, note that all the activated sludge communities used in this study were obtained from nitrifying activated sludge. We therefore cannot know how the approach would perform with, e.g., C-eliminating activated sludge only. It could be speculated though that due to the lower sludge residence time of such activated sludge systems, and due to their known reduced biodiversity and micropollutant biotransformation capacity,^34−36^ their performance in read-across approaches would be inferior. Further caution must be exercised to apply the read-across model to novel structures that differ substantially from the reference compounds and to quantitatively interpret predicted DT50_OECD307/308_ values that lie outside the approximate range of 1 to 200 days. Additional reference compounds, especially with high log K_OC_ and fast biodegradability, should likely be added to the calibration set to better represent APIs and broaden the applicability domain of the approach.

Finally, classification models with the current setup provide reasonable predictions of biodegradability/non-persistence of PPPs and APIs. Discriminating between persistent and non-persistent compounds might be suitable for situations where fast screening or prioritization are needed. Specifically, early-stage assessment of persistence in research and development of chemicals would require medium/high-throughput methodologies able to provide reliable estimates of biodegradability in a short time that can be aligned to Design-Synthesis-Testing-Analysis (DSTA) cycles.^37^ The current implementation of the read-across approach using activated sludge assays shows promise as a medium-throughput method for early-stage assessment of persistence. However, to maximize its potential, the setup should be optimized and tailored to meet industry’s everyday demand for continuous screening. Also, acceptable levels of uncertainty in classification and regression have to be defined in close collaboration with interested industry partners. With these further optimizations in place, the read-across approach with activated sludge assays could ultimately aid in the development of SSbD chemicals by providing estimates of persistence and mechanistic insights into biotransformation reactions.

## Supporting Information Available

Compound selection, activated sludge experiments, analytical methods for LC-HRMS, time series of tested compounds, DegT50_sludge_ of tested compounds, classification models, TPs (PDF)
