# Supplementary material for: Read-Across of Biotransformation Potential between Activated Sludge and the Terrestrial Environment: Toward Making It Practical and Plausible
Source: Environ Sci Technol. 2025 Jan 14;59(3):1790–800. doi: 10.1021/acs.est.4c09306 (PMC11780744; doi:10.1021/acs.est.4c09306)
Supplement: Supplementary file 1 — es4c09306_si_001.pdf [file es4c09306_si_001.pdf]

Read-across of biotransformation potential between activated sludge and the terrestrial environment: Toward making it practical and plausible

SUPPORTING INFORMATION

Claudia Coll[1,2], Claudio Screpanti[2], Jasmin Hafner[1,3], Kunyang Zhang[1,3], Kathrin Fenner[1,3]

[1] Eawag, Swiss Federal Institute of Aquatic Science and Technology, 8600 Dübendorf, Switzerland
[2] Syngenta Crop Protection AG, Chemical Research, Schaffhauserstrasse 101, CH-4332 Stein, Switzerland
[3] Department of Chemistry, University of Zürich, 8057 Zürich, Switzerland.

KEYWORDS Biotransformation, read-across, activated sludge, OECD 307, OECD 308

Summary: 130 pages, 14 tables, 73 figures.

Table of Contents

S1. Compound selection 8
S2. Activated sludge experiments 12
S2.1 Experimental design 12
S2.2 Comparison of operational parameters of Eawag pilot WWTP plant in the experimental hall and WWTP Neugut 14
Eawag experimental hall (“Versuchshalle”, VH) 14
WWTP Neugut (NE) 14
S2.3 Preparation of sludge biomass solutions, TSS, pH and dissolved oxygen measurements 14
S3. Analytical method for LC-HRMS 16
S4. Time series of tested compounds 22
S4.1 Reference compounds 22
S4.2 Plant protection products (PPPs) 43
S4.3 Active pharmaceutical ingredients (APIs) 44
S5. $DegT50_{sludge}$ of tested compounds 59
S5.1 $DegT50_{sludge}$ calculation 59
S5.2 Quality controls and calculation of $DegT50_{sludge}$ 59
S5.3 All $DegT50_{sludge}$ and kinetic fit parameters 60
S5.4 Bayesian mean of log $DegT50_{sludge}$ of each compound 85
S5.5 Reference $DegT50_{sludge}$ and correlation across experiments 89
Regression models 91
Prediction of OECD 307 91
S5.6 91
S5.7 Prediction of OECD 308 94
S5.8 Comparison to other predictions of DT50s 97
S5.9 DT50 vs logKoc chemical space 99
S6. Classification models 100
S6.1 Equations of the classification models 100
S6.2 Prediction of non-persistence in classification models for OECD 307 DT50s 102
S6.3 Prediction of non-persistence in classification models for OECD 308 DT50s 104
S7. Transformation products 107
S8. References 129

Table of Figures

Figure S1. Total suspended solids- TSS in dry weight of sludge solids per liter of sludge for each experiment 14

Figure S2. pH measurements in sludge experiments 15

Figure S3. Dissolved oxygen- DO [mg/L] measurements in sludge experiments 16

Figure S4. Timeseries of semi-quantified concentrations of azoxystrobin in all 5 experiments .. 23

Figure S5. Timeseries of semi-quantified concentrations of benzovindiflupyr in all 5 experiments 24

Figure S6. Timeseries of semi-quantified concentrations of bromoxynil in the 2 experiments where it was added to spike solution. 25

Figure S7. Timeseries of semi-quantified concentrations of cyantraniliprole in all 5 experiments 26

Figure S8. Timeseries of semi-quantified concentrations of cyclaniliprole in all 5 experiments. 27

Figure S9. Timeseries of semi-quantified concentrations of dicamba in the 3 experiments where measurements were made in ESI negative mode. 28

Figure S10. Timeseries of semi-quantified concentrations of diuron in all 5 experiments 29

Figure S11. Timeseries of semi-quantified concentrations of fenhexamid in all 5 experiments .. 30

Figure S12. Timeseries of semi-quantified concentrations of fenoxycarb in all 5 experiments... 31

Figure S13. Timeseries of semi-quantified concentrations of fipronil in all 5 experiments........ 32

Figure S14. Timeseries of semi-quantified concentrations of florasulam in all 5 experiments ... 33

Figure S15. Timeseries of semi-quantified concentrations of fluopyram in all 5 experiments 34

Figure S16. Timeseries of semi-quantified concentrations of flupyradifurone in all 5 experiments 35

Figure S17. Timeseries of semi-quantified concentrations of imidacloprid in all 5 experiments 36

Figure S18. Timeseries of semi-quantified concentrations of isoproturon in all 5 experiments .. 37

Figure S19. Timeseries of semi-quantified concentrations of kresoxim-methyl in all 5 experiments 38

Figure S20. Timeseries of semi-quantified concentrations of mandipropamid in all 5 experiments 39

Figure S21. Timeseries of semi-quantified concentrations of mesotrione in all 5 experiments ... 40

Figure S22. Timeseries of semi-quantified concentrations of oxathiapiprolin in all 5 experiments 41

Figure S23. Timeseries of semi-quantified concentrations of terbuthylazine in all 5 experiments 42

Figure S24. Timeseries of semi-quantified concentrations of topramezone in all 4 experiments where it was detected 43

Figure S25. Timeseries of semi-quantified concentrations of acalabrutinib in experiment 2021-fall 44

Figure S26. Timeseries of semi-quantified concentrations of aliskiren in experiment 2021-fall. 44

Figure S27. Timeseries of semi-quantified concentrations of amlodipine in experiment 2021-fall 44

Figure S28. Timeseries of semi-quantified concentrations of atomoxetine in experiment 2021-fall 45

Figure S29, Timeseries of semi-quantified concentrations of atovaquone in experiment 2021-fall 45

Figure S30. Timeseries of semi-quantified concentrations of atazanavir in experiment 2021-fall 45

Figure S31. Timeseries of semi-quantified concentrations of budesonide in experiment 2021-fall 46

Figure S32. Timeseries of semi-quantified concentrations of canagliflozin in experiment 2021-fall 46

Figure S33. Timeseries of semi-quantified concentrations of ceritinib in experiment 2021-fall . 46

Figure S34. Timeseries of semi-quantified concentrations of clopidogrel in experiment 2021-fall 47

Figure S35. Timeseries of semi-quantified concentrations of clotrimazol in experiment 2021-fall 47

Figure S36. Timeseries of semi-quantified concentrations of dapagliflozin in experiment 2021-fall 47

Figure S37. Timeseries of semi-quantified concentrations of dasatinib in experiment 2021-fall 48

Figure S38. Timeseries of semi-quantified concentrations of dienogest in experiment 2021-fall 48

Figure S39. Timeseries of semi-quantified concentrations of dolutegravir in experiment 2021-fall 48

Figure S40. Timeseries of semi-quantified concentrations of duloxetine in experiment 2021-fall 49

Figure S41. Timeseries of semi-quantified concentrations of efavirenz in experiment 2021-fall 49

Figure S42. Timeseries of semi-quantified concentrations of ezetimbe in experiment 2021-fall 49

Figure S43. Timeseries of semi-quantified concentrations of fexofenadine in experiment 2021-fall 50

Figure S44. Timeseries of semi-quantified concentrations of fingolimod in experiment 2021-fall 50

Figure S45. Timeseries of semi-quantified concentrations of hydrochlorothiazide in experiment 2021-fall 50

Figure S46. Timeseries of semi-quantified concentrations of irbesartan in experiment 2021-fall51

Figure S47. Timeseries of semi-quantified concentrations of keto-desogestrel in experiment 2021-fall 51

Figure S48. Timeseries of semi-quantified concentrations of lumiracoxib in experiment 2021-fall 51

Figure S49. Timeseries of semi-quantified concentrations of metformin in experiment 2021-fall 52

Figure S50. Timeseries of semi-quantified concentrations of mirtazepine in experiment 2021-fall 52

Figure S51. Timeseries of semi-quantified concentrations of mometasone in experiment 2021-fall 52

Figure S52. Timeseries of semi-quantified concentrations of naloxegol in experiment 2021-fall53

Figure S53. Timeseries of semi-quantified concentrations of nilotinib in experiment 2021-fall. 53

Figure S54 Timeseries of semi-quantified concentrations of olanzepine in experiment 2021-fall 53

Figure S55. Timeseries of semi-quantified concentrations of omeprazole in experiment 2021-fall 54

Figure S56. Timeseries of semi-quantified concentrations of orlistat in experiment 2021-fall ... 54

Figure S57. Timeseries of semi-quantified concentrations of panabinostat in experiment 2021-fall 54

Figure S58. Timeseries of semi-quantified concentrations of pemetrexed in experiment 2021-fall 55

Figure S59. Timeseries of semi-quantified concentrations of pioglitazone in experiment 2021-fall 55

Figure S60. Timeseries of semi-quantified concentrations of quetiapine in experiment 2021-fall 55

Figure S61. Timeseries of semi-quantified concentrations of regorafenib in experiment 2021-fall 56

Figure S62. Timeseries of semi-quantified concentrations of rivastigmine in experiment 2021-fall 56

Figure S63. Timeseries of semi-quantified concentrations of rosuvastatin in experiment 2021-fall 56

Figure S64. Timeseries of semi-quantified concentrations of tadalafil in experiment 2021-fall . 57

Figure S65. Timeseries of semi-quantified concentrations of terbinafine in experiment 2021-fall 57

Figure S66. Timeseries of semi-quantified concentrations of ticagrelor in experiment 2021-fall 57

Figure S67. Timeseries of semi-quantified concentrations of valsartan in experiment 2021-fall 58

Figure S68. Timeseries of semi-quantified concentrations of vildagliptin in experiment 2021-fall 58

Figure S69. Timeseries of semi-quantified concentrations of vorinostat in experiment 2021-fall 58

Figure S70. Correlation matrix (Pearson and Spearman) of the mean $logDegT50_{sludge}$ of reference compounds in different experiments with sludge. 90

Figure S71. Comparison of experimental DT50s from OECD 307 and OECD 308 to the predicted DT50s with OPERA tool and VEGA models for soil and sediment. Compounds for which VEGA provided experimental DT50s are labelled with an “E”, and VEGA predictions with moderate reliability are indicated with a “M”. All other predicted DT50s have a low reliability according to VEGA. 98

Figure S72. A) Pearson and B) Spearman correlations between measured logDT50s from OECD 307 and OECD 308 and the predicted logDT50s from VEGA soil and sediment models and OPERA Biodeg model. 99

Figure S73. Koc vs DT50 of reference, test APIs and test PPPs 100

Table of Tables

Table S1. Compounds tested for the read-across approach, molecular weight (MW), measured or predicted values for Koc or logP and measured / predicted values for degradation half-life (DT50) in the environment. The column labelled "Charge" indicates if the compound is mostly neutral (N), anionic (A) or cationic (C) at the pH levels tested in activated sludge. 8

Table S2. Experimental design and variations on each of the experiments with activated sludge. 13

Table S3. Differences in analytical method used in each experiment. 16

Table S4. Limits of quantification (LOQ) in the LC-HRMS measurements for each compound and experiment in µg/L. na: compound not added, nf: compound not found.............................. 17

Table S5. Internal standards added to each experiment to perform semi-quantification of reference and test compounds. 19

Table S6. $DT50_{sludge}$ [h] of reference, APIs and PPPs tested in all sludge experiments by incubation. Incubations using activated sludge from the WWTP in Eawag-experimental hall are indicated in the biomass column as HB-VH. All other incubations use activated sludge from the WWTP Neugut... 60

Table S7. Log $DegT50_{sludge}$ parameters obtained from Bayesian inference for reference compounds, using kinetic data as available from all experiments. $DegT50_{sludge}$ given in days were used for the calculation of the parameters. 86

Table S8. log $DegT50_{sludge}$ [d] Bayesian posterior parameters of all compounds by experiment. Parameters were estimated separately for the WWTP Neugut (NE) and the Eawag experimental hall (NE) for 2021-summer. 86

Table S9. Fit and performance parameters of regression models to predict soil half-lives (OECD 307) from $DegT50_{sludge}$ data – for PPPs from experiment 2018-summer data, for APIs from experiment 2021-fall data, as well as regression models fitted with data from experiments 2020-summer and 2021-winter for calibration substances only. 91

Table S10. Fit and performance parameters of regression models to predict water-sediment half-lives (OECD 308) from $DegT50_{sludge}$ – for PPPs from experiment 2018-summer data, for APIs from experiment 2021-fall data, as well as regression models fitted with data from experiments 2020-summer and 2021-winter for calibration substances only. 94

Table S11. Results for classification models for the PPP test set with a threshold of 70 d and 100 d for OECD 307. The model uses data from the 2018-summer experiment and either experimental log Koc from OECD 106, predicted log Koc from OPERA, experimental log P from OECD 107 or log P predicted with ChemAxon... 102

Table S12. Results for classification models of the API test set with a threshold of 70 d and 100 d for OECD 308. The model uses data from the 2021-fall experiment and either experimental log Koc from OECD 106, predicted log Koc from OPERA, experimental log P from OECD 107 or log P predicted with ChemAxon.. 104

Table S13. Results for classification models for the PPP test set with a threshold of 70 d and 100 d for OECD 308. The model uses data from the 2018-summer experiment and either exp

experimental log Koc from OECD 106, predicted log Koc from OPERA, experimental log P from OECD 107 or logP predicted with ChemAxon 105

Table S14. Detailed table with transformation products of reference compounds observed in sludge and/or soil 107

S1. Compound selection

The compounds selected are divided in three sets: 21 reference compounds, 46 test APIs and 26 test PPPs.

Table S1. Compounds tested for the read-across approach, molecular weight (MW), measured or predicted values for Koc or logP and measured / predicted values for degradation half-life (DT50) in the environment. The column labelled "Charge" indicates if the compound is mostly neutral (N), anionic (A) or cationic (C) at the pH levels tested in activated sludge.

	Compound name	Short name	MW	Compound set	logKoc OECD 106	logKoc OPERA	logP OECD 107	logP Chem Axon	Charge Chem Axon	DT50 sediment VEGA (days)	DT50 soil VEGA (days)	DT50 OPERA (days)	DT50 OECD 307 (days)	DT50 OECD 308 (days)
1	Azoxystrobin	Azo	403.4	reference	2.77	3.74	2.50	2.65	N	157	34	5.62	93.40	205.23
2	Benzovindiflupyr	Bez	397.1	reference	3.75	3.93	4.30	4.36	N	227	117	5.25	639.20	610.31
3	Bromoxynil	Bro	276.9	reference	2.48	2.67	0.27	3.06	A	49	5	4.90	1.04	13.00
4	Cyantraniliprole	Cya	472.0	reference	2.38	1.64	2.02	1.91	N	72	117	3.39	38.20	9.86
5	Cyclaniliprole	Cyc	598.9	reference	2.90	4.98	2.70	4.32	N	49	117	33.11	840.40	554.40
6	Dicamba	Dic	220.0	reference	1.09	1.50	-1.80	2.40	A	71	23	3.55	4.90	40.42
7	Diuron	Diu	233.1	reference	2.91	2.40	2.87	2.74	N	156	70	4.47	79.20	105.53
8	Fenhexamid	Fnh	301.1	reference	2.68	3.67	3.51	5.86	A	229	40	6.31	0.30	10.74
9	Fenoxycarb	Fno	301.1	reference	3.26	3.00	4.07	4.07	N	157	26	5.13	1.30	5.26
10	Fipronil	Fip	437.1	reference	2.86	2.61	3.75	3.81	N	157	71	3.55	232.80	79.39
11	Florasulam	Flor	359.0	reference	1.34	2.11	-1.22	1.30	A	227	34	4.27	3.50	15.11
12	Fluopyram	Fluo	396.0	reference	2.45	2.58	3.30	3.27	N	227	117	3.55	332.10	1031.83
13	Flupyradifurone	Flup	288.7	reference	1.99	2.51	1.20	0.82	N	71	70	4.90	64.70	228.12
14	Imidacloprid	Imi	255.7	reference	2.35	2.06	0.57	0.60	N	71	71	3.55	185.90	129.00
15	Isoproturon	Iso	206.1	reference	2.09	2.00	2.50	2.40	N	156	20	4.37	11.50	123.25
16	Kresoxim-methyl	Kre	313.1	reference	2.49	3.10	3.40	3.19	N	157	26	4.47	0.50	1.31
17	Mandipropamid	Man	411.9	reference	2.93	4.45	3.20	3.23	N	227	2272	3.39	52.60	12.39
18	Mesotrione	Mes	339.0	reference	1.69	1.76	0.11	1.42	A	23	34	3.55	11.70	8.56
19	Oxathiapiprolin	Oxa	539.1	reference	4.11	5.22	3.66	5.50	N	157	23	3.39	135.80	41.52

20	Terbuthylazine	Tbz	229.1	reference	2.36	2.32	3.40	3.36	N	129	71	4.07	109.60	69.88
21	Topramezone	Top	363.1	reference	2.18	2.25	-1.52	0.31	A	23	34	3.16	201.30	87.96
22	Acalabrutinib	Aca	465.5	test API	6.10	4.40	NA	2.56	N	227	34	11.48	NA	19.80
23	Aliskiren	Ali	551.8	test API	NA	NA	2.70	3.12	C	49	8	NA	NA	89.58
24	Amlodipine	Aml	567.1	test API	NA	1.57	-0.06	1.64	C	227	70	3.39	NA	2.80
25	Atazanavir	Ata	802.9	test API	3.14	4.51	3.30	4.54	N	49	23	4.27	NA	158.15
26	Atomoxetine	Atm	255.4	test API	NA	3.56	NA	3.81	C	232	26	3.31	NA	426.70
27	Atovaquone	Atv	366.8	test API	3.85	4.49	NA	5.00	A	227	2272	72.44	1.00	1.00
28	Budesonide	Bud	430.5	test API	NA	4.73	3.23	2.73	N	13	94	97.72	NA	15.04
29	Canagliflozin	Can	905.0	test API	NA	4.37	3.43	3.52	N	227	94	93.33	NA	34.21
30	Ceritinib	Ceri	558.1	test API	5.09	5.39	5.10	5.81	C	49	117	31.62	121.79	197.83
31	Clopidogrel	Clp	419.9	test API	3.23	3.17	3.96	4.03	N	157	217	7.41	NA	10.50
32	Clotrimazol	Clt	344.8	test API	NA	3.92	NA	5.84	N-C	232	1250	20.42	NA	352.00
33	Dapagliflozin	Dap	408.9	test API	NA	2.31	2.34	2.11	N	23	2272	34.67	NA	111.13
34	Dasatinib	Das	523.5	test API	NA	2.99	3.56	4.01	N-C	227	117	3.39	NA	102.18
35	Dienogest	Die	311.4	test API	2.75	5.18	NA	2.31	N	13	26	93.33	NA	NA
36	Dolutegravir	Dol	441.4	test API	NA	NA	NA	1.10	N	227	34	NA	1000.00	1000.00
37	Duloxetine	Dul	297.4	test API	NA	3.47	NA	4.20	C	232	34	6.46	NA	137.20
38	Efavirenz	Efa	315.7	test API	3.33	3.17	NA	5.15	N	227	117	3.55	NA	1125.30
39	Ezetimibe	Eze	409.4	test API	4.20	4.92	4.37	4.56	N	227	23	21.38	NA	15.94
40	Fexofenadine	Fex	501.7	test API	NA	5.20	NA	2.94	Z	49	5	93.33	NA	457.80
41	Fingolimod	Fin	342.9	test API	2.79	3.28	5.50	4.06	C	13	23	4.27	NA	0.37
42	Hydrochlorothiazide	Hyd	297.7	test API	NA	1.72	0.09	-0.58	N	229	23	7.24	NA	35.87
43	Irbesartan	Irb	428.5	test API	NA	4.33	1.13	4.47	N-A	227	23	34.67	NA	10.43
44	Keto-desogestrel	Ket	324.5	test API	2.88	5.35	NA	3.60	N	13	23	97.72	NA	21.45
45	Lumiracoxib	Lum	293.7	test API	2.41	3.14	NA	4.31	A	49	117	4.57	NA	NA
46	Metformin	Met	164.6	test API	2.92	0.98	0.002	-0.92	C	707	49	4.79	NA	24.77
47	Mirtazapine	Mir	265.4	test API	4.11	2.81	2.78	3.21	N-C	71	101	14.79	170.74	55.00
48	Mometasone	Mom	521.4	test API	3.83	4.96	4.68	5.06	N	229	2272	147.91	NA	92.22
49	Naloxegol	Nal	651.8	test API	NA	4.94	NA	1.36	N-C	72	8	144.54	NA	NA
50	Nilotinib	Nil	529.5	test API	5.93	5.40	NA	5.36	N-C	49	23	3.55	40.26	1.41

51	Olanzapine	Ola	312.4	test API	NA	2.71	NA	3.39	N-C	72	67	45.71	NA	1.00
52	Omeprazole	Ome	345.4	test API	NA	2.97	NA	2.43	N	23	34	3.39	NA	4.59
53	Orlistat	Orl	495.7	test API	2.43	4.32	NA	8.11	N	13	23	12.88	5.31	18.47
54	Panobinostat	Pan	288.1	test API	4.52	1.79	NA	-3.85	Z	23	34	3.72	0.90	0.45
55	Pemetrexed	Pem	427.4	test API	2.14	NA	0.30	0.43	A	49	5	NA	NA	0.50
56	Pioglitazone	Pio	391.9	test API	3.20	4.14	3.35	3.40	N-A	227	34	6.61	NA	NA
57	Quetiapine	Que	383.5	test API	4.13	3.05	NA	2.81	N-C	23	94	5.25	NA	NA
58	Regorafenib	Reg	482.8	test API	5.14	5.18	NA	4.49	N	227	117	3.55	181.00	NA
59	Ridaforolimus	Rid	990.2	test API	5.47	NA	4.68	7.25	N	13	23	NA	NA	52.25
60	Rivastigmine	Riv	285.8	test API	2.61	2.87	1.00	2.41	C	87	20	4.68	NA	177.92
61	Rosuvastatin	Ros	481.5	test API	2.03	1.54	NA	1.92	A	157	22	3.39	NA	NA
62	Tadalafil	Tad	389.4	test API	NA	4.79	2.32	1.64	N	227	34	30.20	NA	90.50
63	Terbinafine	Ter	326.9	test API	4.01	3.27	5.20	5.53	C	2292	708	3.98	NA	22.27
64	Ticagrelor	Tic	522.6	test API	NA	3.23	4.02	2.28	N	227	94	35.48	NA	14.88
65	Valsartan	Val	435.5	test API	NA	2.77	5.80	5.27	C	227	23	7.41	NA	13.90
66	Vildagliptin	Vil	303.4	test API	NA	2.96	0.06	-0.22	C	13	26	229.09	NA	130.58
67	Vorinostat	Vor	264.3	test API	NA	3.09	1.42	2.00	A	13	8	3.55	NA	1.70
68	Asulam	Asu	230.2	test PPP	1.30	1.60	-0.27	-0.27	N	49	5	4.68	4.69	71.90
69	Bixafen	Bix	414.2	test PPP	3.59	2.54	4.04	4.04	N	227	117	3.55	365.00	NA
70	Carbendazim	Cam	191.2	test PPP	2.35	2.35	2.12	2.12	A	156	23	4.47	34.14	33.70
71	Carbetamide	Cae	236.3	test PPP	1.95	2.36	1.00	1.00	N	156	16	3.55	9.01	55.50
72	Chlorantraniliprole	Chl	483.1	test PPP	2.56	1.99	2.74	2.74	N	49	117	4.79	219.35	170.00
73	Clomazone	Clm	239.7	test PPP	2.48	2.44	2.54	2.54	N	87	202	3.31	39.07	54.00
74	Dimethenamid	Dme	275.8	test PPP	1.84	2.37	2.45	2.45	N	71	13	3.31	14.17	NA
75	Dimoxystrobin	Dmo	326.4	test PPP	2.69	2.64	3.12	3.12	N	227	26	3.39	271.46	NA
76	Flonicamid	Fln	229.2	test PPP	0.20	1.44	0.34	0.34	N	71	23	3.55	1.16	40.00
77	Fluopicolide	Flp	383.6	test PPP	2.51	3.38	3.13	3.13	N	71	217	3.55	379.50	1117.00
78	Flutianil	Flt	426.5	test PPP	4.55	3.28	3.10	3.10	N	157	23	4.68	335.83	607.00
79	Fluxapyroxad	Flx	381.3	test PPP	2.86	2.54	3.20	3.20	N	227	23	3.55	237.09	847.00
80	Iprovalicarb	Ipr	320.4	test PPP	2.03	3.19	3.20	3.20	N	49	26	4.57	5.77	181.00
81	Isofetamid	Isf	359.5	test PPP	2.69	3.37	3.63	3.63	N	227	23	2.57	44.08	NA

82	Isopyrazam	Isp	359.4	test PPP	3.38	4.14	4.44	4.44	N	227	23	5.25	258.01	628.00
83	Mesosulfuron-methyl	Mem	503.5	test PPP	1.96	1.65	NA	1.60	A	72	26	4.27	42.41	48.90
84	Napropamide	Nap	271.4	test PPP	2.92	3.24	3.25	3.25	N	157	34	4.27	342.99	316.00
85	Picoxystrobin	Pic	367.3	test PPP	2.98	2.94	3.78	3.78	N	157	34	5.75	23.76	56.00
86	Pinoxaden	Pin	400.5	test PPP	2.54	NA	3.20	5.08	N	NA	NA	NA	0.34	0.28
87	Proquinazid	Pro	372.2	test PPP	4.11	2.9	5.50	5.50	N	157	34	3.39	135.81	70.50
88	Pyroxsulam	Pyr	434.4	test PPP	1.52	1.51	-1.01	-1.01	A	227	34	5.13	3.90	NA
89	Sedaxane	Sed	331.4	test PPP	2.73	2.94	3.30	3.30	N	227	23	3.55	82.77	866.00
90	Spirotetramat	Spt	373.4	test PPP	2.46	2.14	2.93	2.93	N	227	23	4.27	0.38	0.78
91	Sulfoxaflor	Sul	277.3	test PPP	1.61	2.59	0.73	0.73	N	71	101	3.55	0.09	NA
92	Tembotrione	Tem	440.8	test PPP	1.90	1.98	2.11	2.11	A	71	70	3.55	12.40	108.00
93	Trinexapac-ethyl	Tri	252.3	test PPP	2.06	2.01	1.60	1.60	A	13	34	3.31	0.12	4.50
94	Valifenalate	Vlf	398.9	test PPP	2.88	2.41	NA	3.40	N	49	117	4.68	0.13	5.15

a ChemAxon properties were calculated in (https://chemicalize.com/). NA: value not available. DT50s from OECD 307 and OECD 308 are reported here as the geometric mean if there were more than on DT50 available from different tests in soil or water-sediment systems

Soil biodegradation and partition coefficient data for the PPPs and reference compounds were taken from EFSA assessment reports and the Pesticide Properties Database of the University of Hertfordshire[1] (PPDB) (http://sitem.herts.ac.uk/aeru/ppdb/en/) as stated in the previous publication [2]. The OECD 308 values for PPPs were obtained from the same sources, but some of the studies might have been conducted in relevant water-sediment conditions though not necessarily strictly according to OECD 308 test guidelines.

Biodegradation and partition coefficient data for the APIs were extracted from iPiEsum (https://ipiesys.eu/). As the platform does not support machine-readable download options, we searched the database for OECD 307 and 308 data using the search function of iPiEsum. Data obtained for the 46 APIs was then copied into text files, from where it was reformatted into comma-separated tables using a simple Python script. The output was manually curated and corrected where necessary, but in some cases the type and values of the DT50s remained unclear and they were therefore not considered further. Additional OECD 308 test information for APIs was requested directly from API industry partners of the PREMIER project (https://imi-premier.eu/) and thus all of the 46 APIs were included in the activated sludge assays under the assumption that they have a DT50. However, although they were tested in OECD 308, for dienogest, naloxegol, pioglitazone, quetiapine, regorafenib and rosuvastatin we ended up not being able to retrieve actual total system DT50 values. Therefore, the maximum number of APIs that could be used for read-across modelling was 40.

Partition coefficients for all compound sets were estimated using the K_{OC} model [3] of the OPERA tool [4] version 2.7 installed from GitHub (https://github.com/kmansouri/OPERA). OPERA takes SMILES as inputs and provides an estimate for the log-transformed partition coefficient, log K_{oc}. As shown in Table S1, OPERA could estimate partition coefficients for most PPPs, APIs and reference compounds with five exceptions: aliskiren, dolutegravir, pemetrexed, pinoxaden and ridaforolimus, for which OPERA did not provide any log K_{oc} estimate.

S2. Activated sludge experiments

S2.1 Experimental design

The experiments consisted of four different types of bottle incubations, all in amber 100 mL HACH bottles, filled with 50mL of activated sludge:
1) biotransformation incubations (BT) as main treatments to track biotransformation of chemicals from which $DegT50_{sludge}$ were derived.
2) abiotic controls (AB) to assess abiotic dissipation of chemicals in activated sludge supernatant. The supernatant was autoclaved before spiking the mix of reference and test compounds.
3) sorption controls (SC) to assess sorption processes to sludge. Sludge was autoclaved twice (consecutively) before spiking the mix of reference and test compounds.
4) unspiked controls (UC) to identify background levels.
$DegT50_{sludge}$ were not derived from abiotic or sorption controls. These controls were used for qualitative assessment of dissipation processes that could not be attributed to biotransformation or the presence of other microorganisms in the activated sludge. Most importantly, this helped

confirm that compounds were spiked even though they were not detected at any timepoint (for example, usually at HB levels) and to have an explanation for that observation.

Smaller variations in experimental design for each experiment with activated sludge are detailed in the following Table S2.

Table S2. Experimental design and variations on each of the experiments with activated sludge.

	**Spiked compounds**	**Initial concentration of compounds**	**Duration**	**pH-levels**	**Biomass levels and controls**	**Sampling timepoints**	**Source activated sludge**
**Exp 2018 summer**	20 reference compounds + 32 PPPs	5 µg/L	48 h	One level: 7.5	BT: Two biomass levels: HB, DB. Triplicate reactors. (NB excluded)<br><br>Controls: AB, SC, UC	BT: 0, 2, 4, 7, 15, 24, 30 and 48 h<br>UC: 0, 24<br>AB:0, 24, 48<br>SC: 0.12	WWTP Neugut
**Exp 2020 fall**	20 reference compounds	5 µg/L	48 h	Two levels: 6 and 7.5	BT: Two biomass levels: HB, DB. Duplicate reactors.<br><br>Controls: AB, SC, UC	BT: 0, 2, 4, 7, 15, 24, 30 and 48 h<br>UC: 0, 24, 48<br>AB: 0, 12, 24, 36<br>SC:0, 12, 24	WWTP Neugut
**Exp 2021 winter**	20 reference compounds	8 µg/L	48 h	Two levels: 6 and 7.5	BT: Two biomass levels: HB, DB. Triplicate reactors.<br><br>Controls: AB, SC, UC	BT: 0, 2, 4, 7, 15, 24, 30 and 48 h<br>UC: 0, 24, 48<br>AB:0, 36<br>SC:	WWTP Neugut
**Exp 2021 summer**	21 reference compounds	6 µg/L	72 h	Two levels: 6 and 7.5	BT: Sludges from two WWTP. One biomass level: HB sludge from NE and HB sludge from VH.<br>Duplicate reactors<br><br>Controls: AB, SC, UC	BT: 0, 2, 4, 7, 15, 24, 30 and 48 h<br>UC: 0, 24, 48<br>AB: 0, 24, 48<br>SC:	WWTP Neugut (NE) and Eawag-Experimental hall (VH)
**Exp 2021 fall**	21 reference compounds + 46 APIs	8 µg/L	72 h	Two levels: 6 and 7.5	BT: Two biomass levels: HB, DB. Duplicate reactors.<br><br>Controls: AB, SC, UC	BT: 0, 2, 4, 7, 15, 24, 30 and 48 h<br>UC: 2, 24, 48<br>AB: 0, 12, 24<br>SC: 0, 24	WWTP Neugut

The normal sludge biomass level was excluded from Exp2018 and not performed in subsequent experiments because the DB and HB biomass levels had been found to better capture the degradation kinetics of sorptive or fast degrading compounds (DB) and of medium to slowly degrading compounds (HB).

S2.2 Comparison of operational parameters of Eawag pilot WWTP plant in the experimental hall and WWTP Neugut

Eawag experimental hall (“Versuchshalle”, VH)
The pilot-scale WWTP in Versuchshalle at Eawag receives wastewater from the municipality of Dübendorf. It is operated as a Sequencing Batch Reactor (SBR). At the time of sampling the activated sludge, the biological reactor had a volume capacity of 8 m^3, it was continuously filled and emptied every 8-12h and operating under nitrifying/denitrifying conditions.

WWTP Neugut (NE)
WWTP Neugut is a full-scale WWTP serving the municipalities of Dübendorf, Dietlikon and Wangen-Brüttisellen in Switzerland with an average load of 50,000 population equivalents. The biological treatment includes phosphate removal, nitrification and denitrification. Activated sludge was collected from the nitrification biological reactor (volume 2,500 m^3).

S2.3 Preparation of sludge biomass solutions, TSS, pH and dissolved oxygen measurements

The high and dilute biomass levels (HB and DB) in activated sludge were prepared as follows:

- HB: centrifuging 3x700 mL of normal activated sludge collected from the WWTP and removing 400 mL of supernatant from each vessel, yielding 900 mL of HB sludge. For experiment 2018-summer, 6x500 mL were centrifuged and 400-500 mL of supernatant were removed overall.
- DB: adding 400 mL of supernatant from centrifugation of sludge to 100 mL of normal sludge, yielding 500 mL of DB sludge

Total suspended solids (TSS) measurements were carried out for the different biomass levels of sludge (HB and DB). A volume of 3-20 mL was vacuum-filtered through a 0.2 µm glass microfiber filter. The clean filters were weighed before sludge contact, and after filtering and drying at 105 °C for 24 h. The weight difference was used as the dry weight of suspended solids in sludge.

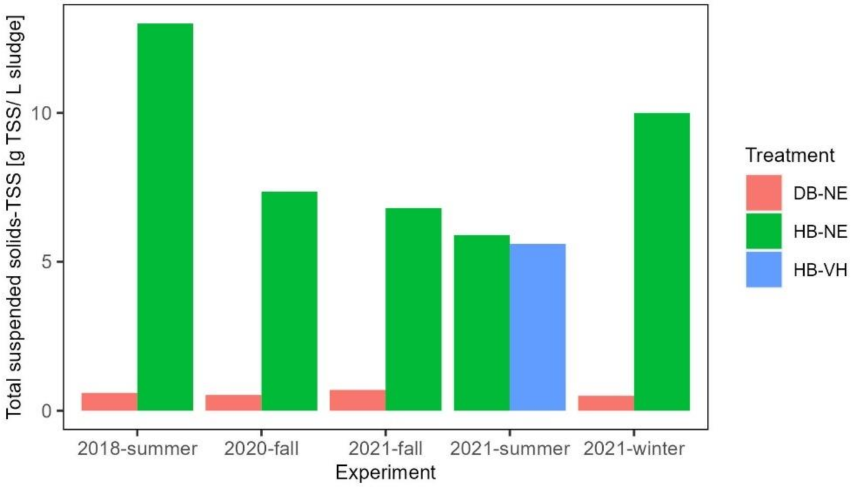

Figure S1. Total suspended solids- TSS in dry weight of sludge solids per liter of sludge for each experiment

The pH and dissolved oxygen levels in the biotransformation reactors were monitored using a portable meter (Hach HQD). The monitoring frequency varied for each experiment. pH was monitored more frequently than DO to tune the CO_2 bubbling. The pH was not monitored over time in experiment 2018-summer (single point measurements are reported in the previous publication[2]).

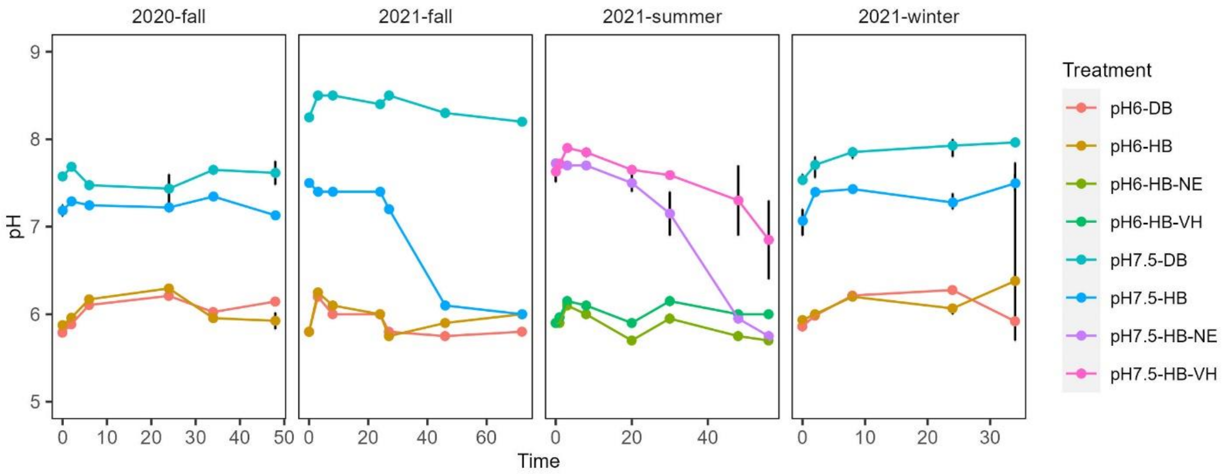

Figure S2. pH measurements in sludge experiments

On experiments 2021 summer and 2021-fall, which were performed with longer incubation times (72h instead of 48h), we observed a drop in pH at the pH7.5-HB sludge incubations from 7.5 to 6. We hypothesize that this drop of pH could be related to nitrification processes in the pH7.5-HB reactors. [5, 6] The acidic pH in the pH6-HB and pH6-DB reactors might inhibit first-step nitrification (conversion of ammonia to nitrites). [7] Both pH and oxygen levels can affect the ammonia oxidation and nitrite oxidation rate balance, [8] which in turn could affect other functions mediated by ammonia oxidizing and nitrite oxidizing bacteria (AOB and NOB). These AOB/NOB have been associated with biotransformation functions of some organic contaminants[9, 10] and this could be one factor influencing degradation kinetics in the different BT reactors.[11]

We could also expect some changes in the microbiome in activated sludge at the two pH levels. For example, under acidic conditions, the permeability of the protozoan cells may increase, raising energetic costs associated with osmoregulation and the amount of food (bacteria) needed by the protozoan in the activated sludge. Some protozoans sensitive to pH changes could also choose to encyst. [12].

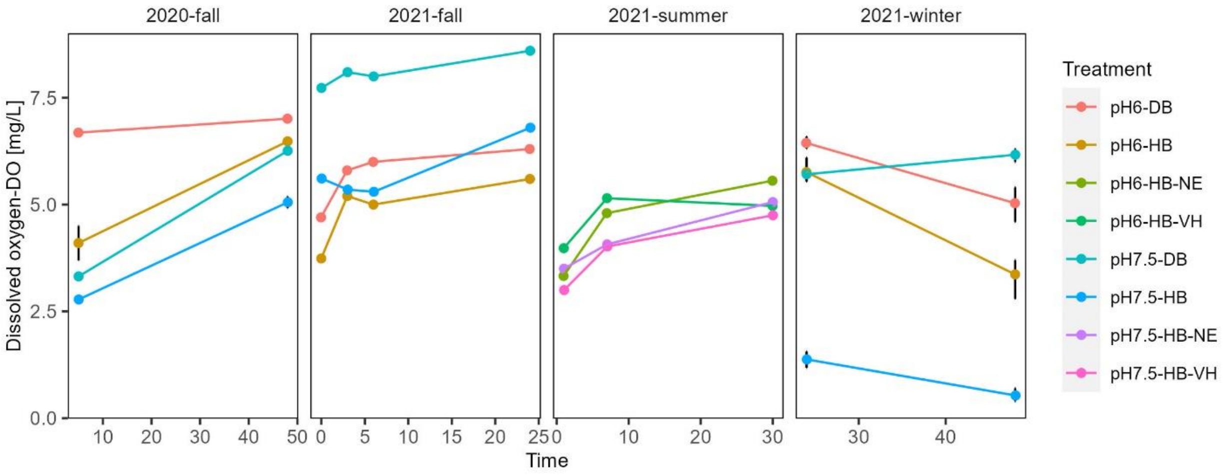

Figure S3. Dissolved oxygen- DO [mg/L] measurements in sludge experiments

Dissolved oxygen levels tended to be lower for the HB reactors at the higher pH levels. For experiment 2021-winter, the DO levels dropped below 0.5 mg/L. No measurements of DO in sludge reactors were performed in the experiment 2018-summer.

S3. Analytical method for LC-HRMS

Samples of all experiments were measured with the same liquid chromatography method using an *Atlantis T3* column (C18, 3µm, 3.0x150mm, *Waters*) with a *VanGuard* pre-column (C18, 5µm, 3.9x5mm, *Waters*). The mobile phase consisted of nanopure water (*NANOpure®* Diamond™ UV ultrapure water purification system D11911) and methanol (Optima™ LC/MS grade, Fisher Chemical, Fisher Scientific), each with 0.1 % formic acid (98-100%, Merck) at a flow rate of 300 µL/min. Initial conditions for the mobile phase of 95:5 water:methanol were maintained for 1 min. Afterwards a linear gradient was started to reach 5:95 water:methanol within 16 mins, which was maintained for 8 mins. This was followed by a rapid decrease within 0.1 min back to initial conditions kept for 4.9 mins.

Table S3. Differences in analytical method used in each experiment.

	**LC**	**ESI**	**HRMS**	**Compounds in inclusion list**	
**Exp 2018 summer**	Injection volume: 100 µL<br><br>Mobile phase water / methanol + 0.1% formic acid<br><br>Atlantis T3 column	Positive and negative	Full scan- polarity switching mode (no MS2 acquisition)	none	
**Exp 2020 fall**		Positive	Top 5- data dependent MS2 acquisition with inclusion list	20 reference compounds + reported TPs of PPPs formed in soil	
**Exp 2021 winter**		Positive	Top 5- data dependent MS2 acquisition with inclusion list	20 reference compounds + reported TPs of PPPs formed in soil	

**Exp 2021 summer**	Duration: 28 mins	Positive and negative	Top 5- data dependent MS2 acquisition with inclusion list	21 reference compounds + reported TPs of PPPs formed in soil	
**Exp 2021 fall**		Positive and negative	Top 5- data dependent MS2 acquisition with inclusion list	21 reference compounds + 46 APIs + enviPath-predicted TPs of APIs	

The limits of quantification (LOQ) were determined as the calibration standard with the lowest concentration that had a distinguishable peak with S/N ratio of >3:1 and for which the calibration curve estimated concentration had less than 50% error.

Table S4. Limits of quantification (LOQ) in the LC-HRMS measurements for each compound and experiment in µg/L. na: compound not added, nf: compound not found.

Compound	Compound set	2018-summer	2020-fall	2021-winter	2021-summer	2021-fall
Azoxystrobin	reference	0.3	0.1	0.1	0.5	0.5
Benzovindiflupyr	reference	0.3	0.5	0.5	0.2	0.5
Bromoxynil	reference	*na*	*na*	*na*	0.5	0.5
Cyantraniliprole	reference	0.3	0.05	0.1	0.1	0.05
Cyclaniliprole	reference	0.3	0.2	0.2	0.2	0.2
Dicamba	reference	0.3	*na*	*na*	0.2	0.1
Diuron	reference	0.3	0.2	0.1	0.1	0.05
Fenhexamid	reference	0.3	0.05	0.1	0.05	0.05
Fenoxycarb	reference	0.3	0.2	0.2	0.2	0.2
Fipronil	reference	0.3	0.5	0.5	0.5	0.5
Florasulam	reference	0.3	0.05	0.1	0.05	0.05
Fluopyram	reference	0.3	0.05	0.1	0.05	0.05
Flupyradifurone	reference	0.3	0.2	0.1	0.2	0.05
Imidacloprid	reference	0.3	0.1	0.2	0.05	0.05
Isoproturon	reference	0.3	0.2	0.1	0.1	0.05
Kresoxim-methyl	reference	0.3	0.2	*nf*	0.2	0.1
Mandipropamid	reference	0.3	0.2	0.2	0.2	0.2
Mesotrione	reference	0.3	0.2	0.2	0.05	0.1
Oxathiapiprolin	reference	0.3	0.1	0.1	0.05	0.1
Terbuthylazine	reference	0.3	0.2	0.1	0.1	0.1
Topramezone	reference	0.3	0.2	0.1	0.2	*nf*
Acalabrutinib	test API	*na*	*na*	*na*	*na*	0.5
Aliskiren	test API	*na*	*na*	*na*	*na*	0.1
Amlodipine	test API	*na*	*na*	*na*	*na*	0.1
Atazanavir	test API	*na*	*na*	*na*	*na*	0.2
Atomoxetine	test API	*na*	*na*	*na*	*na*	0.05
Atovaquone	test API	*na*	*na*	*na*	*na*	0.1
Budesonide	test API	*na*	*na*	*na*	*na*	0.2
Canagliflozin	test API	*na*	*na*	*na*	*na*	0.1
Ceritinib	test API	*na*	*na*	*na*	*na*	0.2
Clopidogrel	test API	*na*	*na*	*na*	*na*	0.05
Clotrimazol	test API	*na*	*na*	*na*	*na*	0.1
Dapagliflozin	test API	*na*	*na*	*na*	*na*	0.2
Dasatinib	test API	*na*	*na*	*na*	*na*	0.05
Dienogest	test API	*na*	*na*	*na*	*na*	0.2
Dolutegravir	test API	*na*	*na*	*na*	*na*	0.1
Duloxetine	test API	*na*	*na*	*na*	*na*	0.05
Efavirenz	test API	*na*	*na*	*na*	*na*	0.1

Ezetimibe	test API	*na*	*na*	*na*	*na*	0.2
Fexofenadine	test API	*na*	*na*	*na*	*na*	0.05
Fingolimod	test API	*na*	*na*	*na*	*na*	0.2
Hydrochlorothiazide	test API	*na*	*na*	*na*	*na*	0.05
Irbesartan	test API	*na*	*na*	*na*	*na*	0.05
Keto-desogestrel	test API	*na*	*na*	*na*	*na*	0.2
Lumiracoxib	test API	*na*	*na*	*na*	*na*	0.1
Metformin	test API	*na*	*na*	*na*	*na*	0.5
Mirtazapine	test API	*na*	*na*	*na*	*na*	0.05
Mometasone	test API	*na*	*na*	*na*	*na*	0.2
Naloxegol	test API	*na*	*na*	*na*	*na*	0.05
Nilotinib	test API	*na*	*na*	*na*	*na*	0.05
Olanzapine	test API	*na*	*na*	*na*	*na*	0.2
Omeprazole	test API	*na*	*na*	*na*	*na*	0.5
Orlistat	test API	*na*	*na*	*na*	*na*	0.5
Panobinostat	test API	*na*	*na*	*na*	*na*	0.2
Pemetrexed	test API	*na*	*na*	*na*	*na*	0.05
Pioglitazone	test API	*na*	*na*	*na*	*na*	0.2
Quetiapine	test API	*na*	*na*	*na*	*na*	0.05
Regorafenib	test API	*na*	*na*	*na*	*na*	0.05
Ridaforolimus	test API	*na*	*na*	*na*	*na*	
Rivastigmine	test API	*na*	*na*	*na*	*na*	0.05
Rosuvastatin	test API	*na*	*na*	*na*	*na*	0.05
Tadalafil	test API	*na*	*na*	*na*	*na*	0.2
Terbinafine	test API	*na*	*na*	*na*	*na*	0.05
Ticagrelor	test API	*na*	*na*	*na*	*na*	0.2
Valsartan	test API	*na*	*na*	*na*	*na*	0.05
Vildagliptin	test API	*na*	*na*	*na*	*na*	0.05
Vorinostat	test API	*na*	*na*	*na*	*na*	0.05
Asulam	test PPP	0.3	*na*	*na*	*na*	*na*
Bixafen	test PPP	0.3	*na*	*na*	*na*	*na*
Carbendazim	test PPP	0.3	*na*	*na*	*na*	*na*
Carbetamide	test PPP	0.3	*na*	*na*	*na*	*na*
Chlorantraniliprole	test PPP	0.3	*na*	*na*	*na*	*na*
Clomazone	test PPP	0.3	*na*	*na*	*na*	*na*
Dimethenamid	test PPP	0.3	*na*	*na*	*na*	*na*
Dimoxystrobin	test PPP	0.3	*na*	*na*	*na*	*na*
Flonicamid	test PPP	0.3	*na*	*na*	*na*	*na*
Fluopicolide	test PPP	0.3	*na*	*na*	*na*	*na*
Flutianil	test PPP	0.3	*na*	*na*	*na*	*na*
Fluxapyroxad	test PPP	0.3	*na*	*na*	*na*	*na*
Iprovalicarb	test PPP	0.3	*na*	*na*	*na*	*na*
Isofetamid	test PPP	0.3	*na*	*na*	*na*	*na*
Isopyrazam	test PPP	0.3	*na*	*na*	*na*	*na*
Mesosulfuron-methyl	test PPP	0.3	*na*	*na*	*na*	*na*
Napropamide	test PPP	0.3	*na*	*na*	*na*	*na*
Picoxystrobin	test PPP	0.3	*na*	*na*	*na*	*na*
Proquinazid	test PPP	0.3	*na*	*na*	*na*	*na*
Pyroxsulam	test PPP	0.3	*na*	*na*	*na*	*na*
Sedaxane	test PPP	0.3	*na*	*na*	*na*	*na*
Spirotetramat	test PPP	0.3	*na*	*na*	*na*	*na*
Sulfoxaflor	test PPP	0.3	*na*	*na*	*na*	*na*
Tembotrione	test PPP	0.3	*na*	*na*	*na*	*na*
Trinexapac-ethyl	test PPP	0.3	*na*	*na*	*na*	*na*
Valifenalate	test PPP	0.3	*na*	*na*	*na*	*na*

Internal standards

One mix of diverse isotopically labelled versions of commercial PPPs was added to all samples in all experiments. In addition, a second mix containing isotopically labelled versions of commercial APIs was added to the experiment 2021-fall. As the isotopically labelled version of most test APIs and a a few PPPs were not available, internal standards were selected based on the similarity in analyte retention times and a low relative standard deviation of their areas across all samples (RSD < 7%).

Table S5. Internal standards added to each experiment to perform semi-quantification of reference and test compounds.

Name	2018-summer	2020-fall	2021-winter	2021-summer	2021-fall
2,4-D-D3	2ug/L	2ug/L	4ug/L	4ug/L	4ug/L
2,6-Dichlorbenzamid-3,4,5-D3	2ug/L	2ug/L	4ug/L	4ug/L	4ug/L
Aclonifen-D5	2ug/L	2ug/L	4ug/L	4ug/L	4ug/L
Alachlor-D13	2ug/L	2ug/L	4ug/L	4ug/L	4ug/L
Aldicarb (N-methyl-13C-D3-carbamoyl-13C)	2ug/L	2ug/L	4ug/L	4ug/L	4ug/L
Atrazin-D5	2ug/L	2ug/L	4ug/L	4ug/L	4ug/L
Atrazin-Desethyl-15N3	2ug/L	2ug/L	4ug/L	4ug/L	4ug/L
Atrazin-2-Hydroxy-D5	2ug/L	2ug/L	4ug/L	4ug/L	4ug/L
Atrazin-desisopropyl-D5	2ug/L	2ug/L	4ug/L	4ug/L	4ug/L
Azoxystrobin-d4	2ug/L	2ug/L	4ug/L	4ug/L	4ug/L
Bentazon-D6	2ug/L	2ug/L	4ug/L	4ug/L	4ug/L
Boscalid-D4	2ug/L	2ug/L	4ug/L	4ug/L	4ug/L
Bupirimate-D5	2ug/L	2ug/L	4ug/L	4ug/L	4ug/L
Carbendazim-D4	2ug/L	2ug/L	4ug/L	4ug/L	4ug/L
Chloridazon-D5	2ug/L	2ug/L	4ug/L	4ug/L	4ug/L
Chloridazon-desphenyl-15N2	2ug/L	2ug/L	4ug/L	4ug/L	4ug/L
Chloridazon-methyl-desphenyl-D3	2ug/L	2ug/L	4ug/L	4ug/L	4ug/L
Chlorotoluron-D6	2ug/L	2ug/L	4ug/L	4ug/L	4ug/L
Chlorpyrifos-D10	2ug/L	2ug/L	4ug/L	4ug/L	4ug/L
Chlorpyrifos-methyl-D6	2ug/L	2ug/L	4ug/L	4ug/L	4ug/L
Clothianidin-D3	2ug/L	2ug/L	4ug/L	4ug/L	4ug/L
Cyprodinil-D5	2ug/L	2ug/L	4ug/L	4ug/L	4ug/L
Diazinon-D10	2ug/L	2ug/L	4ug/L	4ug/L	4ug/L
Dicamba-D3	2ug/L	2ug/L	4ug/L	4ug/L	4ug/L
Dichlorprop-D6	2ug/L	2ug/L	4ug/L	4ug/L	4ug/L
Diflufenican-D3	2ug/L	2ug/L	4ug/L	4ug/L	4ug/L
Dimethenamid-D3	2ug/L	2ug/L	4ug/L	4ug/L	4ug/L
Dimethoate-D6	2ug/L	2ug/L	4ug/L	4ug/L	4ug/L
Diuron-D6	2ug/L	2ug/L	4ug/L	4ug/L	4ug/L
Epoxyconazole-D4	2ug/L	2ug/L	4ug/L	4ug/L	4ug/L
Fenhexamid-D3	2ug/L	2ug/L	4ug/L	4ug/L	4ug/L
Fipronil-13C2 15N2	2ug/L	2ug/L	4ug/L	4ug/L	4ug/L
(+-)-Fluazifop-D4	2ug/L	2ug/L	4ug/L	4ug/L	4ug/L
Fludioxonil-13C3	2ug/L	2ug/L	4ug/L	4ug/L	4ug/L
Flufenacet-D4	2ug/L	2ug/L	4ug/L	4ug/L	4ug/L
Fluopyram-D4	2ug/L	2ug/L	4ug/L	4ug/L	4ug/L
Haloxyfop-D4	2ug/L	2ug/L	4ug/L	4ug/L	4ug/L
Hexazinon-D6	2ug/L	2ug/L	4ug/L	4ug/L	4ug/L
Imidacloprid-D4	2ug/L	2ug/L	4ug/L	4ug/L	4ug/L
Irgarol-D9	2ug/L	2ug/L	4ug/L	4ug/L	4ug/L
Isoproturon-D6	2ug/L	2ug/L	4ug/L	4ug/L	4ug/L
Lenacil-D4	2ug/L	2ug/L	4ug/L	4ug/L	4ug/L
Linuron-D6	2ug/L	2ug/L	4ug/L	4ug/L	4ug/L
MCPA-D3	2ug/L	2ug/L	4ug/L	4ug/L	4ug/L

MCPB-D6	2ug/L	2ug/L	4ug/L	4ug/L	4ug/L
Mecoprop-D6	2ug/L	2ug/L	4ug/L	4ug/L	4ug/L
Mesotrion-D3	2ug/L	2ug/L	4ug/L	4ug/L	4ug/L
Metalaxyl-D6	2ug/L	2ug/L	4ug/L	4ug/L	4ug/L
Metamitron-D5	2ug/L	2ug/L	4ug/L	4ug/L	4ug/L
Metazachlor-D6	2ug/L	2ug/L	4ug/L	4ug/L	4ug/L
Metconazole-D6	2ug/L	2ug/L	4ug/L	4ug/L	4ug/L
Methiocarb-D3	2ug/L	2ug/L	4ug/L	4ug/L	4ug/L
Methomyl-D3	2ug/L	2ug/L	4ug/L	4ug/L	4ug/L
Methoxyfenozid-D9	2ug/L	2ug/L	4ug/L	4ug/L	4ug/L
Metolachlor-D6	2ug/L	2ug/L	4ug/L	4ug/L	4ug/L
Metolachlor -ESA-D11	2ug/L	2ug/L	4ug/L	4ug/L	4ug/L
Metrafenone-D9	2ug/L	2ug/L	4ug/L	4ug/L	4ug/L
Metribuzin-D3	2ug/L	2ug/L	4ug/L	4ug/L	4ug/L
Metsulfuron-methyl-D3	2ug/L	2ug/L	4ug/L	4ug/L	4ug/L
Monolinuron-D6	2ug/L	2ug/L	4ug/L	4ug/L	4ug/L
Myclobutanil-D4	2ug/L	2ug/L	4ug/L	4ug/L	4ug/L
N,N-diethyl-3-methylbenzamide-D10 (DEET-D10)	2ug/L	2ug/L	4ug/L	4ug/L	4ug/L
Nicosulfuron-D6	2ug/L	2ug/L	4ug/L	4ug/L	4ug/L
Octilinone-D17	2ug/L	2ug/L	4ug/L	4ug/L	4ug/L
Pirimicarb-D6	2ug/L	2ug/L	4ug/L	4ug/L	4ug/L
Prochloraz-D7	2ug/L	2ug/L	4ug/L	4ug/L	4ug/L
Propachlor-ESA-D5	2ug/L	2ug/L	4ug/L	4ug/L	4ug/L
Propachlor-OXA-D5	2ug/L	2ug/L	4ug/L	4ug/L	4ug/L
Propamocarb-D7	2ug/L	2ug/L	4ug/L	4ug/L	4ug/L
Propazin-D6	2ug/L	2ug/L	4ug/L	4ug/L	4ug/L
Propiconazol-D7	2ug/L	2ug/L	4ug/L	4ug/L	4ug/L
Propyzamide-D3	2ug/L	2ug/L	4ug/L	4ug/L	4ug/L
Pyraclostrobin-D3	2ug/L	2ug/L	4ug/L	4ug/L	4ug/L
Pyrimethanil-D5	2ug/L	2ug/L	4ug/L	4ug/L	4ug/L
Simazin-D5	2ug/L	2ug/L	4ug/L	4ug/L	4ug/L
Sulcotrion-D3	2ug/L	2ug/L	4ug/L	4ug/L	4ug/L
Tebuconazole-D9	2ug/L	2ug/L	4ug/L	4ug/L	4ug/L
Terbutryn-D5	2ug/L	2ug/L	4ug/L	4ug/L	4ug/L
Terbutylazin-D5	2ug/L	2ug/L	4ug/L	4ug/L	4ug/L
Thiacloprid-D4	2ug/L	2ug/L	4ug/L	4ug/L	4ug/L
Thiamethoxame-D3	2ug/L	2ug/L	4ug/L	4ug/L	4ug/L
Triclocarban-13C6	2ug/L	2ug/L	4ug/L	4ug/L	4ug/L
2',2'-Difluoro-2-deoxyuridin-13C,15N2	*na*	*na*	*na*	*na*	4ug/L
5-Fluorouracil-15N2C13	*na*	*na*	*na*	*na*	4ug/L
5-Metyhl-1H-benzotriazole-D6	*na*	*na*	*na*	*na*	4ug/L
Amisulpride-d5	*na*	*na*	*na*	*na*	4ug/L
Atenolol acid-d5 (Metoprolol acid-d5)	*na*	*na*	*na*	*na*	4ug/L
Atenolol-D7	*na*	*na*	*na*	*na*	4ug/L
Atomoxetine-d3	*na*	*na*	*na*	*na*	4ug/L
Atorvastatin-d5	*na*	*na*	*na*	*na*	4ug/L
Azithromycin-D3	*na*	*na*	*na*	*na*	4ug/L
Benzotriazol-D4	*na*	*na*	*na*	*na*	4ug/L
Bezafibrat-D4	*na*	*na*	*na*	*na*	4ug/L
Bicalutamid-d4	*na*	*na*	*na*	*na*	4ug/L
Bisphenol A-D4	*na*	*na*	*na*	*na*	4ug/L
Candesartan-d5	*na*	*na*	*na*	*na*	4ug/L
Carbamazepin 10,11-epoxide-d8	*na*	*na*	*na*	*na*	4ug/L
Carbamazepin-D8	*na*	*na*	*na*	*na*	4ug/L
Carbidopa-d5	*na*	*na*	*na*	*na*	4ug/L
Citalopram-d6	*na*	*na*	*na*	*na*	4ug/L
Clarithromycin-D3	*na*	*na*	*na*	*na*	4ug/L
Climbazol-D4	*na*	*na*	*na*	*na*	4ug/L
Clofibrinsäure-D4	*na*	*na*	*na*	*na*	4ug/L

Clotrimazol-D5	*na*	*na*	*na*	*na*	4ug/L
Clozapine-D8	*na*	*na*	*na*	*na*	4ug/L
Codeine-D6	*na*	*na*	*na*	*na*	4ug/L
Coffein-D9	*na*	*na*	*na*	*na*	4ug/L
Cyclophosphamid-D4	*na*	*na*	*na*	*na*	4ug/L
Darunavir-d9	*na*	*na*	*na*	*na*	4ug/L
Diazepam-D5	*na*	*na*	*na*	*na*	4ug/L
Diclofenac-D4	*na*	*na*	*na*	*na*	4ug/L
Doxycycline-d3	*na*	*na*	*na*	*na*	4ug/L
Emtricitabine-13C,15N2	*na*	*na*	*na*	*na*	4ug/L
Eprosartan-D3	*na*	*na*	*na*	*na*	4ug/L
Erythromycin-13C2,d3	*na*	*na*	*na*	*na*	4ug/L
Fenofibrate-D6	*na*	*na*	*na*	*na*	4ug/L
Fluconazol-D4	*na*	*na*	*na*	*na*	4ug/L
Fluoxetine-D5	*na*	*na*	*na*	*na*	4ug/L
Furosemid-D5	*na*	*na*	*na*	*na*	4ug/L
Gabapentin-D4	*na*	*na*	*na*	*na*	4ug/L
Gemcitabine-13C,15N2	*na*	*na*	*na*	*na*	4ug/L
Hydrochlorothiazid-C13, D2	*na*	*na*	*na*	*na*	4ug/L
Hydromorphon-D3	*na*	*na*	*na*	*na*	4ug/L
Ibuprofen-D3	*na*	*na*	*na*	*na*	4ug/L
Indomethacin-D4	*na*	*na*	*na*	*na*	4ug/L
Irbesartan-D4	*na*	*na*	*na*	*na*	4ug/L
Isoniazid-d4	*na*	*na*	*na*	*na*	4ug/L
Lamotrigin-13C3,d3	*na*	*na*	*na*	*na*	4ug/L
Levetiracetam-d3	*na*	*na*	*na*	*na*	4ug/L
Lidocain-D10	*na*	*na*	*na*	*na*	4ug/L
Meclizine-d8 (=Meclozine)	*na*	*na*	*na*	*na*	4ug/L
Mefenamicacid-D3	*na*	*na*	*na*	*na*	4ug/L
Metformin-D6	*na*	*na*	*na*	*na*	4ug/L
Methylprednisolon-D3	*na*	*na*	*na*	*na*	4ug/L
Metoprolol-D7	*na*	*na*	*na*	*na*	4ug/L
Metronidazol-D4	*na*	*na*	*na*	*na*	4ug/L
Morphine-D3	*na*	*na*	*na*	*na*	4ug/L
N4-Acetyl-Sulfamethoxazol-D5	*na*	*na*	*na*	*na*	4ug/L
N4-Acetyl-Sulfathiazol-D4	*na*	*na*	*na*	*na*	4ug/L
Naproxen-d3	*na*	*na*	*na*	*na*	4ug/L
Nelfinavir-d3	*na*	*na*	*na*	*na*	4ug/L
O-Desmethylvenlafaxin-D6	*na*	*na*	*na*	*na*	4ug/L
Oxazepam-D5	*na*	*na*	*na*	*na*	4ug/L
Oxcarbazepine-d4	*na*	*na*	*na*	*na*	4ug/L
Oxycodone-d6	*na*	*na*	*na*	*na*	4ug/L
Paracetamol-D4 (Acetaminophen-d4)	*na*	*na*	*na*	*na*	4ug/L
Phenazon-D3 (Antipyrin-d3)	*na*	*na*	*na*	*na*	4ug/L
Pravastatin-d3	*na*	*na*	*na*	*na*	4ug/L
Primidon-D5	*na*	*na*	*na*	*na*	4ug/L
Propranolol-D7	*na*	*na*	*na*	*na*	4ug/L
Ranitidin-D6	*na*	*na*	*na*	*na*	4ug/L
Ritalinsäure-D10	*na*	*na*	*na*	*na*	4ug/L
Ritonavir-D6	*na*	*na*	*na*	*na*	4ug/L
Roxithromycin-d7	*na*	*na*	*na*	*na*	4ug/L
Sotalol-D6	*na*	*na*	*na*	*na*	4ug/L
Sulfadiazin-D4	*na*	*na*	*na*	*na*	4ug/L
Sulfadimethoxin-D4	*na*	*na*	*na*	*na*	4ug/L
Sulfamethazine-d4	*na*	*na*	*na*	*na*	4ug/L
Sulfamethoxazol-D4	*na*	*na*	*na*	*na*	4ug/L
Sulfapyridin-D4	*na*	*na*	*na*	*na*	4ug/L
Sulfathiazol-D4	*na*	*na*	*na*	*na*	4ug/L
Sulpride-D3	*na*	*na*	*na*	*na*	4ug/L

Telmisartan-D3	*na*	*na*	*na*	*na*	4ug/L
Tramadol-D6	*na*	*na*	*na*	*na*	4ug/L
Trimethoprim-D3	*na*	*na*	*na*	*na*	4ug/L
Valsartan-13C5,15N	*na*	*na*	*na*	*na*	4ug/L
Valsartansäure-D4	*na*	*na*	*na*	*na*	4ug/L
Venlafaxin-D6	*na*	*na*	*na*	*na*	4ug/L
Venlafaxine-N,O-didesmethyl-D3	*na*	*na*	*na*	*na*	4ug/L
Verapamil-D6	*na*	*na*	*na*	*na*	4ug/L

S4. Time series of tested compounds

S4.1 Reference compounds

Dicamba concentration-time series were obtained in the 3 experiments where measurements were also performed in negative mode, whereas bromoxynil has data for the two experiments where it was added to the spike solution. Topramezone was not detected in the last experiment, possibly due to degradation in the spike solution.

Incubations containing sludge at high biomass concentrations (HB) consistently produced shorter $DegT50_{sludge}$ of all reference compounds than incubations with dilute biomass (DB). Higher biomass can be expected to result in higher amounts of degrading microorganisms, which explains the shorter $DegT50_{sludge}$. However, the dilute biomass level was useful to assess the kinetics of three fast degrading compounds (kresoxim-methyl, mandipropamid and oxathiapiprolin), which had most (>80%) $DegT50_{sludge}$ <0.5 days in high biomass incubations.

Substantial sorption to biomass organic carbon was observed for benzovindiflupyr, where there was a close to two-fold difference in concentrations between abiotic control levels (4-6 ug/L) and initial concentrations in the sorption and biotransformation reactors (~2-3 ug/L) at dilute biomass levels, and an almost six-fold difference at high biomass (i.e., initial concentrations in sorption and biotransformation reactors < 0.5 ug/L).

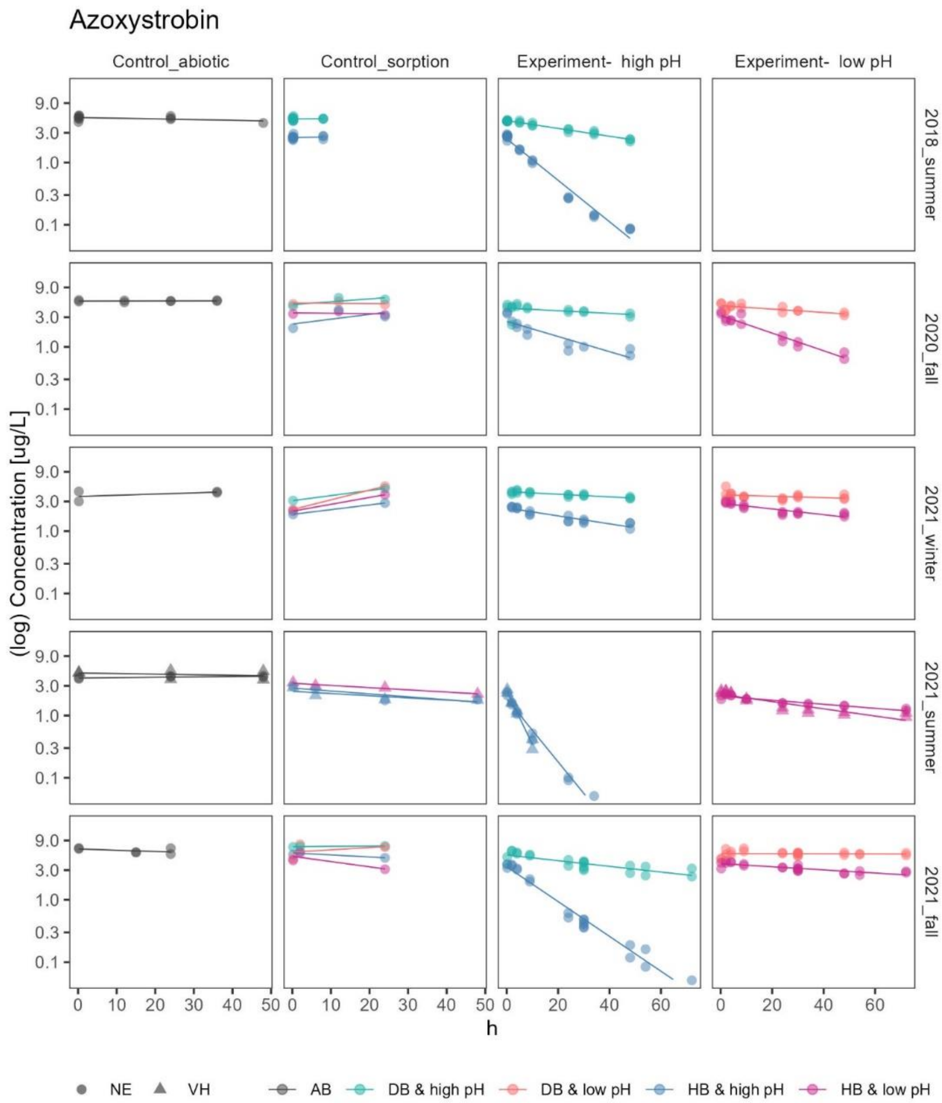

Figure S4. Timeseries of semi-quantified concentrations of azoxystrobin in all 5 experiments

408

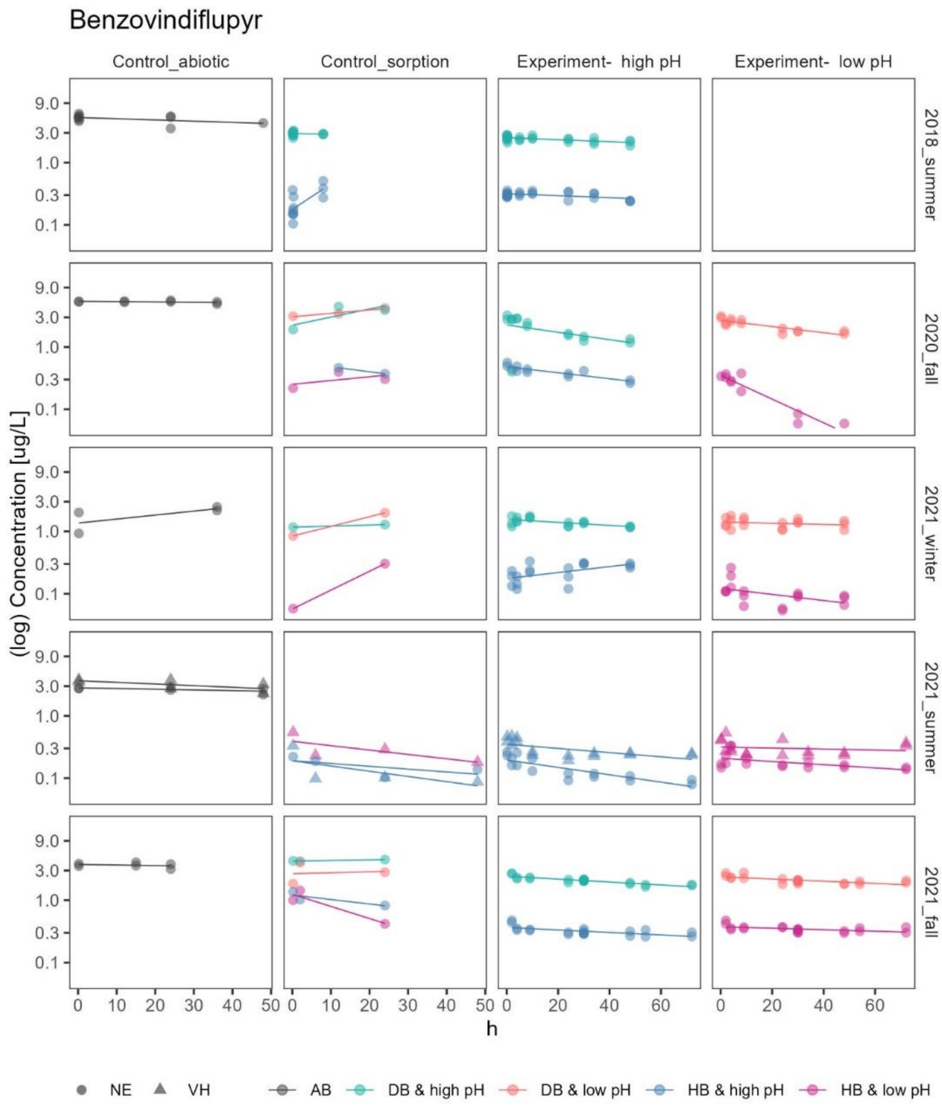

409 **Figure S5. Timeseries of semi-quantified concentrations of benzovindiflupyr in all 5 experiments**

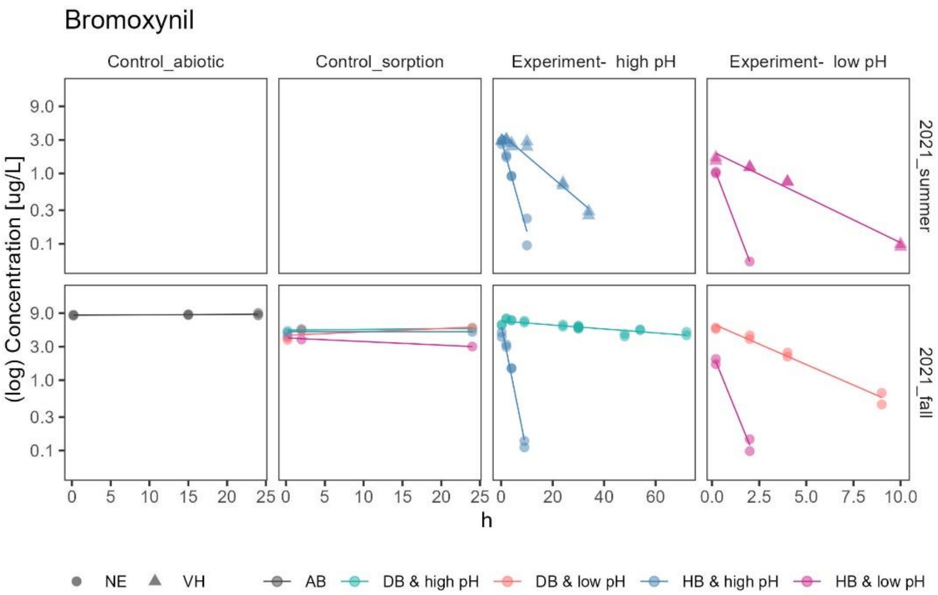

Figure S6. Timeseries of semi-quantified concentrations of bromoxynil in the 2 experiments where it was added to spike solution.

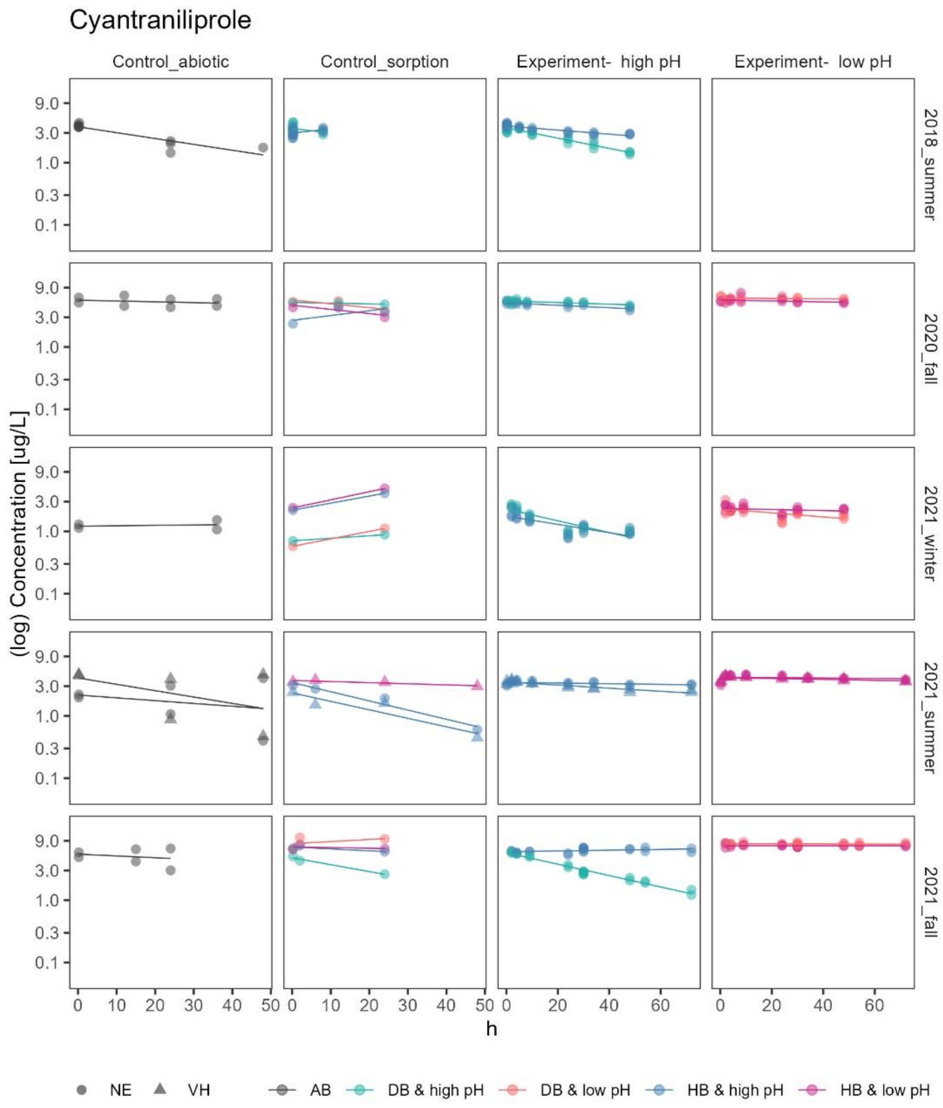

414

415 **Figure S7. Timeseries of semi-quantified concentrations of cyantraniliprole in all 5 experiments**

416

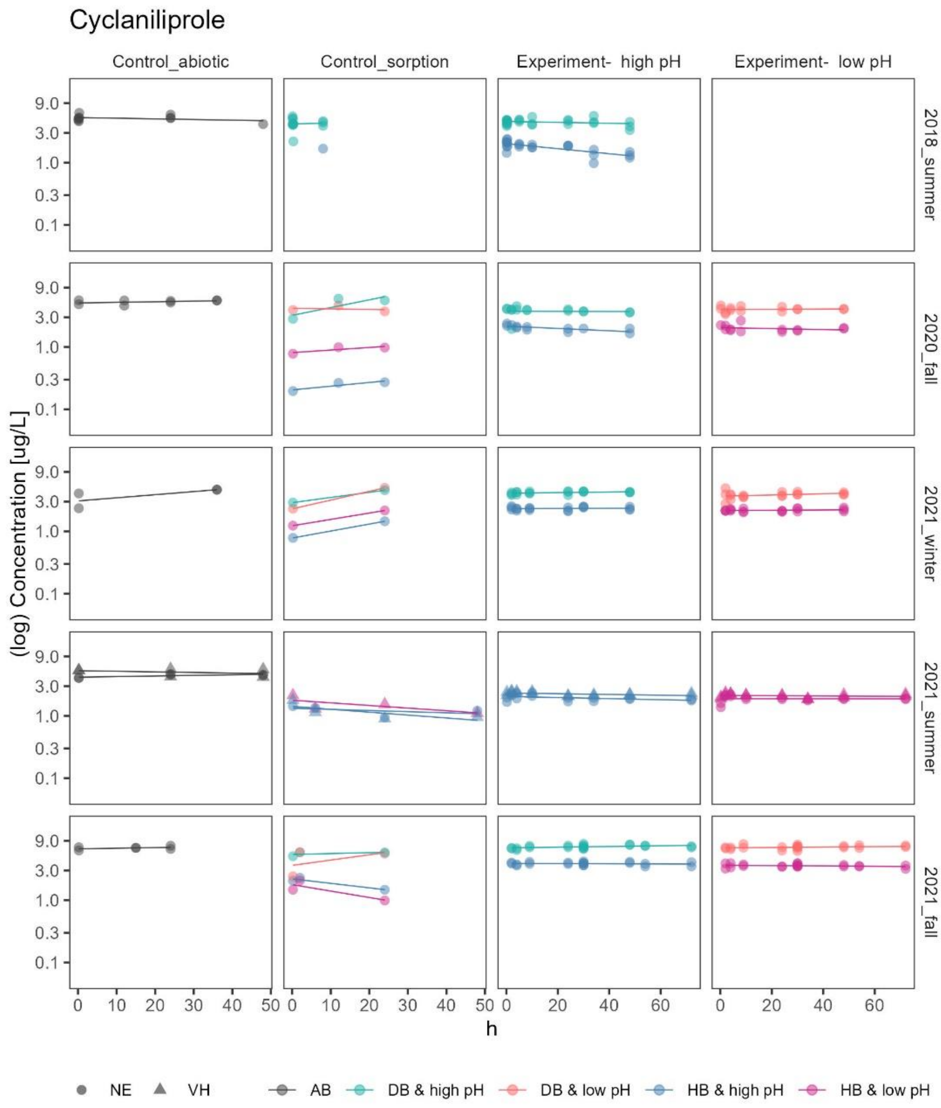

Figure S8. Timeseries of semi-quantified concentrations of cyclaniliprole in all 5 experiments

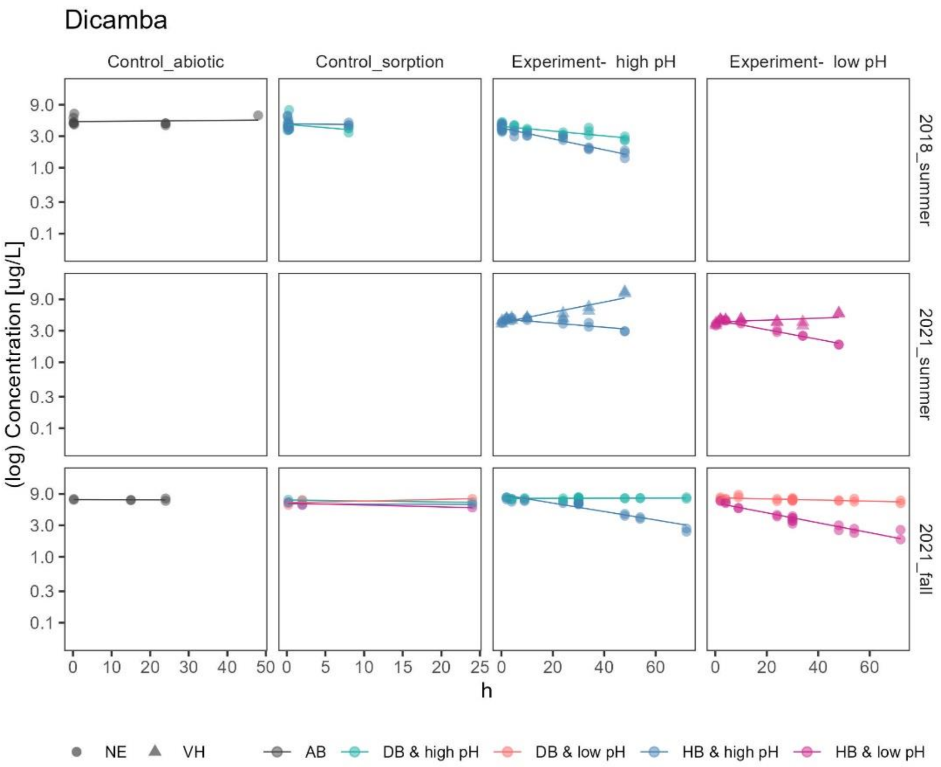

Figure S9. Timeseries of semi-quantified concentrations of dicamba in the 3 experiments where measurements were made in ESI negative mode.

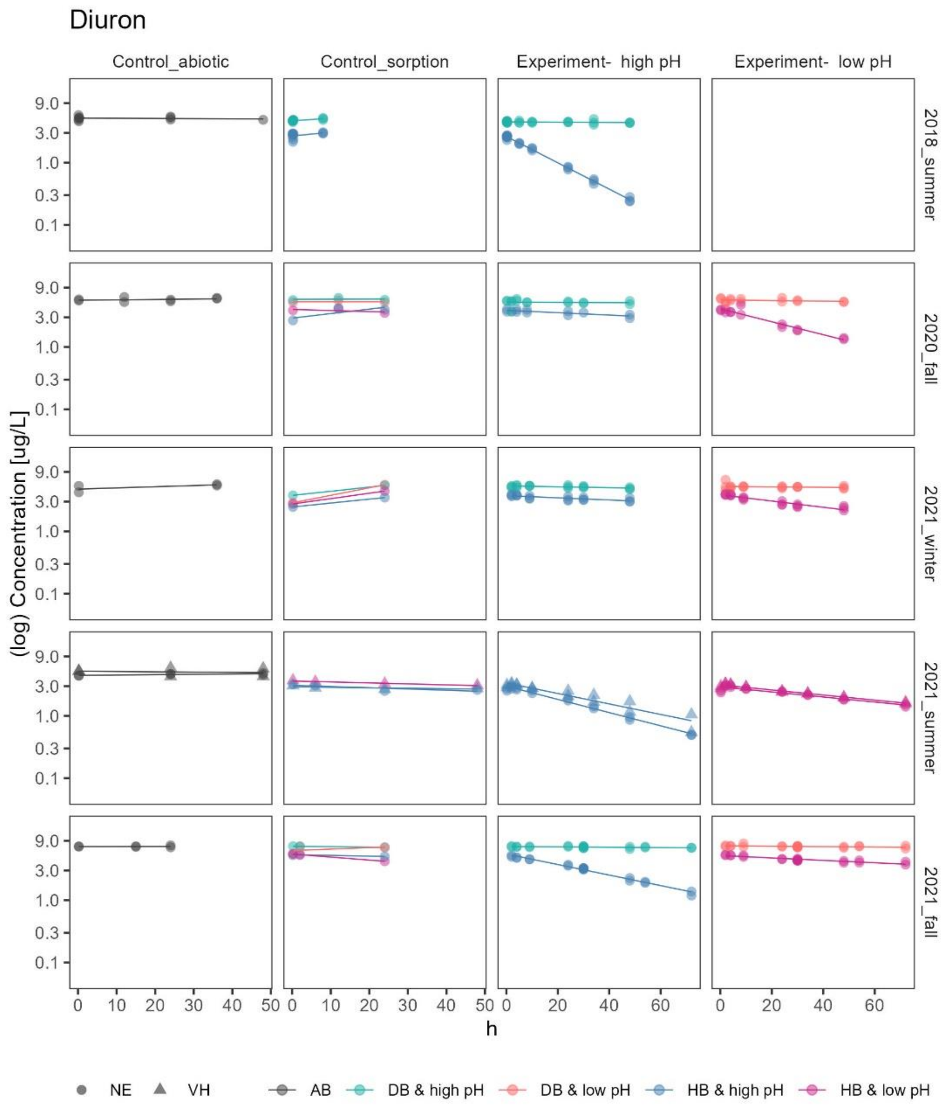

Figure S10. Timeseries of semi-quantified concentrations of diuron in all 5 experiments

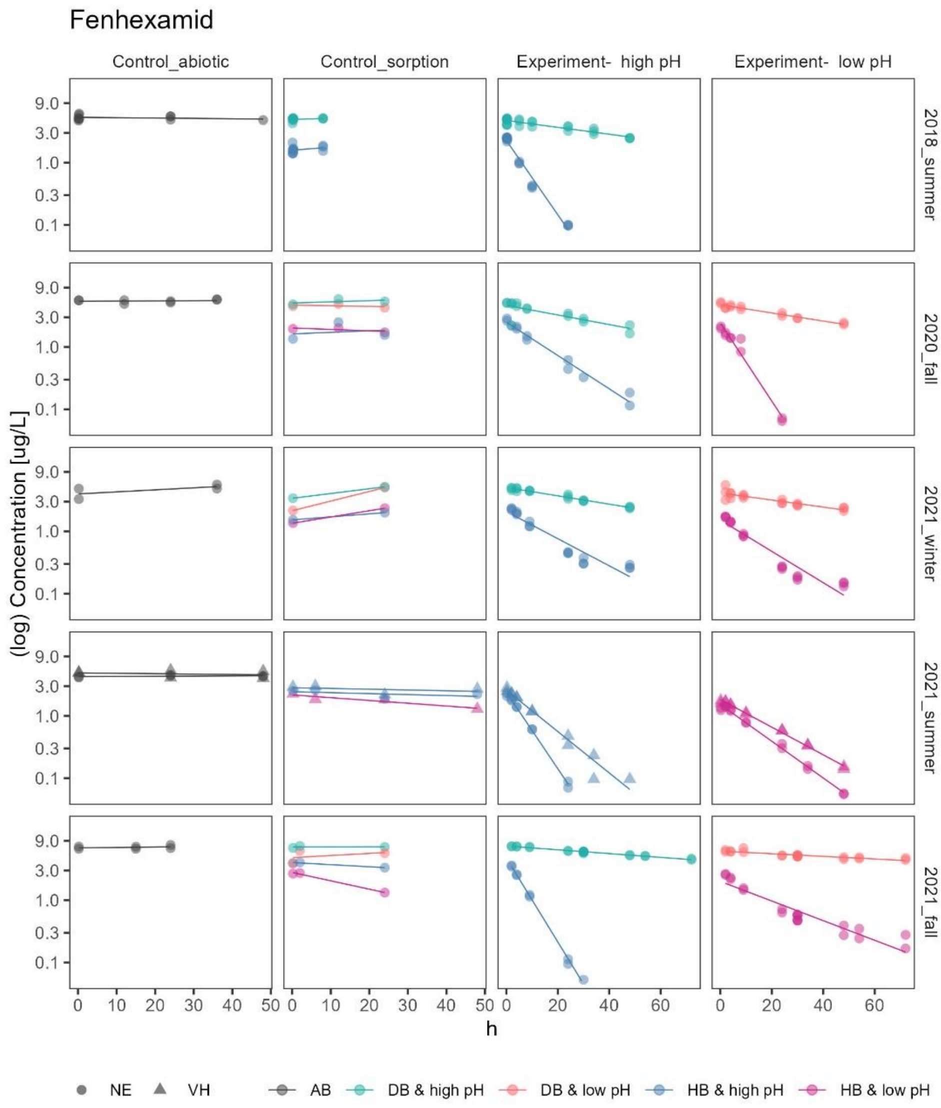

Figure S11. Timeseries of semi-quantified concentrations of fenhexamid in all 5 experiments

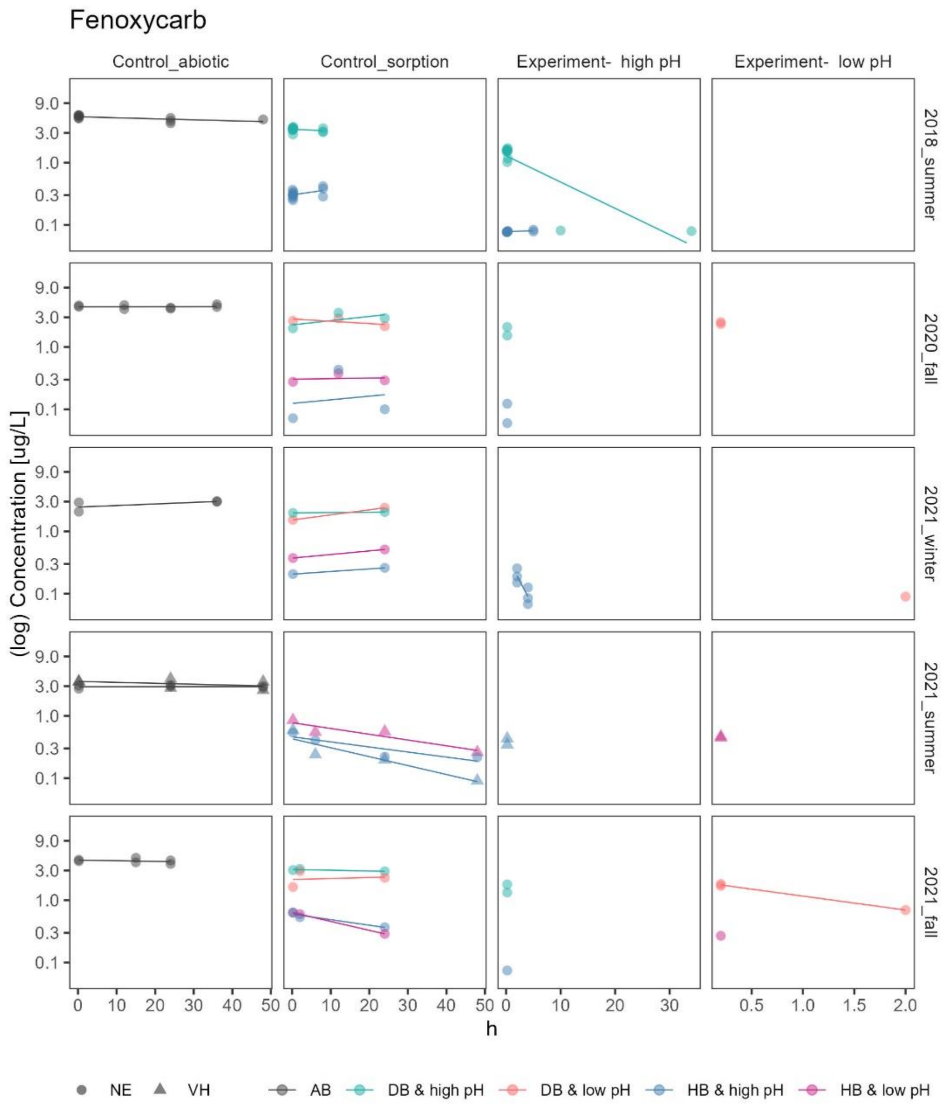

Figure S12. Timeseries of semi-quantified concentrations of fenoxycarb in all 5 experiments

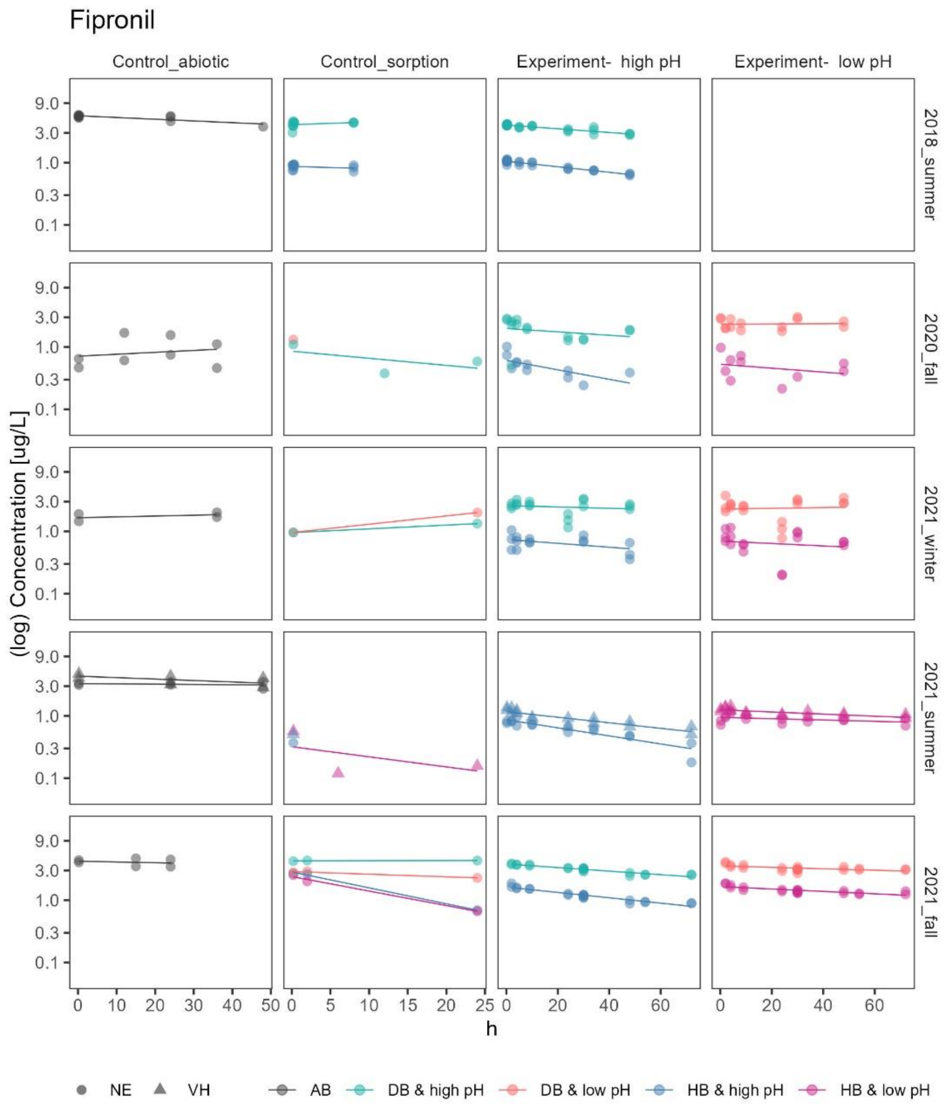

Figure S13. Timeseries of semi-quantified concentrations of fipronil in all 5 experiments

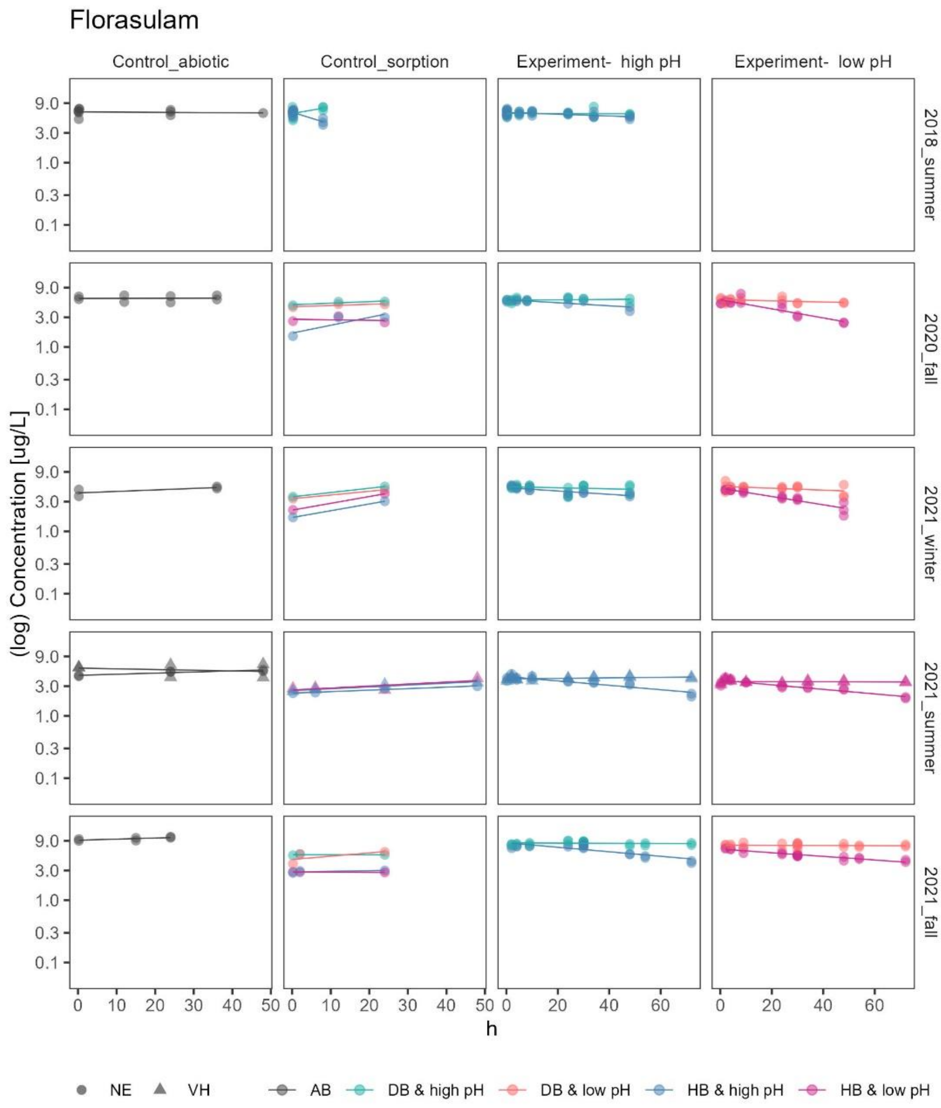

Figure S14. Timeseries of semi-quantified concentrations of florasulam in all 5 experiments

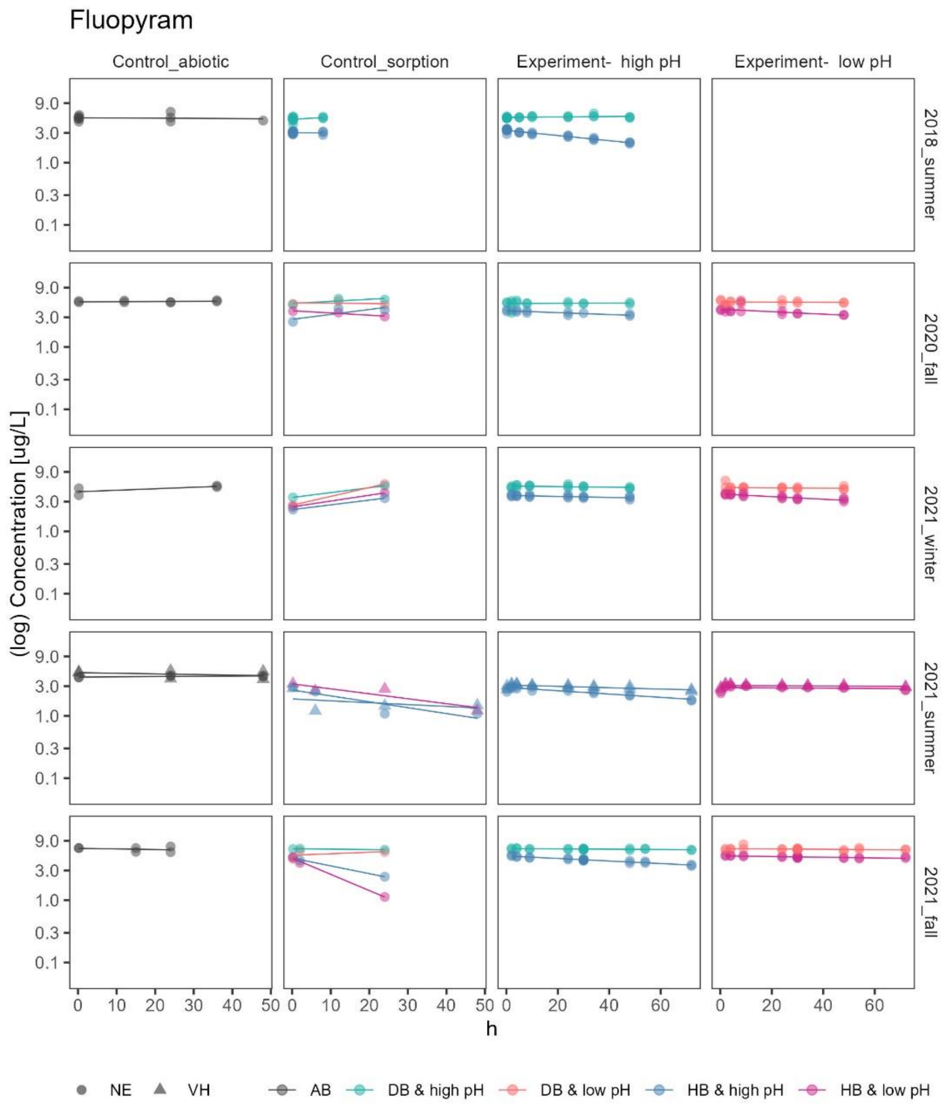

Figure S15. Timeseries of semi-quantified concentrations of fluopyram in all 5 experiments

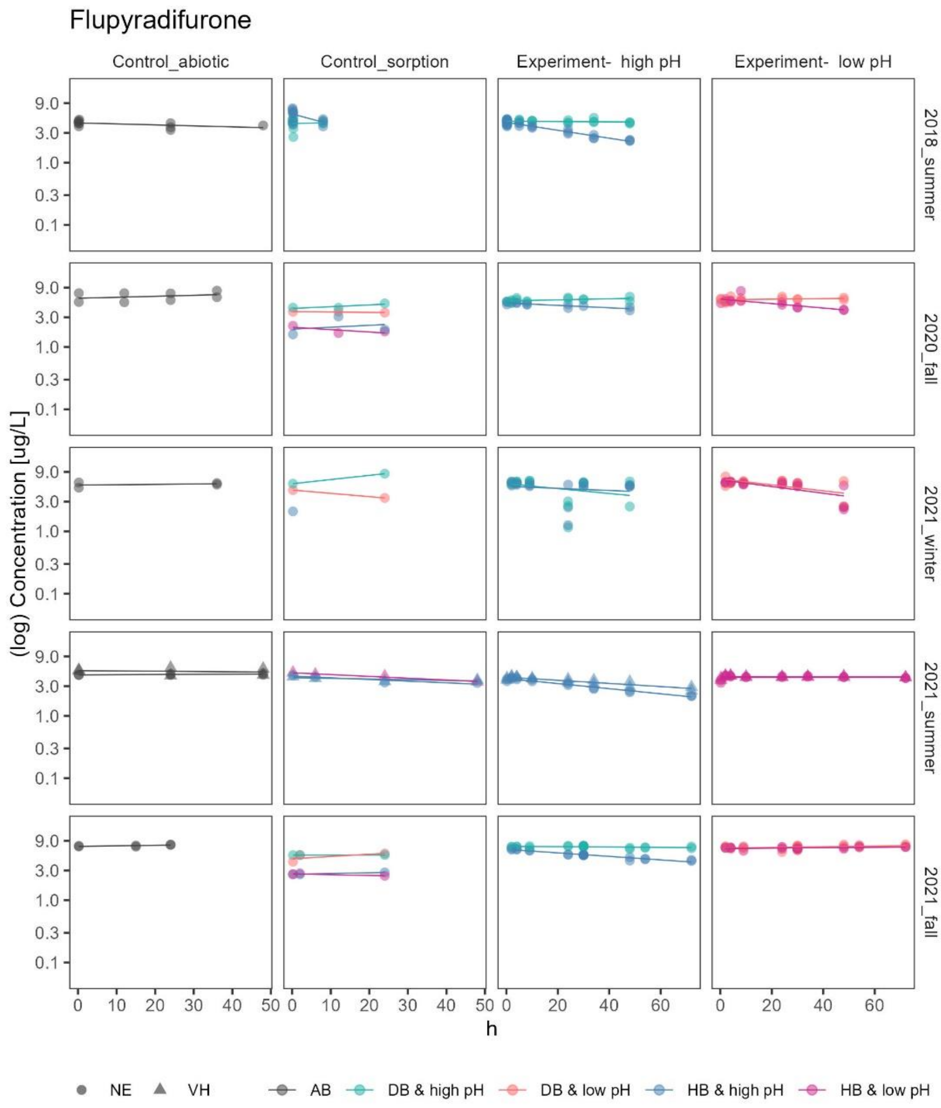

Figure S16. Timeseries of semi-quantified concentrations of flupyradifurone in all 5 experiments

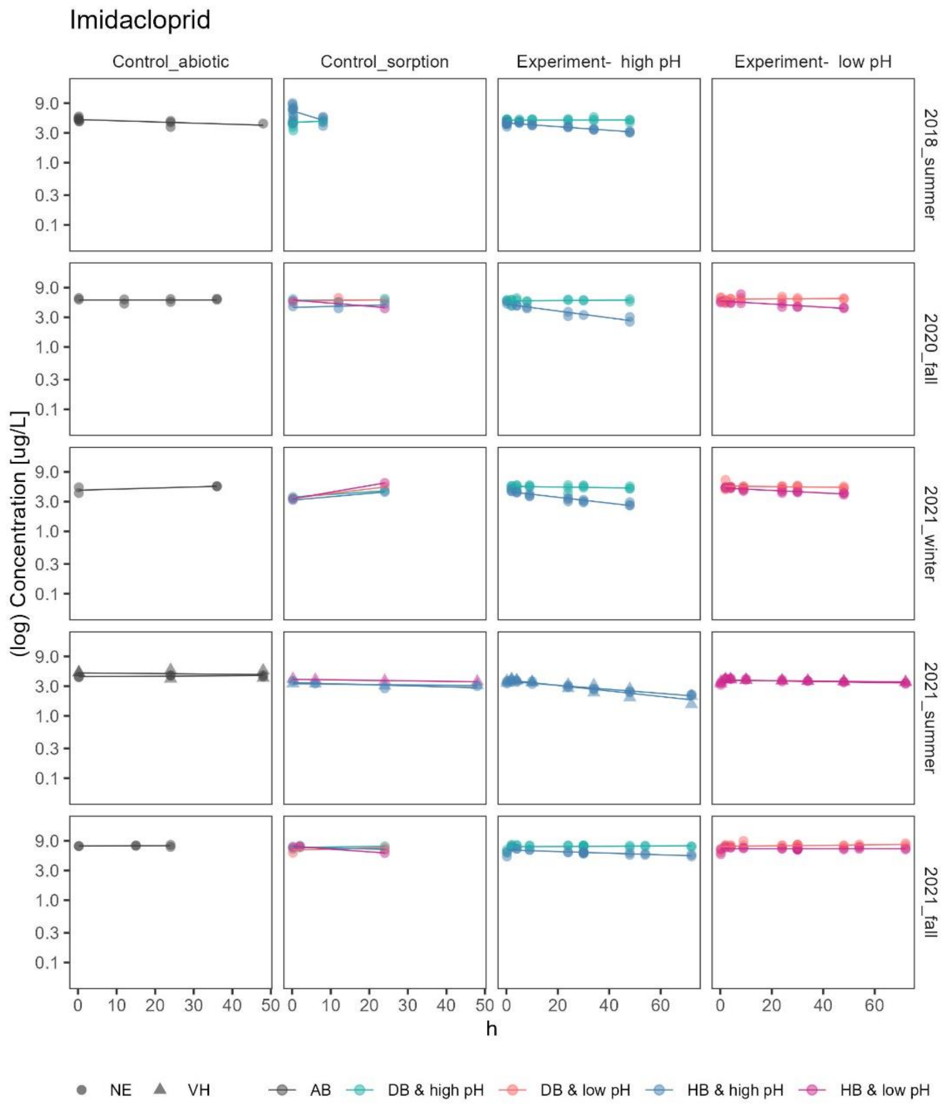

Figure S17. Timeseries of semi-quantified concentrations of imidacloprid in all 5 experiments

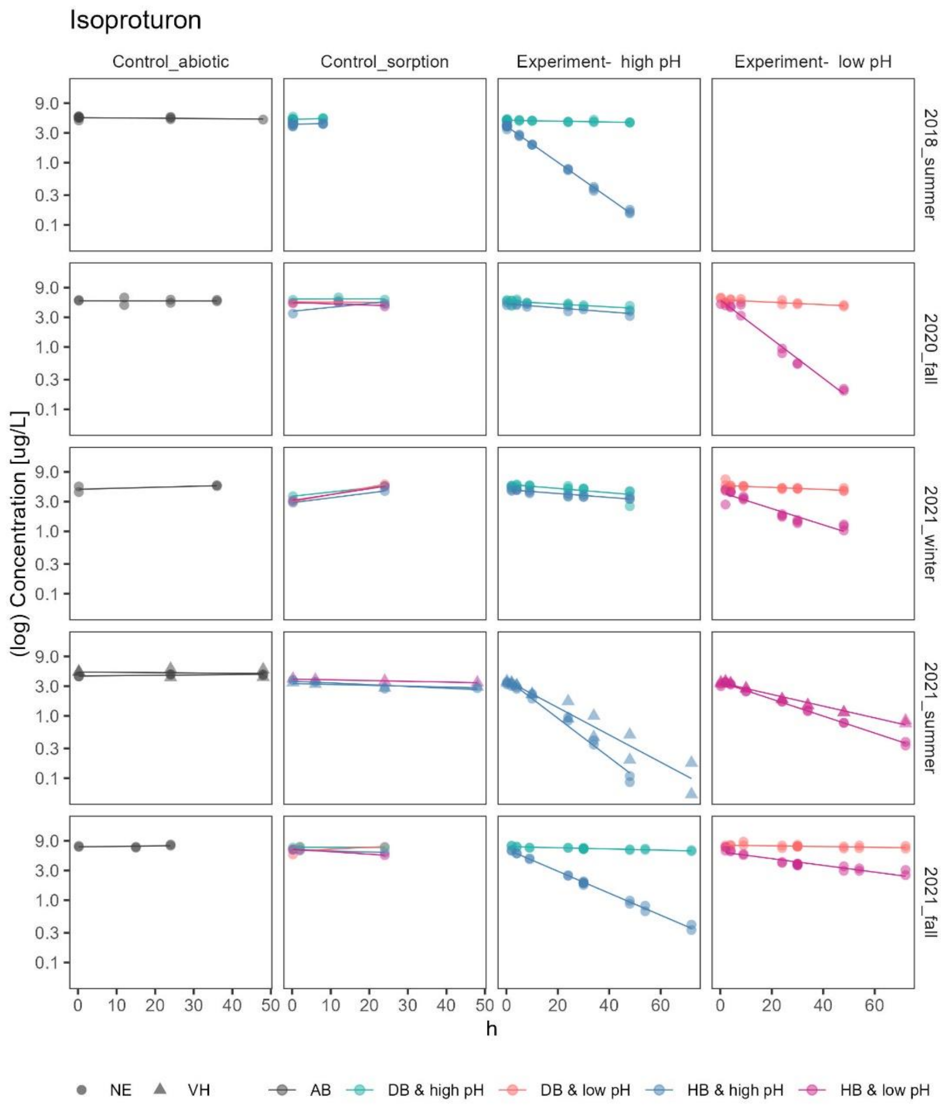

Figure S18. Timeseries of semi-quantified concentrations of isoproturon in all 5 experiments

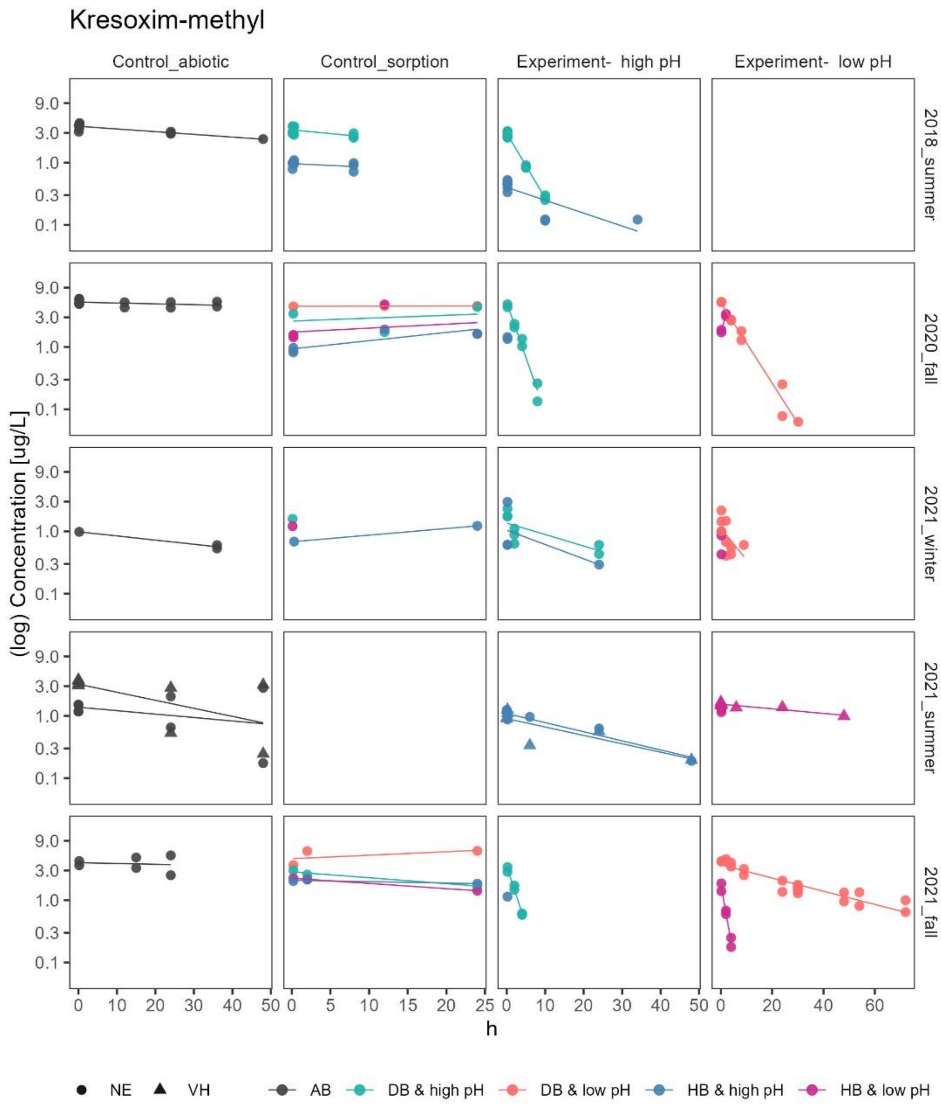

459

460 **Figure S19. Timeseries of semi-quantified concentrations of kresoxim-methyl in all 5 experiments**

461

462

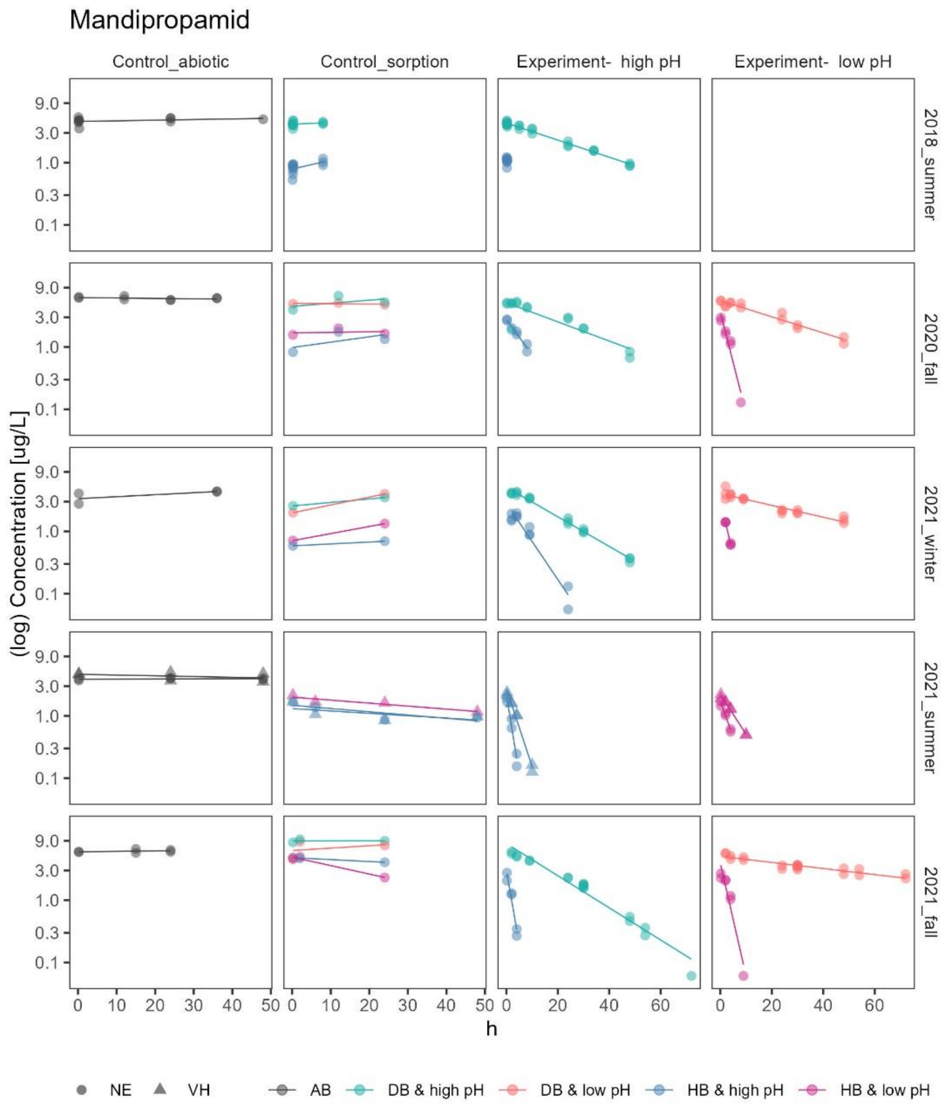

Figure S20. Timeseries of semi-quantified concentrations of mandipropamid in all 5 experiments

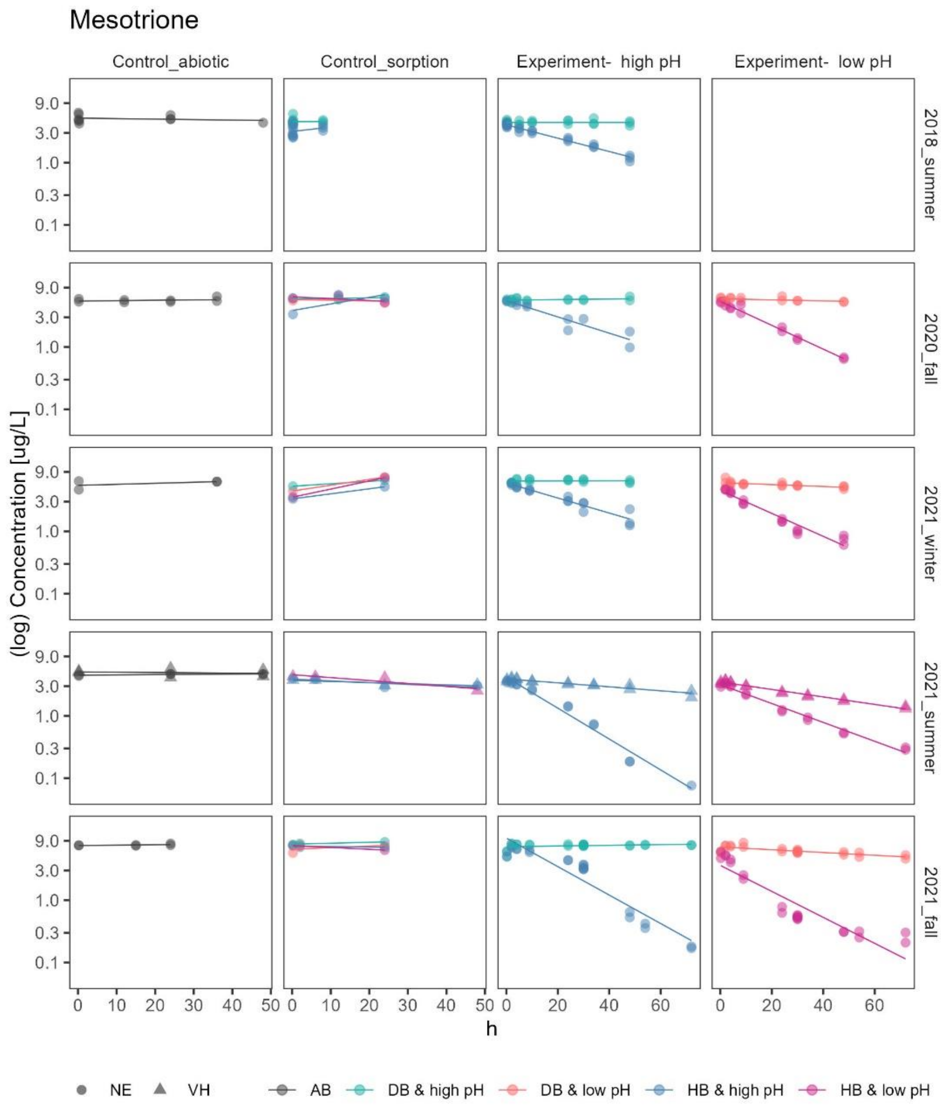

Figure S21. Timeseries of semi-quantified concentrations of mesotrione in all 5 experiments

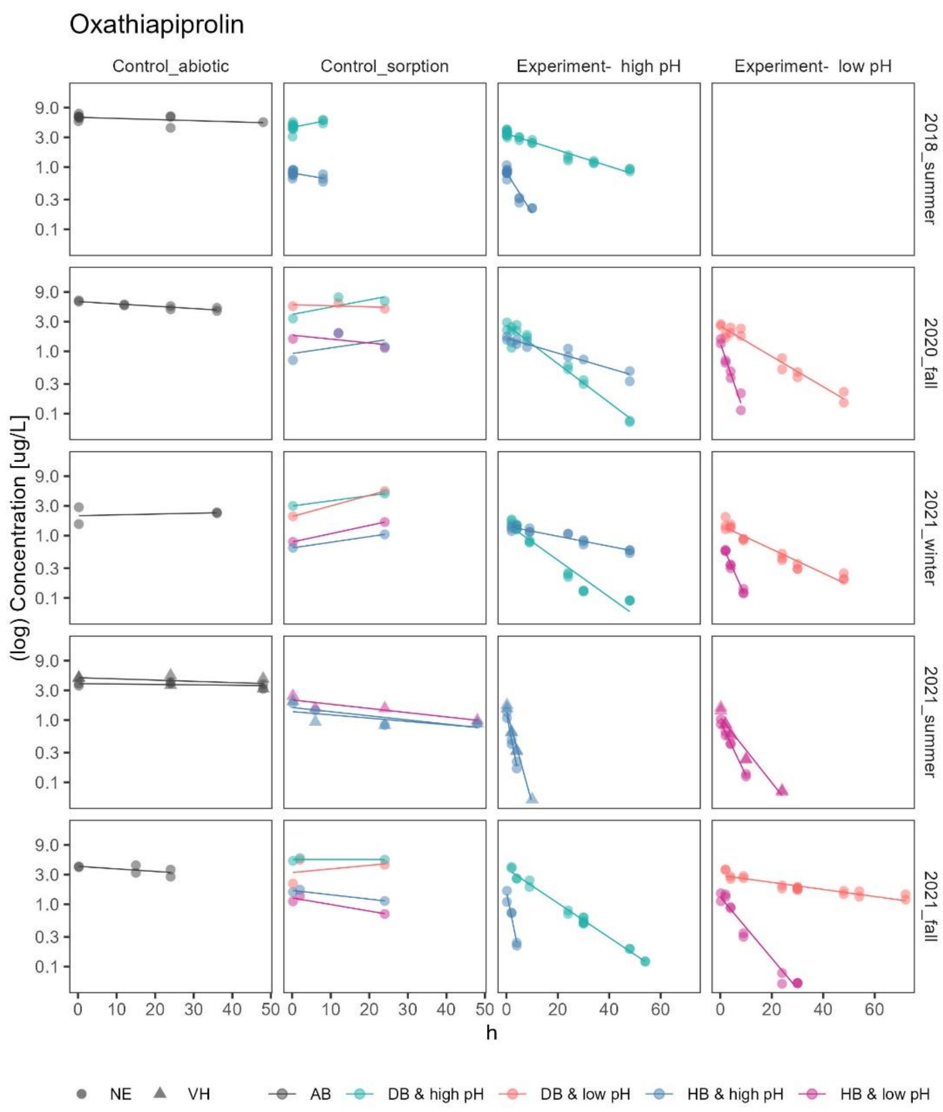

Figure S22. Timeseries of semi-quantified concentrations of oxathiapiprolin in all 5 experiments

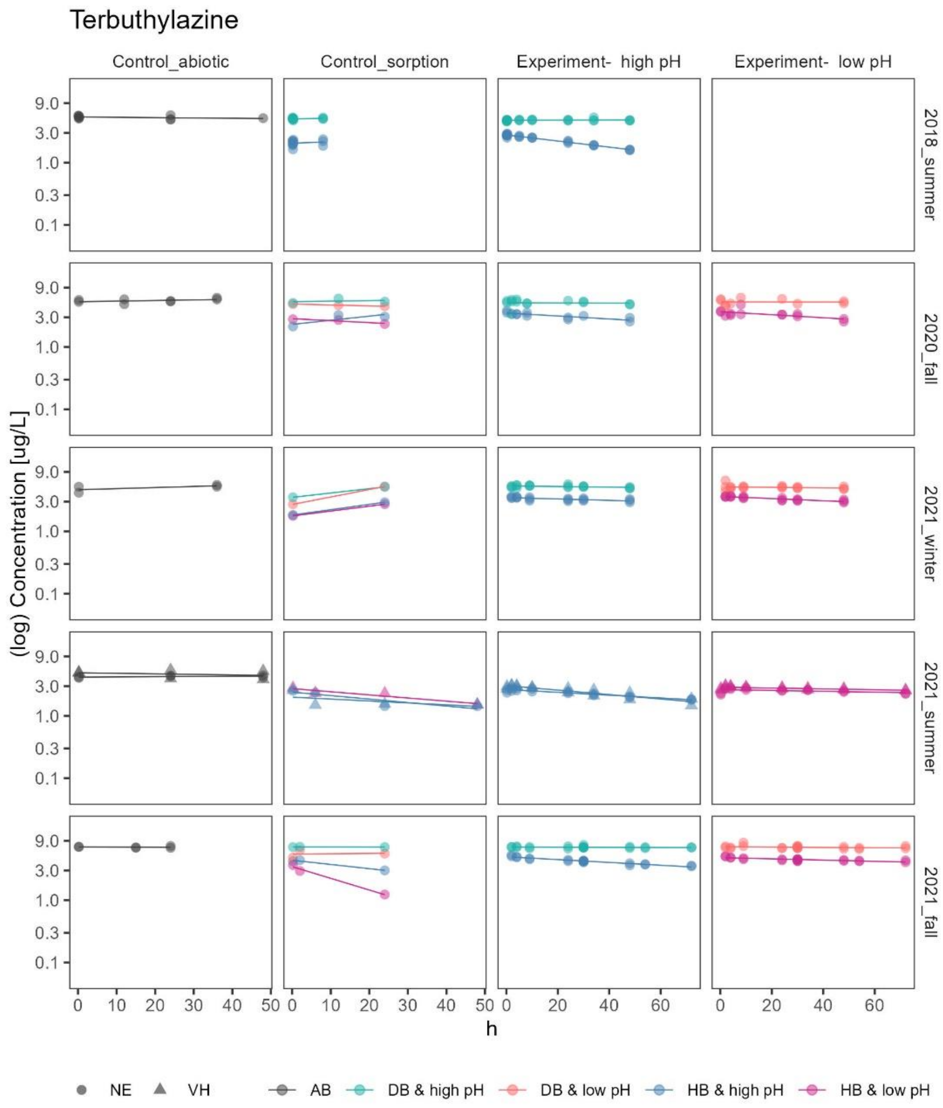

Figure S23. Timeseries of semi-quantified concentrations of terbuthylazine in all 5 experiments

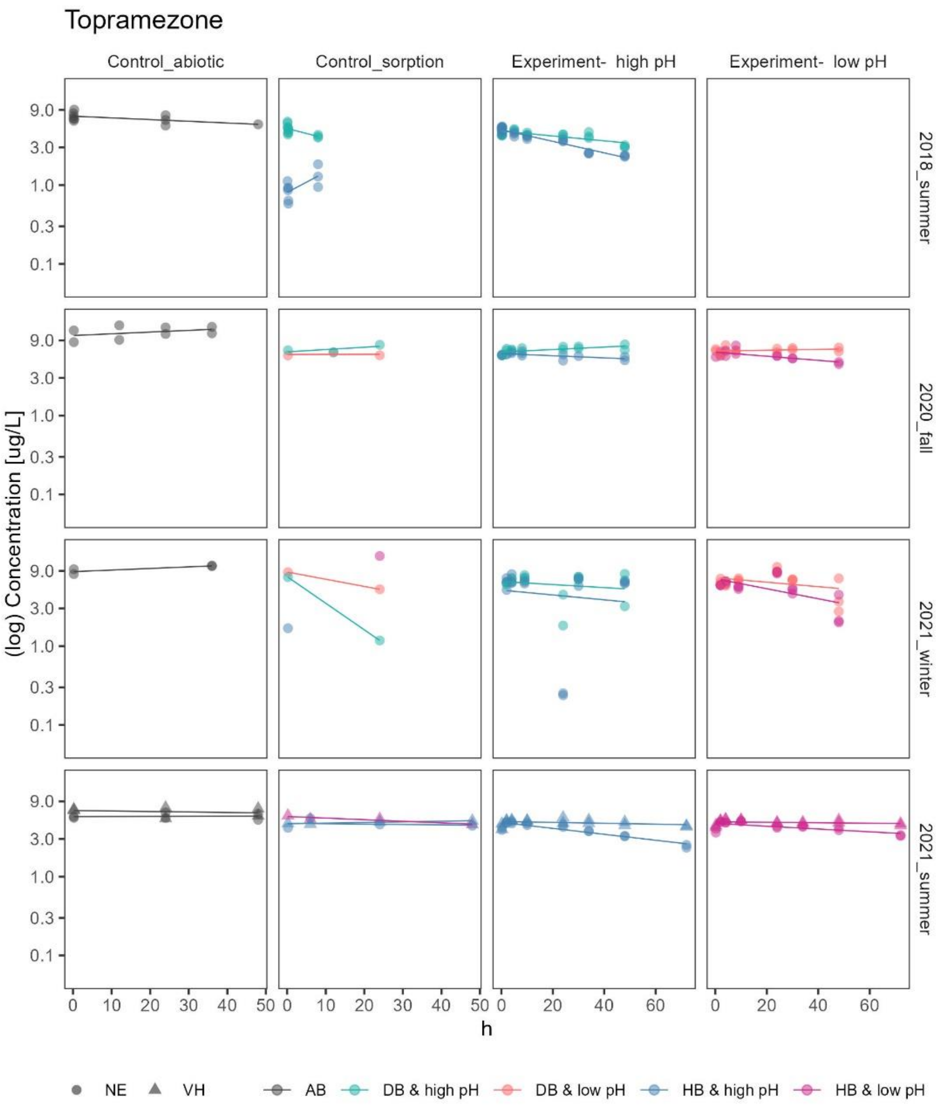

Figure S24. Timeseries of semi-quantified concentrations of topramezone in all 4 experiments where it was detected

S4.2 Plant protection products (PPPs)

The concentration time series of the 26 PPPs in the test set can be seen in the supporting information of the previous publication[2]: https://pubs.acs.org/doi/10.1021/acs.est.9b05104.

S4.3 Active pharmaceutical ingredients (APIs)

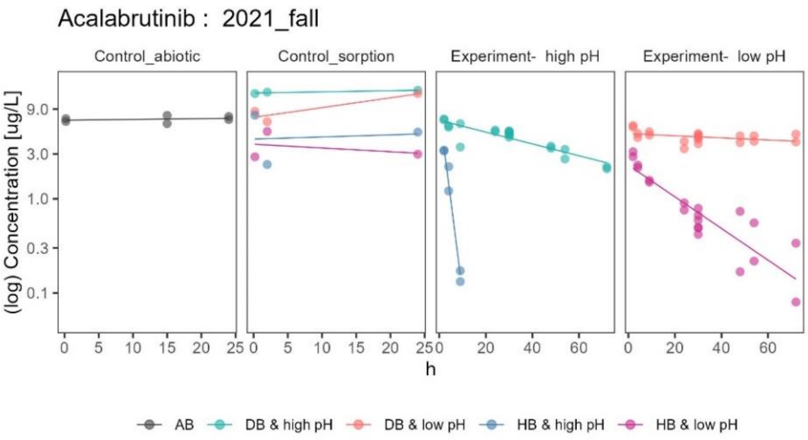

Figure S25. Timeseries of semi-quantified concentrations of acalabrutinib in experiment 2021-fall

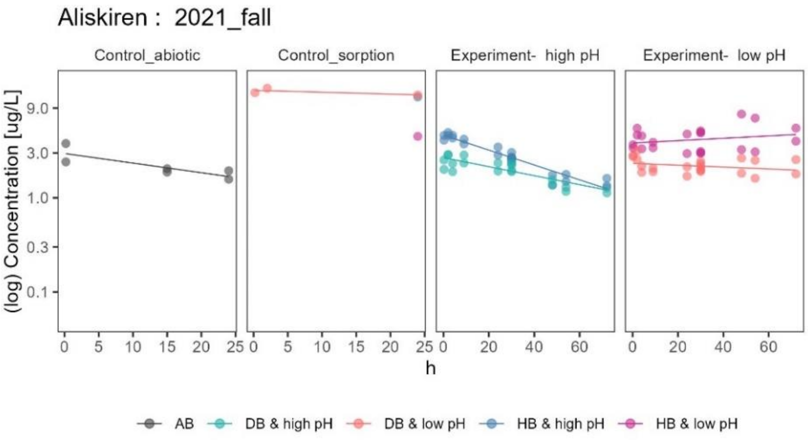

Figure S26. Timeseries of semi-quantified concentrations of aliskiren in experiment 2021-fall

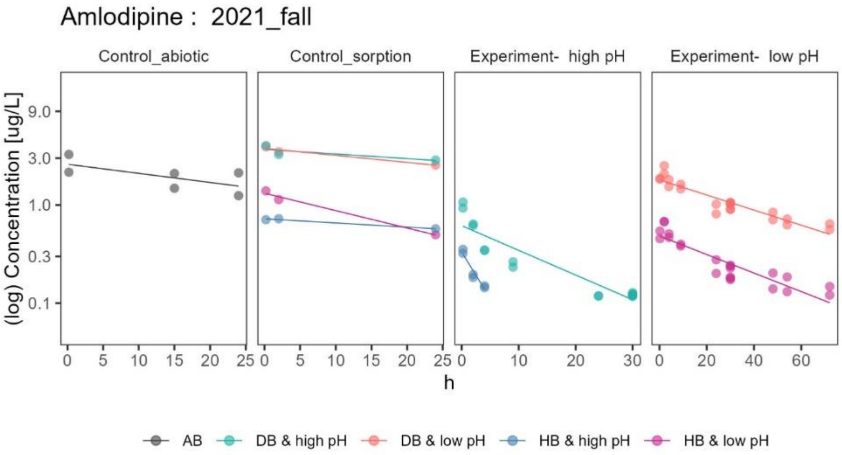

Figure S27. Timeseries of semi-quantified concentrations of amlodipine in experiment 2021-fall

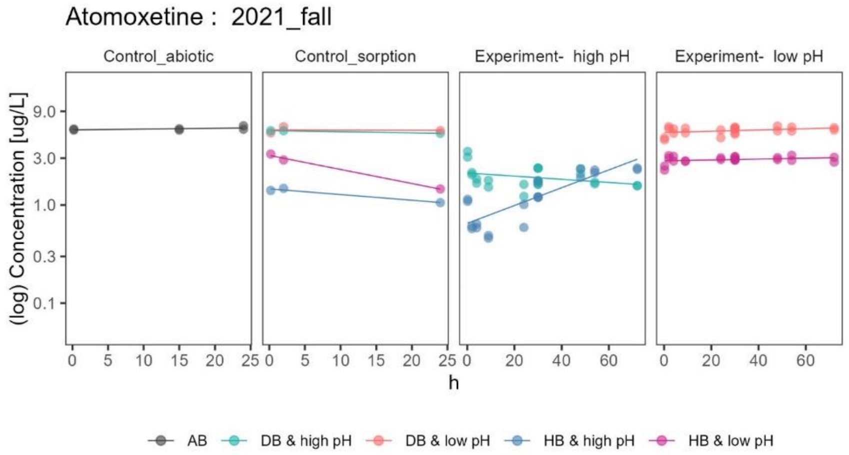

Figure S28. Timeseries of semi-quantified concentrations of atomoxetine in experiment 2021-fall

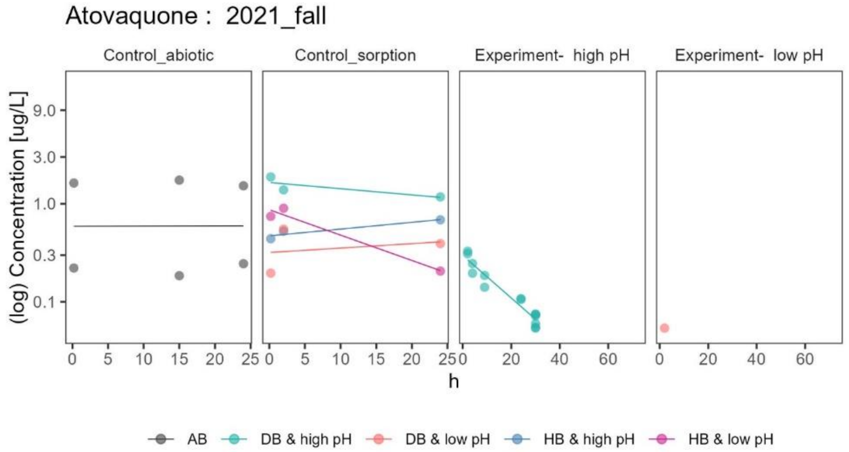

Figure S29, Timeseries of semi-quantified concentrations of atovaquone in experiment 2021-fall

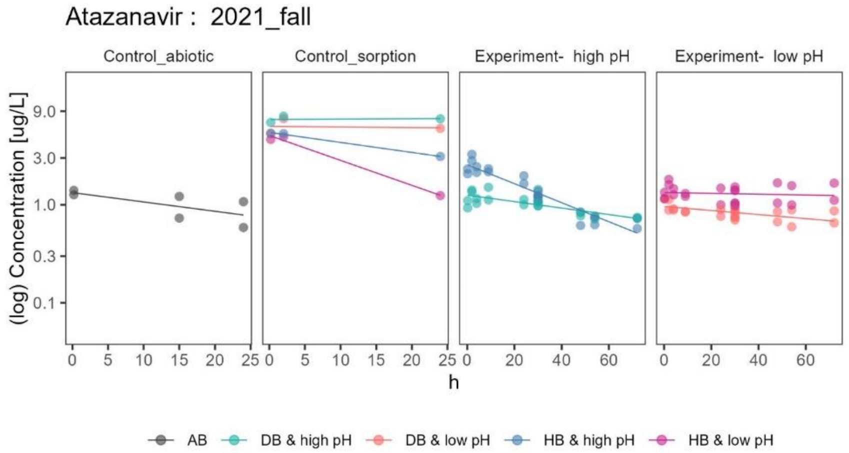

Figure S30. Timeseries of semi-quantified concentrations of atazanavir in experiment 2021-fall

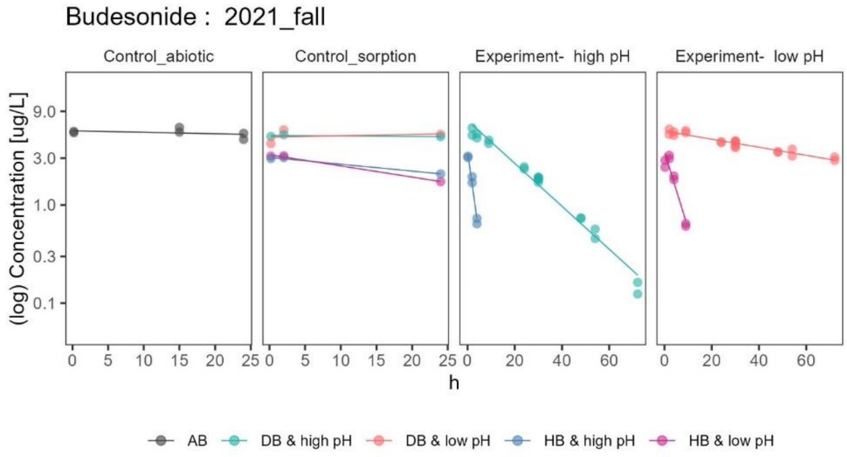

Figure S31. Timeseries of semi-quantified concentrations of budesonide in experiment 2021-fall

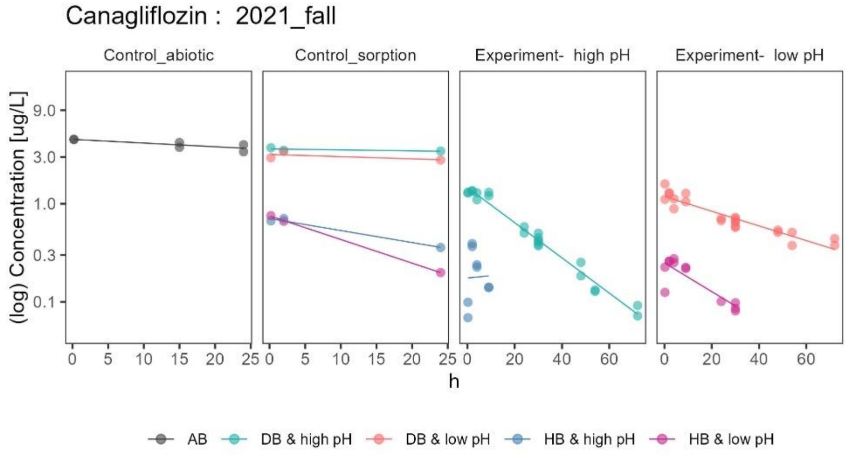

Figure S32. Timeseries of semi-quantified concentrations of canagliflozin in experiment 2021-fall

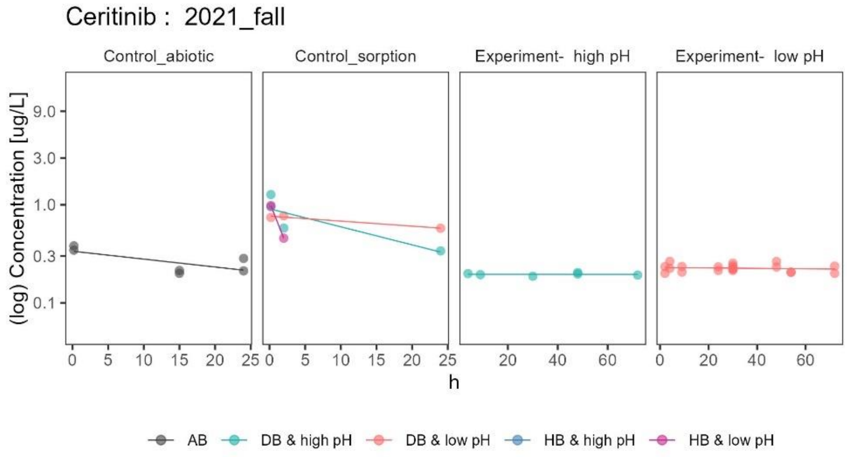

Figure S33. Timeseries of semi-quantified concentrations of ceritinib in experiment 2021-fall

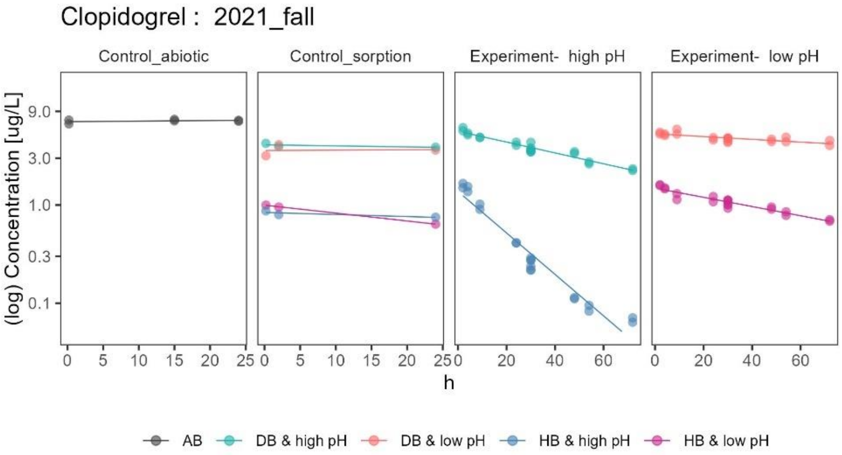

Figure S34. Timeseries of semi-quantified concentrations of clopidogrel in experiment 2021-fall

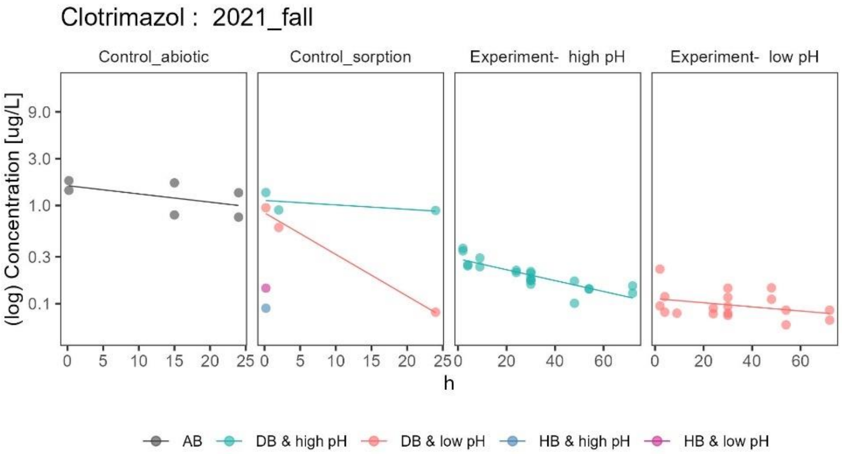

Figure S35. Timeseries of semi-quantified concentrations of clotrimazol in experiment 2021-fall

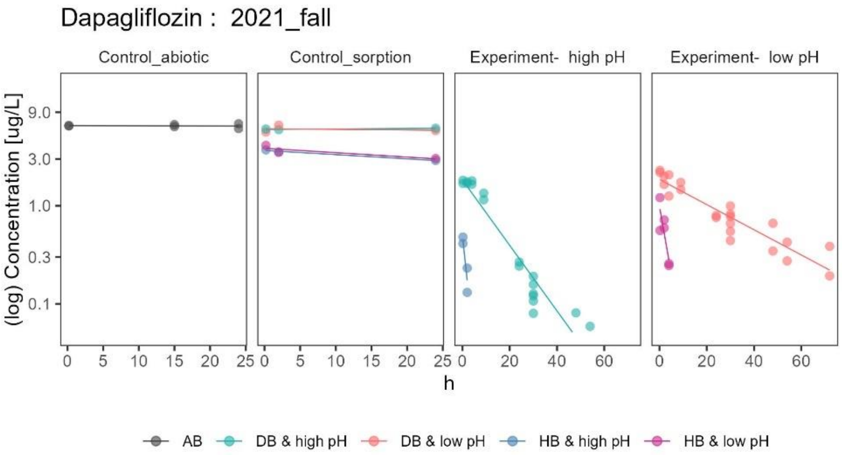

Figure S36. Timeseries of semi-quantified concentrations of dapagliflozin in experiment 2021-fall

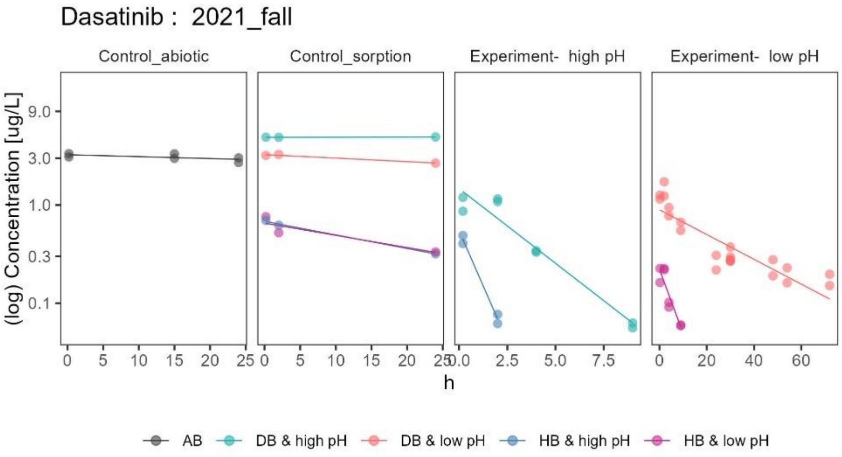

Figure S37. Timeseries of semi-quantified concentrations of dasatinib in experiment 2021-fall

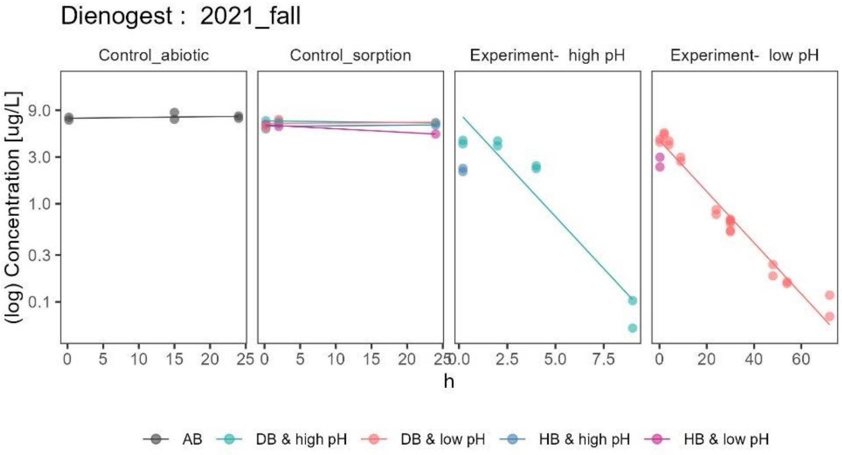

Figure S38. Timeseries of semi-quantified concentrations of dienogest in experiment 2021-fall

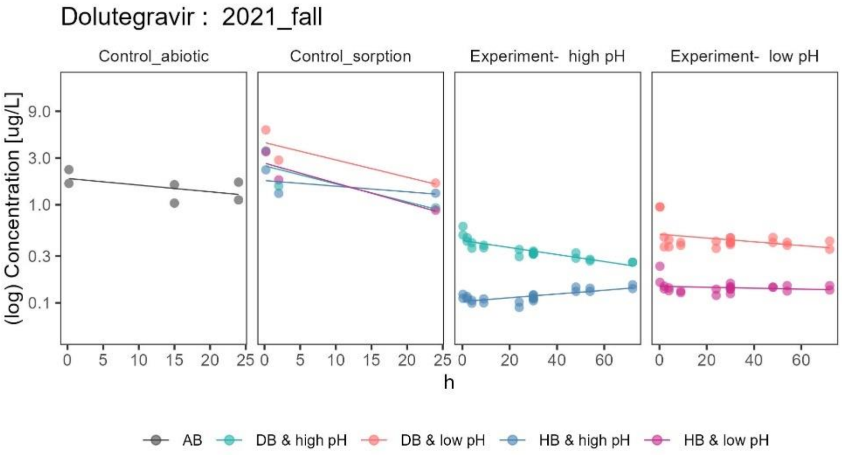

Figure S39. Timeseries of semi-quantified concentrations of dolutegravir in experiment 2021-fall

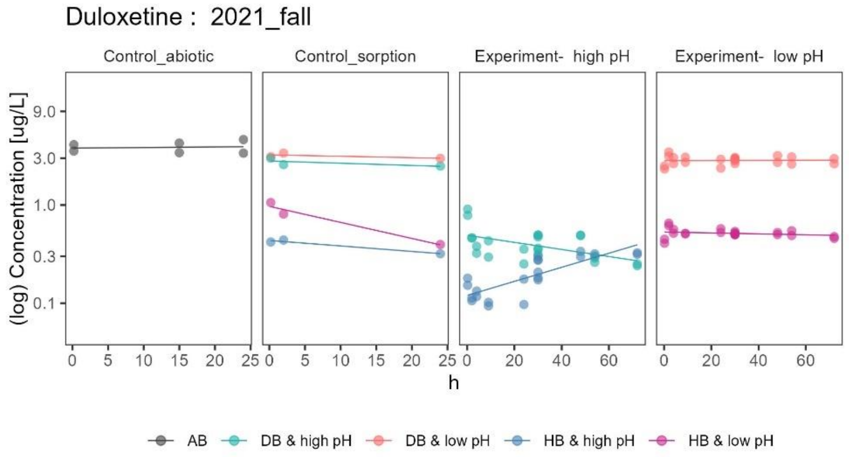

Figure S40. Timeseries of semi-quantified concentrations of duloxetine in experiment 2021-fall

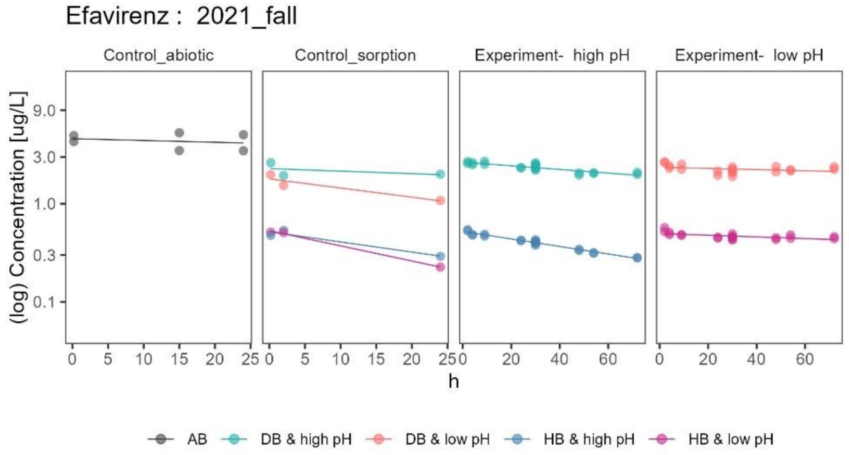

Figure S41. Timeseries of semi-quantified concentrations of efavirenz in experiment 2021-fall

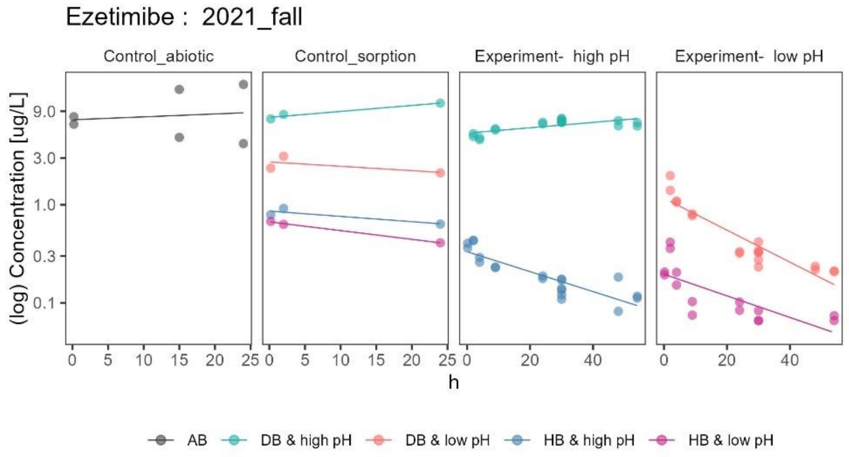

Figure S42. Timeseries of semi-quantified concentrations of ezetimbe in experiment 2021-fall

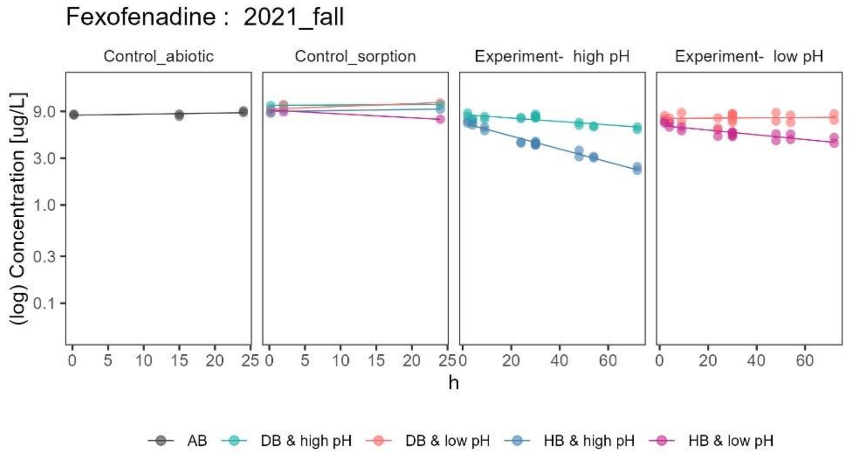

Figure S43. Timeseries of semi-quantified concentrations of fexofenadine in experiment 2021-fall

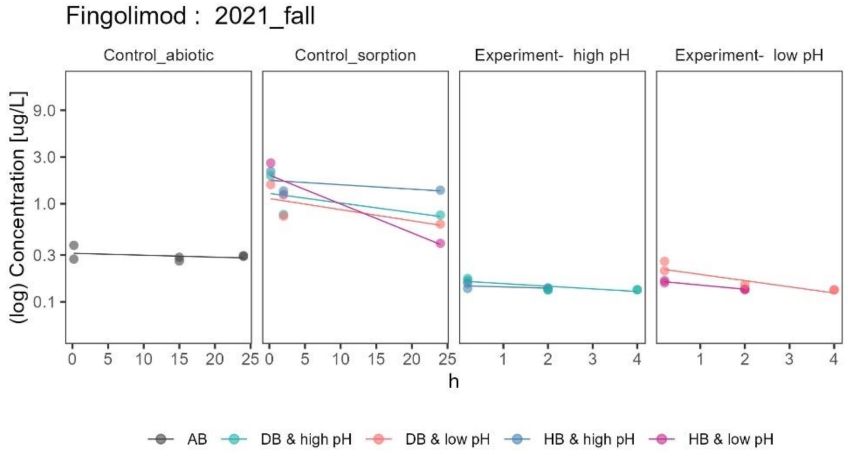

Figure S44. Timeseries of semi-quantified concentrations of fingolimod in experiment 2021-fall

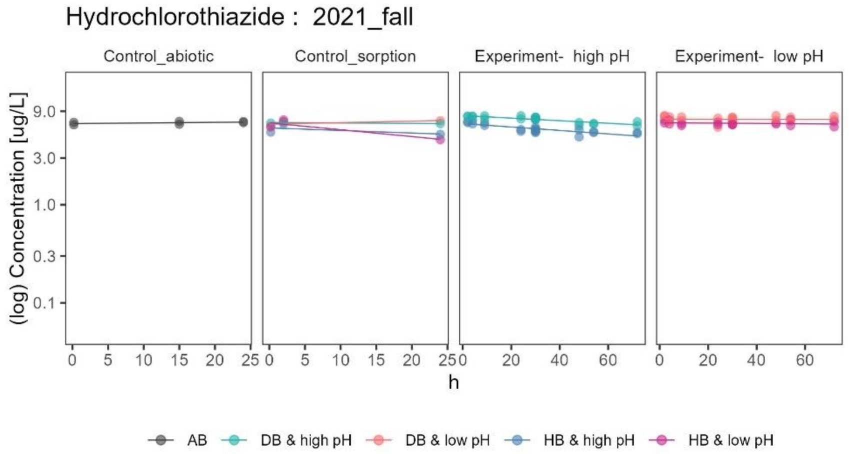

Figure S45. Timeseries of semi-quantified concentrations of hydrochlorothiazide in experiment 2021-fall

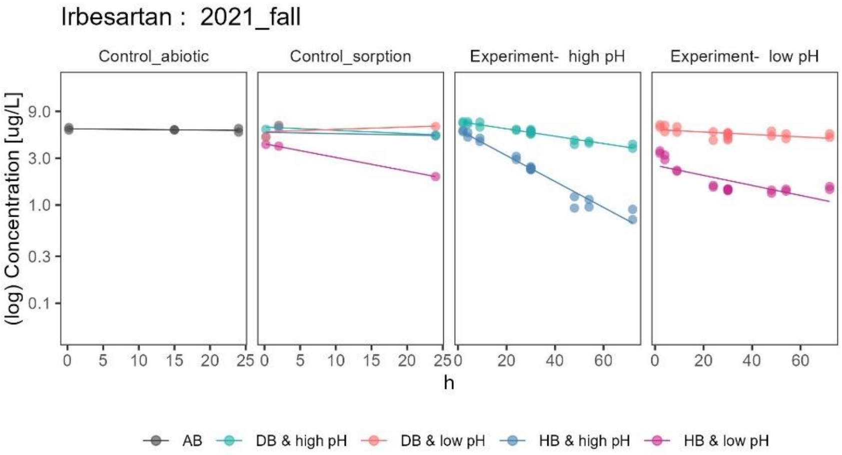

Figure S46. Timeseries of semi-quantified concentrations of irbesartan in experiment 2021-fall

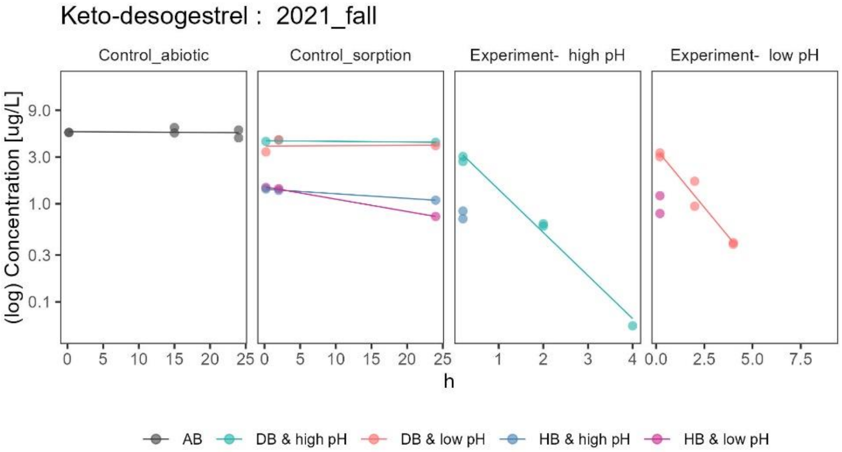

Figure S47. Timeseries of semi-quantified concentrations of keto-desogestrel in experiment 2021-fall

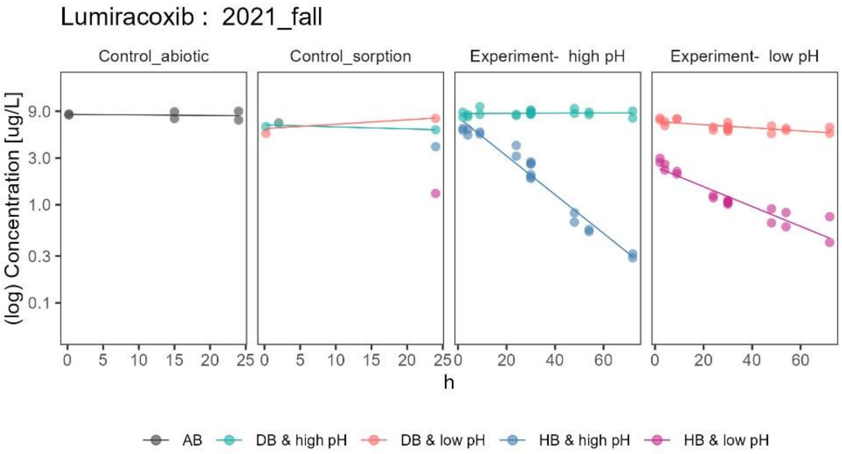

Figure S48. Timeseries of semi-quantified concentrations of lumiracoxib in experiment 2021-fall

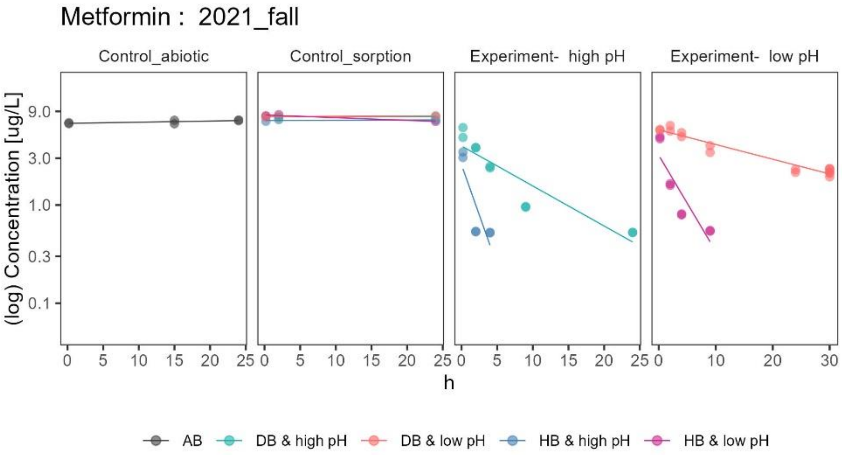

Figure S49. Timeseries of semi-quantified concentrations of metformin in experiment 2021-fall

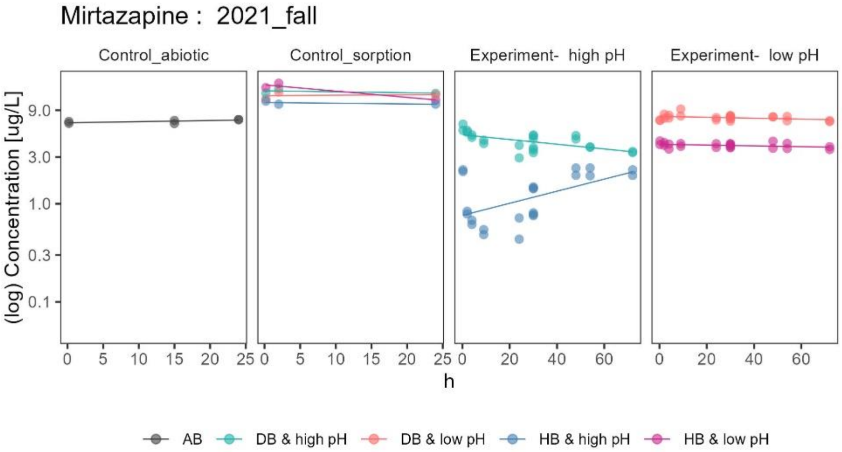

Figure S50. Timeseries of semi-quantified concentrations of mirtazepine in experiment 2021-fall

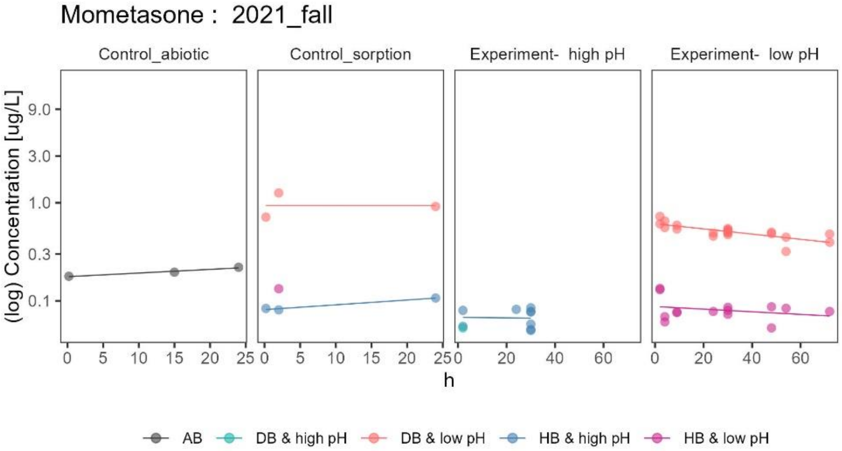

Figure S51. Timeseries of semi-quantified concentrations of mometasone in experiment 2021-fall

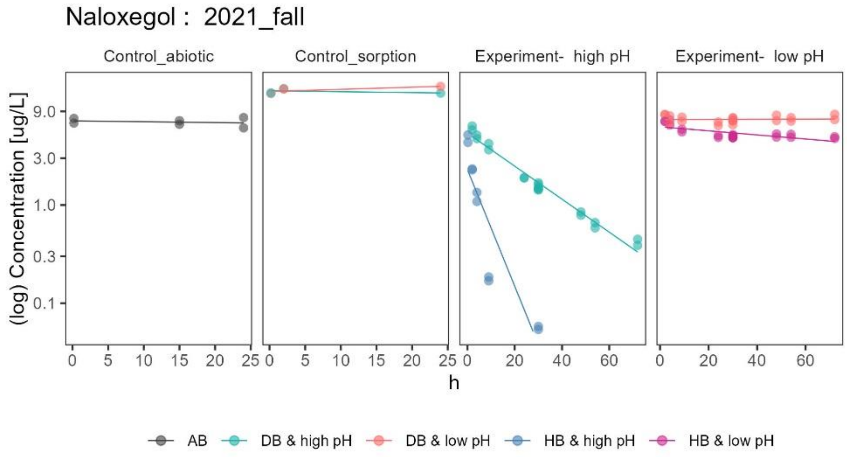

Figure S52. Timeseries of semi-quantified concentrations of naloxegol in experiment 2021-fall

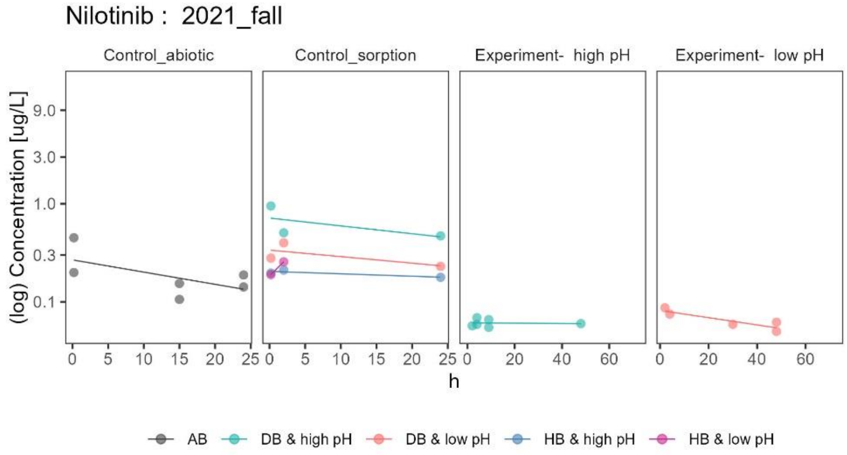

Figure S53. Timeseries of semi-quantified concentrations of nilotinib in experiment 2021-fall

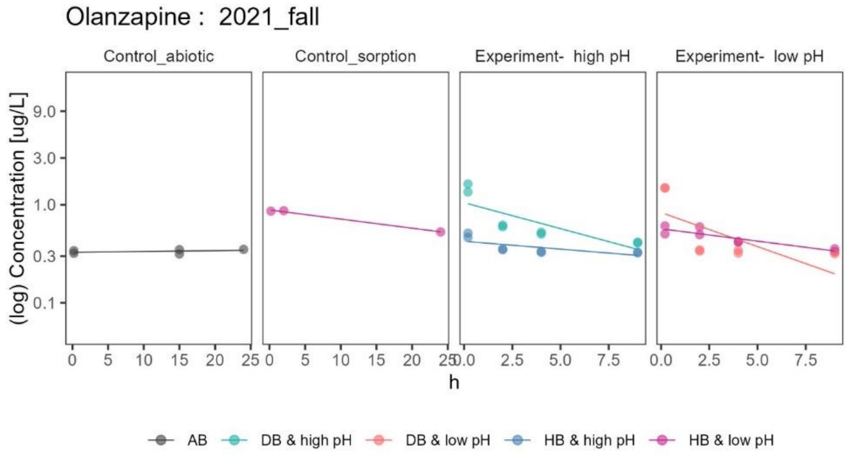

Figure S54 Timeseries of semi-quantified concentrations of olanzepine in experiment 2021-fall

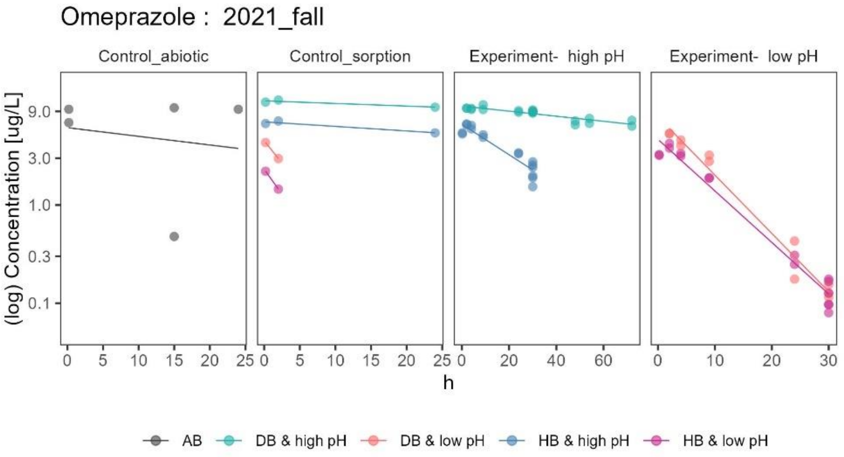

Figure S55. Timeseries of semi-quantified concentrations of omeprazole in experiment 2021-fall

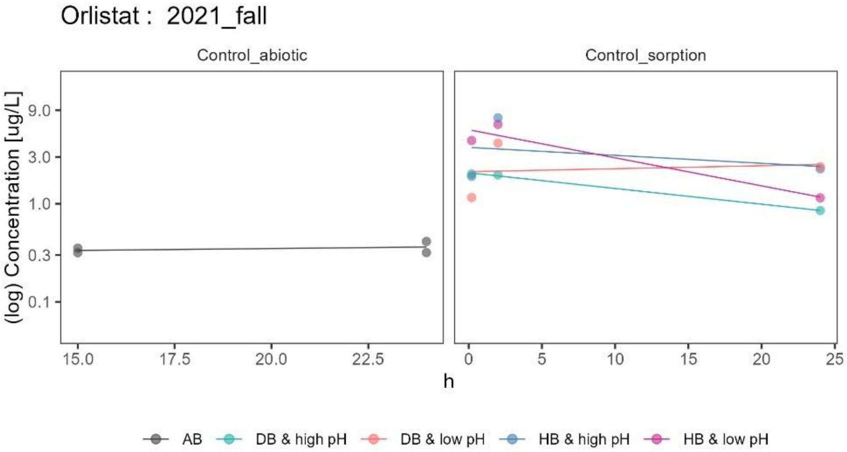

Figure S56. Timeseries of semi-quantified concentrations of orlistat in experiment 2021-fall

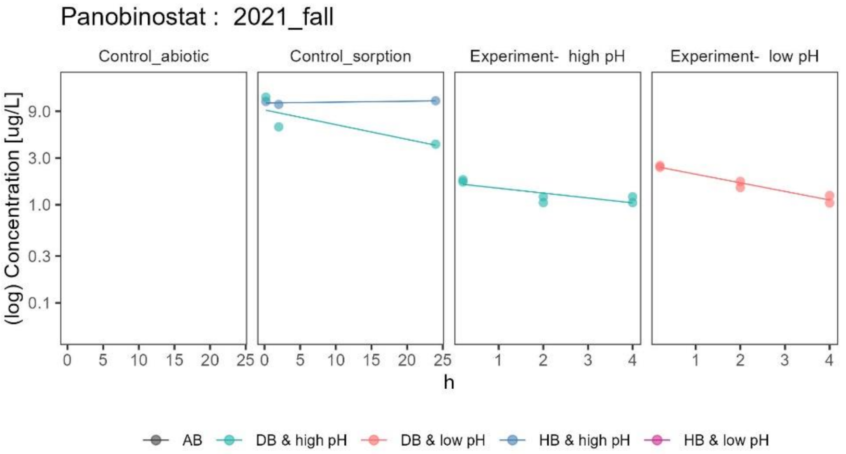

Figure S57. Timeseries of semi-quantified concentrations of panobinostat in experiment 2021-fall

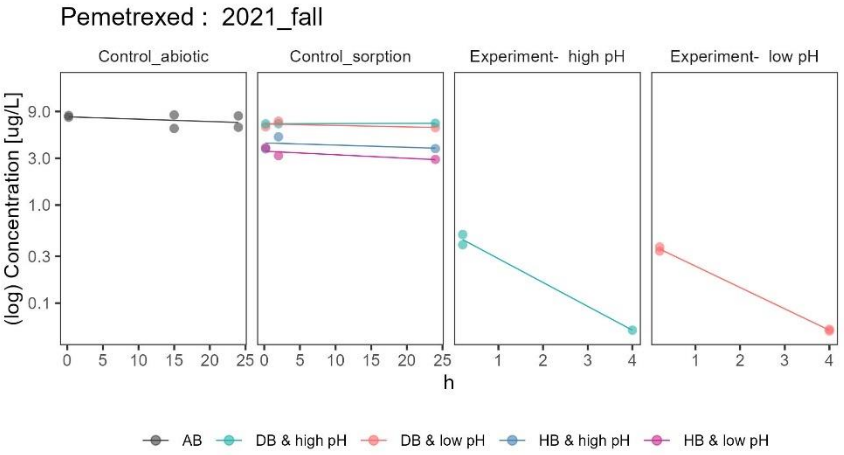

Figure S58. Timeseries of semi-quantified concentrations of pemetrexed in experiment 2021-fall

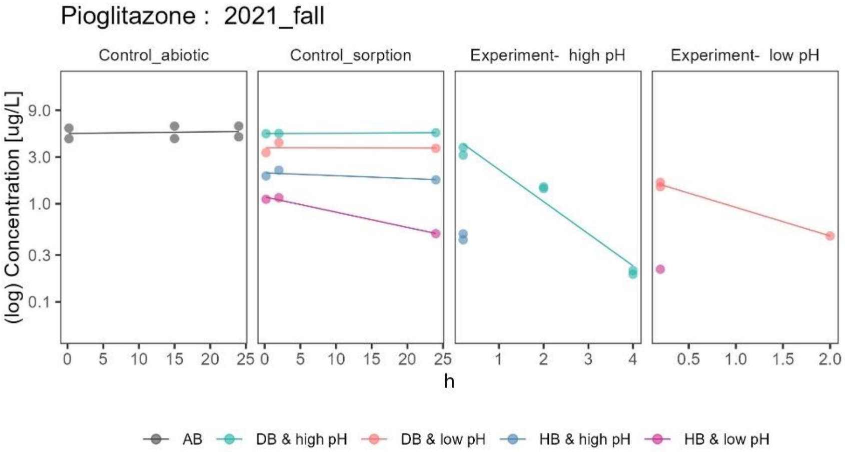

Figure S59. Timeseries of semi-quantified concentrations of pioglitazone in experiment 2021-fall

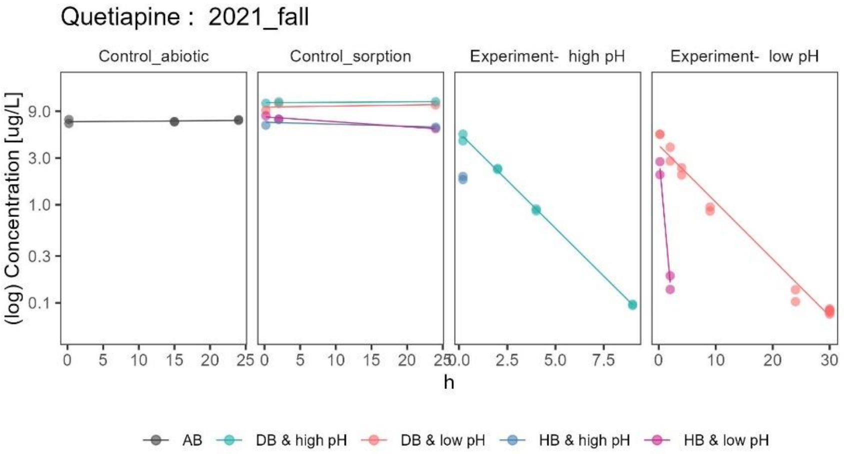

Figure S60. Timeseries of semi-quantified concentrations of quetiapine in experiment 2021-fall

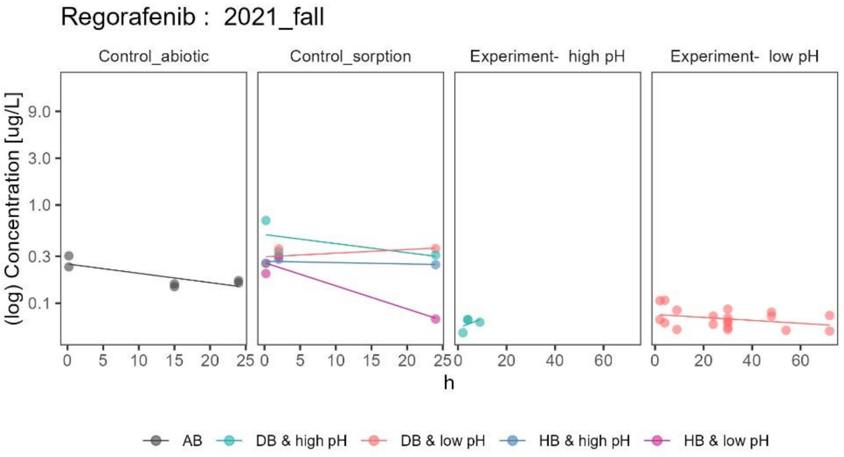

Figure S61. Timeseries of semi-quantified concentrations of regorafenib in experiment 2021-fall

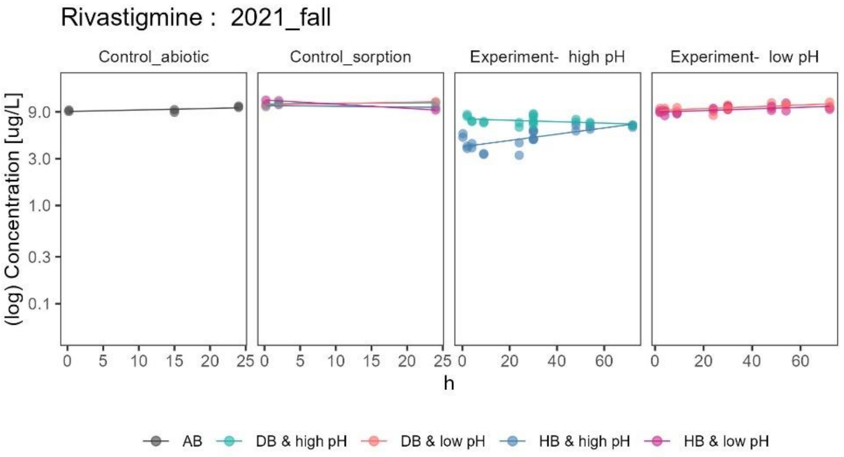

Figure S62. Timeseries of semi-quantified concentrations of rivastigmine in experiment 2021-fall

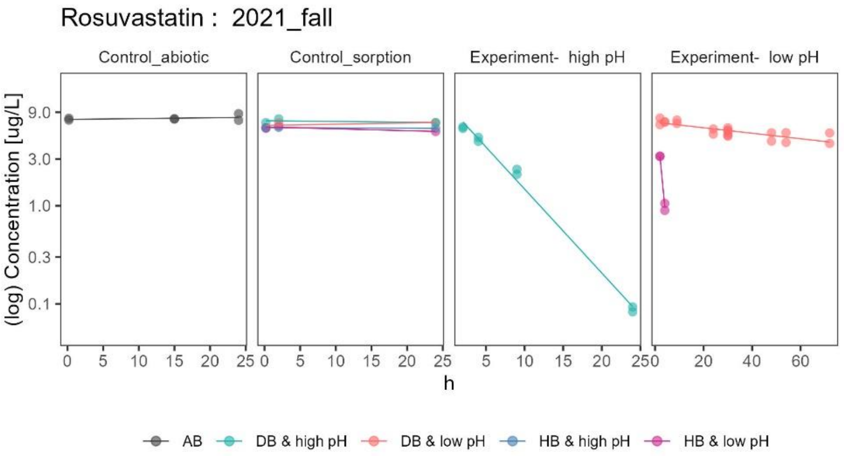

Figure S63. Timeseries of semi-quantified concentrations of rosuvastatin in experiment 2021-fall

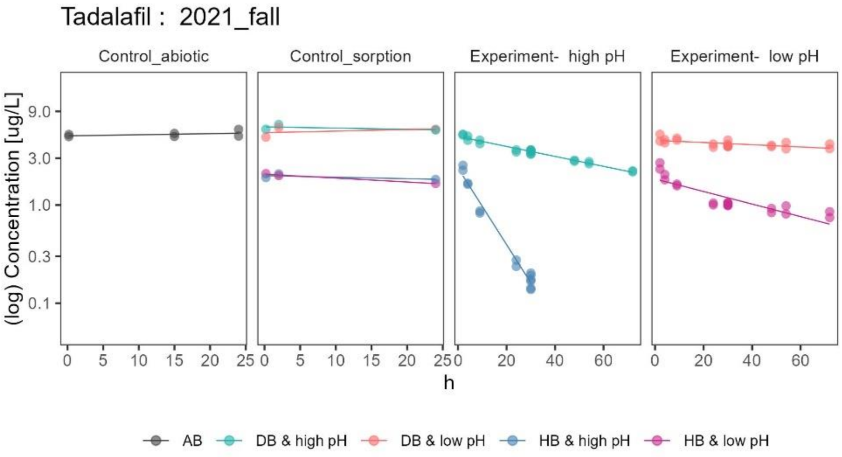

Figure S64. Timeseries of semi-quantified concentrations of tadalafil in experiment 2021-fall

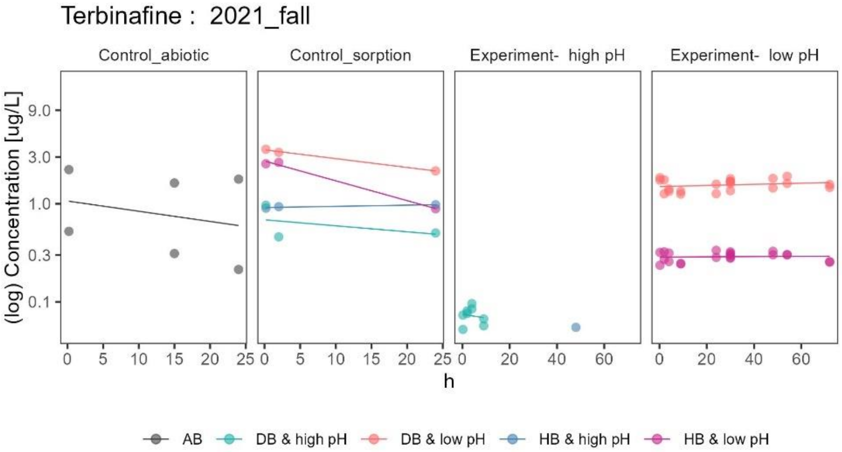

Figure S65. Timeseries of semi-quantified concentrations of terbinafine in experiment 2021-fall

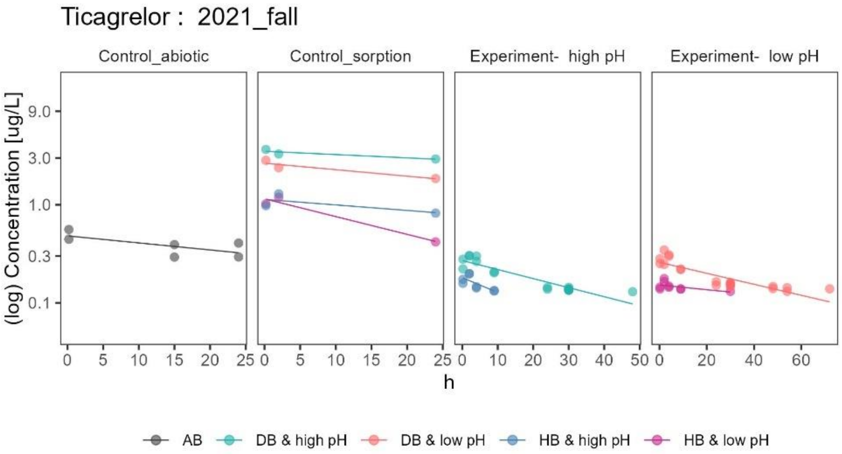

Figure S66. Timeseries of semi-quantified concentrations of ticagrelor in experiment 2021-fall

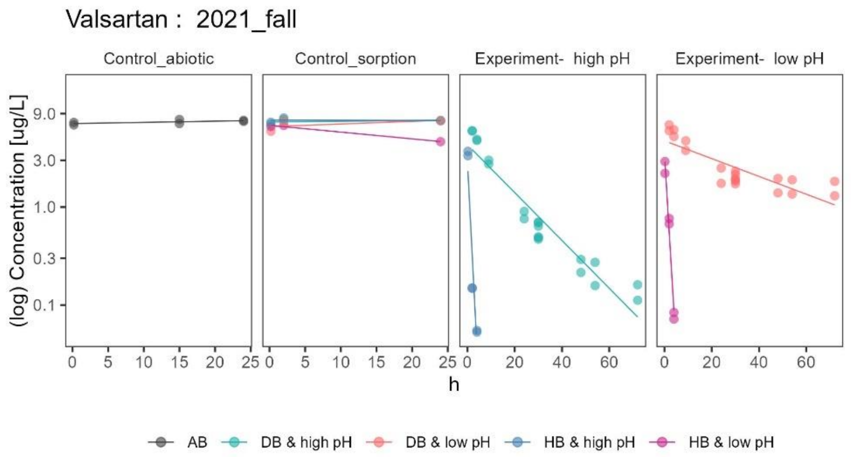

Figure S67. Timeseries of semi-quantified concentrations of valsartan in experiment 2021-fall

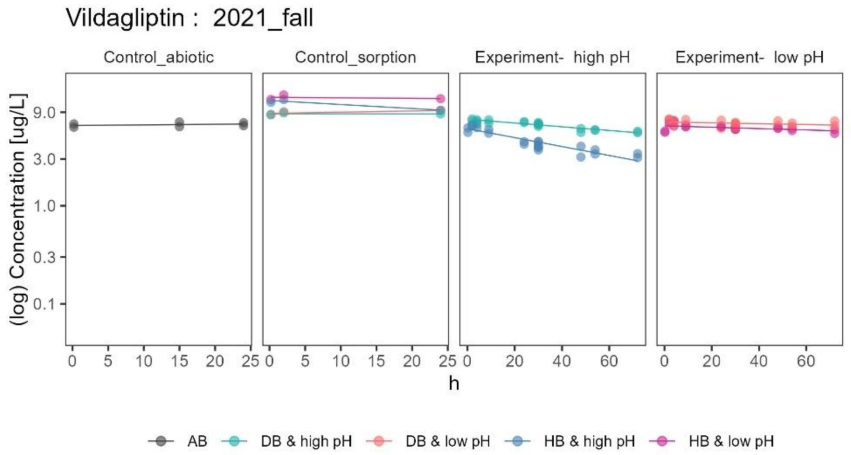

Figure S68. Timeseries of semi-quantified concentrations of vildagliptin in experiment 2021-fall

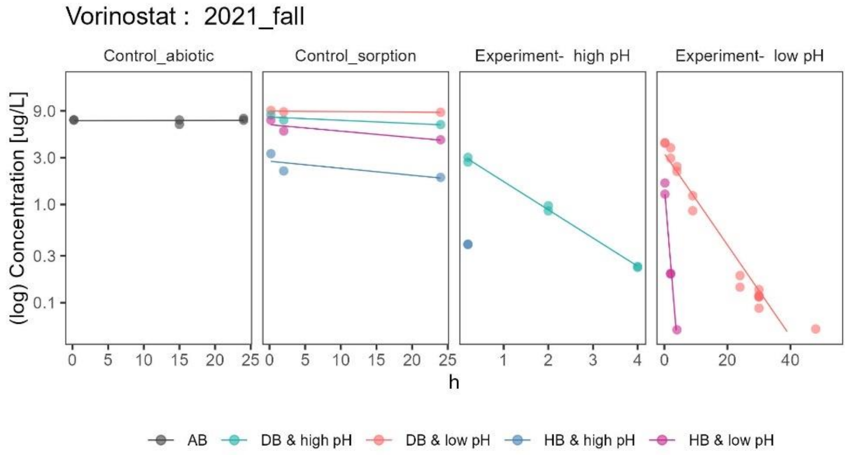

Figure S69. Timeseries of semi-quantified concentrations of vorinostat in experiment 2021-fall

S5. DegT50$_{sludge}$ of tested compounds

S5.1 DegT50$_{sludge}$ calculation

In preparation for DegT50$_{sludge}$ calculations, the time points with concentrations below limits of quantification (Table S4) were removed and concentration-time series of BT incubations with less than 3 time points were excluded. The time series of the spiked controls (SC and AB) were compared to those of the BT incubations. Lower initial concentration levels in both BT and SC compared to AB were interpreted as sorption (with little or no degradation), whereas lower initial concentration levels in BT compared to SC were interpreted as fast degradation in BT.

S5.2 Quality controls and calculation of DegT50$_{sludge}$

The concentrations below LOQs were generally excluded from the concentration-time series used for linear regressions to fit the pseudo-first order kinetic model (see equation below). If the compound dissipated very fast and it had available concentrations above LOQ at the first two time points (i.e., 0 h and 2 h), then the subsequent time point below LOQ (i.e., 4 h) was included in the fits.

$$k_{dis} = \left(\frac{\ln(C_0 - C_t)}{t}\right);\ DegT50 = -\frac{\ln 2}{k_{dis}}$$

We observed that most compounds had at least one concentration-time series where the p-value was not significant and/or R^2 was low but that could be assessed qualitatively (visually) as indicative of fast degradation or very slow degradation (i.e., persistence). Calculating the DegT50$_{sludge}$ mean values without this information (only with correctly fitted kinetics) would be biased. Therefore, for compounds with fast dissipation, a lower reporting limit (LRL) was imputed. The LRL was determined to be 1.2 h (0.05 days), given that at least three time points (0, 2 h and 4 h) were required to fit a first-order kinetics regression and assuming a limit of quantification of 10% of the spiked concentration for all parent compounds. The LRL was derived as follows:

$$k_{dis-LRL} = \left(\frac{\ln(100\% - 90\%)}{4h}\right) = -0.57564\ ;\ DegT50_{LRL} = 1.204\ h$$

For compounds with slow dissipation (k_{dis} not significantly different from zero) or even formation (k_{dis} >0), the upper reporting limit (URL) was set as ~315 h (13.16 days) for 48 h experiments and as ~473h (19.74 days) for 72 h experiments, based on the assumption that a DegT50 can be accurately calculated only if at least 10% dissipation of the compound's initial amount is observed during the time span of the experiment.

$$k_{dis-URL-48h} = \left(\frac{\ln(100\% - 10\%)}{48h}\right) = -0.002195;\ DegT50_{LRL-48h} = 315.8\ h$$

$$k_{dis-URL-72h} = \left(\frac{\ln(100\% - 10\%)}{72h}\right) = -0.001463;\ DegT50_{LRL-72h} = 473.7\ h$$

S5.3 All DegT50$_{sludge}$ and kinetic fit parameters

In Table S8, the DegT50$_{sludge}$ are listed for each incubation, experiment and compound. The kinetic fit parameters (R^2 and p-value) correspond to the results of the linear regression assuming 1st order degradation. The DegT50 model column shows whether a censored value for the upper or lower reporting limit of DegT50 were used for the classification models and/or calculation of the mean log DegT50. NA's in DegT50 raw values indicate that no rate constant was derived because less than three concentration-time points were available.

Table S6. DT50$_{sludge}$ [h] of reference compounds, APIs and PPPs tested in all sludge experiments by incubation. Incubations using activated sludge from the WWTP in Eawag-experimental hall are indicated in the biomass column as HB-VH. All other incubations use activated sludge from the WWTP Neugut.

Compound	Group	Experiment	Biomass	pH level	n	DegT50 raw [h]	Rsq	p- value	DegT50 model [h]
Acalabrutinib	API	2021_fall	DB	low pH	8	473	31.5%	0.148	>474
Acalabrutinib	API	2021_fall	DB	low pH	8	878	18.3%	0.290	>474
Acalabrutinib	API	2021_fall	DB	high pH	8	130	71.3%	0.008	130
Acalabrutinib	API	2021_fall	DB	high pH	8	95.9	95.1%	0.000	95.9
Acalabrutinib	API	2021_fall	HB	low pH	8	59.8	92.8%	0.000	59.8
Acalabrutinib	API	2021_fall	HB	low pH	8	31.4	97.8%	0.000	31.4
Acalabrutinib	API	2021_fall	HB	high pH	3	3.86	99.7%	0.033	3.86
Acalabrutinib	API	2021_fall	HB	high pH	3	3.33	97.3%	0.106	3.33
Aliskiren	API	2021_fall	HB	low pH	9	-202	51.6%	0.029	>474
Aliskiren	API	2021_fall	HB	low pH	9	806	8.7%	0.441	>474
Aliskiren	API	2021_fall	DB	low pH	9	41162	0.0%	0.986	>474
Aliskiren	API	2021_fall	DB	low pH	9	357	41.9%	0.060	357
Aliskiren	API	2021_fall	DB	high pH	9	145	70.8%	0.004	145
Aliskiren	API	2021_fall	DB	high pH	9	140	90.6%	0.000	140
Aliskiren	API	2021_fall	HB	high pH	9	92.1	95.5%	0.000	92.1
Aliskiren	API	2021_fall	HB	high pH	9	83.8	94.2%	0.000	83.8
Amlodipine	API	2021_fall	HB	high pH	3	7.14	89.5%	0.211	<1.2
Amlodipine	API	2021_fall	HB	high pH	3	7.57	97.3%	0.105	<1.2
Amlodipine	API	2021_fall	DB	low pH	9	92.0	90.1%	0.000	92.0
Amlodipine	API	2021_fall	DB	low pH	9	88.9	89.4%	0.000	88.9
Amlodipine	API	2021_fall	HB	low pH	9	82.1	90.8%	0.000	82.1
Amlodipine	API	2021_fall	HB	low pH	9	67.6	86.2%	0.000	67.6
Amlodipine	API	2021_fall	DB	high pH	6	25.3	87.1%	0.007	25.3
Amlodipine	API	2021_fall	DB	high pH	6	25.0	81.5%	0.014	25.0
Asulam	PPP	2018_summer	DB	high pH	6	-17716	0.2%	0.933	>316
Asulam	PPP	2018_summer	DB	high pH	6	5653	3.1%	0.739	>316
Asulam	PPP	2018_summer	DB	high pH	6	74627	0.0%	0.991	>316
Asulam	PPP	2018_summer	NB	high pH	5	122	97.1%	0.002	122
Asulam	PPP	2018_summer	NB	high pH	5	107	95.2%	0.004	107
Asulam	PPP	2018_summer	NB	high pH	5	106	98.9%	0.001	106
Asulam	PPP	2018_summer	HB	high pH	6	17.8	98.1%	0.000	17.8
Asulam	PPP	2018_summer	HB	high pH	6	17.6	98.5%	0.000	17.6

Asulam	PPP	2018_summer	HB	high pH	6	17.0	99.4%	0.000	17.0
Atazanavir	API	2021_fall	HB	low pH	9	-668	25.1%	0.169	>474
Atazanavir	API	2021_fall	HB	low pH	9	384	40.4%	0.066	>474
Atazanavir	API	2021_fall	DB	low pH	9	581	36.9%	0.083	>474
Atazanavir	API	2021_fall	DB	low pH	9	239	77.1%	0.002	239
Atazanavir	API	2021_fall	DB	high pH	9	211	88.0%	0.000	211
Atazanavir	API	2021_fall	DB	high pH	9	201	59.9%	0.014	201
Atazanavir	API	2021_fall	HB	high pH	9	75.0	91.7%	0.000	75.0
Atazanavir	API	2021_fall	HB	high pH	9	68.8	92.8%	0.000	68.8
Atomoxetin	API	2021_fall	HB	low pH	9	-6101	1.7%	0.737	>474
Atomoxetin	API	2021_fall	DB	low pH	9	-1337	17.2%	0.267	>474
Atomoxetin	API	2021_fall	DB	low pH	9	-943	20.3%	0.224	>474
Atomoxetin	API	2021_fall	HB	low pH	9	-921	17.6%	0.261	>474
Atomoxetin	API	2021_fall	HB	high pH	9	-78.4	71.3%	0.004	>474
Atomoxetin	API	2021_fall	HB	high pH	9	-75.8	74.0%	0.003	>474
Atomoxetin	API	2021_fall	DB	high pH	9	391	24.2%	0.178	>474
Atomoxetin	API	2021_fall	DB	high pH	9	465	8.4%	0.448	>474
Atovaquone	API	2021_fall	DB	low pH	8	-922	1.9%	0.744	NA
Atovaquone	API	2021_fall	DB	low pH	6	159	39.6%	0.181	NA
Atovaquone	API	2021_fall	DB	high pH	8	45.2	92.6%	0.000	45.2
Atovaquone	API	2021_fall	DB	high pH	8	42.0	89.9%	0.000	42.0
Azoxystrobin	reference	2018_summer	DB	high pH	6	112	99.2%	0.000	112
Azoxystrobin	reference	2018_summer	DB	high pH	6	111	97.7%	0.000	111
Azoxystrobin	reference	2018_summer	DB	high pH	6	104	93.3%	0.002	104
Azoxystrobin	reference	2018_summer	NB	high pH	5	29.0	99.6%	0.000	29.0
Azoxystrobin	reference	2018_summer	NB	high pH	5	28.9	99.7%	0.000	28.9
Azoxystrobin	reference	2018_summer	NB	high pH	5	28.7	99.6%	0.000	28.7
Azoxystrobin	reference	2018_summer	HB	high pH	6	21.6	96.9%	0.000	21.6
Azoxystrobin	reference	2018_summer	HB	high pH	6	21.5	97.0%	0.000	21.5
Azoxystrobin	reference	2018_summer	HB	high pH	6	21.4	96.9%	0.000	21.4
Azoxystrobin	reference	2020_fall	DB	high pH	7	257	84.1%	0.010	257
Azoxystrobin	reference	2020_fall	DB	low pH	7	217	89.4%	0.004	219
Azoxystrobin	reference	2020_fall	DB	low pH	7	193	89.7%	0.004	192
Azoxystrobin	reference	2020_fall	DB	high pH	7	182	97.2%	0.000	181
Azoxystrobin	reference	2020_fall	HB	high pH	6	62.5	83.9%	0.029	62.2
Azoxystrobin	reference	2020_fall	HB	high pH	7	55.0	82.0%	0.013	54.9
Azoxystrobin	reference	2020_fall	HB	low pH	7	49.2	94.7%	0.001	49.3
Azoxystrobin	reference	2020_fall	HB	low pH	7	45.6	98.4%	0.000	45.7
Azoxystrobin	reference	2021_fall	DB	low pH	9	-4690	0.8%	0.819	>474
Azoxystrobin	reference	2021_fall	DB	low pH	9	2228	3.3%	0.642	>474
Azoxystrobin	reference	2021_fall	HB	low pH	9	287	73.0%	0.003	287
Azoxystrobin	reference	2021_fall	HB	low pH	9	270	83.4%	0.001	270
Azoxystrobin	reference	2021_fall	DB	high pH	9	217	63.7%	0.010	217
Azoxystrobin	reference	2021_fall	DB	high pH	9	119	92.4%	0.000	119
Azoxystrobin	reference	2021_fall	HB	high pH	9	26.5	99.0%	0.000	26.5
Azoxystrobin	reference	2021_fall	HB	high pH	9	22.3	99.5%	0.000	22.3
Azoxystrobin	reference	2021_summer	HB	low pH	9	287	81.3%	0.001	287
Azoxystrobin	reference	2021_summer	HB	low pH	9	258	89.6%	0.000	258
Azoxystrobin	reference	2021_summer	HB_VH	low pH	9	175	73.6%	0.003	175
Azoxystrobin	reference	2021_summer	HB_VH	low pH	9	151	78.2%	0.002	151
Azoxystrobin	reference	2021_summer	HB	high pH	6	14.5	97.6%	0.000	14.5
Azoxystrobin	reference	2021_summer	HB	high pH	6	11.8	99.8%	0.000	11.8
Azoxystrobin	reference	2021_summer	HB_VH	high pH	5	8.36	99.9%	0.000	8.36
Azoxystrobin	reference	2021_summer	HB_VH	high pH	4	7.46	100.0%	0.000	7.46
Azoxystrobin	reference	2021_winter	DB	high pH	6	385	62.9%	0.060	385
Azoxystrobin	reference	2021_winter	DB	low pH	6	-578	45.7%	0.140	>316
Azoxystrobin	reference	2021_winter	DB	low pH	6	217	55.4%	0.090	>316

Azoxystrobin	reference	2021_winter	DB	low pH	6	495	36.9%	0.201	>316
Azoxystrobin	reference	2021_winter	DB	high pH	6	315	92.0%	0.002	315
Azoxystrobin	reference	2021_winter	DB	high pH	6	289	85.4%	0.008	289
Azoxystrobin	reference	2021_winter	HB	low pH	6	165	84.7%	0.009	166
Azoxystrobin	reference	2021_winter	HB	low pH	6	151	82.0%	0.013	150
Azoxystrobin	reference	2021_winter	HB	low pH	6	136	85.0%	0.009	137
Azoxystrobin	reference	2021_winter	HB	high pH	6	120	79.6%	0.017	119
Azoxystrobin	reference	2021_winter	HB	high pH	6	117	96.5%	0.000	117
Azoxystrobin	reference	2021_winter	HB	high pH	6	92.4	96.3%	0.001	92.8
Benzovindiflupyr	reference	2018_summer	NB	high pH	5	236	58.6%	0.131	>316
Benzovindiflupyr	reference	2018_summer	HB	high pH	6	287	48.3%	0.125	>316
Benzovindiflupyr	reference	2018_summer	HB	high pH	6	353	39.2%	0.183	>316
Benzovindiflupyr	reference	2018_summer	NB	high pH	5	355	37.1%	0.276	>316
Benzovindiflupyr	reference	2018_summer	DB	high pH	6	433	68.3%	0.043	>316
Benzovindiflupyr	reference	2018_summer	HB	high pH	6	439	28.7%	0.273	>316
Benzovindiflupyr	reference	2018_summer	NB	high pH	5	458	67.8%	0.087	>316
Benzovindiflupyr	reference	2018_summer	DB	high pH	6	8861	0.2%	0.934	>316
Benzovindiflupyr	reference	2018_summer	DB	high pH	6	213	90.9%	0.003	213
Benzovindiflupyr	reference	2020_fall	HB	high pH	7	158	71.9%	0.033	159
Benzovindiflupyr	reference	2020_fall	DB	low pH	7	144	85.5%	0.008	143
Benzovindiflupyr	reference	2020_fall	HB	high pH	6	126	95.0%	0.005	127
Benzovindiflupyr	reference	2020_fall	DB	low pH	7	114	80.4%	0.016	113
Benzovindiflupyr	reference	2020_fall	DB	high pH	7	85.6	85.9%	0.008	85.4
Benzovindiflupyr	reference	2020_fall	DB	high pH	7	76.2	91.9%	0.003	75.8
Benzovindiflupyr	reference	2020_fall	DB	low pH	5	35.7	83.8%	0.084	35.7
Benzovindiflupyr	reference	2020_fall	HB	low pH	5	35.4	97.7%	0.012	35.4
Benzovindiflupyr	reference	2021_fall	HB	high pH	8	516	24.2%	0.216	>474
Benzovindiflupyr	reference	2021_fall	HB	low pH	8	901	15.2%	0.340	>474
Benzovindiflupyr	reference	2021_fall	DB	low pH	8	458	58.4%	0.027	458
Benzovindiflupyr	reference	2021_fall	HB	low pH	8	424	61.8%	0.021	424
Benzovindiflupyr	reference	2021_fall	DB	low pH	8	341	55.6%	0.034	341
Benzovindiflupyr	reference	2021_fall	DB	high pH	8	325	76.0%	0.005	325
Benzovindiflupyr	reference	2021_fall	DB	high pH	8	271	87.2%	0.001	271
Benzovindiflupyr	reference	2021_fall	HB	high pH	8	262	75.2%	0.005	262
Benzovindiflupyr	reference	2021_summer	HB_VH	high pH	9	409	20.5%	0.221	>474
Benzovindiflupyr	reference	2021_summer	HB_VH	high pH	9	411	23.2%	0.189	>474
Benzovindiflupyr	reference	2021_summer	HB	low pH	9	632	12.1%	0.359	>474
Benzovindiflupyr	reference	2021_summer	HB	low pH	9	641	9.3%	0.425	>474
Benzovindiflupyr	reference	2021_summer	HB_VH	low pH	9	2922	0.8%	0.818	>474
Benzovindiflupyr	reference	2021_summer	HB_VH	low pH	9	12594	0.0%	0.972	>474
Benzovindiflupyr	reference	2021_summer	HB	high pH	9	233	49.2%	0.035	233
Benzovindiflupyr	reference	2021_summer	HB	high pH	9	135	69.8%	0.005	135
Benzovindiflupyr	reference	2021_winter	DB	low pH	6	-330	34.0%	0.225	>316
Benzovindiflupyr	reference	2021_winter	HB	high pH	6	-267	20.3%	0.369	>316
Benzovindiflupyr	reference	2021_winter	HB	high pH	6	-141	37.5%	0.196	>316
Benzovindiflupyr	reference	2021_winter	HB	high pH	6	-102	41.8%	0.165	>316
Benzovindiflupyr	reference	2021_winter	HB	low pH	6	110	30.3%	0.258	>316
Benzovindiflupyr	reference	2021_winter	HB	low pH	6	128	20.4%	0.369	>316
Benzovindiflupyr	reference	2021_winter	HB	low pH	6	169	14.7%	0.453	>316
Benzovindiflupyr	reference	2021_winter	DB	low pH	6	224	46.6%	0.135	>316
Benzovindiflupyr	reference	2021_winter	DB	high pH	6	289	29.3%	0.267	>316
Benzovindiflupyr	reference	2021_winter	DB	low pH	6	365	29.0%	0.271	>316
Benzovindiflupyr	reference	2021_winter	DB	high pH	6	385	38.7%	0.187	>316
Benzovindiflupyr	reference	2021_winter	DB	high pH	6	224	73.3%	0.029	227
Bixafen	PPP	2018_summer	HB	high pH	6	327	73.2%	0.030	327
Bixafen	PPP	2018_summer	DB	high pH	6	-13321	0.1%	0.953	>316
Bixafen	PPP	2018_summer	HB	high pH	6	222	60.9%	0.067	>316

Bixafen	PPP	2018_summer	NB	high pH	5	444	46.7%	0.204	>316
Bixafen	PPP	2018_summer	DB	high pH	6	519	27.1%	0.290	>316
Bixafen	PPP	2018_summer	NB	high pH	5	620	28.3%	0.356	>316
Bixafen	PPP	2018_summer	NB	high pH	5	624	19.2%	0.461	>316
Bixafen	PPP	2018_summer	DB	high pH	6	1532	5.3%	0.661	>316
Bixafen	PPP	2018_summer	HB	high pH	6	182	86.1%	0.008	182
Bromoxynil	reference	2021_fall	HB	low pH	2	NA	NA	NA	<1.2
Bromoxynil	reference	2021_fall	HB	low pH	2	NA	NA	NA	<1.2
Bromoxynil	reference	2021_fall	DB	high pH	9	273	78.6%	0.001	273
Bromoxynil	reference	2021_fall	DB	high pH	9	228	79.8%	0.001	228
Bromoxynil	reference	2021_fall	DB	low pH	4	6.38	99.2%	0.004	6.38
Bromoxynil	reference	2021_fall	DB	low pH	4	5.56	99.2%	0.004	5.56
Bromoxynil	reference	2021_fall	HB	high pH	4	3.95	97.4%	0.013	3.95
Bromoxynil	reference	2021_fall	HB	high pH	4	3.64	98.3%	0.008	3.64
Bromoxynil	reference	2021_summer	HB	low pH	2	0.74	100.0%	NA	<1.2
Bromoxynil	reference	2021_summer	HB	low pH	2	0.99	100.0%	NA	<1.2
Bromoxynil	reference	2021_summer	HB_VH	high pH	6	21.7	93.8%	0.001	21.7
Bromoxynil	reference	2021_summer	HB_VH	high pH	7	18.9	96.5%	0.000	18.9
Bromoxynil	reference	2021_summer	HB	high pH	4	6.41	99.7%	0.002	6.41
Bromoxynil	reference	2021_summer	HB_VH	low pH	4	5.36	98.6%	0.007	5.36
Bromoxynil	reference	2021_summer	HB_VH	low pH	4	5.33	97.6%	0.012	5.33
Bromoxynil	reference	2021_summer	HB	high pH	4	4.49	99.6%	0.002	4.49
Budesonide	API	2021_fall	HB	high pH	3	3.89	98.9%	0.068	<1.2
Budesonide	API	2021_fall	HB	high pH	3	4.14	97.4%	0.104	<1.2
Budesonide	API	2021_fall	DB	low pH	8	174	95.9%	0.000	174
Budesonide	API	2021_fall	DB	low pH	8	164	95.2%	0.000	164
Budesonide	API	2021_fall	DB	high pH	8	32.4	98.9%	0.000	32.4
Budesonide	API	2021_fall	DB	high pH	8	31.0	97.7%	0.000	31.0
Budesonide	API	2021_fall	HB	low pH	5	7.74	99.4%	0.000	7.74
Budesonide	API	2021_fall	HB	low pH	5	5.94	97.5%	0.002	5.94
Canagliflozin	API	2021_fall	HB	high pH	4	-86.8	0.9%	0.906	<1.2
Canagliflozin	API	2021_fall	HB	high pH	4	195	0.3%	0.945	<1.2
Canagliflozin	API	2021_fall	DB	low pH	9	95.4	91.5%	0.000	95.4
Canagliflozin	API	2021_fall	DB	low pH	9	90.6	88.9%	0.000	90.6
Canagliflozin	API	2021_fall	HB	low pH	5	68.6	40.2%	0.250	68.6
Canagliflozin	API	2021_fall	HB	low pH	6	40.6	93.8%	0.001	40.6
Canagliflozin	API	2021_fall	DB	high pH	9	40.0	98.4%	0.000	40.0
Canagliflozin	API	2021_fall	DB	high pH	9	37.3	98.7%	0.000	37.3
Carbendazim	PPP	2018_summer	DB	high pH	6	-52309	0.0%	0.970	>316
Carbendazim	PPP	2018_summer	DB	high pH	6	-2310	9.7%	0.547	>316
Carbendazim	PPP	2018_summer	NB	high pH	5	573	82.4%	0.033	>316
Carbendazim	PPP	2018_summer	NB	high pH	5	673	64.2%	0.103	>316
Carbendazim	PPP	2018_summer	NB	high pH	5	675	90.0%	0.014	>316
Carbendazim	PPP	2018_summer	DB	high pH	6	9764	2.0%	0.789	>316
Carbendazim	PPP	2018_summer	HB	high pH	6	86.2	99.0%	0.000	86.2
Carbendazim	PPP	2018_summer	HB	high pH	6	78.4	98.8%	0.000	78.4
Carbendazim	PPP	2018_summer	HB	high pH	6	74.5	98.6%	0.000	74.5
Carbetamide	PPP	2018_summer	DB	high pH	6	581	39.2%	0.184	>316
Carbetamide	PPP	2018_summer	DB	high pH	6	195	85.1%	0.009	195
Carbetamide	PPP	2018_summer	DB	high pH	6	177	82.9%	0.012	177
Carbetamide	PPP	2018_summer	NB	high pH	5	168	87.1%	0.020	168
Carbetamide	PPP	2018_summer	NB	high pH	5	125	93.5%	0.007	125
Carbetamide	PPP	2018_summer	NB	high pH	5	125	99.9%	0.000	125
Carbetamide	PPP	2018_summer	HB	high pH	6	60.7	99.9%	0.000	60.7
Carbetamide	PPP	2018_summer	HB	high pH	6	52.4	99.7%	0.000	52.4
Carbetamide	PPP	2018_summer	HB	high pH	6	51.9	98.7%	0.000	51.9
Ceritinib	API	2021_fall	DB	low pH	8	-3957	1.2%	0.794	>474

Ceritinib	API	2021_fall	DB	high pH	4	-3738	5.4%	0.767	>474
Ceritinib	API	2021_fall	DB	low pH	8	1113	15.0%	0.343	>474
Ceritinib	API	2021_fall	DB	high pH	2	NA	NA	NA	>474
Chlorantraniliprole	PPP	2018_summer	HB	high pH	6	328	70.7%	0.036	328
Chlorantraniliprole	PPP	2018_summer	DB	high pH	6	377	35.4%	0.213	>316
Chlorantraniliprole	PPP	2018_summer	NB	high pH	5	439	77.5%	0.049	>316
Chlorantraniliprole	PPP	2018_summer	DB	high pH	6	736	34.4%	0.221	>316
Chlorantraniliprole	PPP	2018_summer	NB	high pH	5	819	45.1%	0.214	>316
Chlorantraniliprole	PPP	2018_summer	DB	high pH	6	841	26.1%	0.300	>316
Chlorantraniliprole	PPP	2018_summer	NB	high pH	5	361384	0.0%	0.998	>316
Chlorantraniliprole	PPP	2018_summer	HB	high pH	6	244	80.6%	0.015	244
Chlorantraniliprole	PPP	2018_summer	HB	high pH	6	169	65.1%	0.052	169
Clomazone	PPP	2018_summer	DB	high pH	6	707	48.9%	0.122	>316
Clomazone	PPP	2018_summer	DB	high pH	6	953	27.3%	0.287	>316
Clomazone	PPP	2018_summer	DB	high pH	6	3752	1.9%	0.794	>316
Clomazone	PPP	2018_summer	NB	high pH	5	134	95.8%	0.004	134
Clomazone	PPP	2018_summer	NB	high pH	5	126	95.2%	0.005	126
Clomazone	PPP	2018_summer	NB	high pH	5	122	96.9%	0.002	122
Clomazone	PPP	2018_summer	HB	high pH	6	29.0	98.6%	0.000	29.0
Clomazone	PPP	2018_summer	HB	high pH	6	28.4	97.6%	0.000	28.4
Clomazone	PPP	2018_summer	HB	high pH	6	27.7	98.3%	0.000	27.7
Clopidogrel	API	2021_fall	DB	low pH	8	698	57.4%	0.029	>474
Clopidogrel	API	2021_fall	DB	low pH	8	381	77.6%	0.004	381
Clopidogrel	API	2021_fall	HB	low pH	8	152	91.0%	0.000	152
Clopidogrel	API	2021_fall	HB	low pH	8	144	98.2%	0.000	144
Clopidogrel	API	2021_fall	DB	high pH	8	134	95.5%	0.000	134
Clopidogrel	API	2021_fall	DB	high pH	8	119	96.8%	0.000	119
Clopidogrel	API	2021_fall	HB	high pH	8	33.6	94.8%	0.000	33.6
Clopidogrel	API	2021_fall	HB	high pH	8	32.1	96.8%	0.000	32.1
Clothianidin	PPP	2018_summer	DB	high pH	6	-39995	0.0%	0.975	>316
Clothianidin	PPP	2018_summer	NB	high pH	5	-24921	0.1%	0.966	>316
Clothianidin	PPP	2018_summer	NB	high pH	5	-7683	3.0%	0.781	>316
Clothianidin	PPP	2018_summer	NB	high pH	5	-4839	9.8%	0.608	>316
Clothianidin	PPP	2018_summer	DB	high pH	6	-609	55.8%	0.088	>316
Clothianidin	PPP	2018_summer	HB	high pH	6	496	76.7%	0.022	>316
Clothianidin	PPP	2018_summer	HB	high pH	6	583	68.1%	0.043	>316
Clothianidin	PPP	2018_summer	HB	high pH	6	628	61.2%	0.066	>316
Clothianidin	PPP	2018_summer	DB	high pH	6	3787	2.6%	0.758	>316
Clotrimazol	API	2021_fall	DB	low pH	8	-356	3.3%	0.667	>474
Clotrimazol	API	2021_fall	DB	low pH	8	287	12.1%	0.398	>474
Clotrimazol	API	2021_fall	DB	high pH	8	126	87.8%	0.001	126
Clotrimazol	API	2021_fall	DB	high pH	8	125	67.3%	0.013	125
Cyantraniliprole	reference	2018_summer	HB	high pH	6	256	79.3%	0.017	256
Cyantraniliprole	reference	2018_summer	HB	high pH	6	230	91.4%	0.003	230
Cyantraniliprole	reference	2018_summer	HB	high pH	6	210	79.0%	0.018	210
Cyantraniliprole	reference	2018_summer	NB	high pH	5	146	96.0%	0.003	146
Cyantraniliprole	reference	2018_summer	NB	high pH	5	123	94.3%	0.006	123
Cyantraniliprole	reference	2018_summer	NB	high pH	5	120	90.7%	0.012	120
Cyantraniliprole	reference	2018_summer	DB	high pH	6	97.0	91.7%	0.003	97.0
Cyantraniliprole	reference	2018_summer	DB	high pH	6	78.7	96.6%	0.000	78.7
Cyantraniliprole	reference	2018_summer	DB	high pH	6	74.7	96.9%	0.000	74.7
Cyantraniliprole	reference	2020_fall	DB	high pH	7	408	83.0%	0.012	408
Cyantraniliprole	reference	2020_fall	HB	high pH	7	365	78.4%	0.019	364
Cyantraniliprole	reference	2020_fall	HB	high pH	6	347	96.8%	0.002	352
Cyantraniliprole	reference	2020_fall	HB	low pH	7	408	25.9%	0.302	>316
Cyantraniliprole	reference	2020_fall	DB	high pH	7	462	67.4%	0.045	>316
Cyantraniliprole	reference	2020_fall	DB	low pH	7	990	28.2%	0.279	>316

Cyantraniliprole	reference	2020_fall	DB	low pH	7	1155	22.2%	0.345	>316
Cyantraniliprole	reference	2020_fall	HB	low pH	7	3466	25.4%	0.308	>316
Cyantraniliprole	reference	2021_fall	HB	low pH	8	-4848	5.9%	0.561	>474
Cyantraniliprole	reference	2021_fall	DB	low pH	8	-3728	15.5%	0.335	>474
Cyantraniliprole	reference	2021_fall	HB	high pH	8	-1658	9.0%	0.470	>474
Cyantraniliprole	reference	2021_fall	HB	high pH	8	-854	40.9%	0.088	>474
Cyantraniliprole	reference	2021_fall	DB	low pH	8	1594	28.7%	0.171	>474
Cyantraniliprole	reference	2021_fall	HB	low pH	8	3503	10.8%	0.428	>474
Cyantraniliprole	reference	2021_fall	DB	high pH	8	79.8	99.0%	0.000	79.8
Cyantraniliprole	reference	2021_fall	DB	high pH	8	72.2	99.0%	0.000	72.2
Cyantraniliprole	reference	2021_summer	HB	high pH	9	868	61.4%	0.013	>474
Cyantraniliprole	reference	2021_summer	HB_VH	low pH	9	1100	48.0%	0.039	>474
Cyantraniliprole	reference	2021_summer	HB_VH	low pH	9	1256	27.8%	0.145	>474
Cyantraniliprole	reference	2021_summer	HB	high pH	9	1460	40.9%	0.064	>474
Cyantraniliprole	reference	2021_summer	HB	low pH	9	1712	13.0%	0.341	>474
Cyantraniliprole	reference	2021_summer	HB	low pH	9	1771	5.7%	0.535	>474
Cyantraniliprole	reference	2021_summer	HB_VH	high pH	9	422	82.9%	0.001	422
Cyantraniliprole	reference	2021_summer	HB_VH	high pH	9	271	90.9%	0.000	271
Cyantraniliprole	reference	2021_winter	HB	low pH	6	-3466	0.4%	0.902	>316
Cyantraniliprole	reference	2021_winter	HB	high pH	6	154	40.7%	0.173	>316
Cyantraniliprole	reference	2021_winter	DB	low pH	6	169	47.2%	0.132	>316
Cyantraniliprole	reference	2021_winter	HB	low pH	6	365	34.2%	0.222	>316
Cyantraniliprole	reference	2021_winter	HB	low pH	6	866	7.2%	0.607	>316
Cyantraniliprole	reference	2021_winter	DB	low pH	6	1155	2.6%	0.762	>316
Cyantraniliprole	reference	2021_winter	DB	low pH	6	133	60.6%	0.068	134
Cyantraniliprole	reference	2021_winter	HB	high pH	6	108	62.1%	0.063	109
Cyantraniliprole	reference	2021_winter	HB	high pH	6	76.2	81.3%	0.014	76.1
Cyantraniliprole	reference	2021_winter	DB	high pH	6	75.3	73.6%	0.029	75.7
Cyantraniliprole	reference	2021_winter	DB	high pH	6	73.7	68.4%	0.042	74.0
Cyantraniliprole	reference	2021_winter	DB	high pH	6	70.7	87.1%	0.007	70.8
Cyclaniliprole	reference	2018_summer	DB	high pH	6	-2654	0.7%	0.878	>316
Cyclaniliprole	reference	2018_summer	NB	high pH	5	694	20.4%	0.446	>316
Cyclaniliprole	reference	2018_summer	NB	high pH	5	750	22.4%	0.421	>316
Cyclaniliprole	reference	2018_summer	NB	high pH	5	809	85.4%	0.025	>316
Cyclaniliprole	reference	2018_summer	DB	high pH	6	4158	1.4%	0.826	>316
Cyclaniliprole	reference	2018_summer	HB	high pH	6	259	73.5%	0.029	259
Cyclaniliprole	reference	2018_summer	DB	high pH	6	217	65.9%	0.050	217
Cyclaniliprole	reference	2018_summer	HB	high pH	6	165	79.7%	0.017	165
Cyclaniliprole	reference	2018_summer	HB	high pH	6	126	64.6%	0.054	126
Cyclaniliprole	reference	2020_fall	HB	high pH	7	347	65.3%	0.052	347
Cyclaniliprole	reference	2020_fall	DB	low pH	7	-3466	2.8%	0.752	>316
Cyclaniliprole	reference	2020_fall	DB	high pH	7	462	64.9%	0.053	>316
Cyclaniliprole	reference	2020_fall	DB	low pH	6	495	13.5%	0.542	>316
Cyclaniliprole	reference	2020_fall	HB	high pH	6	533	58.8%	0.131	>316
Cyclaniliprole	reference	2020_fall	DB	high pH	7	630	93.0%	0.002	>316
Cyclaniliprole	reference	2020_fall	DB	low pH	7	693	51.1%	0.110	>316
Cyclaniliprole	reference	2020_fall	HB	low pH	7	1733	3.9%	0.708	>316
Cyclaniliprole	reference	2021_fall	HB	high pH	8	-22802	0.1%	0.943	>474
Cyclaniliprole	reference	2021_fall	DB	low pH	8	-7402	0.6%	0.853	>474
Cyclaniliprole	reference	2021_fall	DB	high pH	8	-1757	17.3%	0.306	>474
Cyclaniliprole	reference	2021_fall	DB	low pH	8	-1245	88.8%	0.000	>474
Cyclaniliprole	reference	2021_fall	DB	high pH	8	-1002	45.4%	0.067	>474
Cyclaniliprole	reference	2021_fall	HB	low pH	8	1540	25.9%	0.198	>474
Cyclaniliprole	reference	2021_fall	HB	high pH	8	2164	12.7%	0.386	>474
Cyclaniliprole	reference	2021_fall	HB	low pH	8	6474	1.1%	0.803	>474
Cyclaniliprole	reference	2021_summer	HB_VH	low pH	9	-16482	0.2%	0.907	>474
Cyclaniliprole	reference	2021_summer	HB	low pH	9	-5755	1.5%	0.755	>474

Cyclaniliprole	reference	2021_summer	HB	low pH	9	-1581	5.6%	0.541	>474
Cyclaniliprole	reference	2021_summer	HB	high pH	9	802	49.6%	0.034	>474
Cyclaniliprole	reference	2021_summer	HB_VH	high pH	9	1253	37.9%	0.078	>474
Cyclaniliprole	reference	2021_summer	HB_VH	high pH	9	2504	13.9%	0.322	>474
Cyclaniliprole	reference	2021_summer	HB_VH	low pH	9	2645	9.4%	0.423	>474
Cyclaniliprole	reference	2021_summer	HB	high pH	9	3691	2.9%	0.662	>474
Cyclaniliprole	reference	2021_winter	DB	high pH	6	-6931	9.9%	0.543	>316
Cyclaniliprole	reference	2021_winter	DB	low pH	6	-2310	6.2%	0.635	>316
Cyclaniliprole	reference	2021_winter	HB	low pH	6	-2310	12.9%	0.485	>316
Cyclaniliprole	reference	2021_winter	DB	high pH	6	-1386	27.2%	0.289	>316
Cyclaniliprole	reference	2021_winter	HB	low pH	6	-990	29.9%	0.262	>316
Cyclaniliprole	reference	2021_winter	DB	high pH	6	-770	44.4%	0.149	>316
Cyclaniliprole	reference	2021_winter	HB	high pH	6	-770	28.8%	0.273	>316
Cyclaniliprole	reference	2021_winter	DB	low pH	6	-187	70.1%	0.038	>316
Cyclaniliprole	reference	2021_winter	DB	low pH	6	693	16.5%	0.424	>316
Cyclaniliprole	reference	2021_winter	HB	high pH	6	1386	26.7%	0.294	>316
Cyclaniliprole	reference	2021_winter	HB	low pH	6	3466	12.7%	0.488	>316
Cyclaniliprole	reference	2021_winter	HB	high pH	6	6931	0.3%	0.913	>316
Dapagliflozin	API	2021_fall	HB	low pH	3	3.89	97.6%	0.099	<1.2
Dapagliflozin	API	2021_fall	HB	low pH	3	7.23	72.2%	0.354	<1.2
Dapagliflozin	API	2021_fall	HB	high pH	2	NA	NA	NA	<1.2
Dapagliflozin	API	2021_fall	HB	high pH	2	NA	NA	NA	<1.2
Dapagliflozin	API	2021_fall	DB	low pH	9	65.1	92.3%	0.000	65.1
Dapagliflozin	API	2021_fall	DB	low pH	9	46.5	96.2%	0.000	46.5
Dapagliflozin	API	2021_fall	DB	high pH	9	27.5	93.9%	0.000	27.5
Dapagliflozin	API	2021_fall	DB	high pH	7	18.8	94.7%	0.000	18.8
Dasatinib	API	2021_fall	HB	high pH	2	NA	NA	NA	<1.2
Dasatinib	API	2021_fall	HB	high pH	2	NA	NA	NA	<1.2
Dasatinib	API	2021_fall	DB	low pH	9	58.1	81.0%	0.001	58.1
Dasatinib	API	2021_fall	DB	low pH	9	52.9	82.6%	0.001	52.9
Dasatinib	API	2021_fall	HB	low pH	6	27.2	78.0%	0.020	27.2
Dasatinib	API	2021_fall	HB	low pH	6	24.1	88.5%	0.005	24.1
Dasatinib	API	2021_fall	DB	high pH	4	4.62	92.3%	0.040	4.62
Dasatinib	API	2021_fall	DB	high pH	4	4.49	97.0%	0.015	4.49
Dicamba	reference	2018_summer	DB	high pH	6	285	45.0%	0.145	>316
Dicamba	reference	2018_summer	DB	high pH	6	187	81.0%	0.015	187
Dicamba	reference	2018_summer	NB	high pH	5	174	98.3%	0.001	174
Dicamba	reference	2018_summer	NB	high pH	5	159	84.3%	0.028	159
Dicamba	reference	2018_summer	NB	high pH	5	151	91.4%	0.011	151
Dicamba	reference	2018_summer	DB	high pH	6	141	97.1%	0.000	141
Dicamba	reference	2018_summer	HB	high pH	6	96.1	85.1%	0.009	96.1
Dicamba	reference	2018_summer	HB	high pH	6	86.1	95.9%	0.001	86.1
Dicamba	reference	2018_summer	HB	high pH	6	77.4	95.1%	0.001	77.4
Dicamba	reference	2021_fall	DB	low pH	8	588	62.0%	0.020	588
Dicamba	reference	2021_fall	DB	high pH	8	-5059	21.6%	0.246	>474
Dicamba	reference	2021_fall	DB	low pH	8	1342	54.7%	0.036	>474
Dicamba	reference	2021_fall	DB	high pH	8	38900	0.1%	0.941	>474
Dicamba	reference	2021_fall	HB	high pH	8	109	87.3%	0.001	109
Dicamba	reference	2021_fall	HB	high pH	8	107	93.2%	0.000	107
Dicamba	reference	2021_fall	HB	low pH	8	106	95.5%	0.000	106
Dicamba	reference	2021_fall	HB	low pH	8	81.3	98.1%	0.000	81.3
Dicamba	reference	2021_summer	HB_VH	low pH	7	-709	9.9%	0.492	>474
Dicamba	reference	2021_summer	HB_VH	low pH	7	-359	40.1%	0.127	>474
Dicamba	reference	2021_summer	HB_VH	high pH	7	-91.8	77.2%	0.009	>474
Dicamba	reference	2021_summer	HB_VH	high pH	7	-86.8	92.0%	0.001	>474
Dicamba	reference	2021_summer	HB	high pH	7	223	83.1%	0.004	223
Dicamba	reference	2021_summer	HB	high pH	7	212	66.7%	0.025	212

Dicamba	reference	2021_summer	HB	low pH	7	95.4	9544%	0.001	95.4
Dicamba	reference	2021_summer	HB	low pH	7	93.7	9367%	0.000	93.7
Dienogest	API	2021_fall	HB	high pH	2	NA	NA	NA	<1.2
Dienogest	API	2021_fall	HB	high pH	2	NA	NA	NA	<1.2
Dienogest	API	2021_fall	HB	low pH	2	NA	NA	NA	<1.2
Dienogest	API	2021_fall	HB	low pH	2	NA	NA	NA	<1.2
Dienogest	API	2021_fall	DB	low pH	9	27.8	97.0%	0.000	27.8
Dienogest	API	2021_fall	DB	low pH	9	25.6	98.2%	0.000	25.6
Dienogest	API	2021_fall	DB	high pH	4	3.51	91.8%	0.042	3.51
Dienogest	API	2021_fall	DB	high pH	4	3.07	90.8%	0.047	3.07
Dimethenamid	PPP	2018_summer	DB	high pH	6	406	81.4%	0.014	>316
Dimethenamid	PPP	2018_summer	DB	high pH	6	259	87.7%	0.006	259
Dimethenamid	PPP	2018_summer	DB	high pH	6	248	98.1%	0.000	248
Dimethenamid	PPP	2018_summer	NB	high pH	5	57.9	98.7%	0.001	57.9
Dimethenamid	PPP	2018_summer	NB	high pH	5	55.7	99.1%	0.000	55.7
Dimethenamid	PPP	2018_summer	NB	high pH	5	53.7	99.6%	0.000	53.7
Dimethenamid	PPP	2018_summer	HB	high pH	6	18.2	95.9%	0.001	18.2
Dimethenamid	PPP	2018_summer	HB	high pH	6	18.2	96.8%	0.000	18.2
Dimethenamid	PPP	2018_summer	HB	high pH	5	15.0	98.9%	0.000	15.0
Dimoxystrobin	PPP	2018_summer	DB	high pH	8	-2003	14.7%	0.348	>316
Dimoxystrobin	PPP	2018_summer	DB	high pH	8	-1818	10.6%	0.431	>316
Dimoxystrobin	PPP	2018_summer	NB	high pH	8	921	27.5%	0.227	>316
Dimoxystrobin	PPP	2018_summer	NB	high pH	8	13188	0.3%	0.905	>316
Dimoxystrobin	PPP	2018_summer	DB	high pH	8	27450	0.1%	0.958	>316
Dimoxystrobin	PPP	2018_summer	HB	high pH	8	116	99.1%	0.000	116
Dimoxystrobin	PPP	2018_summer	HB	high pH	8	113	90.8%	0.000	113
Diuron	reference	2018_summer	DB	high pH	6	1447	32.5%	0.237	>316
Diuron	reference	2018_summer	DB	high pH	6	5084	8.7%	0.571	>316
Diuron	reference	2018_summer	DB	high pH	6	8618	0.5%	0.890	>316
Diuron	reference	2018_summer	NB	high pH	5	176	97.8%	0.001	176
Diuron	reference	2018_summer	NB	high pH	5	174	96.0%	0.003	174
Diuron	reference	2018_summer	NB	high pH	5	163	98.6%	0.001	163
Diuron	reference	2018_summer	HB	high pH	6	35.1	99.8%	0.000	35.1
Diuron	reference	2018_summer	HB	high pH	6	32.2	99.9%	0.000	32.2
Diuron	reference	2018_summer	HB	high pH	6	31.4	99.8%	0.000	31.4
Diuron	reference	2020_fall	HB	high pH	6	385	97.6%	0.002	380
Diuron	reference	2020_fall	HB	high pH	7	365	76.1%	0.023	357
Diuron	reference	2020_fall	DB	high pH	7	630	90.3%	0.004	>316
Diuron	reference	2020_fall	DB	low pH	7	693	42.6%	0.160	>316
Diuron	reference	2020_fall	DB	low pH	7	990	36.4%	0.205	>316
Diuron	reference	2020_fall	DB	high pH	7	1733	10.8%	0.524	>316
Diuron	reference	2020_fall	HB	low pH	7	68.6	99.0%	0.000	68.3
Diuron	reference	2020_fall	HB	low pH	7	64.2	90.4%	0.004	64.2
Diuron	reference	2021_fall	DB	low pH	8	1144	40.2%	0.091	>474
Diuron	reference	2021_fall	DB	high pH	8	2418	52.4%	0.042	>474
Diuron	reference	2021_fall	DB	high pH	8	2599	20.3%	0.263	>474
Diuron	reference	2021_fall	DB	low pH	8	20686	1.4%	0.783	>474
Diuron	reference	2021_fall	HB	low pH	8	411	90.3%	0.000	411
Diuron	reference	2021_fall	HB	low pH	8	312	98.0%	0.000	312
Diuron	reference	2021_fall	HB	high pH	8	85.3	99.5%	0.000	85.3
Diuron	reference	2021_fall	HB	high pH	8	79.2	98.8%	0.000	79.2
Diuron	reference	2021_summer	HB_VH	low pH	9	211	93.2%	0.000	211
Diuron	reference	2021_summer	HB	low pH	9	204	89.2%	0.000	204
Diuron	reference	2021_summer	HB_VH	low pH	9	193	94.8%	0.000	193
Diuron	reference	2021_summer	HB	low pH	9	189	93.5%	0.000	189
Diuron	reference	2021_summer	HB_VH	high pH	9	98.7	98.5%	0.000	98.7
Diuron	reference	2021_summer	HB	high pH	9	74.3	97.0%	0.000	74.3

Diuron	reference	2021_summer	HB_VH	high pH	9	66.5	98.8%	0.000	66.5
Diuron	reference	2021_summer	HB	high pH	9	66.1	99.5%	0.000	66.1
Diuron	reference	2021_winter	HB	high pH	6	385	88.9%	0.005	376
Diuron	reference	2021_winter	DB	low pH	6	-578	71.1%	0.035	>316
Diuron	reference	2021_winter	DB	low pH	6	433	37.1%	0.199	>316
Diuron	reference	2021_winter	HB	high pH	6	630	32.8%	0.235	>316
Diuron	reference	2021_winter	DB	high pH	6	770	53.6%	0.098	>316
Diuron	reference	2021_winter	DB	low pH	6	990	70.0%	0.038	>316
Diuron	reference	2021_winter	DB	high pH	6	1155	41.6%	0.167	>316
Diuron	reference	2021_winter	DB	high pH	6	1386	74.5%	0.027	>316
Diuron	reference	2021_winter	HB	high pH	6	301	92.8%	0.002	304
Diuron	reference	2021_winter	HB	low pH	6	147	88.9%	0.005	149
Diuron	reference	2021_winter	HB	low pH	6	144	93.8%	0.001	144
Diuron	reference	2021_winter	HB	low pH	6	124	95.2%	0.001	125
Dolutegravir	API	2021_fall	HB	high pH	9	-429	53.5%	0.025	>474
Dolutegravir	API	2021_fall	HB	high pH	9	-308	63.0%	0.011	>474
Dolutegravir	API	2021_fall	DB	low pH	9	328	16.6%	0.276	>474
Dolutegravir	API	2021_fall	DB	low pH	9	399	14.7%	0.308	>474
Dolutegravir	API	2021_fall	HB	low pH	9	1040	5.1%	0.561	>474
Dolutegravir	API	2021_fall	HB	low pH	9	2160	4.7%	0.575	>474
Dolutegravir	API	2021_fall	DB	high pH	9	222	82.6%	0.001	222
Dolutegravir	API	2021_fall	DB	high pH	9	174	73.4%	0.003	174
Duloxetine	API	2021_fall	DB	high pH	9	175	46.0%	0.045	NA
Duloxetine	API	2021_fall	DB	high pH	9	207	22.4%	0.198	NA
Duloxetine	API	2021_fall	DB	low pH	9	-2042	3.7%	0.622	>474
Duloxetine	API	2021_fall	HB	high pH	9	-101	73.2%	0.003	>474
Duloxetine	API	2021_fall	HB	high pH	9	-94.7	73.6%	0.003	>474
Duloxetine	API	2021_fall	HB	low pH	9	1040	15.5%	0.295	>474
Duloxetine	API	2021_fall	HB	low pH	9	2667	1.5%	0.754	>474
Duloxetine	API	2021_fall	DB	low pH	9	3168	2.8%	0.670	>474
Efavirenz	API	2021_fall	DB	low pH	8	371	83.6%	0.001	371
Efavirenz	API	2021_fall	DB	low pH	8	321	83.8%	0.001	321
Efavirenz	API	2021_fall	HB	low pH	8	310	70.1%	0.010	310
Efavirenz	API	2021_fall	DB	high pH	8	284	91.8%	0.000	284
Efavirenz	API	2021_fall	DB	high pH	8	283	78.9%	0.003	283
Efavirenz	API	2021_fall	HB	low pH	8	253	84.0%	0.001	253
Efavirenz	API	2021_fall	HB	high pH	8	96.0	97.0%	0.000	96.0
Efavirenz	API	2021_fall	HB	high pH	8	89.2	97.1%	0.000	89.2
Ezetimibe	API	2021_fall	DB	high pH	7	-321	53.1%	0.063	>474
Ezetimibe	API	2021_fall	DB	high pH	7	-234	62.1%	0.035	>474
Ezetimibe	API	2021_fall	HB	high pH	8	84.9	73.1%	0.007	84.9
Ezetimibe	API	2021_fall	HB	low pH	7	71.4	52.3%	0.066	71.4
Ezetimibe	API	2021_fall	HB	low pH	7	59.8	59.4%	0.043	59.8
Ezetimibe	API	2021_fall	HB	high pH	8	59.6	86.7%	0.001	59.6
Ezetimibe	API	2021_fall	DB	low pH	7	45.2	91.0%	0.001	45.2
Ezetimibe	API	2021_fall	DB	low pH	7	41.0	81.8%	0.005	41.0
Fenhexamid	reference	2018_summer	DB	high pH	6	156	99.6%	0.000	156
Fenhexamid	reference	2018_summer	DB	high pH	6	118	95.2%	0.001	118
Fenhexamid	reference	2018_summer	DB	high pH	6	104	98.6%	0.000	104
Fenhexamid	reference	2018_summer	NB	high pH	5	42.6	99.9%	0.000	42.6
Fenhexamid	reference	2018_summer	NB	high pH	5	42.0	99.9%	0.000	42.0
Fenhexamid	reference	2018_summer	NB	high pH	5	41.5	99.6%	0.000	41.5
Fenhexamid	reference	2018_summer	HB	high pH	4	12.3	97.8%	0.011	12.3
Fenhexamid	reference	2018_summer	HB	high pH	4	12.1	96.9%	0.015	12.1
Fenhexamid	reference	2018_summer	HB	high pH	4	12.1	97.6%	0.012	12.1
Fenhexamid	reference	2020_fall	DB	low pH	7	103	96.6%	0.000	103
Fenhexamid	reference	2020_fall	DB	low pH	7	99.0	98.1%	0.000	98.3

Fenhexamid	reference	2020_fall	DB	high pH	7	90.0	98.0%	0.000	89.8
Fenhexamid	reference	2020_fall	DB	high pH	7	70.0	99.2%	0.000	70.1
Fenhexamid	reference	2020_fall	HB	high pH	6	28.4	99.3%	0.000	28.4
Fenhexamid	reference	2020_fall	HB	high pH	7	24.4	99.4%	0.000	24.4
Fenhexamid	reference	2020_fall	HB	low pH	5	10.9	96.0%	0.020	10.9
Fenhexamid	reference	2020_fall	HB	low pH	5	10.8	99.3%	0.004	10.8
Fenhexamid	reference	2021_fall	DB	low pH	8	380	89.7%	0.000	380
Fenhexamid	reference	2021_fall	DB	low pH	8	289	81.3%	0.002	289
Fenhexamid	reference	2021_fall	DB	high pH	8	233	99.1%	0.000	233
Fenhexamid	reference	2021_fall	DB	high pH	8	223	99.7%	0.000	223
Fenhexamid	reference	2021_fall	HB	low pH	8	48.4	92.1%	0.000	48.4
Fenhexamid	reference	2021_fall	HB	low pH	8	40.2	94.9%	0.000	40.2
Fenhexamid	reference	2021_fall	HB	high pH	6	13.9	92.6%	0.002	13.9
Fenhexamid	reference	2021_fall	HB	high pH	6	13.7	91.8%	0.003	13.7
Fenhexamid	reference	2021_summer	HB_VH	low pH	9	33.7	98.5%	0.000	33.7
Fenhexamid	reference	2021_summer	HB_VH	low pH	9	33.4	99.0%	0.000	33.4
Fenhexamid	reference	2021_summer	HB	low pH	7	23.8	99.6%	0.000	23.8
Fenhexamid	reference	2021_summer	HB	low pH	7	23.2	99.5%	0.000	23.2
Fenhexamid	reference	2021_summer	HB_VH	high pH	7	22.8	99.8%	0.000	22.8
Fenhexamid	reference	2021_summer	HB_VH	high pH	6	16.8	99.2%	0.000	16.8
Fenhexamid	reference	2021_summer	HB	high pH	5	11.6	100.0%	0.000	11.6
Fenhexamid	reference	2021_summer	HB	high pH	5	11.1	99.7%	0.000	11.1
Fenhexamid	reference	2021_winter	DB	low pH	6	210	89.7%	0.004	209
Fenhexamid	reference	2021_winter	DB	high pH	6	107	98.4%	0.000	106
Fenhexamid	reference	2021_winter	DB	high pH	6	103	97.6%	0.000	104
Fenhexamid	reference	2021_winter	DB	low pH	6	102	98.2%	0.000	102
Fenhexamid	reference	2021_winter	DB	high pH	6	102	99.2%	0.000	102
Fenhexamid	reference	2021_winter	DB	low pH	6	100	88.5%	0.005	100
Fenhexamid	reference	2021_winter	HB	high pH	6	32.2	93.1%	0.002	32.2
Fenhexamid	reference	2021_winter	HB	high pH	6	32.1	90.3%	0.004	32.1
Fenhexamid	reference	2021_winter	HB	high pH	6	31.4	87.7%	0.006	31.3
Fenhexamid	reference	2021_winter	HB	low pH	6	28.2	90.4%	0.004	28.1
Fenhexamid	reference	2021_winter	HB	low pH	6	28.0	91.0%	0.003	27.9
Fenhexamid	reference	2021_winter	HB	low pH	6	26.7	90.9%	0.003	26.7
Fenoxycarb	reference	2018_summer	NB	high pH	3	-27999	8.1%	0.816	<1.2
Fenoxycarb	reference	2018_summer	NB	high pH	3	-5450	10.9%	0.786	<1.2
Fenoxycarb	reference	2018_summer	HB	high pH	2	-503	100.0%	NA	<1.2
Fenoxycarb	reference	2018_summer	HB	high pH	2	-121	100.0%	NA	<1.2
Fenoxycarb	reference	2018_summer	DB	high pH	3	22.0	53.0%	0.481	<1.2
Fenoxycarb	reference	2020_fall	DB	high pH	1	NA	NA	NA	<1.2
Fenoxycarb	reference	2020_fall	DB	high pH	1	NA	NA	NA	<1.2
Fenoxycarb	reference	2020_fall	DB	low pH	1	NA	NA	NA	<1.2
Fenoxycarb	reference	2020_fall	DB	low pH	1	NA	NA	NA	<1.2
Fenoxycarb	reference	2020_fall	HB	high pH	1	NA	NA	NA	<1.2
Fenoxycarb	reference	2020_fall	HB	high pH	1	NA	NA	NA	<1.2
Fenoxycarb	reference	2020_fall	HB	low pH	1	NA	NA	NA	<1.2
Fenoxycarb	reference	2020_fall	HB	low pH	1	NA	NA	NA	<1.2
Fenoxycarb	reference	2021_fall	DB	high pH	2	NA	NA	NA	<1.2
Fenoxycarb	reference	2021_fall	DB	high pH	2	NA	NA	NA	<1.2
Fenoxycarb	reference	2021_fall	DB	low pH	2	NA	NA	NA	<1.2
Fenoxycarb	reference	2021_fall	DB	low pH	2	NA	NA	NA	<1.2
Fenoxycarb	reference	2021_fall	HB	high pH	2	NA	NA	NA	<1.2
Fenoxycarb	reference	2021_fall	HB	high pH	2	NA	NA	NA	<1.2
Fenoxycarb	reference	2021_fall	HB	low pH	2	NA	NA	NA	<1.2
Fenoxycarb	reference	2021_fall	HB	low pH	2	NA	NA	NA	<1.2
Fenoxycarb	reference	2021_summer	HB	low pH	1	NA	NA	NA	<1.2
Fenoxycarb	reference	2021_summer	HB	low pH	1	NA	NA	NA	<1.2

Fenoxycarb	reference	2021_summer	HB_VH	high pH	1	NA	NA	NA	<1.2
Fenoxycarb	reference	2021_summer	HB_VH	high pH	1	NA	NA	NA	<1.2
Fenoxycarb	reference	2021_summer	HB_VH	low pH	1	NA	NA	NA	<1.2
Fenoxycarb	reference	2021_summer	HB_VH	low pH	1	NA	NA	NA	<1.2
Fenoxycarb	reference	2021_winter	HB	high pH	2	3.12	100.0%	NA	<1.2
Fenoxycarb	reference	2021_winter	HB	high pH	2	4.53	100.0%	NA	<1.2
Fenoxycarb	reference	2021_winter	HB	high pH	2	5.55	100.0%	NA	<1.2
Fenoxycarb	reference	2021_winter	DB	high pH	0	NA	NA	NA	<1.2
Fenoxycarb	reference	2021_winter	DB	high pH	0	NA	NA	NA	<1.2
Fenoxycarb	reference	2021_winter	DB	high pH	0	NA	NA	NA	<1.2
Fenoxycarb	reference	2021_winter	DB	low pH	0	NA	NA	NA	<1.2
Fenoxycarb	reference	2021_winter	DB	low pH	1	NA	NA	NA	<1.2
Fenoxycarb	reference	2021_winter	DB	low pH	0	NA	NA	NA	<1.2
Fenoxycarb	reference	2021_winter	HB	low pH	0	NA	NA	NA	<1.2
Fenoxycarb	reference	2021_winter	HB	low pH	0	NA	NA	NA	<1.2
Fenoxycarb	reference	2021_winter	HB	low pH	0	NA	NA	NA	<1.2
Fexofenadine	API	2021_fall	DB	low pH	8	-162002	0.0%	0.993	>474
Fexofenadine	API	2021_fall	DB	low pH	8	-2042	16.4%	0.320	>474
Fexofenadine	API	2021_fall	DB	high pH	8	401	65.3%	0.015	401
Fexofenadine	API	2021_fall	DB	high pH	8	397	80.0%	0.003	397
Fexofenadine	API	2021_fall	HB	low pH	8	361	89.0%	0.000	361
Fexofenadine	API	2021_fall	HB	low pH	8	240	84.7%	0.001	240
Fexofenadine	API	2021_fall	HB	high pH	8	110	97.5%	0.000	110
Fexofenadine	API	2021_fall	HB	high pH	8	99.6	97.9%	0.000	99.6
Fingolimod	API	2021_fall	DB	low pH	3	9.11	86.6%	0.238	<1.2
Fingolimod	API	2021_fall	DB	low pH	3	13.8	72.3%	0.353	<1.2
Fingolimod	API	2021_fall	DB	high pH	3	23.3	72.3%	0.353	<1.2
Fingolimod	API	2021_fall	DB	high pH	3	29.0	69.6%	0.372	<1.2
Fipronil	reference	2018_summer	DB	high pH	6	302	67.5%	0.045	302
Fipronil	reference	2018_summer	NB	high pH	5	298	93.9%	0.006	298
Fipronil	reference	2018_summer	NB	high pH	5	255	88.6%	0.017	255
Fipronil	reference	2018_summer	DB	high pH	6	246	93.8%	0.001	246
Fipronil	reference	2018_summer	NB	high pH	5	212	94.2%	0.006	212
Fipronil	reference	2018_summer	DB	high pH	6	194	90.6%	0.003	194
Fipronil	reference	2018_summer	HB	high pH	6	172	93.2%	0.002	172
Fipronil	reference	2018_summer	HB	high pH	6	154	96.7%	0.000	154
Fipronil	reference	2018_summer	HB	high pH	6	145	97.5%	0.000	145
Fipronil	reference	2020_fall	DB	low pH	6	-1155	3.3%	0.729	>316
Fipronil	reference	2020_fall	HB	high pH	5	54.6	65.0%	0.194	>316
Fipronil	reference	2020_fall	HB	high pH	7	107	47.1%	0.132	>316
Fipronil	reference	2020_fall	DB	high pH	7	126	43.7%	0.153	>316
Fipronil	reference	2020_fall	HB	low pH	7	133	16.8%	0.420	>316
Fipronil	reference	2020_fall	DB	high pH	7	178	37.2%	0.199	>316
Fipronil	reference	2020_fall	DB	low pH	4	347	61.4%	0.427	>316
Fipronil	reference	2020_fall	DB	low pH	7	462	7.2%	0.607	>316
Fipronil	reference	2021_fall	DB	low pH	8	564	70.8%	0.009	564
Fipronil	reference	2021_fall	DB	low pH	8	635	35.8%	0.117	>474
Fipronil	reference	2021_fall	HB	low pH	8	366	72.8%	0.007	366
Fipronil	reference	2021_fall	HB	low pH	8	354	69.7%	0.010	354
Fipronil	reference	2021_fall	DB	high pH	8	251	94.4%	0.000	251
Fipronil	reference	2021_fall	DB	high pH	8	236	90.6%	0.000	236
Fipronil	reference	2021_fall	HB	high pH	8	169	97.8%	0.000	169
Fipronil	reference	2021_fall	HB	high pH	8	154	88.6%	0.000	154
Fipronil	reference	2021_summer	HB	low pH	9	596	29.6%	0.130	>474
Fipronil	reference	2021_summer	HB_VH	low pH	9	607	39.4%	0.070	>474
Fipronil	reference	2021_summer	HB_VH	low pH	9	723	38.8%	0.073	>474
Fipronil	reference	2021_summer	HB	low pH	9	2026	4.9%	0.567	>474

Fipronil	reference	2021_summer	HB_VH	high pH	9	203	84.5%	0.000	203
Fipronil	reference	2021_summer	HB	high pH	9	177	93.4%	0.000	177
Fipronil	reference	2021_summer	HB	high pH	9	142	57.7%	0.018	142
Fipronil	reference	2021_summer	HB_VH	high pH	9	128	94.3%	0.000	128
Fipronil	reference	2021_winter	DB	low pH	6	-1733	0.2%	0.929	>316
Fipronil	reference	2021_winter	HB	low pH	5	-408	8.4%	0.636	>316
Fipronil	reference	2021_winter	DB	low pH	6	-217	6.1%	0.638	>316
Fipronil	reference	2021_winter	HB	high pH	5	93.7	76.0%	0.054	>316
Fipronil	reference	2021_winter	HB	low pH	6	165	8.3%	0.581	>316
Fipronil	reference	2021_winter	HB	low pH	6	210	5.0%	0.671	>316
Fipronil	reference	2021_winter	DB	high pH	6	330	22.6%	0.340	>316
Fipronil	reference	2021_winter	DB	low pH	6	385	5.5%	0.654	>316
Fipronil	reference	2021_winter	HB	high pH	5	495	6.8%	0.672	>316
Fipronil	reference	2021_winter	DB	high pH	6	1155	0.9%	0.860	>316
Fipronil	reference	2021_winter	DB	high pH	6	1155	0.4%	0.901	>316
Fipronil	reference	2021_winter	HB	high pH	5	1155	4.9%	0.721	>316
Flonicamid	PPP	2018_summer	DB	high pH	6	360	76.6%	0.022	360
Flonicamid	PPP	2018_summer	DB	high pH	6	358	92.4%	0.002	358
Flonicamid	PPP	2018_summer	NB	high pH	5	322	80.4%	0.039	322
Flonicamid	PPP	2018_summer	DB	high pH	6	426	33.2%	0.231	>316
Flonicamid	PPP	2018_summer	NB	high pH	5	291	94.5%	0.006	291
Flonicamid	PPP	2018_summer	NB	high pH	5	224	95.5%	0.004	224
Flonicamid	PPP	2018_summer	HB	high pH	6	110	98.3%	0.000	110
Flonicamid	PPP	2018_summer	HB	high pH	6	106	98.6%	0.000	106
Flonicamid	PPP	2018_summer	HB	high pH	6	101	97.2%	0.000	101
Florasulam	reference	2018_summer	HB	high pH	6	390	69.8%	0.038	390
Florasulam	reference	2018_summer	DB	high pH	6	-1510	2.7%	0.758	>316
Florasulam	reference	2018_summer	NB	high pH	5	607	48.1%	0.194	>316
Florasulam	reference	2018_summer	DB	high pH	6	719	29.5%	0.266	>316
Florasulam	reference	2018_summer	HB	high pH	6	985	10.2%	0.538	>316
Florasulam	reference	2018_summer	DB	high pH	6	2859	9.1%	0.560	>316
Florasulam	reference	2018_summer	NB	high pH	5	4157	5.7%	0.698	>316
Florasulam	reference	2018_summer	NB	high pH	5	5285	1.4%	0.850	>316
Florasulam	reference	2018_summer	HB	high pH	6	316	73.5%	0.029	>316
Florasulam	reference	2020_fall	DB	high pH	7	-2310	9.7%	0.548	>316
Florasulam	reference	2020_fall	HB	high pH	6	315	66.4%	0.093	>316
Florasulam	reference	2020_fall	DB	low pH	7	433	43.9%	0.152	>316
Florasulam	reference	2020_fall	DB	low pH	7	578	71.4%	0.034	>316
Florasulam	reference	2020_fall	DB	high pH	7	1386	8.6%	0.574	>316
Florasulam	reference	2020_fall	HB	high pH	7	257	64.1%	0.056	257
Florasulam	reference	2020_fall	HB	low pH	7	97.6	93.7%	0.002	98.3
Florasulam	reference	2020_fall	HB	low pH	7	88.9	71.5%	0.034	89.2
Florasulam	reference	2021_fall	DB	high pH	8	-8956	1.0%	0.810	>474
Florasulam	reference	2021_fall	DB	high pH	8	1661	14.7%	0.348	>474
Florasulam	reference	2021_fall	DB	low pH	8	4614	6.2%	0.551	>474
Florasulam	reference	2021_fall	DB	low pH	8	5548	1.6%	0.765	>474
Florasulam	reference	2021_fall	HB	low pH	8	242	95.7%	0.000	242
Florasulam	reference	2021_fall	HB	low pH	8	233	92.4%	0.000	233
Florasulam	reference	2021_fall	HB	high pH	8	196	78.4%	0.003	196
Florasulam	reference	2021_fall	HB	high pH	8	188	84.6%	0.001	188
Florasulam	reference	2021_summer	HB_VH	high pH	9	-2233	20.4%	0.223	>474
Florasulam	reference	2021_summer	HB_VH	low pH	9	2703	20.4%	0.222	>474
Florasulam	reference	2021_summer	HB_VH	high pH	9	4205	5.0%	0.562	>474
Florasulam	reference	2021_summer	HB_VH	low pH	9	6703	1.8%	0.728	>474
Florasulam	reference	2021_summer	HB	high pH	9	207	93.0%	0.000	207
Florasulam	reference	2021_summer	HB	low pH	9	205	96.2%	0.000	205
Florasulam	reference	2021_summer	HB	low pH	9	194	89.0%	0.000	194

Florasulam	reference	2021_summer	HB	high pH	9	158	93.9%	0.000	158
Florasulam	reference	2021_winter	DB	high pH	6	-2310	14.4%	0.459	>316
Florasulam	reference	2021_winter	DB	low pH	6	-330	82.7%	0.012	>316
Florasulam	reference	2021_winter	DB	high pH	6	301	27.2%	0.288	>316
Florasulam	reference	2021_winter	HB	high pH	6	315	44.6%	0.147	>316
Florasulam	reference	2021_winter	DB	high pH	6	1386	2.8%	0.750	>316
Florasulam	reference	2021_winter	HB	high pH	6	315	61.5%	0.065	315
Florasulam	reference	2021_winter	DB	low pH	6	217	71.5%	0.034	218
Florasulam	reference	2021_winter	HB	high pH	6	193	89.4%	0.004	193
Florasulam	reference	2021_winter	DB	low pH	6	193	71.6%	0.034	193
Florasulam	reference	2021_winter	HB	low pH	6	151	95.0%	0.001	149
Florasulam	reference	2021_winter	HB	low pH	6	99.0	96.8%	0.000	99.2
Florasulam	reference	2021_winter	HB	low pH	6	83.5	92.4%	0.002	83.1
Fluopicolide	PPP	2018_summer	HB	high pH	6	587	77.9%	0.020	587
Fluopicolide	PPP	2018_summer	HB	high pH	6	377	76.9%	0.022	377
Fluopicolide	PPP	2018_summer	HB	high pH	6	355	83.2%	0.011	355
Fluopicolide	PPP	2018_summer	DB	high pH	6	-7867	0.5%	0.894	>316
Fluopicolide	PPP	2018_summer	DB	high pH	6	-2499	21.3%	0.357	>316
Fluopicolide	PPP	2018_summer	DB	high pH	6	-523	75.7%	0.024	>316
Fluopicolide	PPP	2018_summer	NB	high pH	5	870	41.3%	0.242	>316
Fluopicolide	PPP	2018_summer	NB	high pH	5	3001	7.0%	0.666	>316
Fluopicolide	PPP	2018_summer	NB	high pH	5	4071	0.9%	0.880	>316
Fluopyram	reference	2018_summer	DB	high pH	6	-2965	44.8%	0.146	>316
Fluopyram	reference	2018_summer	DB	high pH	6	-2676	3.2%	0.736	>316
Fluopyram	reference	2018_summer	DB	high pH	6	-2528	19.5%	0.381	>316
Fluopyram	reference	2018_summer	NB	high pH	5	826	47.2%	0.200	>316
Fluopyram	reference	2018_summer	NB	high pH	5	899	83.7%	0.029	>316
Fluopyram	reference	2018_summer	NB	high pH	5	1740	11.7%	0.574	>316
Fluopyram	reference	2018_summer	HB	high pH	6	184	95.7%	0.001	184
Fluopyram	reference	2018_summer	HB	high pH	6	179	98.8%	0.000	179
Fluopyram	reference	2018_summer	HB	high pH	6	159	97.8%	0.000	159
Fluopyram	reference	2020_fall	HB	high pH	7	495	78.4%	0.019	495
Fluopyram	reference	2020_fall	HB	high pH	6	462	99.1%	0.000	462
Fluopyram	reference	2020_fall	HB	low pH	7	433	89.9%	0.004	433
Fluopyram	reference	2020_fall	HB	low pH	7	277	38.9%	0.186	>316
Fluopyram	reference	2020_fall	DB	low pH	7	990	32.4%	0.238	>316
Fluopyram	reference	2020_fall	DB	high pH	7	1386	83.6%	0.011	>316
Fluopyram	reference	2020_fall	DB	low pH	7	1733	12.8%	0.486	>316
Fluopyram	reference	2020_fall	DB	high pH	7	3466	5.4%	0.657	>316
Fluopyram	reference	2021_fall	HB	low pH	8	1151	86.0%	0.001	>474
Fluopyram	reference	2021_fall	DB	low pH	8	1331	15.1%	0.341	>474
Fluopyram	reference	2021_fall	HB	low pH	8	1411	61.0%	0.022	>474
Fluopyram	reference	2021_fall	DB	high pH	8	1828	68.3%	0.011	>474
Fluopyram	reference	2021_fall	DB	high pH	8	11871	2.3%	0.720	>474
Fluopyram	reference	2021_fall	DB	low pH	8	21340	0.8%	0.839	>474
Fluopyram	reference	2021_fall	HB	high pH	8	371	94.1%	0.000	371
Fluopyram	reference	2021_fall	HB	high pH	8	318	96.4%	0.000	318
Fluopyram	reference	2021_summer	HB_VH	high pH	9	597	87.0%	0.000	597
Fluopyram	reference	2021_summer	HB_VH	high pH	9	570	73.6%	0.003	570
Fluopyram	reference	2021_summer	HB	low pH	9	2470	11.3%	0.376	>474
Fluopyram	reference	2021_summer	HB_VH	low pH	9	2628	26.3%	0.158	>474
Fluopyram	reference	2021_summer	HB	low pH	9	2943	4.0%	0.605	>474
Fluopyram	reference	2021_summer	HB_VH	low pH	9	3821	5.9%	0.528	>474
Fluopyram	reference	2021_summer	HB	high pH	9	269	93.4%	0.000	269
Fluopyram	reference	2021_summer	HB	high pH	9	267	94.1%	0.000	267
Fluopyram	reference	2021_winter	HB	high pH	6	408	80.3%	0.016	408
Fluopyram	reference	2021_winter	HB	low pH	6	385	96.2%	0.001	385

Fluopyram	reference	2021_winter	HB	low pH	6	347	74.0%	0.028	348
Fluopyram	reference	2021_winter	DB	low pH	6	-365	71.4%	0.034	>316
Fluopyram	reference	2021_winter	DB	low pH	6	385	42.9%	0.158	>316
Fluopyram	reference	2021_winter	DB	low pH	6	770	86.9%	0.007	>316
Fluopyram	reference	2021_winter	DB	high pH	6	990	27.7%	0.283	>316
Fluopyram	reference	2021_winter	DB	high pH	6	1733	25.0%	0.312	>316
Fluopyram	reference	2021_winter	HB	high pH	6	1733	92.3%	0.002	>316
Fluopyram	reference	2021_winter	DB	high pH	6	2310	6.5%	0.627	>316
Fluopyram	reference	2021_winter	HB	high pH	6	2310	11.5%	0.510	>316
Fluopyram	reference	2021_winter	HB	low pH	6	277	97.3%	0.000	277
Flupyradifurone	reference	2018_summer	NB	high pH	5	493	87.7%	0.019	>316
Flupyradifurone	reference	2018_summer	DB	high pH	6	1005	40.7%	0.173	>316
Flupyradifurone	reference	2018_summer	DB	high pH	6	1512	51.8%	0.107	>316
Flupyradifurone	reference	2018_summer	NB	high pH	5	2425	9.3%	0.618	>316
Flupyradifurone	reference	2018_summer	NB	high pH	5	4466	2.8%	0.787	>316
Flupyradifurone	reference	2018_summer	DB	high pH	6	7965	0.4%	0.911	>316
Flupyradifurone	reference	2018_summer	HB	high pH	6	119	97.0%	0.000	119
Flupyradifurone	reference	2018_summer	HB	high pH	6	111	94.2%	0.001	111
Flupyradifurone	reference	2018_summer	HB	high pH	6	100	97.8%	0.000	100
Flupyradifurone	reference	2020_fall	HB	high pH	7	315	83.8%	0.010	319
Flupyradifurone	reference	2020_fall	DB	low pH	7	-3466	2.6%	0.759	>316
Flupyradifurone	reference	2020_fall	DB	high pH	7	-2310	7.4%	0.602	>316
Flupyradifurone	reference	2020_fall	DB	high pH	7	-578	45.0%	0.145	>316
Flupyradifurone	reference	2020_fall	DB	low pH	6	128	66.8%	0.091	>316
Flupyradifurone	reference	2020_fall	HB	high pH	6	462	93.7%	0.007	>316
Flupyradifurone	reference	2020_fall	DB	low pH	7	6931	0.2%	0.932	>316
Flupyradifurone	reference	2020_fall	HB	low pH	7	248	87.3%	0.006	245
Flupyradifurone	reference	2021_fall	HB	low pH	8	-3210	43.5%	0.075	>474
Flupyradifurone	reference	2021_fall	HB	low pH	8	-1570	21.5%	0.247	>474
Flupyradifurone	reference	2021_fall	DB	low pH	8	-1553	13.6%	0.369	>474
Flupyradifurone	reference	2021_fall	DB	low pH	8	-1061	83.0%	0.002	>474
Flupyradifurone	reference	2021_fall	DB	high pH	8	1888	33.2%	0.135	>474
Flupyradifurone	reference	2021_fall	DB	high pH	8	4816	12.2%	0.396	>474
Flupyradifurone	reference	2021_fall	HB	high pH	8	264	94.6%	0.000	264
Flupyradifurone	reference	2021_fall	HB	high pH	8	231	93.6%	0.000	231
Flupyradifurone	reference	2021_summer	HB	low pH	9	-36161	0.1%	0.932	>474
Flupyradifurone	reference	2021_summer	HB_VH	low pH	9	-14392	0.5%	0.852	>474
Flupyradifurone	reference	2021_summer	HB	low pH	9	-8252	0.6%	0.841	>474
Flupyradifurone	reference	2021_summer	HB_VH	low pH	9	18818	1.3%	0.767	>474
Flupyradifurone	reference	2021_summer	HB_VH	high pH	9	335	96.6%	0.000	335
Flupyradifurone	reference	2021_summer	HB_VH	high pH	9	258	95.6%	0.000	258
Flupyradifurone	reference	2021_summer	HB	high pH	9	213	93.1%	0.000	213
Flupyradifurone	reference	2021_summer	HB	high pH	9	212	90.5%	0.000	212
Flupyradifurone	reference	2021_winter	DB	low pH	6	-693	37.6%	0.196	>316
Flupyradifurone	reference	2021_winter	DB	high pH	6	88.9	49.8%	0.117	>316
Flupyradifurone	reference	2021_winter	HB	high pH	6	231	3.8%	0.712	>316
Flupyradifurone	reference	2021_winter	DB	high pH	6	315	1.8%	0.800	>316
Flupyradifurone	reference	2021_winter	DB	high pH	6	408	5.9%	0.644	>316
Flupyradifurone	reference	2021_winter	HB	high pH	6	433	3.7%	0.716	>316
Flupyradifurone	reference	2021_winter	HB	high pH	6	1386	49.2%	0.120	>316
Flupyradifurone	reference	2021_winter	HB	low pH	6	1386	33.8%	0.226	>316
Flupyradifurone	reference	2021_winter	DB	low pH	6	97.6	66.1%	0.049	97.3
Flupyradifurone	reference	2021_winter	HB	low pH	6	92.4	70.8%	0.036	92.0
Flupyradifurone	reference	2021_winter	HB	low pH	6	86.6	72.1%	0.032	86.7
Flupyradifurone	reference	2021_winter	DB	low pH	6	84.5	71.2%	0.035	84.4
Flutianil	PPP	2018_summer	HB	high pH	5	446	26.7%	0.372	>316
Flutianil	PPP	2018_summer	NB	high pH	8	659	38.7%	0.136	>316

Flutianil	PPP	2018_summer	NB	high pH	8	821	25.8%	0.245	>316
Flutianil	PPP	2018_summer	HB	high pH	3	2863	0.8%	0.944	>316
Flutianil	PPP	2018_summer	DB	high pH	8	230	67.6%	0.012	230
Flutianil	PPP	2018_summer	DB	high pH	8	152	78.5%	0.003	152
Flutianil	PPP	2018_summer	DB	high pH	8	147	74.7%	0.006	147
Fluxapyroxad	PPP	2018_summer	NB	high pH	5	523	79.4%	0.043	523
Fluxapyroxad	PPP	2018_summer	NB	high pH	5	365	68.5%	0.084	365
Fluxapyroxad	PPP	2018_summer	DB	high pH	6	-1193	9.0%	0.563	>316
Fluxapyroxad	PPP	2018_summer	NB	high pH	5	674	45.1%	0.214	>316
Fluxapyroxad	PPP	2018_summer	DB	high pH	6	716	59.3%	0.073	>316
Fluxapyroxad	PPP	2018_summer	DB	high pH	6	2077	3.6%	0.718	>316
Fluxapyroxad	PPP	2018_summer	HB	high pH	6	209	78.4%	0.019	209
Fluxapyroxad	PPP	2018_summer	HB	high pH	6	198	65.8%	0.050	198
Fluxapyroxad	PPP	2018_summer	HB	high pH	6	193	95.9%	0.001	193
Hydrochlorothiazide	API	2021_fall	DB	high pH	8	584	76.7%	0.004	584
Hydrochlorothiazide	API	2021_fall	DB	high pH	8	482	85.0%	0.001	482
Hydrochlorothiazide	API	2021_fall	DB	low pH	8	-31356	0.4%	0.880	>474
Hydrochlorothiazide	API	2021_fall	HB	low pH	8	-8274	1.1%	0.809	>474
Hydrochlorothiazide	API	2021_fall	HB	low pH	8	1643	75.4%	0.005	>474
Hydrochlorothiazide	API	2021_fall	DB	low pH	8	8291	0.5%	0.870	>474
Hydrochlorothiazide	API	2021_fall	HB	high pH	8	406	85.4%	0.001	406
Hydrochlorothiazide	API	2021_fall	HB	high pH	8	378	74.3%	0.006	378
Imidacloprid	reference	2018_summer	DB	high pH	6	-1135	26.7%	0.294	>316
Imidacloprid	reference	2018_summer	DB	high pH	6	1425	22.1%	0.347	>316
Imidacloprid	reference	2018_summer	NB	high pH	5	5166	6.5%	0.679	>316
Imidacloprid	reference	2018_summer	DB	high pH	6	9126	1.7%	0.805	>316
Imidacloprid	reference	2018_summer	NB	high pH	5	14639	0.1%	0.962	>316
Imidacloprid	reference	2018_summer	NB	high pH	5	60079	0.0%	0.975	>316
Imidacloprid	reference	2018_summer	HB	high pH	6	260	91.6%	0.003	260
Imidacloprid	reference	2018_summer	HB	high pH	6	212	96.0%	0.001	212
Imidacloprid	reference	2018_summer	HB	high pH	6	203	96.0%	0.001	203
Imidacloprid	reference	2020_fall	HB	low pH	7	385	87.0%	0.007	377
Imidacloprid	reference	2020_fall	DB	high pH	7	-6931	1.8%	0.800	>316
Imidacloprid	reference	2020_fall	DB	low pH	7	-3466	6.4%	0.627	>316
Imidacloprid	reference	2020_fall	DB	low pH	7	1733	15.5%	0.440	>316
Imidacloprid	reference	2020_fall	DB	high pH	7	3466	15.0%	0.449	>316
Imidacloprid	reference	2020_fall	HB	low pH	7	217	53.0%	0.101	219
Imidacloprid	reference	2020_fall	HB	high pH	6	144	94.4%	0.006	145
Imidacloprid	reference	2020_fall	HB	high pH	7	117	96.1%	0.001	117
Imidacloprid	reference	2021_fall	HB	high pH	9	565	62.4%	0.011	565
Imidacloprid	reference	2021_fall	HB	low pH	9	-7853	0.5%	0.860	>474
Imidacloprid	reference	2021_fall	DB	low pH	9	-4356	0.8%	0.817	>474
Imidacloprid	reference	2021_fall	DB	high pH	9	-4108	3.7%	0.620	>474
Imidacloprid	reference	2021_fall	DB	high pH	9	-3298	2.0%	0.715	>474
Imidacloprid	reference	2021_fall	DB	low pH	9	-999	45.1%	0.048	>474
Imidacloprid	reference	2021_fall	HB	high pH	9	513	41.0%	0.063	>474
Imidacloprid	reference	2021_fall	HB	low pH	9	23143	0.5%	0.859	>474
Imidacloprid	reference	2021_summer	HB	low pH	9	1126	49.8%	0.034	>474
Imidacloprid	reference	2021_summer	HB	low pH	9	1471	19.9%	0.229	>474
Imidacloprid	reference	2021_summer	HB_VH	low pH	9	2235	18.9%	0.242	>474
Imidacloprid	reference	2021_summer	HB_VH	low pH	9	2269	44.2%	0.051	>474
Imidacloprid	reference	2021_summer	HB	high pH	9	264	94.8%	0.000	264
Imidacloprid	reference	2021_summer	HB	high pH	9	253	92.7%	0.000	253
Imidacloprid	reference	2021_summer	HB_VH	high pH	9	196	98.2%	0.000	196
Imidacloprid	reference	2021_summer	HB_VH	high pH	9	143	96.5%	0.000	143
Imidacloprid	reference	2021_winter	HB	low pH	6	408	92.0%	0.002	408
Imidacloprid	reference	2021_winter	HB	low pH	6	347	95.0%	0.001	341

Imidacloprid	reference	2021_winter	DB	low pH	6	-866	49.5%	0.119	>316
Imidacloprid	reference	2021_winter	DB	low pH	6	408	38.9%	0.186	>316
Imidacloprid	reference	2021_winter	DB	high pH	6	866	8.5%	0.576	>316
Imidacloprid	reference	2021_winter	DB	high pH	6	1155	26.0%	0.302	>316
Imidacloprid	reference	2021_winter	DB	low pH	6	1733	47.1%	0.132	>316
Imidacloprid	reference	2021_winter	DB	high pH	6	2310	12.3%	0.496	>316
Imidacloprid	reference	2021_winter	HB	low pH	6	301	87.8%	0.006	295
Imidacloprid	reference	2021_winter	HB	high pH	6	169	79.5%	0.017	170
Imidacloprid	reference	2021_winter	HB	high pH	6	147	97.0%	0.000	149
Imidacloprid	reference	2021_winter	HB	high pH	6	126	98.6%	0.000	127
Iprovalicarb	PPP	2018_summer	HB	high pH	3	4.30	96.4%	0.121	<1.2
Iprovalicarb	PPP	2018_summer	HB	high pH	3	4.40	94.6%	0.149	<1.2
Iprovalicarb	PPP	2018_summer	HB	high pH	4	30.6	41.0%	0.360	<1.2
Iprovalicarb	PPP	2018_summer	DB	high pH	6	125	93.7%	0.002	125
Iprovalicarb	PPP	2018_summer	DB	high pH	6	116	96.8%	0.000	116
Iprovalicarb	PPP	2018_summer	DB	high pH	6	97.9	96.4%	0.000	97.9
Iprovalicarb	PPP	2018_summer	NB	high pH	5	20.3	98.1%	0.001	20.3
Iprovalicarb	PPP	2018_summer	NB	high pH	5	19.9	98.1%	0.001	19.9
Iprovalicarb	PPP	2018_summer	NB	high pH	5	19.7	97.9%	0.001	19.7
Irbesartan	API	2021_fall	DB	low pH	8	528	77.6%	0.004	528
Irbesartan	API	2021_fall	DB	low pH	8	594	44.8%	0.070	>474
Irbesartan	API	2021_fall	DB	high pH	8	185	95.6%	0.000	185
Irbesartan	API	2021_fall	DB	high pH	8	184	96.4%	0.000	184
Irbesartan	API	2021_fall	HB	low pH	8	149	61.9%	0.021	149
Irbesartan	API	2021_fall	HB	low pH	8	123	68.1%	0.012	123
Irbesartan	API	2021_fall	HB	high pH	8	55.4	97.6%	0.000	55.4
Irbesartan	API	2021_fall	HB	high pH	8	49.9	97.0%	0.000	49.9
Isofetamid	PPP	2018_summer	DB	high pH	8	379	54.9%	0.035	379
Isofetamid	PPP	2018_summer	DB	high pH	8	469	40.4%	0.090	>316
Isofetamid	PPP	2018_summer	DB	high pH	8	246	84.5%	0.001	246
Isofetamid	PPP	2018_summer	NB	high pH	8	68.2	96.1%	0.000	68.2
Isofetamid	PPP	2018_summer	NB	high pH	8	61.8	98.8%	0.000	61.8
Isofetamid	PPP	2018_summer	HB	high pH	8	25.3	97.6%	0.000	25.3
Isofetamid	PPP	2018_summer	HB	high pH	8	25.0	96.9%	0.000	25.0
Isoproturon	reference	2018_summer	DB	high pH	6	900	77.4%	0.021	>316
Isoproturon	reference	2018_summer	DB	high pH	6	996	83.0%	0.011	>316
Isoproturon	reference	2018_summer	DB	high pH	6	1159	40.5%	0.175	>316
Isoproturon	reference	2018_summer	NB	high pH	5	130	99.6%	0.000	130
Isoproturon	reference	2018_summer	NB	high pH	5	129	99.6%	0.000	129
Isoproturon	reference	2018_summer	NB	high pH	5	129	97.8%	0.001	129
Isoproturon	reference	2018_summer	HB	high pH	6	25.1	100.0%	0.000	25.1
Isoproturon	reference	2018_summer	HB	high pH	6	23.9	99.9%	0.000	23.9
Isoproturon	reference	2018_summer	HB	high pH	6	23.3	99.9%	0.000	23.3
Isoproturon	reference	2020_fall	DB	high pH	7	330	83.8%	0.010	332
Isoproturon	reference	2020_fall	DB	low pH	7	289	83.5%	0.011	286
Isoproturon	reference	2020_fall	DB	low pH	7	277	86.7%	0.007	277
Isoproturon	reference	2020_fall	HB	high pH	6	248	98.6%	0.001	252
Isoproturon	reference	2020_fall	DB	high pH	7	224	98.1%	0.000	223
Isoproturon	reference	2020_fall	HB	high pH	7	193	93.0%	0.002	194
Isoproturon	reference	2020_fall	HB	low pH	7	22.5	99.0%	0.000	22.5
Isoproturon	reference	2020_fall	DB	low pH	6	21.2	97.4%	0.002	21.2
Isoproturon	reference	2021_fall	DB	low pH	8	720	42.9%	0.078	>474
Isoproturon	reference	2021_fall	DB	high pH	8	721	86.3%	0.001	>474
Isoproturon	reference	2021_fall	DB	high pH	8	732	84.6%	0.001	>474
Isoproturon	reference	2021_fall	DB	low pH	8	3847	31.8%	0.145	>474
Isoproturon	reference	2021_fall	HB	low pH	8	139	87.4%	0.001	139
Isoproturon	reference	2021_fall	HB	low pH	8	118	95.4%	0.000	118

Isoproturon	reference	2021_fall	HB	high pH	8	40.9	99.8%	0.000	40.9
Isoproturon	reference	2021_fall	HB	high pH	8	37.8	99.9%	0.000	37.8
Isoproturon	reference	2021_summer	HB_VH	low pH	9	94.0	92.6%	0.000	94.0
Isoproturon	reference	2021_summer	HB_VH	low pH	9	86.3	95.0%	0.000	86.3
Isoproturon	reference	2021_summer	HB	low pH	9	58.2	98.2%	0.000	58.2
Isoproturon	reference	2021_summer	HB	low pH	9	56.8	97.8%	0.000	56.8
Isoproturon	reference	2021_summer	HB_VH	high pH	9	38.4	99.6%	0.000	38.4
Isoproturon	reference	2021_summer	HB_VH	high pH	8	26.9	99.8%	0.000	26.9
Isoproturon	reference	2021_summer	HB	high pH	7	22.5	98.4%	0.000	22.5
Isoproturon	reference	2021_summer	HB	high pH	7	21.7	98.4%	0.000	21.7
Isoproturon	reference	2021_winter	DB	low pH	6	330	95.7%	0.001	331
Isoproturon	reference	2021_winter	DB	high pH	6	330	95.7%	0.001	327
Isoproturon	reference	2021_winter	DB	high pH	6	330	84.0%	0.010	323
Isoproturon	reference	2021_winter	DB	low pH	6	-6931	2.5%	0.765	>316
Isoproturon	reference	2021_winter	HB	high pH	6	267	70.4%	0.037	270
Isoproturon	reference	2021_winter	DB	low pH	6	239	67.4%	0.045	241
Isoproturon	reference	2021_winter	HB	high pH	6	224	96.3%	0.001	222
Isoproturon	reference	2021_winter	HB	high pH	6	182	99.2%	0.000	185
Isoproturon	reference	2021_winter	DB	high pH	6	116	74.1%	0.028	115
Isoproturon	reference	2021_winter	HB	low pH	6	55.9	87.5%	0.006	56.0
Isoproturon	reference	2021_winter	HB	low pH	6	51.7	94.2%	0.001	51.8
Isoproturon	reference	2021_winter	HB	low pH	6	51.3	91.8%	0.003	51.5
Isopyrazam	PPP	2018_summer	DB	high pH	8	806	15.6%	0.333	>316
Isopyrazam	PPP	2018_summer	DB	high pH	8	294	88.6%	0.000	294
Isopyrazam	PPP	2018_summer	NB	high pH	8	271	91.8%	0.001	271
Isopyrazam	PPP	2018_summer	DB	high pH	8	262	63.6%	0.018	262
Isopyrazam	PPP	2018_summer	NB	high pH	8	199	76.6%	0.010	199
Isopyrazam	PPP	2018_summer	HB	high pH	8	138	95.5%	0.000	138
Isopyrazam	PPP	2018_summer	HB	high pH	8	138	90.3%	0.000	138
Keto-desogestrel	API	2021_fall	HB	high pH	2	NA	NA	NA	<1.2
Keto-desogestrel	API	2021_fall	HB	high pH	2	NA	NA	NA	<1.2
Keto-desogestrel	API	2021_fall	HB	low pH	2	NA	NA	NA	<1.2
Keto-desogestrel	API	2021_fall	HB	low pH	2	NA	NA	NA	<1.2
Keto-desogestrel	API	2021_fall	DB	low pH	4	3.14	99.0%	0.005	3.14
Keto-desogestrel	API	2021_fall	DB	low pH	4	2.09	98.3%	0.009	2.09
Keto-desogestrel	API	2021_fall	DB	high pH	3	1.52	99.2%	0.056	1.52
Keto-desogestrel	API	2021_fall	DB	high pH	3	1.48	98.6%	0.075	1.48
Kresoxim-methyl	reference	2018_summer	HB	high pH	2	11.2	100.0%	NA	<1.2
Kresoxim-methyl	reference	2018_summer	HB	high pH	3	55.5	53.0%	0.481	<1.2
Kresoxim-methyl	reference	2018_summer	HB	high pH	1	NA	NA	NA	<1.2
Kresoxim-methyl	reference	2018_summer	NB	high pH	1	NA	NA	NA	<1.2
Kresoxim-methyl	reference	2018_summer	NB	high pH	1	NA	NA	NA	<1.2
Kresoxim-methyl	reference	2018_summer	NB	high pH	1	NA	NA	NA	<1.2
Kresoxim-methyl	reference	2018_summer	DB	high pH	3	7.18	99.7%	0.032	7.18
Kresoxim-methyl	reference	2018_summer	DB	high pH	3	6.47	99.9%	0.016	6.47
Kresoxim-methyl	reference	2018_summer	DB	high pH	3	6.46	99.8%	0.031	6.46
Kresoxim-methyl	reference	2021_fall	HB	high pH	2	NA	NA	NA	<1.2
Kresoxim-methyl	reference	2021_fall	HB	high pH	2	NA	NA	NA	<1.2
Kresoxim-methyl	reference	2021_fall	DB	low pH	9	74.3	95.1%	0.000	74.3
Kresoxim-methyl	reference	2021_fall	DB	low pH	9	59.2	93.2%	0.000	59.2
Kresoxim-methyl	reference	2021_fall	DB	high pH	3	3.42	99.0%	0.065	3.42
Kresoxim-methyl	reference	2021_fall	HB	low pH	3	2.94	99.5%	0.044	2.94
Kresoxim-methyl	reference	2021_fall	HB	low pH	4	2.56	99.5%	0.002	2.56
Kresoxim-methyl	reference	2021_fall	DB	high pH	4	1.71	93.9%	0.031	1.71
Lumiracoxib	API	2021_fall	DB	low pH	8	578	53.5%	0.039	578
Lumiracoxib	API	2021_fall	DB	high pH	8	-2303	5.1%	0.591	>474
Lumiracoxib	API	2021_fall	DB	high pH	8	5829	3.2%	0.673	>474

Lumiracoxib	API	2021_fall	DB	low pH	8	349	70.1%	0.009	349
Lumiracoxib	API	2021_fall	HB	low pH	8	87.6	88.9%	0.000	87.6
Lumiracoxib	API	2021_fall	HB	low pH	8	56.2	97.8%	0.000	56.2
Lumiracoxib	API	2021_fall	HB	high pH	8	35.2	95.0%	0.000	35.2
Lumiracoxib	API	2021_fall	HB	high pH	8	35.1	99.0%	0.000	35.1
Mandipropamid	PPP	2018_summer	HB	high pH	6	NA	NA	NA	<1.2
Mandipropamid	PPP	2018_summer	HB	high pH	6	NA	NA	NA	<1.2
Mandipropamid	PPP	2018_summer	HB	high pH	6	NA	NA	NA	<1.2
Mandipropamid	reference	2018_summer	DB	high pH	6	51.4	98.4%	0.000	51.4
Mandipropamid	reference	2018_summer	DB	high pH	6	50.1	98.9%	0.000	50.1
Mandipropamid	reference	2018_summer	DB	high pH	6	48.3	99.1%	0.000	48.3
Mandipropamid	reference	2018_summer	NB	high pH	2	9.76	100.0%	NA	9.76
Mandipropamid	reference	2018_summer	NB	high pH	2	8.97	100.0%	NA	8.97
Mandipropamid	reference	2018_summer	NB	high pH	2	8.53	100.0%	NA	8.53
Mandipropamid	reference	2020_fall	HB	low pH	4	3.96	94.5%	0.151	<1.2
Mandipropamid	reference	2020_fall	HB	low pH	3	7.04	100.0%	NA	<1.2
Mandipropamid	reference	2020_fall	DB	low pH	7	55.0	96.7%	0.000	54.8
Mandipropamid	reference	2020_fall	DB	low pH	7	47.5	99.4%	0.000	47.6
Mandipropamid	reference	2020_fall	DB	high pH	7	42.5	96.0%	0.001	42.6
Mandipropamid	reference	2020_fall	DB	high pH	7	38.1	95.5%	0.001	38.1
Mandipropamid	reference	2020_fall	HB	high pH	4	13.7	100.0%	0.002	13.7
Mandipropamid	reference	2020_fall	HB	high pH	4	10.8	99.8%	0.026	10.8
Mandipropamid	reference	2021_fall	HB	high pH	3	2.91	99.0%	0.065	<1.2
Mandipropamid	reference	2021_fall	HB	high pH	3	2.95	92.7%	0.174	<1.2
Mandipropamid	reference	2021_fall	DB	low pH	8	156	93.4%	0.000	156
Mandipropamid	reference	2021_fall	DB	low pH	8	130	92.5%	0.000	130
Mandipropamid	reference	2021_fall	DB	high pH	8	26.0	97.0%	0.000	26.0
Mandipropamid	reference	2021_fall	DB	high pH	8	20.4	90.3%	0.000	20.4
Mandipropamid	reference	2021_fall	HB	low pH	4	3.55	95.9%	0.021	3.55
Mandipropamid	reference	2021_fall	HB	low pH	4	3.27	92.2%	0.040	3.27
Mandipropamid	reference	2021_summer	HB_VH	low pH	4	11.3	98.9%	0.006	11.3
Mandipropamid	reference	2021_summer	HB_VH	low pH	4	10.5	99.8%	0.001	10.5
Mandipropamid	reference	2021_summer	HB	low pH	3	6.35	98.5%	0.079	6.35
Mandipropamid	reference	2021_summer	HB	low pH	3	5.95	99.7%	0.036	5.95
Mandipropamid	reference	2021_summer	HB_VH	high pH	4	5.76	99.5%	0.003	5.76
Mandipropamid	reference	2021_summer	HB_VH	high pH	4	5.36	98.2%	0.009	5.36
Mandipropamid	reference	2021_summer	HB	high pH	3	2.92	98.8%	0.070	2.92
Mandipropamid	reference	2021_summer	HB	high pH	3	2.53	99.5%	0.047	2.53
Mandipropamid	reference	2021_winter	HB	high pH	3	18.8	76.2%	0.325	NA
Mandipropamid	reference	2021_winter	DB	low pH	6	102	95.2%	0.001	102
Mandipropamid	reference	2021_winter	DB	low pH	6	64.8	96.6%	0.000	65.0
Mandipropamid	reference	2021_winter	DB	low pH	6	62.5	92.2%	0.002	62.7
Mandipropamid	reference	2021_winter	DB	high pH	6	29.8	99.2%	0.000	29.8
Mandipropamid	reference	2021_winter	DB	high pH	6	29.5	99.4%	0.000	29.4
Mandipropamid	reference	2021_winter	DB	high pH	6	28.4	99.4%	0.000	28.4
Mandipropamid	reference	2021_winter	HB	high pH	4	12.4	98.2%	0.009	12.4
Mandipropamid	reference	2021_winter	HB	high pH	4	9.93	97.8%	0.011	9.90
Mandipropamid	reference	2021_winter	HB	low pH	2	4.20	100.0%	NA	4.20
Mandipropamid	reference	2021_winter	HB	low pH	2	3.86	100.0%	NA	3.90
Mandipropamid	reference	2021_winter	HB	low pH	2	3.78	100.0%	NA	3.80
Mesosulfuron-methyl	PPP	2018_summer	HB	high pH	6	573	62.9%	0.060	573
Mesosulfuron-methyl	PPP	2018_summer	HB	high pH	6	327	38.1%	0.191	327
Mesosulfuron-methyl	PPP	2018_summer	DB	high pH	6	-2782	2.9%	0.748	>316

Mesosulfuron-methyl	PPP	2018_summer	NB	high pH	5	-1620	38.2%	0.266	>316
Mesosulfuron-methyl	PPP	2018_summer	NB	high pH	5	-1489	24.0%	0.402	>316
Mesosulfuron-methyl	PPP	2018_summer	DB	high pH	6	-1104	10.9%	0.524	>316
Mesosulfuron-methyl	PPP	2018_summer	NB	high pH	5	976	27.3%	0.367	>316
Mesosulfuron-methyl	PPP	2018_summer	DB	high pH	6	1921	8.3%	0.581	>316
Mesosulfuron-methyl	PPP	2018_summer	HB	high pH	6	205	90.3%	0.004	205
Mesotrione	reference	2018_summer	NB	high pH	5	369	55.6%	0.148	369
Mesotrione	reference	2018_summer	NB	high pH	5	-80698	0.0%	0.996	>316
Mesotrione	reference	2018_summer	DB	high pH	6	-615	39.6%	0.180	>316
Mesotrione	reference	2018_summer	DB	high pH	6	846	18.5%	0.395	>316
Mesotrione	reference	2018_summer	NB	high pH	5	1295	14.0%	0.536	>316
Mesotrione	reference	2018_summer	DB	high pH	6	1697	14.1%	0.463	>316
Mesotrione	reference	2018_summer	HB	high pH	6	74.6	97.3%	0.000	74.6
Mesotrione	reference	2018_summer	HB	high pH	6	63.6	99.6%	0.000	63.6
Mesotrione	reference	2018_summer	HB	high pH	6	59.0	97.1%	0.000	59.0
Mesotrione	reference	2020_fall	DB	high pH	7	-990	29.7%	0.263	>316
Mesotrione	reference	2020_fall	DB	low pH	7	578	69.9%	0.038	>316
Mesotrione	reference	2020_fall	DB	low pH	7	578	50.6%	0.113	>316
Mesotrione	reference	2020_fall	DB	high pH	7	6931	2.9%	0.746	>316
Mesotrione	reference	2020_fall	HB	high pH	7	70.0	98.6%	0.000	70.0
Mesotrione	reference	2020_fall	HB	high pH	6	41.3	96.9%	0.002	41.2
Mesotrione	reference	2020_fall	HB	low pH	7	37.3	99.9%	0.000	37.2
Mesotrione	reference	2020_fall	HB	low pH	7	35.6	97.9%	0.000	35.6
Mesotrione	reference	2021_fall	DB	high pH	9	-2189	5.4%	0.549	>474
Mesotrione	reference	2021_fall	DB	high pH	9	-916	9.1%	0.429	>474
Mesotrione	reference	2021_fall	DB	low pH	9	422	67.4%	0.007	422
Mesotrione	reference	2021_fall	DB	low pH	9	249	68.2%	0.006	249
Mesotrione	reference	2021_fall	HB	low pH	9	34.4	85.2%	0.000	34.4
Mesotrione	reference	2021_fall	HB	low pH	9	32.9	92.3%	0.000	32.9
Mesotrione	reference	2021_fall	HB	high pH	9	30.7	92.2%	0.000	30.7
Mesotrione	reference	2021_fall	HB	high pH	9	29.5	93.9%	0.000	29.5
Mesotrione	reference	2021_summer	HB_VH	high pH	9	215	95.2%	0.000	215
Mesotrione	reference	2021_summer	HB_VH	high pH	9	168	97.2%	0.000	168
Mesotrione	reference	2021_summer	HB_VH	low pH	9	136	97.3%	0.000	136
Mesotrione	reference	2021_summer	HB_VH	low pH	9	129	95.2%	0.000	129
Mesotrione	reference	2021_summer	HB	low pH	9	53.7	96.8%	0.000	53.7
Mesotrione	reference	2021_summer	HB	low pH	9	53.2	95.7%	0.000	53.2
Mesotrione	reference	2021_summer	HB	high pH	9	31.9	97.2%	0.000	31.9
Mesotrione	reference	2021_summer	HB	high pH	9	27.8	98.5%	0.000	27.8
Mesotrione	reference	2021_winter	DB	low pH	6	330	96.8%	0.000	329
Mesotrione	reference	2021_winter	DB	high pH	6	-6931	0.3%	0.914	>316
Mesotrione	reference	2021_winter	DB	high pH	6	-2310	6.0%	0.640	>316
Mesotrione	reference	2021_winter	DB	high pH	6	6931	0.3%	0.914	>316
Mesotrione	reference	2021_winter	DB	low pH	6	NA	0.1%	0.952	>316
Mesotrione	reference	2021_winter	DB	low pH	6	289	69.4%	0.039	290
Mesotrione	reference	2021_winter	HB	high pH	6	70.0	84.3%	0.010	70.3
Mesotrione	reference	2021_winter	HB	high pH	6	54.2	98.0%	0.000	54.1
Mesotrione	reference	2021_winter	HB	high pH	6	51.3	95.0%	0.001	51.4
Mesotrione	reference	2021_winter	HB	low pH	6	38.9	94.8%	0.001	38.9
Mesotrione	reference	2021_winter	HB	low pH	6	38.7	90.6%	0.003	38.7
Mesotrione	reference	2021_winter	HB	low pH	6	35.4	97.6%	0.000	35.4

Metformin	API	2021_fall	HB	high pH	3	3.28	73.3%	0.346	<1.2
Metformin	API	2021_fall	HB	high pH	3	3.49	73.5%	0.344	<1.2
Metformin	API	2021_fall	HB	low pH	4	7.04	79.6%	0.108	<1.2
Metformin	API	2021_fall	HB	low pH	4	7.14	79.5%	0.108	<1.2
Metformin	API	2021_fall	DB	low pH	6	45.1	93.7%	0.002	45.1
Metformin	API	2021_fall	DB	low pH	6	41.8	96.9%	0.000	41.8
Metformin	API	2021_fall	DB	high pH	5	17.5	87.1%	0.021	17.5
Metformin	API	2021_fall	DB	high pH	5	16.6	84.3%	0.028	16.6
Mirtazapine	API	2021_fall	HB	high pH	9	-120	28.0%	0.143	>474
Mirtazapine	API	2021_fall	HB	high pH	9	-104	41.8%	0.060	>474
Mirtazapine	API	2021_fall	DB	high pH	9	279	35.4%	0.091	>474
Mirtazapine	API	2021_fall	HB	low pH	9	1070	39.1%	0.072	>474
Mirtazapine	API	2021_fall	DB	low pH	9	1296	12.6%	0.348	>474
Mirtazapine	API	2021_fall	DB	low pH	9	1914	19.5%	0.234	>474
Mirtazapine	API	2021_fall	HB	low pH	9	4627	6.0%	0.527	>474
Mirtazapine	API	2021_fall	DB	high pH	9	304	58.9%	0.016	304
Mometasone	API	2021_fall	HB	high pH	8	693	1.0%	0.811	NA
Mometasone	API	2021_fall	HB	low pH	8	1804	1.2%	0.800	NA
Mometasone	API	2021_fall	DB	low pH	8	312	63.5%	0.018	312
Mometasone	API	2021_fall	DB	low pH	8	226	69.0%	0.011	226
Mometasone	API	2021_fall	HB	low pH	8	85.3	65.9%	0.014	85.3
Mometasone	API	2021_fall	HB	high pH	8	48.4	52.4%	0.042	48.4
Mometasone	API	2021_fall	DB	high pH	3	2.74	98.8%	0.071	2.74
Naloxegol	API	2021_fall	DB	low pH	8	-4495	2.4%	0.715	>474
Naloxegol	API	2021_fall	DB	low pH	8	13406	0.2%	0.924	>474
Naloxegol	API	2021_fall	HB	low pH	8	335	54.1%	0.038	335
Naloxegol	API	2021_fall	HB	low pH	8	332	62.9%	0.019	332
Naloxegol	API	2021_fall	DB	high pH	8	42.7	97.9%	0.000	42.7
Naloxegol	API	2021_fall	DB	high pH	8	39.9	98.2%	0.000	39.9
Naloxegol	API	2021_fall	HB	high pH	5	11.6	81.1%	0.037	11.6
Naloxegol	API	2021_fall	HB	high pH	4	4.31	100.0%	0.000	4.31
Napropamide	PPP	2018_summer	DB	high pH	6	582	91.8%	0.003	>316
Napropamide	PPP	2018_summer	DB	high pH	6	658	47.2%	0.132	>316
Napropamide	PPP	2018_summer	DB	high pH	6	2132	8.3%	0.579	>316
Napropamide	PPP	2018_summer	NB	high pH	5	179	92.5%	0.009	179
Napropamide	PPP	2018_summer	NB	high pH	5	176	97.5%	0.002	176
Napropamide	PPP	2018_summer	NB	high pH	5	174	98.8%	0.001	174
Napropamide	PPP	2018_summer	HB	high pH	6	50.6	99.6%	0.000	50.6
Napropamide	PPP	2018_summer	HB	high pH	6	50.5	99.5%	0.000	50.5
Napropamide	PPP	2018_summer	HB	high pH	6	48.0	99.4%	0.000	48.0
Nilotinib	API	2021_fall	DB	high pH	9	271	23.9%	0.181	NA
Nilotinib	API	2021_fall	DB	low pH	8	352	13.8%	0.365	NA
Nilotinib	API	2021_fall	DB	high pH	9	703	3.2%	0.643	NA
Nilotinib	API	2021_fall	DB	low pH	8	741	4.4%	0.617	NA
Olanzepine	API	2021_fall	DB	low pH	3	4.05	74.8%	0.335	<1.2
Olanzepine	API	2021_fall	DB	low pH	4	12.1	45.3%	0.327	<1.2
Olanzepine	API	2021_fall	DB	high pH	4	12.3	65.5%	0.191	<1.2
Olanzepine	API	2021_fall	DB	high pH	4	14.0	70.2%	0.162	<1.2
Olanzepine	API	2021_fall	HB	low pH	4	24.4	87.6%	0.064	<1.2
Olanzepine	API	2021_fall	HB	low pH	4	31.7	97.4%	0.013	<1.2
Olanzepine	API	2021_fall	HB	high pH	4	39.2	51.5%	0.283	<1.2
Olanzepine	API	2021_fall	HB	high pH	4	48.6	56.7%	0.247	<1.2
Omeprazole	API	2021_fall	DB	high pH	8	307	85.9%	0.001	307
Omeprazole	API	2021_fall	DB	high pH	8	239	90.3%	0.000	239
Omeprazole	API	2021_fall	HB	high pH	6	56.7	93.1%	0.002	56.7
Omeprazole	API	2021_fall	HB	high pH	6	43.6	89.1%	0.005	43.6
Omeprazole	API	2021_fall	HB	low pH	6	14.0	98.4%	0.000	14.0

Omeprazole	API	2021_fall	HB	low pH	6	12.7	98.2%	0.000	12.7
Omeprazole	API	2021_fall	DB	low pH	5	12.6	98.9%	0.000	12.6
Omeprazole	API	2021_fall	DB	low pH	5	8.35	96.6%	0.003	8.35
Oxathiapiprolin	reference	2018_summer	HB	high pH	2	6.93	100.0%	NA	<1.2
Oxathiapiprolin	reference	2018_summer	HB	high pH	3	10.9	92.5%	0.177	<1.2
Oxathiapiprolin	reference	2018_summer	HB	high pH	3	12.2	93.3%	0.167	<1.2
Oxathiapiprolin	reference	2018_summer	DB	high pH	6	56.4	94.4%	0.001	56.4
Oxathiapiprolin	reference	2018_summer	DB	high pH	6	55.5	99.0%	0.000	55.5
Oxathiapiprolin	reference	2018_summer	DB	high pH	6	52.2	95.4%	0.001	52.2
Oxathiapiprolin	reference	2018_summer	NB	high pH	4	23.2	91.9%	0.041	23.2
Oxathiapiprolin	reference	2018_summer	NB	high pH	3	19.2	95.5%	0.136	19.2
Oxathiapiprolin	reference	2018_summer	NB	high pH	3	17.7	94.8%	0.146	17.7
Oxathiapiprolin	reference	2020_fall	HB	high pH	6	61.9	97.3%	0.002	61.6
Oxathiapiprolin	reference	2020_fall	HB	high pH	7	53.7	96.9%	0.000	53.6
Oxathiapiprolin	reference	2020_fall	DB	low pH	7	28.9	98.1%	0.000	28.9
Oxathiapiprolin	reference	2020_fall	DB	low pH	7	25.8	99.2%	0.000	25.8
Oxathiapiprolin	reference	2020_fall	DB	high pH	7	21.5	98.3%	0.000	21.6
Oxathiapiprolin	reference	2020_fall	DB	high pH	7	20.8	99.9%	0.000	20.8
Oxathiapiprolin	reference	2020_fall	HB	low pH	4	6.21	98.4%	0.080	6.20
Oxathiapiprolin	reference	2020_fall	HB	low pH	4	5.04	99.9%	0.021	5.00
Oxathiapiprolin	reference	2021_fall	DB	low pH	8	139	79.4%	0.003	139
Oxathiapiprolin	reference	2021_fall	DB	low pH	8	109	92.7%	0.000	109
Oxathiapiprolin	reference	2021_fall	DB	high pH	8	24.8	99.6%	0.000	24.8
Oxathiapiprolin	reference	2021_fall	DB	high pH	8	24.6	99.5%	0.000	24.6
Oxathiapiprolin	reference	2021_fall	HB	low pH	9	19.6	93.5%	0.000	19.6
Oxathiapiprolin	reference	2021_fall	HB	low pH	6	11.8	98.7%	0.000	11.8
Oxathiapiprolin	reference	2021_fall	HB	high pH	3	3.72	93.6%	0.163	3.72
Oxathiapiprolin	reference	2021_fall	HB	high pH	3	3.15	99.7%	0.033	3.15
Oxathiapiprolin	reference	2021_summer	HB_VH	low pH	6	16.2	94.8%	0.001	16.2
Oxathiapiprolin	reference	2021_summer	HB_VH	low pH	6	15.4	94.8%	0.001	15.4
Oxathiapiprolin	reference	2021_summer	HB	low pH	5	12.0	95.2%	0.004	12.0
Oxathiapiprolin	reference	2021_summer	HB	low pH	5	11.5	97.3%	0.002	11.5
Oxathiapiprolin	reference	2021_summer	HB_VH	high pH	5	10.6	75.2%	0.057	10.6
Oxathiapiprolin	reference	2021_summer	HB_VH	high pH	4	4.79	99.0%	0.005	4.79
Oxathiapiprolin	reference	2021_summer	HB	high pH	4	4.57	97.6%	0.012	4.57
Oxathiapiprolin	reference	2021_summer	HB	high pH	4	4.17	98.7%	0.006	4.17
Oxathiapiprolin	reference	2021_winter	HB	low pH	4	25.8	77.8%	0.118	<1.2
Oxathiapiprolin	reference	2021_winter	HB	low pH	4	26.9	72.3%	0.150	<1.2
Oxathiapiprolin	reference	2021_winter	HB	low pH	5	43.3	65.9%	0.095	<1.2
Oxathiapiprolin	reference	2021_winter	HB	high pH	6	99.0	90.9%	0.003	98.8
Oxathiapiprolin	reference	2021_winter	HB	high pH	5	84.5	98.0%	0.001	84.2
Oxathiapiprolin	reference	2021_winter	HB	high pH	6	77.9	94.5%	0.001	77.9
Oxathiapiprolin	reference	2021_winter	DB	low pH	6	42.0	96.5%	0.000	42.0
Oxathiapiprolin	reference	2021_winter	DB	low pH	6	35.0	95.3%	0.001	34.9
Oxathiapiprolin	reference	2021_winter	DB	low pH	6	32.2	93.2%	0.002	32.2
Oxathiapiprolin	reference	2021_winter	DB	high pH	6	24.0	93.6%	0.002	23.9
Oxathiapiprolin	reference	2021_winter	DB	high pH	6	23.4	93.5%	0.002	23.4
Oxathiapiprolin	reference	2021_winter	DB	high pH	6	23.1	92.5%	0.002	23.1
Panobinostat	API	2021_fall	DB	low pH	3	6.92	98.4%	0.082	<1.2
Panobinostat	API	2021_fall	DB	high pH	3	11.4	72.3%	0.353	<1.2
Panobinostat	API	2021_fall	DB	high pH	3	17.8	72.1%	0.354	<1.2
Panobinostat	API	2021_fall	DB	low pH	3	9.20	99.9%	0.020	9.20
Pemetrexed	API	2021_fall	DB	high pH	2	NA	NA	NA	<1.2
Pemetrexed	API	2021_fall	DB	high pH	2	NA	NA	NA	<1.2
Pemetrexed	API	2021_fall	DB	low pH	2	NA	NA	NA	<1.2
Pemetrexed	API	2021_fall	DB	low pH	2	NA	NA	NA	<1.2
Picoxystrobin	PPP	2018_summer	DB	high pH	6	26.2	99.6%	0.000	26.2

Picoxystrobin	PPP	2018_summer	DB	high pH	6	25.8	99.3%	0.000	25.8
Picoxystrobin	PPP	2018_summer	DB	high pH	6	25.7	99.1%	0.000	25.7
Picoxystrobin	PPP	2018_summer	NB	high pH	3	11.6	99.6%	0.038	11.6
Picoxystrobin	PPP	2018_summer	NB	high pH	2	10.1	100.0%	NA	10.1
Picoxystrobin	PPP	2018_summer	NB	high pH	2	9.64	100.0%	NA	9.64
Picoxystrobin	PPP	2018_summer	HB	high pH	3	9.60	98.8%	0.070	9.60
Picoxystrobin	PPP	2018_summer	HB	high pH	3	9.21	99.6%	0.041	9.21
Picoxystrobin	PPP	2018_summer	HB	high pH	3	8.12	99.9%	0.019	8.12
Pinoxaden	PPP	2018_summer	NB	high pH	2	7.14	100.0%	NA	<1.2
Pinoxaden	PPP	2018_summer	NB	high pH	2	7.24	100.0%	NA	<1.2
Pinoxaden	PPP	2018_summer	HB	high pH	1	NA	NA	NA	<1.2
Pinoxaden	PPP	2018_summer	HB	high pH	1	NA	NA	NA	<1.2
Pinoxaden	PPP	2018_summer	HB	high pH	1	NA	NA	NA	<1.2
Pinoxaden	PPP	2018_summer	NB	high pH	1	NA	NA	NA	<1.2
Pinoxaden	PPP	2018_summer	DB	high pH	4	17.0	87.8%	0.063	17.0
Pinoxaden	PPP	2018_summer	DB	high pH	4	16.9	87.9%	0.063	16.9
Pinoxaden	PPP	2018_summer	DB	high pH	4	16.2	86.8%	0.068	16.2
Pioglitazone	API	2021_fall	DB	low pH	2	NA	NA	NA	<1.2
Pioglitazone	API	2021_fall	DB	low pH	2	NA	NA	NA	<1.2
Pioglitazone	API	2021_fall	HB	high pH	2	NA	NA	NA	<1.2
Pioglitazone	API	2021_fall	HB	high pH	2	NA	NA	NA	<1.2
Pioglitazone	API	2021_fall	HB	low pH	2	NA	NA	NA	<1.2
Pioglitazone	API	2021_fall	HB	low pH	2	NA	NA	NA	<1.2
Pioglitazone	API	2021_fall	DB	high pH	3	2.22	95.1%	0.143	2.22
Pioglitazone	API	2021_fall	DB	high pH	3	2.02	97.2%	0.108	2.02
Proquinazid	PPP	2018_summer	HB	high pH	1	NA	NA	NA	<1.2
Proquinazid	PPP	2018_summer	HB	high pH	1	NA	NA	NA	<1.2
Proquinazid	PPP	2018_summer	HB	high pH	1	NA	NA	NA	<1.2
Proquinazid	PPP	2018_summer	NB	high pH	1	NA	NA	NA	<1.2
Proquinazid	PPP	2018_summer	NB	high pH	1	NA	NA	NA	<1.2
Proquinazid	PPP	2018_summer	NB	high pH	1	NA	NA	NA	<1.2
Proquinazid	PPP	2018_summer	DB	high pH	3	10.8	97.4%	0.104	10.8
Proquinazid	PPP	2018_summer	DB	high pH	3	10.8	97.6%	0.099	10.8
Proquinazid	PPP	2018_summer	DB	high pH	3	10.5	100.0%	0.007	10.5
Pyroxsulam	PPP	2018_summer	HB	high pH	6	-1337	22.5%	0.342	>316
Pyroxsulam	PPP	2018_summer	NB	high pH	5	-1228	46.0%	0.208	>316
Pyroxsulam	PPP	2018_summer	DB	high pH	6	-1171	6.3%	0.632	>316
Pyroxsulam	PPP	2018_summer	NB	high pH	5	-1134	16.3%	0.501	>316
Pyroxsulam	PPP	2018_summer	DB	high pH	6	-928	17.4%	0.410	>316
Pyroxsulam	PPP	2018_summer	NB	high pH	5	-473	57.1%	0.140	>316
Pyroxsulam	PPP	2018_summer	DB	high pH	6	-454	79.7%	0.017	>316
Pyroxsulam	PPP	2018_summer	HB	high pH	6	-360	57.6%	0.080	>316
Pyroxsulam	PPP	2018_summer	HB	high pH	6	1441	14.6%	0.454	>316
Quetiapine	API	2021_fall	HB	high pH	2	NA	NA	NA	<1.2
Quetiapine	API	2021_fall	HB	high pH	2	NA	NA	NA	<1.2
Quetiapine	API	2021_fall	DB	low pH	6	11.5	97.5%	0.000	11.5
Quetiapine	API	2021_fall	DB	low pH	6	11.4	98.7%	0.000	11.4
Quetiapine	API	2021_fall	DB	high pH	4	3.62	99.9%	0.001	3.62
Quetiapine	API	2021_fall	DB	high pH	4	3.49	100.0%	0.000	3.49
Quetiapine	API	2021_fall	HB	low pH	3	1.27	98.9%	0.066	1.27
Quetiapine	API	2021_fall	HB	low pH	3	1.12	100.0%	0.014	1.12
Regorafenib	API	2021_fall	DB	low pH	9	-1161	0.4%	0.877	NA
Regorafenib	API	2021_fall	DB	low pH	9	-269	8.1%	0.457	NA
Regorafenib	API	2021_fall	HB	low pH	8	-205	62.7%	0.019	NA
Regorafenib	API	2021_fall	DB	high pH	9	287	6.6%	0.506	NA
Regorafenib	API	2021_fall	HB	low pH	8	308	40.8%	0.088	NA
Regorafenib	API	2021_fall	DB	high pH	9	390	5.8%	0.531	NA

Rivastigmin	API	2021_fall	HB	high pH	9	-227	53.7%	0.025	NA
Rivastigmin	API	2021_fall	HB	high pH	9	-211	60.8%	0.013	NA
Rivastigmin	API	2021_fall	HB	low pH	8	-1027	62.0%	0.020	>474
Rivastigmin	API	2021_fall	DB	low pH	8	-782	51.3%	0.046	>474
Rivastigmin	API	2021_fall	DB	low pH	8	-716	78.5%	0.003	>474
Rivastigmin	API	2021_fall	HB	low pH	8	-703	61.3%	0.022	>474
Rivastigmin	API	2021_fall	DB	high pH	8	883	25.5%	0.202	>474
Rivastigmin	API	2021_fall	DB	high pH	8	1052	19.2%	0.278	>474
Rosuvastatin	API	2021_fall	HB	high pH	2	NA	NA	NA	<1.2
Rosuvastatin	API	2021_fall	HB	high pH	2	NA	NA	NA	<1.2
Rosuvastatin	API	2021_fall	HB	low pH	2	NA	NA	NA	<1.2
Rosuvastatin	API	2021_fall	HB	low pH	2	NA	NA	NA	<1.2
Rosuvastatin	API	2021_fall	DB	low pH	8	335	81.8%	0.002	335
Rosuvastatin	API	2021_fall	DB	low pH	8	197	87.6%	0.001	197
Rosuvastatin	API	2021_fall	DB	high pH	5	6.65	96.5%	0.003	6.65
Rosuvastatin	API	2021_fall	DB	high pH	5	6.19	95.6%	0.004	6.19
Sedaxane	PPP	2018_summer	NB	high pH	8	-803144	0.0%	0.999	>316
Sedaxane	PPP	2018_summer	DB	high pH	8	-786	37.1%	0.109	>316
Sedaxane	PPP	2018_summer	DB	high pH	8	-762	59.9%	0.024	>316
Sedaxane	PPP	2018_summer	DB	high pH	8	-372	36.9%	0.110	>316
Sedaxane	PPP	2018_summer	NB	high pH	8	939	23.3%	0.273	>316
Sedaxane	PPP	2018_summer	HB	high pH	8	174	89.7%	0.000	174
Sedaxane	PPP	2018_summer	HB	high pH	8	147	99.9%	0.000	147
Spirotetramat	PPP	2018_summer	HB	high pH	1	NA	NA	NA	<1.2
Spirotetramat	PPP	2018_summer	HB	high pH	1	NA	NA	NA	<1.2
Spirotetramat	PPP	2018_summer	HB	high pH	1	NA	NA	NA	<1.2
Spirotetramat	PPP	2018_summer	NB	high pH	1	NA	NA	NA	<1.2
Spirotetramat	PPP	2018_summer	NB	high pH	1	NA	NA	NA	<1.2
Spirotetramat	PPP	2018_summer	NB	high pH	1	NA	NA	NA	<1.2
Spirotetramat	PPP	2018_summer	DB	high pH	3	5.75	99.6%	0.040	5.75
Spirotetramat	PPP	2018_summer	DB	high pH	3	5.49	99.4%	0.050	5.49
Spirotetramat	PPP	2018_summer	DB	high pH	3	5.40	99.3%	0.055	5.40
Sulfoxaflor	PPP	2018_summer	DB	high pH	8	321	57.9%	0.028	321
Sulfoxaflor	PPP	2018_summer	DB	high pH	8	420	79.7%	0.003	>316
Sulfoxaflor	PPP	2018_summer	DB	high pH	8	770	13.9%	0.364	>316
Sulfoxaflor	PPP	2018_summer	NB	high pH	8	135	95.7%	0.000	135
Sulfoxaflor	PPP	2018_summer	NB	high pH	8	114	97.9%	0.000	114
Sulfoxaflor	PPP	2018_summer	HB	high pH	8	26.2	98.7%	0.000	26.2
Sulfoxaflor	PPP	2018_summer	HB	high pH	8	25.4	97.7%	0.000	25.4
Tadalafil	API	2021_fall	DB	low pH	8	549	67.5%	0.012	549
Tadalafil	API	2021_fall	DB	low pH	8	684	43.9%	0.074	>474
Tadalafil	API	2021_fall	DB	high pH	8	143	97.4%	0.000	143
Tadalafil	API	2021_fall	DB	high pH	8	131	98.0%	0.000	131
Tadalafil	API	2021_fall	HB	low pH	8	127	74.3%	0.006	127
Tadalafil	API	2021_fall	HB	low pH	8	94.1	83.1%	0.002	94.1
Tadalafil	API	2021_fall	HB	high pH	5	18.1	97.6%	0.002	18.1
Tadalafil	API	2021_fall	HB	high pH	5	16.7	99.0%	0.000	16.7
Tembotrione	PPP	2018_summer	DB	high pH	6	-17275	0.6%	0.888	>316
Tembotrione	PPP	2018_summer	NB	high pH	5	674	63.0%	0.109	>316
Tembotrione	PPP	2018_summer	NB	high pH	5	795	74.1%	0.061	>316
Tembotrione	PPP	2018_summer	NB	high pH	5	833	48.9%	0.189	>316
Tembotrione	PPP	2018_summer	DB	high pH	6	1031	25.3%	0.309	>316
Tembotrione	PPP	2018_summer	DB	high pH	6	3310	1.9%	0.793	>316
Tembotrione	PPP	2018_summer	HB	high pH	6	71.1	99.7%	0.000	71.1
Tembotrione	PPP	2018_summer	HB	high pH	6	67.0	98.3%	0.000	67.0
Tembotrione	PPP	2018_summer	HB	high pH	6	66.6	99.3%	0.000	66.6
Terbinafine	API	2021_fall	HB	high pH	9	-131	26.9%	0.152	<1.2

Terbinafine	API	2021_fall	HB	high pH	9	-92.2	61.4%	0.012	<1.2
Terbinafine	API	2021_fall	DB	high pH	9	47.7	95.0%	0.000	<1.2
Terbinafine	API	2021_fall	DB	high pH	9	59.6	93.1%	0.000	<1.2
Terbinafine	API	2021_fall	HB	low pH	9	-29448	0.0%	0.974	>474
Terbinafine	API	2021_fall	HB	low pH	9	-3415	1.0%	0.802	>474
Terbinafine	API	2021_fall	DB	low pH	9	-1552	4.0%	0.606	>474
Terbinafine	API	2021_fall	DB	low pH	9	-1031	11.3%	0.376	>474
Terbuthylazine	reference	2018_summer	DB	high pH	6	-12988	3.3%	0.731	>316
Terbuthylazine	reference	2018_summer	DB	high pH	6	-2892	5.6%	0.651	>316
Terbuthylazine	reference	2018_summer	NB	high pH	5	711	54.4%	0.155	>316
Terbuthylazine	reference	2018_summer	NB	high pH	5	1163	52.3%	0.167	>316
Terbuthylazine	reference	2018_summer	NB	high pH	5	1313	37.2%	0.275	>316
Terbuthylazine	reference	2018_summer	DB	high pH	6	6068	14.0%	0.465	>316
Terbuthylazine	reference	2018_summer	HB	high pH	6	151	98.8%	0.000	151
Terbuthylazine	reference	2018_summer	HB	high pH	6	139	99.7%	0.000	139
Terbuthylazine	reference	2018_summer	HB	high pH	6	130	99.0%	0.000	130
Terbuthylazine	reference	2020_fall	HB	low pH	7	385	79.1%	0.018	380
Terbuthylazine	reference	2020_fall	HB	low pH	7	187	58.9%	0.075	>316
Terbuthylazine	reference	2020_fall	DB	low pH	7	630	21.6%	0.353	>316
Terbuthylazine	reference	2020_fall	DB	high pH	6	770	36.4%	0.281	>316
Terbuthylazine	reference	2020_fall	DB	high pH	7	1386	17.1%	0.415	>316
Terbuthylazine	reference	2020_fall	DB	low pH	2	1733	100.0%	NA	>316
Terbuthylazine	reference	2020_fall	HB	high pH	6	315	74.9%	0.058	315
Terbuthylazine	reference	2020_fall	HB	high pH	7	277	77.8%	0.020	273
Terbuthylazine	reference	2021_fall	DB	low pH	8	-5876	4.2%	0.625	>474
Terbuthylazine	reference	2021_fall	HB	low pH	8	637	80.4%	0.003	>474
Terbuthylazine	reference	2021_fall	HB	low pH	8	799	66.9%	0.013	>474
Terbuthylazine	reference	2021_fall	DB	low pH	8	1043	27.3%	0.184	>474
Terbuthylazine	reference	2021_fall	DB	high pH	8	4123	26.4%	0.193	>474
Terbuthylazine	reference	2021_fall	DB	high pH	8	19173	0.2%	0.910	>474
Terbuthylazine	reference	2021_fall	HB	high pH	8	342	94.2%	0.000	342
Terbuthylazine	reference	2021_fall	HB	high pH	8	277	92.9%	0.000	277
Terbuthylazine	reference	2021_summer	HB	low pH	9	1049	28.4%	0.139	>474
Terbuthylazine	reference	2021_summer	HB	low pH	9	1145	44.7%	0.049	>474
Terbuthylazine	reference	2021_summer	HB_VH	low pH	9	1180	63.1%	0.011	>474
Terbuthylazine	reference	2021_summer	HB_VH	low pH	9	1267	50.4%	0.032	>474
Terbuthylazine	reference	2021_summer	HB	high pH	9	378	94.8%	0.000	378
Terbuthylazine	reference	2021_summer	HB	high pH	9	337	92.3%	0.000	337
Terbuthylazine	reference	2021_summer	HB_VH	high pH	9	233	97.9%	0.000	233
Terbuthylazine	reference	2021_summer	HB_VH	high pH	9	175	97.6%	0.000	175
Terbuthylazine	reference	2021_winter	HB	high pH	6	385	91.0%	0.003	381
Terbuthylazine	reference	2021_winter	HB	low pH	6	330	96.7%	0.000	324
Terbuthylazine	reference	2021_winter	DB	low pH	6	-533	69.3%	0.040	>316
Terbuthylazine	reference	2021_winter	DB	low pH	6	408	39.8%	0.179	>316
Terbuthylazine	reference	2021_winter	HB	low pH	6	433	86.9%	0.007	>316
Terbuthylazine	reference	2021_winter	HB	low pH	6	462	81.3%	0.014	>316
Terbuthylazine	reference	2021_winter	DB	low pH	6	866	97.1%	0.000	>316
Terbuthylazine	reference	2021_winter	HB	high pH	6	866	84.9%	0.009	>316
Terbuthylazine	reference	2021_winter	DB	high pH	6	1155	31.4%	0.248	>316
Terbuthylazine	reference	2021_winter	HB	high pH	6	1155	17.3%	0.413	>316
Terbuthylazine	reference	2021_winter	DB	high pH	6	1386	53.8%	0.097	>316
Terbuthylazine	reference	2021_winter	DB	high pH	6	2310	7.4%	0.603	>316
Ticagrelor	API	2021_fall	HB	high pH	4	40.4	64.2%	0.199	<1.2
Ticagrelor	API	2021_fall	HB	high pH	4	53.7	45.4%	0.326	<1.2
Ticagrelor	API	2021_fall	HB	low pH	4	153	11.6%	0.660	<1.2
Ticagrelor	API	2021_fall	HB	low pH	5	317	52.2%	0.168	<1.2
Ticagrelor	API	2021_fall	DB	low pH	9	136	76.6%	0.002	136

Ticagrelor	API	2021_fall	DB	low pH	8	110	86.4%	0.001	110
Ticagrelor	API	2021_fall	DB	high pH	7	81.7	84.4%	0.003	81.7
Ticagrelor	API	2021_fall	DB	high pH	6	64.5	85.5%	0.008	64.5
Topramezone	reference	2018_summer	DB	high pH	6	237	47.8%	0.128	>316
Topramezone	reference	2018_summer	DB	high pH	6	199	83.5%	0.011	199
Topramezone	reference	2018_summer	NB	high pH	5	195	93.6%	0.007	195
Topramezone	reference	2018_summer	DB	high pH	6	178	91.3%	0.003	178
Topramezone	reference	2018_summer	NB	high pH	5	173	96.3%	0.003	173
Topramezone	reference	2018_summer	NB	high pH	5	158	97.3%	0.002	158
Topramezone	reference	2018_summer	HB	high pH	6	108	93.9%	0.001	108
Topramezone	reference	2018_summer	HB	high pH	6	95.3	93.1%	0.002	95.3
Topramezone	reference	2018_summer	HB	high pH	6	90.9	95.5%	0.001	90.9
Topramezone	reference	2020_fall	HB	low pH	7	347	66.4%	0.048	349
Topramezone	reference	2020_fall	DB	low pH	7	-1733	7.0%	0.611	>316
Topramezone	reference	2020_fall	DB	high pH	7	-578	35.0%	0.216	>316
Topramezone	reference	2020_fall	DB	high pH	7	-330	67.2%	0.046	>316
Topramezone	reference	2020_fall	HB	high pH	7	433	58.2%	0.078	>316
Topramezone	reference	2020_fall	HB	high pH	6	630	56.9%	0.140	>316
Topramezone	reference	2020_fall	DB	low pH	7	2310	15.0%	0.448	>316
Topramezone	reference	2020_fall	DB	low pH	6	161	84.4%	0.027	162
Topramezone	reference	2021_summer	HB_VH	high pH	9	699	44.3%	0.051	>474
Topramezone	reference	2021_summer	HB_VH	high pH	9	792	50.7%	0.031	>474
Topramezone	reference	2021_summer	HB_VH	low pH	9	840	57.9%	0.017	>474
Topramezone	reference	2021_summer	HB_VH	low pH	9	1641	15.8%	0.289	>474
Topramezone	reference	2021_summer	HB	low pH	9	375	64.6%	0.009	375
Topramezone	reference	2021_summer	HB	low pH	9	351	77.7%	0.002	351
Topramezone	reference	2021_summer	HB	high pH	9	213	91.0%	0.000	213
Topramezone	reference	2021_summer	HB	high pH	9	166	93.8%	0.000	166
Topramezone	reference	2021_winter	DB	high pH	5	-1386	9.6%	0.611	>316
Topramezone	reference	2021_winter	DB	low pH	6	-315	34.0%	0.224	>316
Topramezone	reference	2021_winter	HB	low pH	6	79.7	53.6%	0.098	>316
Topramezone	reference	2021_winter	HB	low pH	6	82.5	48.0%	0.127	>316
Topramezone	reference	2021_winter	DB	low pH	6	103	40.2%	0.177	>316
Topramezone	reference	2021_winter	DB	high pH	6	122	54.3%	0.095	>316
Topramezone	reference	2021_winter	HB	high pH	6	158	1.8%	0.798	>316
Topramezone	reference	2021_winter	HB	high pH	6	165	1.7%	0.807	>316
Topramezone	reference	2021_winter	DB	low pH	6	173	31.7%	0.245	>316
Topramezone	reference	2021_winter	HB	low pH	6	365	15.7%	0.436	>316
Topramezone	reference	2021_winter	DB	high pH	6	2310	0.1%	0.965	>316
Topramezone	reference	2021_winter	HB	high pH	5	NA	0.0%	0.975	>316
Trinexapac-ethyl	PPP	2018_summer	HB	high pH	1	NA	NA	NA	<1.2
Trinexapac-ethyl	PPP	2018_summer	HB	high pH	1	NA	NA	NA	<1.2
Trinexapac-ethyl	PPP	2018_summer	HB	high pH	1	NA	NA	NA	<1.2
Trinexapac-ethyl	PPP	2018_summer	DB	high pH	6	67.9	99.6%	0.000	67.9
Trinexapac-ethyl	PPP	2018_summer	DB	high pH	6	67.0	99.9%	0.000	67.0
Trinexapac-ethyl	PPP	2018_summer	DB	high pH	6	65.5	98.3%	0.000	65.5
Trinexapac-ethyl	PPP	2018_summer	NB	high pH	4	14.1	98.5%	0.007	14.1
Trinexapac-ethyl	PPP	2018_summer	NB	high pH	4	14.0	98.4%	0.008	14.0
Trinexapac-ethyl	PPP	2018_summer	NB	high pH	4	13.8	99.0%	0.005	13.8
Valifenalate	PPP	2018_summer	NB	high pH	4	23.8	70.2%	0.162	<1.2
Valifenalate	PPP	2018_summer	NB	high pH	5	31.0	41.7%	0.240	<1.2
Valifenalate	PPP	2018_summer	NB	high pH	5	33.2	39.0%	0.260	<1.2
Valifenalate	PPP	2018_summer	HB	high pH	5	120	44.2%	0.221	<1.2
Valifenalate	PPP	2018_summer	HB	high pH	4	132	69.6%	0.166	<1.2
Valifenalate	PPP	2018_summer	HB	high pH	5	171	26.0%	0.380	<1.2
Valifenalate	PPP	2018_summer	DB	high pH	6	19.1	93.3%	0.002	19.1
Valifenalate	PPP	2018_summer	DB	high pH	6	18.2	93.6%	0.002	18.2

Valifenalate	PPP	2018_summer	DB	high pH	5	15.0	99.3%	0.000	15.0
Valsartan	API	2021_fall	HB	low pH	4	3.54	81.0%	0.100	<1.2
Valsartan	API	2021_fall	HB	low pH	4	3.67	79.2%	0.110	<1.2
Valsartan	API	2021_fall	HB	high pH	4	3.68	64.4%	0.198	<1.2
Valsartan	API	2021_fall	HB	high pH	4	3.71	62.4%	0.210	<1.2
Valsartan	API	2021_fall	DB	low pH	8	81.4	82.0%	0.002	81.4
Valsartan	API	2021_fall	DB	low pH	8	71.5	80.2%	0.003	71.5
Valsartan	API	2021_fall	DB	high pH	8	30.4	95.0%	0.000	30.4
Valsartan	API	2021_fall	DB	high pH	8	26.6	94.4%	0.000	26.6
Vildagliptin	API	2021_fall	HB	low pH	9	897	43.5%	0.053	>474
Vildagliptin	API	2021_fall	HB	low pH	9	1067	18.7%	0.245	>474
Vildagliptin	API	2021_fall	DB	low pH	8	1405	24.3%	0.214	>474
Vildagliptin	API	2021_fall	DB	low pH	8	1925	44.2%	0.072	>474
Vildagliptin	API	2021_fall	DB	high pH	8	372	87.6%	0.001	372
Vildagliptin	API	2021_fall	DB	high pH	8	356	95.4%	0.000	356
Vildagliptin	API	2021_fall	HB	high pH	9	161	93.3%	0.000	161
Vildagliptin	API	2021_fall	HB	high pH	9	147	90.1%	0.000	147
Vorinostat	API	2021_fall	HB	high pH	3	1.33	96.5%	0.121	<1.2
Vorinostat	API	2021_fall	HB	high pH	2	NA	NA	NA	<1.2
Vorinostat	API	2021_fall	DB	low pH	9	18.7	96.5%	0.000	18.7
Vorinostat	API	2021_fall	DB	low pH	9	17.9	96.8%	0.000	17.9
Vorinostat	API	2021_fall	DB	high pH	4	2.64	99.7%	0.001	2.64
Vorinostat	API	2021_fall	DB	high pH	4	2.44	99.9%	0.000	2.44
Vorinostat	API	2021_fall	HB	low pH	4	1.95	99.2%	0.004	1.95
Vorinostat	API	2021_fall	HB	low pH	3	1.51	100.0%	0.008	1.51

S5.4 Bayesian mean of log DegT50$_{sludge}$ of each compound

The Bayesian approach to calculating mean logDegT50$_{sludge}$ allowed us to use all data available from the sludge experiments, i.e., censored and uncensored values. The prior was chosen to reflect the average distribution of half-lives for a single compound in different experimental setups. In studies following OECD 307 guidelines, a first sample is generally taken after 1 day, and a final sample at 120 days. Therefore, reliable half-life values can be obtained between 0.1 and 1000 days, with values lying beyond this range being generally reported as censored values. In sludge, a first sample was first obtained after 2 hours and final sample at 48 hours. According to the reporting limits, reasonable half-lives could be obtained between 1.2h or -1.3 log(days) and 473h or 1.3 log(days), everything beyond being reported as censored. The distribution of the mean was therefore chosen to well cover this range, with a mean at the center of this range, at 0 log(days), or 1 day. We assumed the standard deviation to be smaller for sludge experiments than for OECD 307 studies, as the sludge experiments are considered to be more homogenous than soil experiments, where regulation requires testing in three different soil types.

The range covered by the 0.25 and 0.75 quantiles of the posterior distributions (indicators of variation and uncertainty in the distribution of log DegT50$_{sludge}$) is larger for compounds for which less log DegT50$_{sludge}$'s could be obtained (e.g., due to non-detects), where some of the DegT50$_{sludge}$ of the compounds could only be indicated as censored values (i.e., below 0.05 d or above 13 d), or where there is actual high variation in the log DegT50$_{sludge}$. For example, kresoxim-methyl has the widest 50th percentile interval in log DegT50$_{sludge}$ among the reference

compounds (>1 log unit) because despite showing consistency in degradation there were only 9 log $DegT50_{sludge}$ values and 8 censored values from 2 experiments to perform the Bayesian update. Summary parameters of the posterior distributions by experiment are reported in Table S8.

Table S7. Log $DegT50_{sludge}$ parameters obtained from Bayesian inference for reference compounds, using kinetic data as available from all experiments. $DegT50_{sludge}$ given in days were used for the calculation of the parameters.

Reference	**Mean** log $DegT50_{sludge}$	**Standard deviation**	**Standard deviation of the mean**	**Quantile 0.25**	**Quantile 0.75**
Azoxystrobin	0.56	0.6	0.09	0.16	0.96
Benzovindiflupyr	1.42	0.57	0.15	1.04	1.80
Bromoxynil	-0.58	0.73	0.2	-1.07	-0.09
Cyantraniliprole	1.25	0.53	0.12	0.89	1.61
Cyclaniliprole	1.48	0.32	0.18	1.26	1.70
Dicamba	0.97	0.35	0.08	0.73	1.21
Diuron	1.07	0.5	0.09	0.73	1.41
Fenhexamid	0.26	0.42	0.06	-0.02	0.54
Fenoxycarb	-2.21	0.22	0.71	-2.36	-2.06
Fipronil	1.17	0.25	0.05	1.00	1.34
Florasulam	1.2	0.33	0.07	0.98	1.42
Fluopyram	1.46	0.3	0.16	1.26	1.66
Flupyradifurone	1.35	0.45	0.12	1.05	1.65
Imidacloprid	1.28	0.32	0.09	1.06	1.50
Isoproturon	0.73	0.53	0.08	0.37	1.09
Kresoxim-methyl	-1.16	0.76	0.24	-1.67	-0.65
Mandipropamid	-0.36	0.61	0.09	-0.77	0.05
Mesotrione	0.92	0.66	0.11	0.47	1.37
Oxathiapiprolin	-0.19	0.55	0.08	-0.56	0.18
Terbuthylazine	1.44	0.35	0.15	1.20	1.68
Topramezone	1.35	0.43	0.13	1.06	1.64

Bayesian means for each compound per experiment are listed in Table S7 below, along with the quantiles of the posterior distribution. For experiment 2021-summer, different posterior distributions were derived for activated sludge from the WWTP at Eawag experimental hall (VH) and from WWTP Neugut (NE).

Table S8. log $DegT50_{sludge}$ [d] Bayesian posterior parameters of all compounds by experiment. Parameters were estimated separately for the WWTP Neugut (NE) and the Eawag experimental hall (NE) for 2021-summer.

**Compound**	**Compound set**	**Experiment**	**Mean**	**Standard deviation**	**Standard deviation of the mean**	**Quantile 0.25**	**Quantile 0.75**
Acalabrutinib	test API	2021-fall	0.44	0.87	0.33	-0.15	1.03
Aliskiren	test API	2021-fall	1.06	0.44	0.19	0.76	1.36
Amlodipine	test API	2021-fall	-0.06	0.72	0.28	-0.55	0.43
Asulam	test PPP	2018-summer	0.65	0.67	0.25	0.2	1.1
Atazanavir	test API	2021-fall	1.05	0.44	0.2	0.75	1.35
Atomoxetin	test API	2021-fall	2.25	0.22	0.68	2.1	2.4
Atovaquone	test API	2021-fall	0.25	0.2	0.18	0.12	0.38
Azoxystrobin	reference	2018-summer	0.23	0.31	0.11	0.02	0.44
Azoxystrobin	reference	2020-fall	0.64	0.31	0.12	0.43	0.85
Azoxystrobin	reference	2021-fall	0.84	0.59	0.24	0.44	1.24
Azoxystrobin	reference	2021-summer-NE	0.38	0.61	0.33	-0.03	0.79

Azoxystrobin	reference	2021-summer-VH	0.17	0.59	0.32	-0.23	0.57
Azoxystrobin	reference	2021-winter	0.98	0.28	0.1	0.79	1.17
Benzovindiflupyr	reference	2018-summer	1.47	0.31	0.29	1.26	1.68
Benzovindiflupyr	reference	2020-fall	0.55	0.25	0.1	0.38	0.72
Benzovindiflupyr	reference	2021-fall	1.21	0.17	0.07	1.1	1.32
Benzovindiflupyr	reference	2021-summer-NE	1.19	0.39	0.28	0.93	1.45
Benzovindiflupyr	reference	2021-summer-VH	2.22	0.22	0.73	2.07	2.37
Benzovindiflupyr	reference	2021-winter	1.52	0.29	0.29	1.32	1.72
Bixafen	test PPP	2018-summer	1.51	0.33	0.33	1.29	1.73
Bromoxynil	reference	2021-fall	-0.43	0.91	0.35	-1.04	0.18
Bromoxynil	reference	2021-summer-NE	-0.92	0.33	0.24	-1.14	-0.7
Bromoxynil	reference	2021-summer-VH	-0.36	0.31	0.18	-0.57	-0.15
Budesonide	test API	2021-fall	-0.25	0.79	0.32	-0.78	0.28
Canagliflozin	test API	2021-fall	-0.05	0.73	0.28	-0.54	0.44
Carbendazim	test PPP	2018-summer	1.24	0.59	0.3	0.84	1.64
Carbetamide	test PPP	2018-summer	0.71	0.3	0.11	0.51	0.91
Ceritinib	test API	2021-fall	2.23	0.23	0.72	2.07	2.39
Chlorantraniliprole	test PPP	2018-summer	1.33	0.3	0.24	1.13	1.53
Clomazone	test PPP	2018-summer	0.74	0.57	0.22	0.36	1.12
Clopidogrel	test API	2021-fall	0.75	0.43	0.16	0.46	1.04
Clothianidin	test PPP	2018-summer	2.06	0.22	0.68	1.91	2.21
Clotrimazol	test API	2021-fall	1.13	0.43	0.3	0.84	1.42
Cyantraniliprole	reference	2018-summer	0.75	0.2	0.08	0.62	0.88
Cyantraniliprole	reference	2020-fall	2.18	0.22	0.7	2.03	2.33
Cyantraniliprole	reference	2021-fall	1.55	0.69	0.38	1.08	2.02
Cyantraniliprole	reference	2021-summer-NE	2.12	0.21	0.68	1.98	2.26
Cyantraniliprole	reference	2021-summer-VH	1.31	0.24	0.19	1.15	1.47
Cyantraniliprole	reference	2021-winter	1	0.49	0.18	0.67	1.33
Cyclaniliprole	reference	2018-summer	1.14	0.29	0.15	0.94	1.34
Cyclaniliprole	reference	2020-fall	2.11	0.21	0.71	1.97	2.25
Cyclaniliprole	reference	2021-fall	2.23	0.22	0.71	2.08	2.38
Cyclaniliprole	reference	2021-summer-NE	2.16	0.23	0.69	2	2.32
Cyclaniliprole	reference	2021-summer-VH	2.16	0.23	0.7	2	2.32
Cyclaniliprole	reference	2021-winter	2.11	0.22	0.7	1.96	2.26
Dapagliflozin	test API	2021-fall	-0.68	0.87	0.38	-1.27	-0.09
Dasatinib	test API	2021-fall	-0.43	0.64	0.26	-0.86	0
Dicamba	reference	2018-summer	0.78	0.22	0.08	0.63	0.93
Dicamba	reference	2021-fall	1.13	0.55	0.27	0.76	1.5
Dicamba	reference	2021-summer-NE	0.77	0.22	0.14	0.62	0.92
Dicamba	reference	2021-summer-VH	2.17	0.23	0.68	2.01	2.33
Dienogest	test API	2021-fall	-0.9	0.64	0.29	-1.33	-0.47
Dimethenamid	test PPP	2018-summer	0.44	0.53	0.19	0.08	0.8
Dimoxystrobin	test PPP	2018-summer	1.26	0.44	0.29	0.96	1.56
Diuron	reference	2018-summer	0.81	0.54	0.21	0.45	1.17
Diuron	reference	2020-fall	1.38	0.64	0.39	0.95	1.81
Diuron	reference	2021-fall	1.26	0.49	0.23	0.93	1.59
Diuron	reference	2021-summer-NE	0.68	0.25	0.14	0.51	0.85
Diuron	reference	2021-summer-VH	0.73	0.24	0.14	0.57	0.89
Diuron	reference	2021-winter	1.24	0.35	0.18	1	1.48
Dolutegravir	test API	2021-fall	1.5	0.41	0.29	1.22	1.78
Duloxetine	test API	2021-fall	2.24	0.23	0.69	2.08	2.4
Efavirenz	test API	2021-fall	0.97	0.24	0.09	0.81	1.13
Ezetimibe	test API	2021-fall	0.67	0.49	0.2	0.34	1
Fenhexamid	reference	2018-summer	0.22	0.41	0.15	-0.06	0.5
Fenhexamid	reference	2020-fall	0.21	0.39	0.15	-0.05	0.47
Fenhexamid	reference	2021-fall	0.52	0.53	0.19	0.16	0.88
Fenhexamid	reference	2021-summer-NE	-0.17	0.2	0.12	-0.3	-0.04
Fenhexamid	reference	2021-summer-VH	0.03	0.19	0.11	-0.1	0.16
Fenhexamid	reference	2021-winter	0.38	0.31	0.1	0.17	0.59
Fenoxycarb	reference	2018-summer	-1.99	0.23	0.72	-2.15	-1.83
Fenoxycarb	reference	2020-fall	-1.98	0.22	0.72	-2.13	-1.83
Fenoxycarb	reference	2021-fall	-1.96	0.22	0.73	-2.11	-1.81

Fenoxycarb	reference	2021-summer-NE	-1.75	0.23	0.75	-1.91	-1.59
Fenoxycarb	reference	2021-summer-VH	-1.86	0.23	0.74	-2.02	-1.7
Fenoxycarb	reference	2021-winter	-2.02	0.23	0.73	-2.18	-1.86
Fexofenadine	test API	2021-fall	1.1	0.32	0.13	0.88	1.32
Fingolimod	test API	2021-fall	-1.91	0.23	0.7	-2.07	-1.75
Fipronil	reference	2018-summer	0.95	0.16	0.06	0.84	1.06
Fipronil	reference	2020-fall	2.15	0.22	0.72	2	2.3
Fipronil	reference	2021-fall	1.1	0.25	0.1	0.93	1.27
Fipronil	reference	2021-summer-NE	1.17	0.39	0.26	0.91	1.43
Fipronil	reference	2021-summer-VH	1.16	0.38	0.27	0.9	1.42
Fipronil	reference	2021-winter	2.17	0.22	0.74	2.02	2.32
Flonicamid	test PPP	2018-summer	1.04	0.34	0.15	0.81	1.27
Florasulam	reference	2018-summer	1.44	0.23	0.25	1.28	1.6
Florasulam	reference	2020-fall	1.19	0.45	0.24	0.89	1.49
Florasulam	reference	2021-fall	1.22	0.31	0.17	1.01	1.43
Florasulam	reference	2021-summer-NE	0.89	0.17	0.1	0.78	1
Florasulam	reference	2021-summer-VH	2.19	0.23	0.7	2.03	2.35
Florasulam	reference	2021-winter	1.04	0.32	0.12	0.82	1.26
Fluopicolide	test PPP	2018-summer	2.08	0.21	0.67	1.94	2.22
Fluopyram	reference	2018-summer	1.22	0.32	0.2	1	1.44
Fluopyram	reference	2020-fall	2.15	0.23	0.69	1.99	2.31
Fluopyram	reference	2021-fall	1.45	0.25	0.19	1.28	1.62
Fluopyram	reference	2021-summer-NE	1.26	0.27	0.2	1.08	1.44
Fluopyram	reference	2021-summer-VH	2.15	0.23	0.7	1.99	2.31
Fluopyram	reference	2021-winter	1.49	0.26	0.29	1.31	1.67
Flupyradifurone	reference	2018-summer	1.22	0.47	0.25	0.9	1.54
Flupyradifurone	reference	2020-fall	1.41	0.26	0.27	1.23	1.59
Flupyradifurone	reference	2021-fall	1.48	0.36	0.25	1.24	1.72
Flupyradifurone	reference	2021-summer-NE	1.22	0.32	0.24	1	1.44
Flupyradifurone	reference	2021-summer-VH	1.28	0.26	0.2	1.1	1.46
Flupyradifurone	reference	2021-winter	1.25	0.56	0.25	0.87	1.63
Flutianil	test PPP	2018-summer	1.14	0.3	0.19	0.94	1.34
Fluxapyroxad	test PPP	2018-summer	1.21	0.26	0.15	1.03	1.39
Hydrochlorothiazide	test API	2021-fall	1.46	0.22	0.17	1.31	1.61
Imidacloprid	reference	2018-summer	1.22	0.24	0.15	1.06	1.38
Imidacloprid	reference	2020-fall	1.19	0.35	0.21	0.95	1.43
Imidacloprid	reference	2021-fall	2.22	0.23	0.69	2.06	2.38
Imidacloprid	reference	2021-summer-NE	1.26	0.28	0.21	1.07	1.45
Imidacloprid	reference	2021-summer-VH	1.17	0.38	0.27	0.91	1.43
Imidacloprid	reference	2021-winter	1.23	0.33	0.16	1.01	1.45
Iprovalicarb	test PPP	2018-summer	-0.29	0.85	0.32	-0.86	0.28
Irbesartan	test API	2021-fall	0.87	0.41	0.17	0.59	1.15
Isofetamid	test PPP	2018-summer	0.65	0.55	0.24	0.28	1.02
Isoproturon	reference	2018-summer	0.71	0.61	0.24	0.3	1.12
Isoproturon	reference	2020-fall	0.74	0.47	0.17	0.42	1.06
Isoproturon	reference	2021-fall	1.08	0.67	0.3	0.63	1.53
Isoproturon	reference	2021-summer-NE	0.17	0.25	0.14	0	0.34
Isoproturon	reference	2021-summer-VH	0.34	0.25	0.15	0.17	0.51
Isoproturon	reference	2021-winter	0.93	0.42	0.15	0.65	1.21
Isopyrazam	test PPP	2018-summer	0.98	0.19	0.08	0.85	1.11
Keto-desogestrel	test API	2021-fall	-1.3	0.27	0.26	-1.48	-1.12
Kresoxim-methyl	reference	2018-summer	-1.1	0.45	0.25	-1.4	-0.8
Kresoxim-methyl	reference	2021-fall	-0.78	0.76	0.31	-1.29	-0.27
Lumiracoxib	test API	2021-fall	0.91	0.64	0.27	0.48	1.34
Mandipropamid	reference	2018-summer	-0.51	0.69	0.27	-0.98	-0.04
Mandipropamid	reference	2020-fall	-0.26	0.64	0.25	-0.69	0.17
Mandipropamid	reference	2021-fall	-0.36	0.8	0.31	-0.9	0.18
Mandipropamid	reference	2021-summer-NE	-0.76	0.21	0.12	-0.9	-0.62
Mandipropamid	reference	2021-summer-VH	-0.49	0.2	0.12	-0.62	-0.36
Mandipropamid	reference	2021-winter	-0.11	0.47	0.15	-0.43	0.21
Mesosulfuron-methyl	test PPP	2018-summer	1.46	0.29	0.31	1.26	1.66
Mesotrione	reference	2018-summer	1.22	0.62	0.32	0.8	1.64

Mesotrione	reference	2020-fall	0.9	0.66	0.3	0.45	1.35
Mesotrione	reference	2021-fall	0.74	0.65	0.26	0.3	1.18
Mesotrione	reference	2021-summer-NE	0.23	0.19	0.12	0.1	0.36
Mesotrione	reference	2021-summer-VH	0.82	0.17	0.1	0.71	0.93
Mesotrione	reference	2021-winter	0.85	0.62	0.22	0.43	1.27
Metformin	test API	2021-fall	-0.71	0.81	0.35	-1.26	-0.16
Mirtazapine	test API	2021-fall	1.62	0.3	0.31	1.42	1.82
Mometasone	test API	2021-fall	1.04	0.21	0.19	0.9	1.18
Naloxegol	test API	2021-fall	0.62	0.83	0.34	0.06	1.18
Napropamide	test PPP	2018-summer	0.85	0.46	0.18	0.54	1.16
Olanzepine	test API	2021-fall	-2	0.23	0.75	-2.16	-1.84
Omeprazole	test API	2021-fall	0.19	0.55	0.2	-0.18	0.56
Oxathiapiprolin	reference	2018-summer	-0.37	0.73	0.29	-0.86	0.12
Oxathiapiprolin	reference	2020-fall	-0.06	0.36	0.14	-0.3	0.18
Oxathiapiprolin	reference	2021-fall	-0.09	0.54	0.2	-0.45	0.27
Oxathiapiprolin	reference	2021-summer-NE	-0.53	0.25	0.15	-0.7	-0.36
Oxathiapiprolin	reference	2021-summer-VH	-0.36	0.24	0.14	-0.52	-0.2
Oxathiapiprolin	reference	2021-winter	-0.14	0.7	0.23	-0.61	0.33
Panobinostat	test API	2021-fall	-1.14	0.48	0.38	-1.46	-0.82
Pemetrexed	test API	2021-fall	-2.03	0.24	0.78	-2.19	-1.87
Picoxystrobin	test PPP	2018-summer	-0.25	0.22	0.08	-0.4	-0.1
Pinoxaden	test PPP	2018-summer	-1.11	0.75	0.37	-1.62	-0.6
Pioglitazone	test API	2021-fall	-2.05	0.22	0.75	-2.2	-1.9
Proquinazid	test PPP	2018-summer	-1.12	0.61	0.32	-1.53	-0.71
Pyroxsulam	test PPP	2018-summer	2.14	0.22	0.72	1.99	2.29
Quetiapine	test API	2021-fall	-0.93	0.45	0.22	-1.23	-0.63
Rivastigmin	test API	2021-fall	2.14	0.21	0.69	2	2.28
Rosuvastatin	test API	2021-fall	-0.73	1.16	0.48	-1.51	0.05
Sedaxane	test PPP	2018-summer	1.25	0.35	0.25	1.01	1.49
Spirotetramat	test PPP	2018-summer	-1.11	0.39	0.24	-1.37	-0.85
Sulfoxaflor	test PPP	2018-summer	0.84	0.62	0.29	0.42	1.26
Tadalafil	test API	2021-fall	0.69	0.6	0.23	0.29	1.09
Tembotrione	test PPP	2018-summer	1.22	0.62	0.32	0.8	1.64
Terbinafine	test API	2021-fall	-0.28	1.56	0.61	-1.33	0.77
Terbuthylazine	reference	2018-summer	1.23	0.39	0.23	0.97	1.49
Terbuthylazine	reference	2020-fall	1.31	0.21	0.16	1.17	1.45
Terbuthylazine	reference	2021-fall	1.46	0.28	0.21	1.27	1.65
Terbuthylazine	reference	2021-summer-NE	1.32	0.23	0.18	1.16	1.48
Terbuthylazine	reference	2021-summer-VH	1.21	0.33	0.23	0.99	1.43
Terbuthylazine	reference	2021-winter	2.1	0.22	0.69	1.95	2.25
Ticagrelor	test API	2021-fall	-0.52	1.12	0.46	-1.28	0.24
Topramezone	reference	2018-summer	0.81	0.21	0.08	0.67	0.95
Topramezone	reference	2020-fall	1.49	0.35	0.35	1.25	1.73
Topramezone	reference	2021-summer-NE	1.03	0.2	0.12	0.9	1.16
Topramezone	reference	2021-summer-VH	2.14	0.23	0.67	1.98	2.3
Topramezone	reference	2021-winter	2.17	0.22	0.72	2.02	2.32
Trinexapac-ethyl	test PPP	2018-summer	-0.39	0.75	0.29	-0.9	0.12
Valifenalate	test PPP	2018-summer	-1.11	0.76	0.37	-1.62	-0.6
Valsartan	test API	2021-fall	-0.6	0.96	0.42	-1.25	0.05
Vildagliptin	test API	2021-fall	1.26	0.34	0.16	1.03	1.49
Vorinostat	test API	2021-fall	-1.08	0.64	0.33	-1.51	-0.65

S5.5 Reference $DegT50_{sludge}$ and correlation across experiments

Pearson and Spearman correlations were calculated using the *cor* function in R with complete pairwise observations to compare the Bayesian means of the $logDegT50_{sludge}$ in sludge of each experiment. The experiments in 2018-summer, 2020-fall, 2021-winter and 2021-fall were

performed with activated sludge from WWTP Neugut. However, in experiment 2021-summer, different means were calculated for activated sludge from WWTP Neugut (NE) and Eawag Versuchshalle (VH).

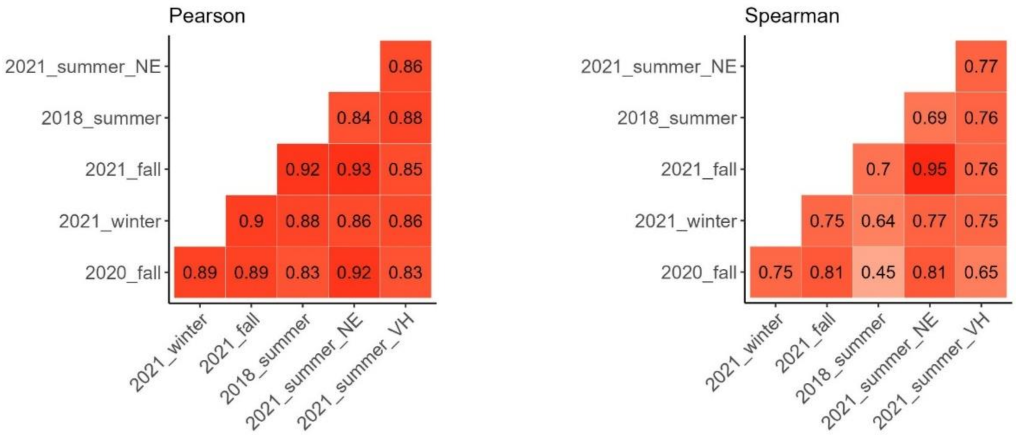

Figure S70. Correlation matrix (Pearson and Spearman) of the mean logDegT50$_{sludge}$ of reference compounds in different experiments with sludge.

Regression models

The next tables show the performance parameters of all models fitted. The model type column indicates if the performance was calculated:

- Before outlier removal: calibrated model with all available reference compounds before removing outliers. Compounds that were not available from the reference set are listed in the column Remarks as "n.a.".
- Final model: model calibrated with reference compounds after removing reference outliers. Outliers are listed in the column Remarks.
- LOOCV: R^2 and RMSE only of the cross validation (using leave-one -out method)
- Prediction test set: R^2 and RMSE of the DT50 predictions for the test set of compounds. Compounds in the test set for which predictions had residuals> 2*RMSE are written in the column Remarks. Number of compounds for the prediction test set are those remaining after outlier removal. RMSE and R2 are given for the remaining test set compounds after outlier removal.

S5.6 Prediction of OECD 307

Table S9. Fit and performance parameters of regression models to predict soil half-lives (OECD 307) from $DegT50_{sludge}$ data – for PPPs from experiment 2018-summer data, for APIs from experiment 2021-fall data, as well as regression models fitted with data from experiments 2020-summer and 2021-winter for calibration substances only.

Experiment for DT50 sludge	log Koc	Model type	RMSE	R2	Model p-value	Intercept	coefficient log $DegT50_{sludge}$	coefficient log Koc or P	number of compounds	Remarks
2018-summer	Koc_OECD106	before outlier removal	0.599	62.0%	0.0003	-1.0994	0.8597	0.8480	20	n.a.=1: Bro
		final model	0.423	76.4%	0.0000	-1.0000	0.8186	0.8576	19	outliers reference compounds = Fnh
		LOOCV	0.519	65.7%	NA	NA	NA	NA	NA	NA
		prediction test set PPPs	0.757	68.2%	NA	NA	NA	NA	26	outliers test set= Flt, Pyr, Sul, Tri, Vlf
	Koc_opera	before outlier removal	0.705	47.5%	0.0042	-0.0816	0.7658	0.4083	20	n.a.=1: Bro
		final model	0.519	76.5%	0.0002	-0.1639	0.7457	0.4803	19	outliers reference compounds = Fnh
		LOOCV	0.606	53.3%	NA	NA	NA	NA	NA	NA
		prediction test set PPPs	0.893	45.0%	NA	NA	NA	NA	26	outliers test set = Fln, Flor, Pro, Pyr, Sul, Tri
	P_OECD107	before outlier removal	0.750	40.6%	0.0120	0.7157	0.7462	0.1856	20	n.a.=1: Bro

		final model	0.592	66.1%	0.0021	0.7476	0.7311	0.2270	19	outliers reference compounds = Fnh
		LOOCV	0.729	32.8%	NA	NA	NA	NA	NA	NA
		prediction test set PPPs	0.903	57.7%	NA	NA	NA	NA	24	outliers test set = Fln, Pyr, Sul, Top, Tri
	P_ ChemAxon	before outlier removal	0.809	30.7%	0.0442	0.9833	0.6368	0.0659	20	n.a.=1: Bro
		final model	0.630	47.7%	0.0056	0.5150	0.7049	0.2627	19	outliers reference compounds = Fnh
		LOOCV	0.745	29.2%	NA	NA	NA	NA	NA	NA
		prediction test set PPPs	0.817	68.7%	NA	NA	NA	NA	26	outliers test set = Sul, Tri, Vlf
2021-fall	Koc_OECD106	before outlier removal	0.545	70.9%	0.0000	-1.3219	0.8131	0.8254	20	n.a.=1: Top
		final model	0.353	89.6%	0.0000	-1.2498	0.7910	0.8421	19	outliers reference compounds = Fnh
		LOOCV	0.409	80.7%	NA	NA	NA	NA	NA	NA
		prediction test set APIs	2.038	80.3%	NA	NA	NA	NA	4	outliers test set = Atv, Ceri, Man, Mir, Pan
	Koc_opera	before outlier removal	0.660	57.2%	0.0007	-0.3154	0.7364	0.3963	20	n.a.=1: Top
		final model	0.470	74.4%	0.0000	-0.4057	0.7254	0.4683	19	outliers reference compounds = Fnh
		LOOCV	0.590	62.9%	NA	NA	NA	NA	NA	NA
		prediction test set APIs	1.264	52.6%	NA	NA	NA	NA	4	outliers test set = Atv, Ceri
	P_OECD107	before outlier removal	0.635	60.5%	0.0004	0.2282	0.7568	0.2682	20	n.a.=1: Top
		final model	0.419	83.3%	0.0000	0.2421	0.7484	0.3161	19	outliers reference compounds = Fnh
		LOOCV	0.484	72.9%	NA	NA	NA	NA	NA	NA
		prediction test set APIs	1.019	100.0%	NA	NA	NA	NA	2	outliers test set = Ceri, Kre
	P_ ChemAxon	before outlier removal	0.752	44.5%	0.0067	0.4107	0.7103	0.1489	20	n.a.=1: Top
		final model	0.501	70.9%	0.0001	-0.2515	0.7946	0.3964	19	outliers reference compounds = Fnh
		LOOCV	0.588	60.9%	NA	NA	NA	NA	NA	NA
		prediction test set APIs	1.808	31.1%	NA	NA	NA	NA	5	outliers test set = Atv, Ceri, Dol, Pan
2020-fall	Koc_OECD106	before outlier removal	0.5691	59.6%	0.0011	-1.027	0.702	0.781	18	n.a.=3: Brol, Dic, Kre
		final model	0.3853	72.7%	0.0001	-0.740	0.615	0.743	17	outliers reference compounds = Fnh

		LOOCV	0.4903	56.3%	NA	NA	NA	NA	NA	NA
	Koc_opera	before outlier removal	0.6454	48.0%	0.0074	0.031	0.626	0.356	18	n.a.=3: Bro, Dic, Kre
		final model	0.4269	74.4%	0.0005	0.064	0.556	0.409	17	outliers reference compounds = Fnh
		LOOCV	0.4983	55.7%	NA	NA	NA	NA	NA	NA
	P_ ChemAxon	before outlier removal	0.7367	32.2%	0.0541	1.075	0.518	0.039	18	n.a.=3: Bro, Dic, Kre
		final model	0.5280	48.7%	0.0093	0.740	0.507	0.209	17	outliers reference compounds = Fnh
		LOOCV	0.6581	23.1%	NA	NA	NA	NA	NA	NA
	P_OECD107	before outlier removal	0.7054	37.9%	0.0282	0.834	0.566	0.136	18	n.a.=3: Bro, Dic, Kre
		final model	0.5266	49.0%	0.0089	0.931	0.498	0.175	17	outliers reference compounds = Fnh
		LOOCV	0.7078	15.4%	NA	NA	NA	NA	NA	NA
2021-winter	Koc_OECD106	before outlier removal	0.5263	65.4%	0.0003	-0.847	0.726	0.689	18	n.a.=3: Bro, Dic, Kre
		final model	0.2944	84.1%	0.0000	-0.620	0.654	0.672	17	outliers reference compounds = Fnh
		LOOCV	0.3509	77.5%	NA	NA	NA	NA	NA	NA
	Koc_opera	before outlier removal	0.6078	53.9%	0.0030	0.158	0.654	0.293	18	n.a.=3: Bro, Dic, Kre
		final model	0.3606	84.4%	0.0000	0.159	0.592	0.356	17	outliers reference compounds = Fnh
		LOOCV	0.4096	70.0%	NA	NA	NA	NA	NA	NA
	P_ ChemAxon	before outlier removal	0.6788	42.5%	0.0158	0.979	0.599	0.035	18	n.a.=3: Bro, Dic, Kre
		final model	0.4382	70.3%	0.0007	0.641	0.589	0.206	17	outliers reference compounds = Fnh
		LOOCV	0.5213	50.4%	NA	NA	NA	NA	NA	NA
	P_OECD107	before outlier removal	0.6411	48.7%	0.0067	0.712	0.649	0.140	18	n.a.=3: Bro, Dic, Kre
		final model	0.4264	66.6%	0.0005	0.794	0.593	0.183	17	outliers reference compounds = Fnh
		LOOCV	0.5398	47.2%	NA	NA	NA	NA	NA	NA

S5.7 Prediction of OECD 308

Table S10. Fit and performance parameters of regression models to predict water-sediment half-lives (OECD 308) from $DegT50_{sludge}$ – for PPPs from experiment 2018-summer data, for APIs from experiment 2021-fall data, as well as regression models fitted with data from experiments 2020-summer and 2021-winter for calibration substances only.

Experiment for DT50 sludge	log Koc	Model type	RMSE	R2	Model p-value	Intercept	coefficient logDegT50 sludge	coefficient logKoc	number of compounds	Remarks
2018-summer	Koc_OECD106	before outlier removal	0.480	58.1%	0.0006	0.0780	0.6739	0.4971	20	n.a.=1: Bro
		final model	0.480	58.1%	0.0006	0.0780	0.6739	0.4971	20	outliers reference compounds = none
		LOOCV	0.558	45.5%	NA	NA	NA	NA	NA	NA
		prediction test set	0.527	64.5%	NA	NA	NA	NA	20	outliers test predicted = Ipr
	Koc_opera	before outlier removal	0.503	53.9%	0.0014	0.5307	0.6382	0.2853	20	n.a.=1: Bro
		final model	0.503	53.9%	0.0014	0.5307	0.6382	0.2853	20	outliers reference compounds = none
		LOOCV	0.612	37.4%	NA	NA	NA	NA	NA	NA
		prediction test set	0.547	67.8%	NA	NA	NA	NA	20	outliers test predicted = Pro
	P_OECD107	before outlier removal	0.524	50.0%	0.0028	1.0537	0.6353	0.1428	20	n.a.=1: Bro
		final model	0.524	50.0%	0.0028	1.0537	0.6353	0.1428	20	outliers reference compounds = none
		LOOCV	0.618	33.8%	NA	NA	NA	NA	NA	NA
		prediction test set	0.507	65.9%	NA	NA	NA	NA	18	outliers test predicted = none
	P_ ChemAxon	before outlier removal	0.554	44.1%	0.0072	1.0346	0.5963	0.1199	20	n.a.=1: Bro
		final model	0.554	44.1%	0.0072	1.0346	0.5963	0.1199	20	outliers reference compounds = none
		LOOCV	0.658	25.7%	NA	NA	NA	NA	NA	NA
		prediction test set	0.545	64.4%	NA	NA	NA	NA	20	outliers test predicted = none
2021-fall	Koc_OECD106	before outlier removal	0.462	62.2%	0.0003	0.0764	0.6001	0.4429	20	n.a.=1: Top
		final model	0.399	76.0%	0.0000	0.1011	0.6425	0.4428	19	outliers reference compounds = Cya
		LOOCV	0.474	61.6%	NA	NA	NA	NA	NA	NA

		prediction test set	1.106	40.4%	NA	NA	NA	NA	15	outliers test set = Aca, Atv, Ceri, Clp, Eze, Fluo, Mir, Pan
	Koc_opera	before outlier removal	0.488	57.8%	0.0007	0.5188	0.5669	0.2440	20	n.a.=1: Top
		final model	0.488	57.8%	0.0007	0.5188	0.5669	0.2440	20	outliers reference compounds = none
		LOOCV	0.581	44.1%	NA	NA	NA	NA	NA	NA
		prediction test set	0.700	39.2%	NA	NA	NA	NA	30	outliers test set = Atv, Dap, Efa
	P_OECD107	before outlier removal	0.489	57.7%	0.0007	0.8925	0.5726	0.1501	20	n.a.=1: Top
		final model	0.428	67.7%	0.0001	0.9092	0.6174	0.1532	19	outliers reference compounds = Cya
		LOOCV	0.513	55.4%	NA	NA	NA	NA	NA	NA
		prediction test set	0.575	50.1%	NA	NA	NA	NA	23	outliers test set = Clp, Dap, Fin
	P_ ChemAxon	before outlier removal	0.513	53.3%	0.0015	0.7628	0.5807	0.1513	20	n.a.=1: Top
		final model	0.513	53.3%	0.0015	0.7628	0.5807	0.1513	20	outliers reference compounds = none
		LOOCV	0.618	36.1%	NA	NA	NA	NA	NA	NA
		prediction test set	0.685	46.7%	NA	NA	NA	NA	33	outliers test set = Atv, Dap, Dol, Met
	Koc_OECD106	before outlier removal	0.5204	40.9%	0.0194	0.233	0.452	0.440	18	n.a.=3: Bro, Dic, Kre
		final model	0.4201	60.1%	0.0016	0.164	0.554	0.460	17	outliers reference compounds = Cya
		LOOCV	0.5136	43.2%	NA	NA	NA	NA	NA	NA
	Koc_opera	before outlier removal	0.5281	39.1%	0.0242	0.675	0.426	0.248	18	n.a.=3: Bro, Dic, Kre
		final model	0.5281	39.1%	0.0242	0.675	0.426	0.248	18	outliers reference compounds = none
		LOOCV	0.6156	22.2%	NA	NA	NA	NA	NA	NA
	P_OECD107	before outlier removal	0.5729	28.3%	0.0822	1.228	0.379	0.078	18	n.a.=3: Bro, Dic, Kre
		final model	0.5729	28.3%	0.0822	1.228	0.379	0.078	18	outliers reference compounds = none
		LOOCV	0.6702	8.0%	NA	NA	NA	NA	NA	NA
	P_ ChemAxon	before outlier removal	0.5455	35.0%	0.0393	1.137	0.402	0.129	18	n.a.=3: Bro, Dic, Kre
		final model	0.4508	54.1%	0.0043	1.094	0.505	0.141	17	outliers reference compounds = Cya
2020-fall		LOOCV	0.5427	36.4%	NA	NA	NA	NA	NA	NA

2021-winter	Koc_OECD106	before outlier removal	0.4677	52.3%	0.0039	0.264	0.505	0.399	18	n.a.=3: Bro, Dic, Kre
		final model	0.5319	40.1%	NA	NA	NA	NA	NA	NA
		LOOCV	0.4677	52.3%	0.0039	0.264	0.505	0.399	18	outliers reference compounds = none
	Koc_opera	before outlier removal	0.4824	49.2%	0.0062	0.715	0.473	0.211	18	n.a.=3: Bro, Dic, Kre
		final model	0.5676	35.1%	NA	NA	NA	NA	NA	NA
		LOOCV	0.4824	49.2%	0.0062	0.715	0.473	0.211	18	outliers reference compounds = none
	P_OECD107	before outlier removal	0.5169	41.7%	0.0175	1.125	0.459	0.079	18	n.a.=3: Bro, Dic, Kre
		final model	0.5169	41.7%	0.0175	1.125	0.459	0.079	18	outliers reference compounds = none
		LOOCV	0.6067	24.8%	NA	NA	NA	NA	NA	NA
	P_ ChemAxon	before outlier removal	0.4812	49.4%	0.0060	1.014	0.487	0.136	18	n.a.=3: Bro, Dic, Kre
		final model	0.4812	49.4%	0.0060	1.014	0.487	0.136	18	outliers reference compounds= none
		LOOCV	0.5552	35.4%	NA	NA	NA	NA	NA	NA

S5.8 Comparison to other predictions of DT50s

Predicted environmental degradation half-lives (DT50s) of reference compounds, test APIs and test PPPs were calculated with the publicly available *in-silico* models OPERA-Biodeg [4], VEGA [13] and EPI Suite ™ BIOWIN4 (primary biodegradation). [14] The output of BIOWIN was transformed into log(DT50s) by using the equation suggested by [15] :

$$\log(DT50) = -1.46 * x_{biowin4} + 6.51$$

Similarly to the Koc calculations, we were not able to obtain OPERA predictions of DT50 for aliskiren, dolutegravir, pemetrexed, pinoxaden and ridaforolimus. Only pinoxaden did not get a predicted DT50 from VEGA. All compounds were plotted against their measured OECD 307 or OECD 308 DT50.

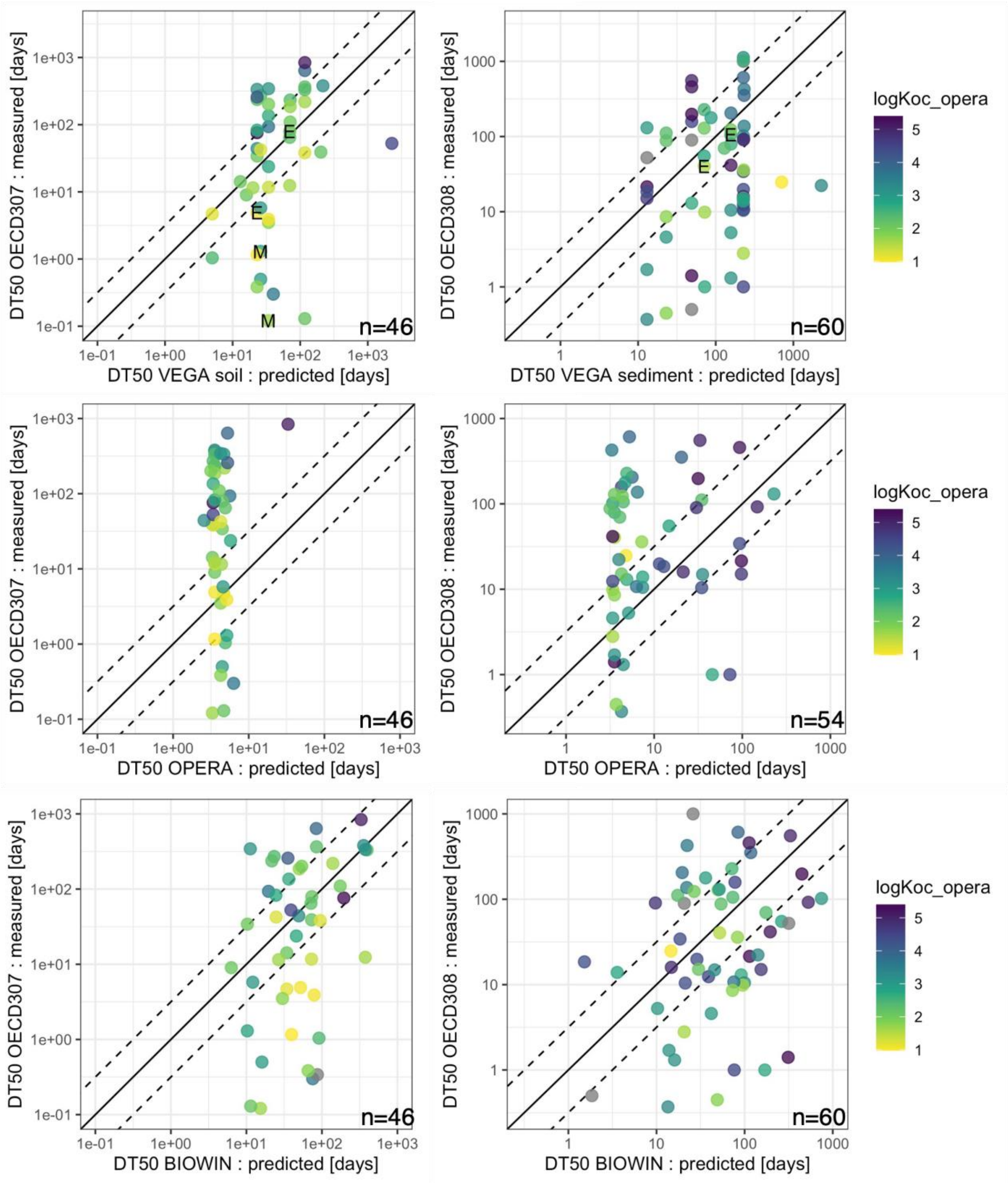

Figure S71. Comparison of experimental DT50s from OECD 307 and OECD 308 to the predicted DT50s with OPERA tool and VEGA models for soil and sediment. Compounds for which VEGA provided experimental DT50s are labelled with an "E", and VEGA predictions with moderate reliability are indicated with a "M". All other predicted DT50s have a low reliability according to VEGA.

The models acknowledge that performance might not be good for prediction of half-lives of all chemicals and provide an estimation of the limits of applicability. OPERA is applicable to hydrocarbons only, and predictions were marked outside the global applicability domain for 90 chemicals in our study (out of 93). Also, the local applicability domain index was also low,

ranging between 0.1 and 0.5 (the minimum value of 0, maximum value of 1). Likewise, VEGA marked the predictions as "low reliability" based on the applicability domain index, except for trinexepac-ethyl and fenoxycarb, which were labelled "moderate reliability" for sediment predictions only, and dicamba and diuron which had the label "experimental value" in both soil and sediment predictions. BIOWIN4 provided predictions for all compounds and did not indicate limitations regarding its applicability domain.

Correlations between measured and predicted values are shown in Figure S72 below.

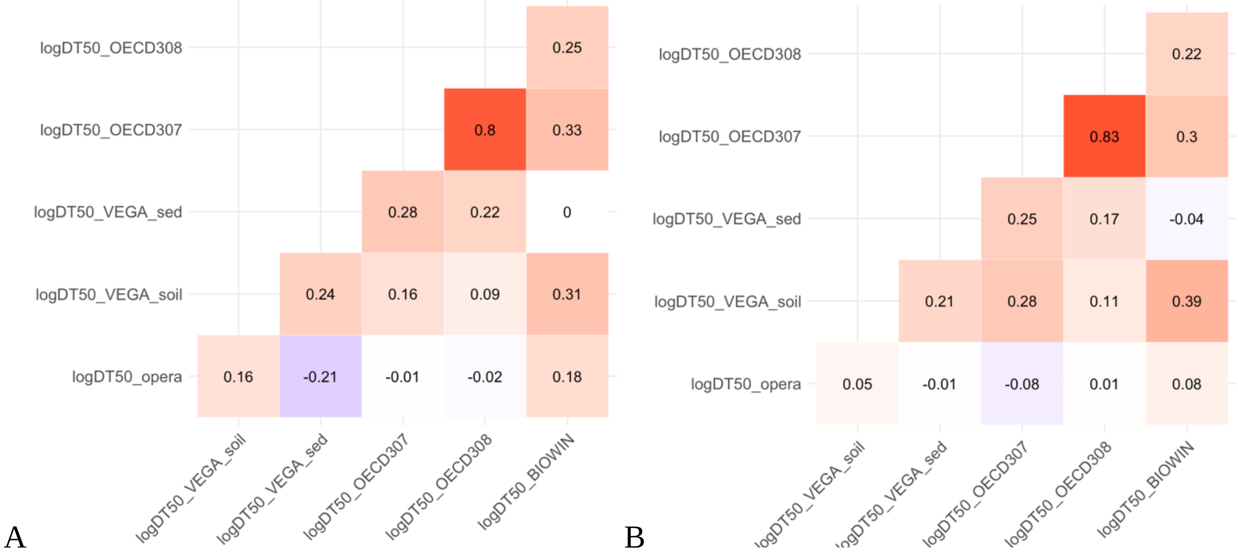

Figure S72. A) Pearson and B) Spearman correlations between measured logDT50s from OECD 307 and OECD 308 and the predicted logDT50s from VEGA soil and sediment models and OPERA Biodeg model.

Number of common DT50 values in each comparison: opera x VEGA_soil: 89, opera x VEGA_sed: 89, opera x OECD307: 54, opera x OECD308: 76, VEGA_soil x VEGA_sed: 93, VEGA_soil x OECD307: 55, VEGA_soil x OECD308: 80, VEGA_sed x OECD307: 55, VEGA_sed x OECD308: 80, OECD307 x OECD308: 48, BIOWIN x opera: 89, BIOWIN x VEGA_soil: 93, BIOWIN x VEGA_sed: 93, BIOWIN x OECD307: 56, BIOWIN x OECD308: 81

S5.9 DT50 vs logKoc chemical space

Below are plots of the OECD 307/ 308 DT50s vs the Koc (from OECD 106 or predicted by OPERA) to assess the chemical space covered by reference compounds, test APIs and test PPPs. Reference compounds were selected to cover the characteristic range of DT50 from OECD 307 and of the Koc from OECD 106 for PPPs.

There seems to be a gap of representation (both in reference and test PPPs) for fast degrading compounds (OECD 307 / 308 DT50 < 10 days) with log Koc > 4. Based on the predicted log Koc from OPERA, the Koc range of APIs extends to higher Koc values than for PPPs, i.e., 18 APIs have log Koc > 4 compared to 4 PPPs (from both the test PPP and reference sets). This

confirms that the set of reference compounds might need to be extended to compounds with higher Koc and low half-lives to better cover the chemical space of APIs.

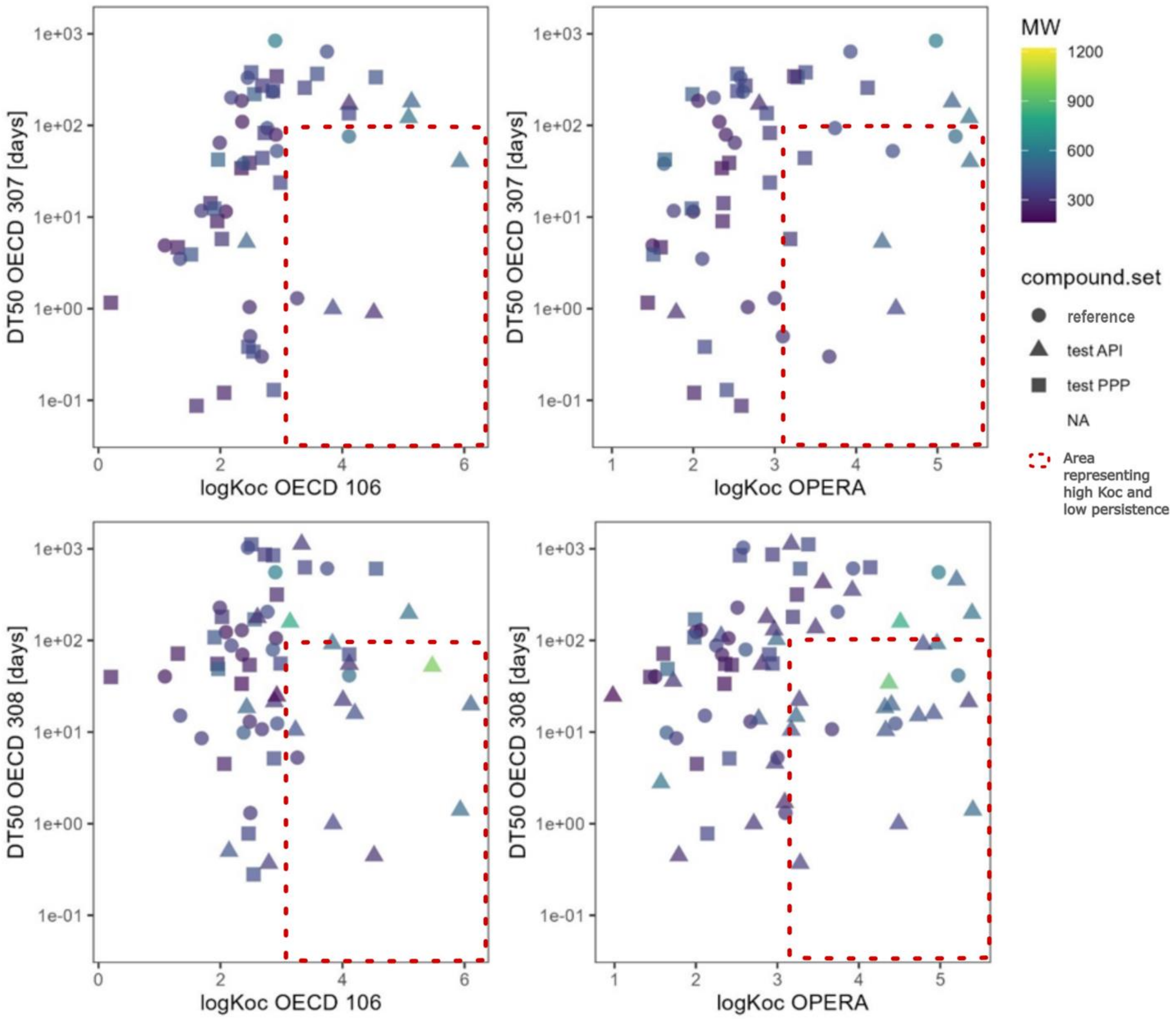

Figure S73. Koc vs DT50 of reference, test APIs and test PPPs

S6. Classification models

S6.1 Equations of the classification models

Several classification models were fitted for each experiment by adjusting three parameters: 1) weighted distance to 1, 2 or 3 neighbors,2) a Koc-coefficient (C_{Koc} = 0.1, 1 and 10) to adjust the importance of the Koc difference compared to differences in $DegT50_{sludge}$ and 3) varying the threshold of classification between 70 days and 100 days.

We calculated the Euclidian distance between each test compound and each of the reference compounds to determine the nearest reference neighbors. A distance vector for each test compound (v_{test}) to each of the reference compounds ($v_{reference}$) was calculated with the differences between the mean and standard deviation (SD) of the posterior distribution of log $DT50_{sludge}$ and log Koc parameter. The Koc-coefficient (C_{Koc}) was applied to the difference between the log Koc's. Afterwards, the Euclidian distance was calculated with the square root of the sum of the square differences.

$$v_{test} = \{\log DT50_{sludge-mean,test}, \log DT50_{sludge-SD,test}, \log K_{OC,test}\}$$

$$v_{reference} = \{\log DT50_{sludge-mean,ref}, \log DT50_{sludge-SD,ref}, \log K_{OC,ref}\}$$

$$distance\_vector = v_{test} - v_{reference}$$

$$distance\_vector = \begin{Bmatrix} \log DT50_{sludge-mean,test} - \log DT50_{sludge-mean,ref}, \\ \log DT50_{sludge-SD,test} - \log DT50_{sludge-SD,ref}, \\ C_{Koc} * (\log K_{OC,test} - \log K_{OC,ref}) \end{Bmatrix}$$

$$Euclidian_{distance} = \sqrt{\sum_{i=1}^{3} (distance\_vector[i])^2}$$

For the performance parameters of the classification models, the positive outcome was defined as "non-persistent" and the negative outcome as the "persistent" and the model performance equations were taken as:

$$accuracy = \frac{true\ positives + true\ negatives}{total\ positives + total\ negatives}$$

$$sensitivity = \frac{true\ positives}{true\ positives + false\ negatives}$$

$$specificity = \frac{true\ negatives}{true\ negatives + false\ positives}$$

$$precision = \frac{true\ positives}{true\ positives + false\ positives}$$

Accuracy assesses the number of correct predictions compared to the total. We interpret sensitivity and precision as the model performance parameters to assess "non-persistent" compounds, and specificity as the performance parameter to assess "persistent" compounds.

S6.2 Prediction of non-persistence in classification models for OECD 307 DT50s

The best overall performance in a classification model was found for models with 1 neighbor, but, for robustness, the results for 2 neighbors are reported in the main text of the paper.

Table S11. Results for classification models for the PPP test set with a threshold of 70 d and 100 d for OECD 307. The model uses data from the 2018-summer experiment and either experimental log Koc from OECD 106, predicted log Koc from OPERA, experimental log P from OECD 107 or log P predicted with ChemAxon

Koc Type	threshold	Number of K-neighbor	Coefficient for Koc	Accuracy	Sensitivity	Specificity	Precision
**Koc OECD 106**	70 days	1	0.1	0.889	0.882	0.900	0.938
			1.0	0.852	0.765	1.000	1.000
			10.0	0.852	0.882	0.800	0.882
		2	0.1	0.482	0.471	0.500	0.615
			1.0	0.852	0.824	0.900	0.933
			10.0	0.704	0.706	0.700	0.800
		3	0.1	0.593	0.353	1.000	1.000
			1.0	0.815	0.765	0.900	0.929
			10.0	0.593	0.529	0.700	0.750
	100 days	1	0.1	0.889	0.944	0.778	0.895
			1.0	0.815	0.833	0.778	0.882
			10.0	0.741	0.889	0.444	0.762
		2	0.1	0.630	0.722	0.444	0.722
			1.0	0.778	0.778	0.778	0.875
			10.0	0.815	0.889	0.667	0.842
		3	0.1	0.704	0.611	0.889	0.917
			1.0	0.778	0.778	0.778	0.875
			10.0	0.482	0.556	0.333	0.625
**Koc OPERA**	70 days	1	0.1	0.731	0.750	0.700	0.800
			1.0	0.769	0.750	0.800	0.857
			10.0	0.423	0.563	0.200	0.529
		2	0.1	0.577	0.500	0.700	0.727
			1.0	0.539	0.625	0.400	0.625
			10.0	0.539	0.563	0.500	0.643
		3	0.1	0.577	0.438	0.800	0.778
			1.0	0.539	0.375	0.800	0.750
			10.0	0.539	0.563	0.500	0.643
	100 days	1	0.1	0.769	0.882	0.556	0.790
			1.0	0.846	0.882	0.778	0.882
			10.0	0.615	0.824	0.222	0.667
		2	0.1	0.692	0.706	0.667	0.800
			1.0	0.654	0.824	0.333	0.700
			10.0	0.577	0.647	0.444	0.688
		3	0.1	0.692	0.647	0.778	0.846
			1.0	0.577	0.471	0.778	0.800
			10.0	0.500	0.588	0.333	0.625
**P OECD 107**	70 days	1	0.1	0.800	0.733	0.900	0.917
			1.0	0.800	0.733	0.900	0.917
			10.0	0.760	0.667	0.900	0.909
		2	0.1	0.640	0.600	0.700	0.750
			1.0	0.720	0.533	1.000	1.000
			10.0	0.480	0.533	0.400	0.571
		3	0.1	0.640	0.467	0.900	0.875
			1.0	0.680	0.533	0.900	0.889
			10.0	0.560	0.467	0.700	0.700
	100 days	1	0.1	0.840	0.875	0.778	0.875

			1.0	0.800	0.813	0.778	0.867
			10.0	0.800	0.750	0.889	0.923
		2	0.1	0.760	0.813	0.667	0.813
			1.0	0.760	0.688	0.889	0.917
			10.0	0.520	0.688	0.222	0.611
		3	0.1	0.720	0.688	0.778	0.846
			1.0	0.720	0.688	0.778	0.846
			10.0	0.640	0.688	0.556	0.733
**P ChemAxon**	70 days	1	0.1	0.852	0.824	0.900	0.933
			1.0	0.778	0.647	1.000	1.000
			10.0	0.741	0.588	1.000	1.000
		2	0.1	0.556	0.471	0.700	0.727
			1.0	0.667	0.529	0.900	0.900
			10.0	0.630	0.588	0.700	0.769
		3	0.1	0.444	0.353	0.600	0.600
			1.0	0.741	0.647	0.900	0.917
			10.0	0.482	0.588	0.300	0.588
	100 days	1	0.1	0.852	0.889	0.778	0.889
			1.0	0.778	0.722	0.889	0.929
			10.0	0.704	0.667	0.778	0.857
		2	0.1	0.741	0.778	0.667	0.824
			1.0	0.667	0.611	0.778	0.846
			10.0	0.593	0.611	0.556	0.733
		3	0.1	0.556	0.611	0.444	0.688
			1.0	0.667	0.722	0.556	0.765
			10.0	0.556	0.667	0.333	0.667

We conducted a more in-depth analysis of the three PPP test compounds misclassified based on sludge data and Koc OECD106 as persistent, while they are actually not persistent in soil (Figure 2A in the main manuscript; carbendazim, dimethenamid, and mesosulfuron-methyl). We analyzed them with respect to their nearest neighbors and their respective initial transformation reactions. For carbendazim, the nearest neighbors are terbuthylazine and fluopyram, which are both persistent in soil (DT50s of 109 and 332 days, respectively). According to the reported transformation products for these three compounds in their registration dossiers (see envipath.org, Package Eawag-Soil), the major transformation pathway for carbendazim in soil is via carbamate hydrolysis, whereas oxidative dealkylation and chlorotriazine hydrolysis, and hydroxylation are reported as initial transformations for terbuthylazine and fluopyram, respectively. For dimethenamid, isoproturon and topramezone are its nearest neighbors. While dimethenamid is predominantly transformed by glutathione conjugation, this pathway is not available for isoproturon and topramezone, but rather mostly oxidative initial transformation reactions are reported for these compounds. Mesosulfuron-methyl has flupyradifurone and imidacloprid with DT50s of 64.7 and 185 days, respectively, as nearest neighbours. Its initial transformation is via ester hydrolysis mainly, whereas flupyradifurone and imidacloprid are transformed via different oxidative reactions. These three examples show that although these compounds are biotransformed to rather similar extents in sludge, and are therefore nearest neighbors according to the classification algorithm, their initial transformation reactions are likely to be very different (i.e., mostly substitution reactions for the outlier compounds, and oxidation reactions for their nearest neighbors). If the relative abundance of the functions (i.e., enzymes) catalyzing those respective transformations differs significantly between soil and sludge, correct classification cannot be achieved. Concretely, this analysis provides some evidence that hydrolytic functions are more abundant relative to oxidative functions in soil than they are in sludge.

S6.3 Prediction of non-persistence in classification models for OECD 308 DT50s

Table S12. Results for classification models of the API test set with a threshold of 70 d and 100 d for OECD 308. The model uses data from the 2021-fall experiment and either experimental log Koc from OECD 106, predicted log Koc from OPERA, experimental log P from OECD 107 or log P predicted with ChemAxon

Koc Type	threshold	Number of K-neighbor	Coefficient for Koc	Accuracy	Sensitivity	Specificity	Precision
**Koc OECD 106**	70 days	1	0.1	0.933	0.909	1.000	1.000
			1.0	0.867	0.818	1.000	1.000
			10.0	0.733	0.818	0.500	0.818
		2	0.1	0.867	0.818	1.000	1.000
			1.0	0.667	0.546	1.000	1.000
			10.0	0.400	0.364	0.500	0.667
		3	0.1	0.600	0.818	0.000	0.692
			1.0	0.867	0.818	1.000	1.000
			10.0	0.600	0.636	0.500	0.778
	100 days	1	0.1	0.800	0.917	0.333	0.846
			1.0	0.800	0.750	1.000	1.000
			10.0	0.667	0.750	0.333	0.818
		2	0.1	0.800	0.750	1.000	1.000
			1.0	0.600	0.667	0.333	0.800
			10.0	0.600	0.583	0.667	0.875
		3	0.1	0.667	0.833	0.000	0.769
			1.0	0.867	0.833	1.000	1.000
			10.0	0.667	0.667	0.667	0.889
**Koc OPERA**	70 days	1	0.1	0.800	0.842	0.727	0.842
			1.0	0.833	0.895	0.727	0.850
			10.0	0.700	0.895	0.364	0.708
		2	0.1	0.800	0.842	0.727	0.842
			1.0	0.767	0.790	0.727	0.833
			10.0	0.500	0.526	0.455	0.625
		3	0.1	0.633	0.790	0.364	0.682
			1.0	0.733	0.895	0.455	0.739
			10.0	0.567	0.790	0.182	0.625
	100 days	1	0.1	0.700	0.762	0.556	0.800
			1.0	0.767	0.857	0.556	0.818
			10.0	0.667	0.857	0.222	0.720
		2	0.1	0.767	0.905	0.444	0.792
			1.0	0.700	0.714	0.667	0.833
			10.0	0.600	0.619	0.556	0.765
		3	0.1	0.667	0.810	0.333	0.739
			1.0	0.767	0.905	0.444	0.792
			10.0	0.633	0.810	0.222	0.708
**P OECD 107**	70 days	1	0.1	0.739	0.800	0.625	0.800
			1.0	0.696	0.733	0.625	0.786
			10.0	0.565	0.467	0.750	0.778
		2	0.1	0.783	0.867	0.625	0.813
			1.0	0.739	0.800	0.625	0.800
			10.0	0.652	0.800	0.375	0.706
		3	0.1	0.609	0.733	0.375	0.688
			1.0	0.696	0.800	0.500	0.750
			10.0	0.304	0.200	0.500	0.429
	100 days	1	0.1	0.609	0.722	0.200	0.765
			1.0	0.609	0.667	0.400	0.800
			10.0	0.478	0.444	0.600	0.800
		2	0.1	0.783	0.833	0.600	0.882

			1.0	0.652	0.778	0.200	0.778
			10.0	0.652	0.778	0.200	0.778
		3	0.1	0.609	0.722	0.200	0.765
			1.0	0.652	0.722	0.400	0.813
			10.0	0.435	0.500	0.200	0.692
**P ChemAxon**	70 days	1	0.1	0.758	0.850	0.615	0.773
			1.0	0.636	0.600	0.692	0.750
			10.0	0.364	0.350	0.385	0.467
		2	0.1	0.758	0.850	0.615	0.773
			1.0	0.485	0.550	0.385	0.579
			10.0	0.485	0.450	0.539	0.600
		3	0.1	0.727	0.800	0.615	0.762
			1.0	0.667	0.800	0.462	0.696
			10.0	0.576	0.600	0.539	0.667
	100 days	1	0.1	0.697	0.870	0.300	0.741
			1.0	0.636	0.609	0.700	0.824
			10.0	0.455	0.478	0.400	0.647
		2	0.1	0.727	0.826	0.500	0.792
			1.0	0.546	0.652	0.300	0.682
			10.0	0.515	0.522	0.500	0.706
		3	0.1	0.636	0.696	0.500	0.762
			1.0	0.667	0.783	0.400	0.750
			10.0	0.576	0.609	0.500	0.737

Table S13. Results for classification models for the PPP test set with a threshold of 70 d and 100 d for OECD 308. The model uses data from the 2018-summer experiment and either exp experimental log Koc from OECD 106, predicted log Koc from OPERA, experimental log P from OECD 107 or logP predicted with ChemAxon

**Koc Type**	**threshold**	**Number of K-neighbor**	**Coefficient for Koc**	**Accuracy**	**Sensitivity**	**Specificity**	**Precision**
**Koc OECD 106**	70 days	1	0.1	0.714	0.700	0.727	0.700
			1.0	0.714	0.700	0.727	0.700
			10.0	0.619	0.600	0.636	0.600
		2	0.1	0.571	0.500	0.636	0.556
			1.0	0.524	0.600	0.455	0.500
			10.0	0.524	0.400	0.636	0.500
		3	0.1	0.667	0.800	0.546	0.615
			1.0	0.667	0.800	0.546	0.615
			10.0	0.476	0.400	0.546	0.444
	100 days	1	0.1	0.667	0.667	0.667	0.727
			1.0	0.857	0.833	0.889	0.909
			10.0	0.667	0.667	0.667	0.727
		2	0.1	0.619	0.583	0.667	0.700
			1.0	0.476	0.667	0.222	0.533
			10.0	0.571	0.500	0.667	0.667
		3	0.1	0.714	0.917	0.444	0.688
			1.0	0.667	0.750	0.556	0.692
			10.0	0.524	0.667	0.333	0.571
**Koc OPERA**	70 days	1	0.1	0.700	0.667	0.727	0.667
			1.0	0.650	0.556	0.727	0.625
			10.0	0.400	0.444	0.364	0.364
		2	0.1	0.550	0.444	0.636	0.500
			1.0	0.600	0.667	0.546	0.546
			10.0	0.450	0.556	0.364	0.417
		3	0.1	0.600	0.889	0.364	0.533
			1.0	0.450	0.333	0.546	0.375

			10.0	0.400	0.444	0.364	0.364
	100 days	1	0.1	0.650	0.636	0.667	0.700
			1.0	0.650	0.636	0.667	0.700
			10.0	0.550	0.636	0.444	0.583
		2	0.1	0.600	0.546	0.667	0.667
			1.0	0.700	0.818	0.556	0.692
			10.0	0.550	0.636	0.444	0.583
		3	0.1	0.600	0.909	0.222	0.588
			1.0	0.550	0.546	0.556	0.600
			10.0	0.500	0.818	0.111	0.529
**P OECD 107**	70 days	1	0.1	0.684	0.500	0.818	0.667
			1.0	0.579	0.500	0.636	0.500
			10.0	0.474	0.250	0.636	0.333
		2	0.1	0.526	0.625	0.455	0.455
			1.0	0.526	0.625	0.455	0.455
			10.0	0.526	0.625	0.455	0.455
		3	0.1	0.579	0.875	0.364	0.500
			1.0	0.526	0.500	0.546	0.444
			10.0	0.368	0.250	0.455	0.250
	100 days	1	0.1	0.632	0.500	0.778	0.714
			1.0	0.684	0.600	0.778	0.750
			10.0	0.526	0.400	0.667	0.571
		2	0.1	0.579	0.700	0.444	0.583
			1.0	0.474	0.600	0.333	0.500
			10.0	0.526	0.600	0.444	0.546
		3	0.1	0.684	0.900	0.444	0.643
			1.0	0.579	0.600	0.556	0.600
			10.0	0.368	0.400	0.333	0.400
**P ChemAxon**	70 days	1	0.1	0.714	0.700	0.727	0.700
			1.0	0.667	0.500	0.818	0.714
			10.0	0.667	0.600	0.727	0.667
		2	0.1	0.476	0.500	0.455	0.455
			1.0	0.476	0.700	0.273	0.467
			10.0	0.429	0.500	0.364	0.417
		3	0.1	0.524	0.700	0.364	0.500
			1.0	0.619	0.500	0.727	0.625
			10.0	0.524	0.800	0.273	0.500
	100 days	1	0.1	0.667	0.667	0.667	0.727
			1.0	0.762	0.667	0.889	0.889
			10.0	0.810	0.833	0.778	0.833
		2	0.1	0.524	0.583	0.444	0.583
			1.0	0.476	0.667	0.222	0.533
			10.0	0.429	0.500	0.333	0.500
		3	0.1	0.619	0.833	0.333	0.625
			1.0	0.524	0.667	0.333	0.571
			10.0	0.429	0.667	0.111	0.500

S7. Transformation products

Table S14. Detailed table with transformation products of reference compounds observed in sludge and/or soil

Reference Compound	Reaction step / depth	Reaction in soil	TP in soil	Agreement soil vs. sludge	Observations in sludge	TP candidate in sludge
Azoxystrobin<br>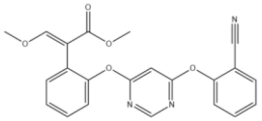<br>**Azoxystrobin**<br>Chemical Formula: $C_{22}H_{17}N_3O_5$<br>Exact Mass: 403.1168	1st step	Hydrolysis of ester<br>-CH2	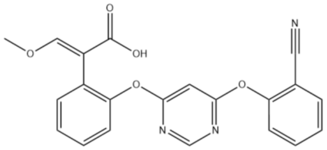<br>**Compound_2_(R234886)**<br>Chemical Formula: $C_{21}H_{15}N_3O_5$<br>Exact Mass: 389.1012<br>★	Observed in both	Confidence level 2b<br>4 experiments<br>Formed equally in NE and VH sludge	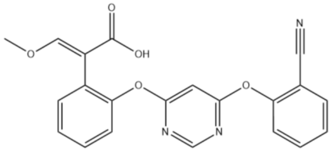<br>**Compound_2_(R234886)**<br>Chemical Formula: $C_{21}H_{15}N_3O_5$<br>Exact Mass: 389.1012
	2nd step	Hydrolysis of nitrile to amide<br>+H2O	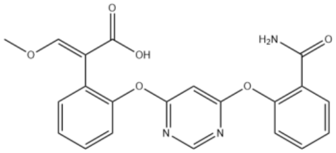<br>**Compound_36_(R403314)**<br>Chemical Formula: $C_{21}H_{17}N_3O_6$<br>Exact Mass: 407.1117	Not observed in sludge		
	2nd step	Cleavage of C-C bond in α-position to carboxylic acid<br>-C2H2O	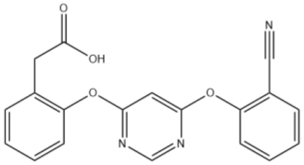<br>**Compound_20_(R402173)**<br>Chemical Formula: $C_{19}H_{13}N_3O_4$<br>Exact Mass: 347.0906	Not observed in sludge		
	> 2nd step	Oxidative ether cleavage Ar-O-Ar<br>+<br><br>Hydroxylation	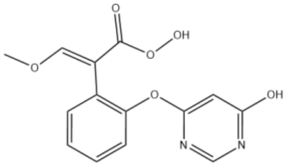<br>**Compound_10**<br>Chemical Formula: $C_{14}H_{12}N_2O_6$<br>Exact Mass: 304.0695	Not observed in sludge		

	1st step	Oxidative ether cleavage Ar-O-Ar	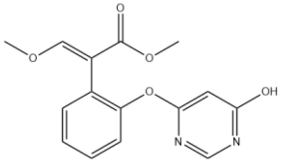 **Compound_3_(R219277)** Chemical Formula: $C_{15}H_{14}N_2O_5$ Exact Mass: 302.0903	Observed in both	Confidence level 2b 4 experiments Formed equally in NE and VH sludge	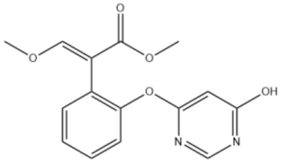 **Compound_3_(R219277)** Chemical Formula: $C_{15}H_{14}N_2O_5$ Exact Mass: 302.0903
	1st step	Oxidative ether cleavage Ar-O-Ar	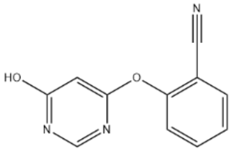 **Compound_28_(R401553)** Chemical Formula: $C_{11}H_7N_3O_2$ Exact Mass: 213.0538	Not observed in sludge		
Bromoxynil 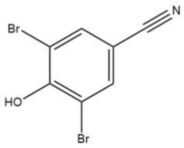 **Bromoxynil** Chemical Formula: $C_7H_3Br_2NO$ Exact Mass: 274.8581	1st step	Hydrolysis of nitrile to amide +H2O	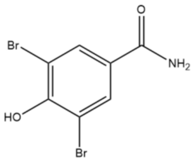 **3,5-dibromo-4-hydroxybenzamide** Chemical Formula: $C_7H_5Br_2NO_2$ Exact Mass: 292.8687 ★	Observed in both	Confidence level 3 2 experiments (neg mode) Formed higher in Neugut sludge	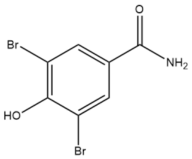 **3,5-dibromo-4-hydroxybenzamide** Chemical Formula: $C_7H_5Br_2NO_2$ Exact Mass: 292.8687
	2nd step	Reductive debromination -Br	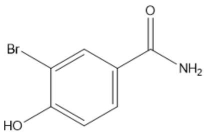 **3-bromo-4-hydroxybenzamide** Chemical Formula: $C_7H_6BrNO_2$ Exact Mass: 214.9582	Not observed in sludge		
	2nd step	Hydrolysis of primary amide to carboxylic acid -NH2 +OH	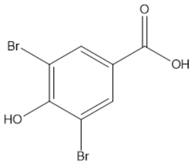 **3,5-dibromo-4-hydroxybenzoic_acid** Chemical Formula: $C_7H_4Br_2O_3$ Exact Mass: 293.8527	Not observed in sludge		

	>2nd step	Reductive debromination (x2)<br>+<br>C-dehydroxylation<br>-Br2-OH (anaerobic conditions)	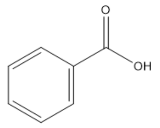<br>**Benzoic acid**<br>Chemical Formula: $C_7H_6O_2$<br>Exact Mass: 122.0368	Not observed in sludge
	>1st step	Reductive debromination<br>-Br<br>+H	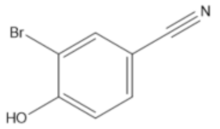<br>**3-bromo-4-hydroxybenzonitrile**<br>Chemical Formula: C_7H_4BrNO<br>★ Exact Mass: 196.9476	Not observed in sludge
	2nd step	Reductive debromination (2x)<br>-Br2<br>+H2	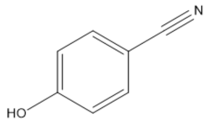<br>**4-hydroxy benzonitrile**<br>Chemical Formula: C_7H_5NO<br>Exact Mass: 119.0371	Not observed in sludge
Benzovindiflupyr	1st step	Oxidative N-demethylation of amine<br>-CH2	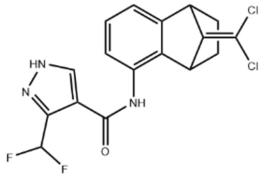<br>**SYN546206**<br>Chemical Formula: $C_{17}H_{13}Cl_2F_2N_3O$<br>Exact Mass: 383.0404	Not observed in sludge

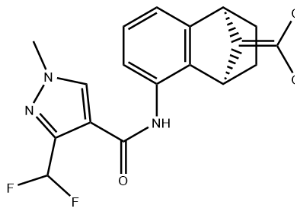 **Benzovindiflupyr** Chemical Formula: $C_{18}H_{15}Cl_2F_2N_3O$ Exact Mass: 397.0560	1st step	C-hydroxylation of non-aromatic ring +O	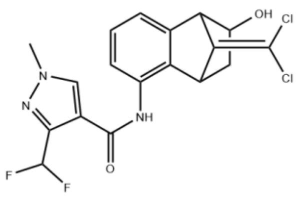 **SYN546040** Chemical Formula: $C_{18}H_{15}Cl_2F_2N_3O_2$ Exact Mass: 413.0509	Not observed in sludge
	1st step	Hydrolysis of secondary amide to carboxylic acid	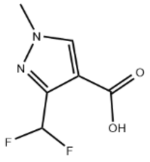 **CSAA798670** Chemical Formula: $C_6H_6F_2N_2O_2$ Exact Mass: 176.0397	Not observed in sludge
Cyantraniliprole	1st step	Intramolecular nucleophilic aromatic substitution-dehydrochlorination -HCl	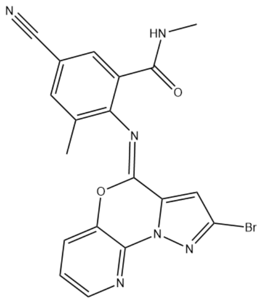 **IN-NXX69** Chemical Formula: $C_{19}H_{13}BrN_6O_2$ Exact Mass: 436.0283	Not observed in sludge

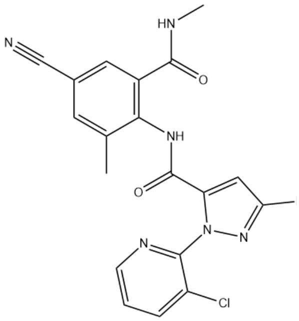 **Cyantraniliprole** Chemical Formula: $C_{19}H_{14}BrClN_6O_2$ Exact Mass: 472.0050	2nd step	Hydrolysis of formimidate derivative + H2O	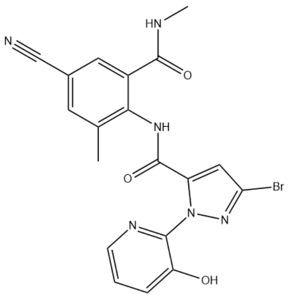 **IN-QKV35** Chemical Formula: $C_{19}H_{15}BrN_6O_3$ Exact Mass: 454.0389	Not observed in sludge
	1st step	Hydrolysis of nitrile to amide +H2O	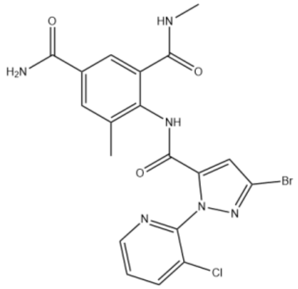 **IN-JCZ38** Chemical Formula: $C_{19}H_{16}BrClN_6O_3$ Exact Mass: 490.0156 ★	Not observed in sludge
	2nd step	Hydrolysis of primary amide to carboxylic acid +O -NH	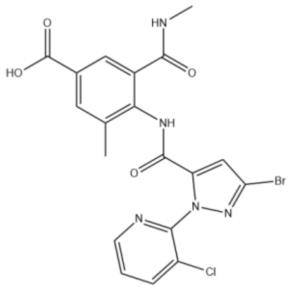 **IN-JSE76** Chemical Formula: $C_{19}H_{15}BrClN_5O_4$ Exact Mass: 490.9996	Not observed in sludge

2nd step	Oxidative N-demethylation of secondary amide	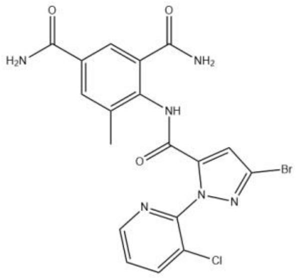<br>**IN-K7H19**<br>Chemical Formula: $C_{18}H_{14}BrClN_6O_3$<br>Exact Mass: 475.9999	Not observed in sludge		
1st step	Nuclephilic aromatic substitution--dehydration<br>-H2O	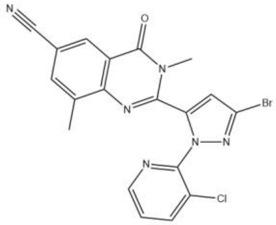<br>**IN-J9Z38**<br>Chemical Formula: $C_{19}H_{12}BrClN_6O$<br>Exact Mass: 453.9944	Observed in both	Confidence level 3<br>3 experiments<br>Possibly abiotic	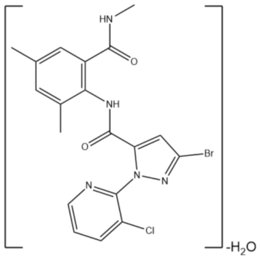<br>Chemical Formula: $C_{19}H_{12}BrClN_6O$<br>Exact Mass: 453.9944
2nd step	Nuclephilic aromatic substitution-dehydrochlorination<br>+ desaturation pyrazole ring<br>-HCl<br>+H2	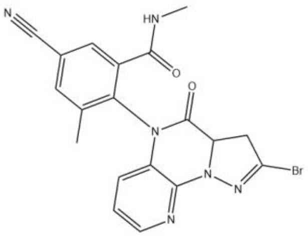<br>**IN-RNU71**<br>Chemical Formula: $C_{19}H_{15}BrN_6O_2$<br>Exact Mass: 438.0440	Not observed in sludge		

	3rd step	Nucleophilic aromatic substitution-dehydrochlorination<br>- desaturation (from IN-RNU71)<br>-H2<br><br>Nucleophilic aromatic substitution-dehydrochlorination<br>- dehydration (from IN-QKV35)<br>-H2O	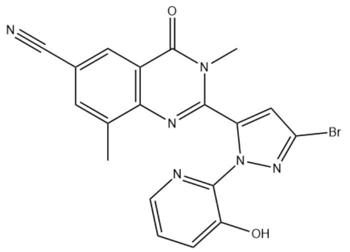<br>**IN-NXX70**<br>Chemical Formula: $C_{19}H_{13}BrN_6O_2$<br>Exact Mass: 436.0283	Not observed in sludge
	2nd step	Hydrolysis of nitrile to amide<br>+H2O	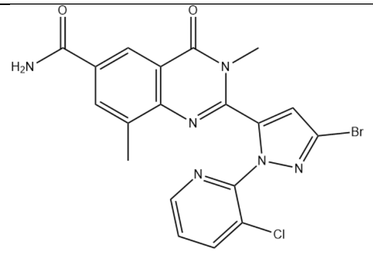<br>**IN-K5A77**<br>Chemical Formula: $C_{19}H_{14}BrClN_6O_2$<br>Exact Mass: 472.0050<br>★	Not observed in sludge
Cyclaniliprole	1st step	Oxidative N-dealkylation of secondary amide	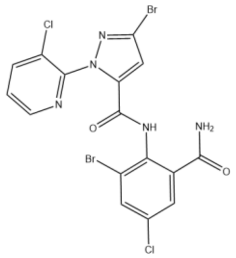<br>**YT-1284**<br>Chemical Formula: $C_{16}H_9Br_2Cl_2N_5O_2$<br>Exact Mass: 530.8500	Not observed in sludge

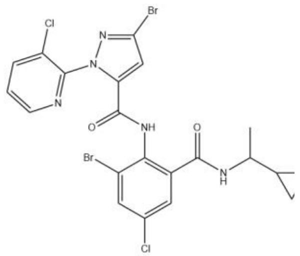<br>**Cyclaniliprole**<br>Chemical Formula: $C_{21}H_{17}Br_2Cl_2N_5O_2$<br>Exact Mass: 598.9126	2nd step	Hydrolysis of primary amide to carboxylic acid<br>+O<br>-NH	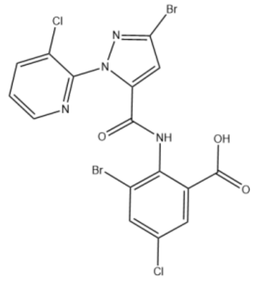<br>**NSY-27**<br>Chemical Formula: $C_{16}H_8Br_2Cl_2N_4O_3$<br>Exact Mass: 531.8340	Not observed in sludge		
Dicamba<br>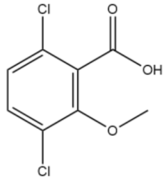<br>**Dicamba**<br>Chemical Formula: $C_8H_6Cl_2O_3$<br>Exact Mass: 219.9694	1st step	C-Hydroxylation of aromatic ring	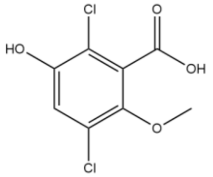<br>**5-OH-Dicamba**<br>Chemical Formula: $C_8H_6Cl_2O_4$<br>Exact Mass: 235.9643	Not observed in sludge		
	1st step	Oxidative ether cleavage<br>-CH3	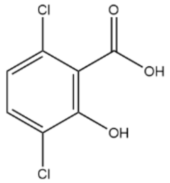<br>**DCSA**<br>Chemical Formula: $C_7H_4Cl_2O_3$<br>Exact Mass: 205.9537<br>★	Observed in both	Confidence level 3<br>1 experiment (neg mode)	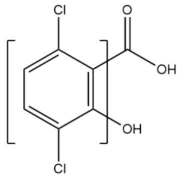<br>Chemical Formula: $C_7H_4Cl_2O_3$<br>Exact Mass: 205.9537
	2nd step	Oxidative ether cleavage<br>+<br>C-Hydroxylation of aromatic ring	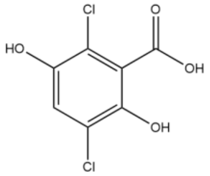<br>**2,5-DIOH**<br>Chemical Formula: $C_7H_4Cl_2O_4$<br>Exact Mass: 221.9487	Not observed in sludge		

Diuron<br>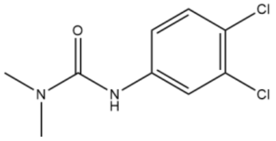<br>**Diuron**<br>Chemical Formula: $C_9H_{10}Cl_2N_2O$<br>Exact Mass: 232.0170	1st step	Oxidative N-demethylation of urea<br>-CH2	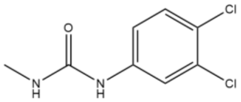<br>**DCPMU**<br>Chemical Formula: $C_8H_8Cl_2N_2O$<br>Exact Mass: 218.0014<br>★	Not observed in sludge		
	2nd step	Oxidative N-demethylation of urea<br>-CH2	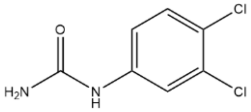<br>**DCPU**<br>Chemical Formula: $C_7H_6Cl_2N_2O$<br>Exact Mass: 203.9857	Observed in both	Confidence level 3<br>1 experiment	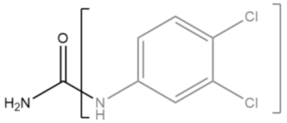<br>Chemical Formula: $C_7H_6Cl_2N_2O$<br>Exact Mass: 203.9857
	3rd step	Hydrolysis of urea<br>-CHNO	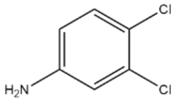<br>**DCA**<br>Chemical Formula: $C_6H_5Cl_2N$<br>Exact Mass: 160.9799	Not observed in sludge		
Fenhexamid<br>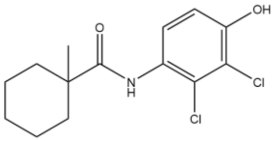<br>**Fenhexamid**<br>Chemical Formula: $C_{14}H_{17}Cl_2NO_2$<br>Exact Mass: 301.0636	1st step	Dimerization	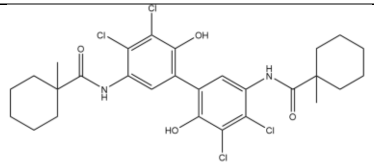<br>**M24**<br>Chemical Formula: $C_{28}H_{32}Cl_4N_2O_4$<br>Exact Mass: 600.1116<br>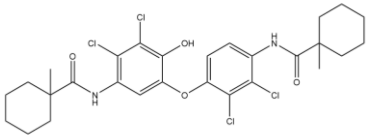<br>**M20_C-O-C_dimer**<br>Chemical Formula: $C_{28}H_{32}Cl_4N_2O_4$<br>Exact Mass: 600.1116	Not observed in sludge		
	1st step	Reductive dechlorination (in sediment)<br>-C<br>+H	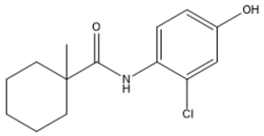<br>**KBR 2738-3-deschloro_M12**<br>Chemical Formula: $C_{14}H_{18}ClNO_2$<br>Exact Mass: 267.1026	Not observed in sludge		

	1st step			Weak evidence in sludge	Hydrolysis of secondary amide into carboxylic acid<br>Confidence level 4 (several isomers)<br>1 experiment	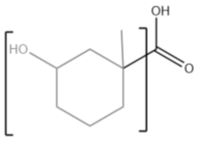Chemical Formula: $C_8H_{14}O_3$<br>Exact Mass: 158.0943
Fenoxycarb<br>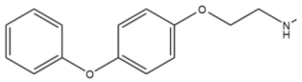**Fenoxycarb**<br>Chemical Formula: $C_{17}H_{19}NO_4$<br>Exact Mass: 301.1314	1st step	Hydrolytic cleavage of C-N bond in α-position to carbamate?<br>-C3H5NO	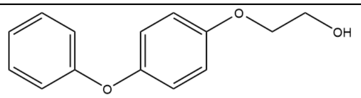**CGA-197811**<br>Chemical Formula: $C_{14}H_{14}O_3$<br>Exact Mass: 230.0943	Not observed in sludge		
	2nd step	Oxidative ether cleavage	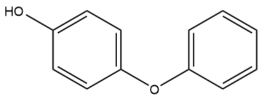**CGA-26021**<br>Chemical Formula: $C_{12}H_{10}O_2$<br>Exact Mass: 186.0681	Not observed in sludge		
	1st step	Carbamate hydrolysis	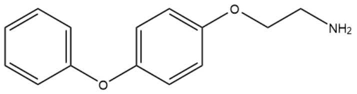**CGA-197810**<br>Chemical Formula: $C_{14}H_{15}NO_2$<br>Exact Mass: 229.1103	Not observed in sludge		
	2nd step	C-Hydroxylation of aromatic ring	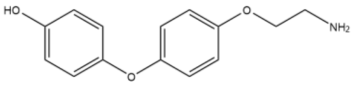**CGA-344889**<br>Chemical Formula: $C_{14}H_{15}NO_3$<br>Exact Mass: 245.1052	Weak evidence in sludge	Confidence level 4<br>1 experiment<br>Only in VH sludge	
	1st step	Oxidative cleavage of amide (α-position to carbamate)<br>+<br>oxidation to carboxylic acid<br>+	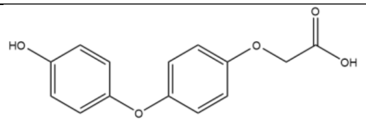**CGA-294848**<br>Chemical Formula: $C_{14}H_{12}O_5$<br>Exact Mass: 260.0685	Not observed in sludge		

		hydroxylation of aromatic ring				
	2nd step	Oxidative ether cleavage	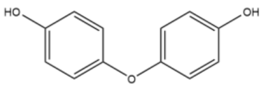<br>**CGA-195935**<br>Chemical Formula: $C_{12}H_{10}O_3$<br>Exact Mass: 202.0630	Not observed in sludge		
	1st step	C-Hydroxylation of aromatic ring<br>+O	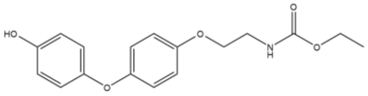<br>**CGA-294850_Ro_16-8797**<br>Chemical Formula: $C_{17}H_{19}NO_5$<br>Exact Mass: 317.1263	Observed in both	Confidence level 3<br>3 experiments	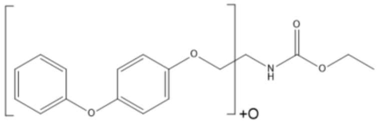<br>Chemical Formula: $C_{17}H_{19}NO_5$<br>Exact Mass: 317.1263
	2nd step	Oxidative ether cleavage	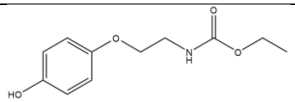<br>**CGA-294847**<br>Chemical Formula: $C_{11}H_{15}NO_4$<br>Exact Mass: 225.1001	Observed in both	Confidence level 2b<br>2 experiments	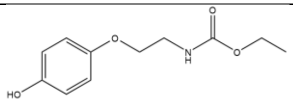<br>**CGA-294847**<br>Chemical Formula: $C_{11}H_{15}NO_4$<br>Exact Mass: 225.1001
Fipronil<br>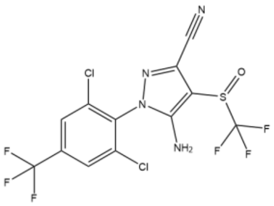<br>**Fipronil**<br>Chemical Formula: $C_{12}H_4Cl_2F_6N_4OS$<br>Exact Mass: 435.9387	1st step	Hydrolysis of nitrile to amide<br>+H2O	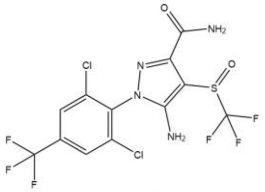<br>★ **RPA_200766**<br>Chemical Formula: $C_{12}H_6Cl_2F_6N_4O_2S$<br>Exact Mass: 453.9493	Not observed in sludge		
	2nd step	Hydrolysis of primary amide to carboxylic acid<br>+O<br>-NH	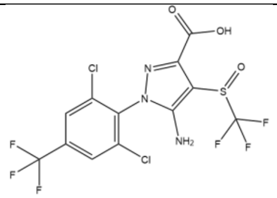<br>**RPA_200761**<br>Chemical Formula: $C_{12}H_5Cl_2F_6N_3O_3S$<br>Exact Mass: 454.9333	Not observed in sludge		

	1st step	Oxidation of sulfoxide to sulfone<br>+O	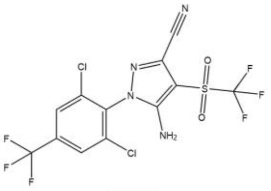<br>**MB_46136**<br>Chemical Formula: $C_{12}H_4Cl_2F_6N_4O_2S$<br>Exact Mass: 451.9336<br>★	Observed in both	Confidence level 3<br>1 experiment<br>Possibly abiotic maybe	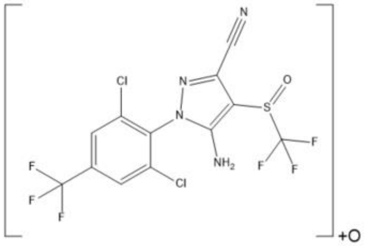<br>Chemical Formula: $C_{12}H_5Cl_2F_6N_3O_2S$<br>Exact Mass: 438.9384
	1st step	S-reduction sulfoxide to sulfide<br>-O	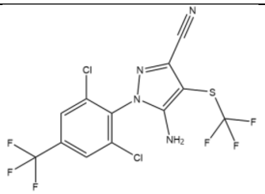<br>**MB_45950**<br>Chemical Formula: $C_{12}H_4Cl_2F_6N_4S$<br>Exact Mass: 419.9438<br>★	Not observed in sludge		
Florasulam<br>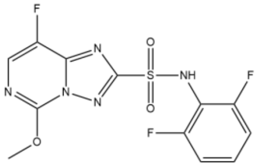<br>**Florasulam**<br>Chemical Formula: $C_{12}H_8F_3N_5O_3S$<br>Exact Mass: 359.0300	1st step	Oxidative ether cleavage<br>-CH2	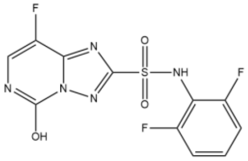<br>**5-OH_Florasulam**<br>Chemical Formula: $C_{11}H_6F_3N_5O_3S$<br>Exact Mass: 345.0143<br>★	Observed in both	Confidence level 2b<br>4 experiments	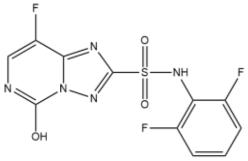<br>**5-OH_Florasulam**<br>Chemical Formula: $C_{11}H_6F_3N_5O_3S$<br>Exact Mass: 345.0143
	2nd step	Oxidation of triazolo-pyrimidine moiety	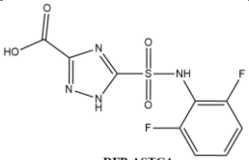<br>**DFP-ASTCA**<br>Chemical Formula: $C_9H_6F_2N_4O_4S$<br>Exact Mass: 304.0078<br>★	Not observed in sludge		
	3rd step	Oxidative cleavage of sulfanilamide moiety	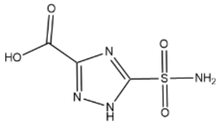<br>**ASTCA**<br>Chemical Formula: $C_3H_4N_4O_4S$<br>Exact Mass: 191.9953<br>★	Not observed in sludge		

	3rd step	Decarboxylation of triazole ring<br>-COO	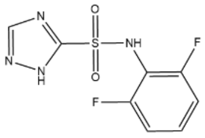<br>**DFP-TSA**<br>Chemical Formula: $C_8H_6F_2N_4O_2S$<br>Exact Mass: 260.0180	Not observed in sludge		
	4th step	Oxidative cleavage of sulfanilamide moiety<br>+<br>Decarboxylation of triazole ring	★ 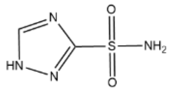<br>**TSA**<br>Chemical Formula: $C_2H_4N_4O_2S$<br>Exact Mass: 148.0055	Not observed in sludge		
Fluopyram<br>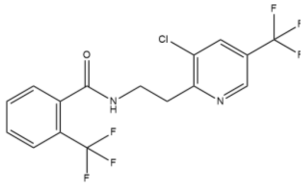<br>**Fluopyram**<br>Chemical Formula: $C_{16}H_{11}ClF_6N_2O$<br>Exact Mass: 396.0464	1st step	C-hydroxylation<br>+O	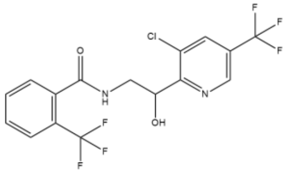<br>**7-hydroxy-Fluopyram**<br>Chemical Formula: $C_{16}H_{11}ClF_6N_2O_2$<br>Exact Mass: 412.0413	Observed in both	Confidence level 3<br>3 experiments	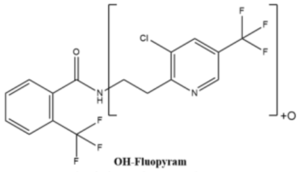<br>**OH-Fluopyram**<br>Chemical Formula: $C_{16}H_{11}ClF_6N_2O_2$<br>Exact Mass: 412.0413
	2nd step	Oxidative N-dealkylation of secondary amide	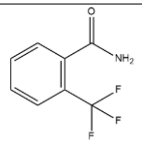<br>**Benzamide**<br>Chemical Formula: $C_8H_6F_3NO$<br>Exact Mass: 189.0401	Observed in both	Confidence level 3<br>1 experiment	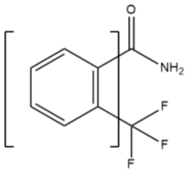<br>Chemical Formula: $C_8H_6F_3NO$<br>Exact Mass: 189.0401
	2nd step	Oxidative cleavage of secondary alcohol	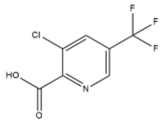<br>**AE_C657188**<br>Chemical Formula: $C_7H_3ClF_3NO_2$<br>Exact Mass: 224.9804	Not observed in sludge		

	3rd step	Sulfoxidation-dechlorination of chlorinated pyridyl ring	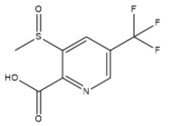 **AE_1344122** Chemical Formula: $C_8H_6F_3NO_3S$ Exact Mass: 253.0020	Not observed in sludge		
Flupyradifurone 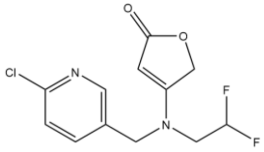 **Flupyradifurone** Chemical Formula: $C_{12}H_{11}ClF_2N_2O_2$ Exact Mass: 288.0477	1st step	Oxidative N-dealkylation of tertiary amine	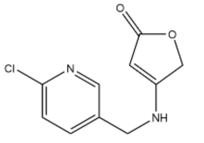 **BYI_02960-desdifluoroethyl** Chemical Formula: $C_{10}H_9ClN_2O_2$ Exact Mass: 224.0353	Weak evidence in sludge	Confidence level 5 (m/z) 1 experiment	
	1st step	Chlorination of lactone ring	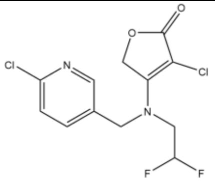 **BYI_02960-chloro** Chemical Formula: $C_{12}H_{10}Cl_2F_2N_2O_2$ Exact Mass: 322.0087	Not observed in sludge		
	1st step	Oxidative N-dealkylation of amine (also TP of imidacloprid)	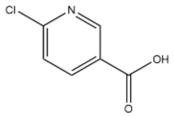 **6-chloronicotinic-acid** Chemical Formula: $C_6H_4ClNO_2$ Exact Mass: 156.9931 ★	Weak evidence in sludge	Confidence level 4 (isomers like chloronitrobenzenes) 2 experiments	
	1st step			Weak evidence in sludge	Oxidative N-dealkylation of amine Confidence level 4 1 experiment	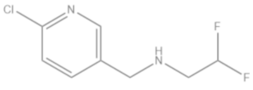 **Flup_TP_016** Chemical Formula: $C_8H_9ClF_2N_2$ Exact Mass: 206.0422
Imidacloprid 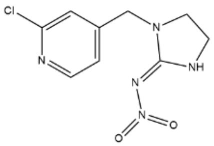 **Imidacloprid** Chemical Formula: $C_9H_{10}ClN_5O_2$ Exact Mass: 255.0523	1st step	C-Hydroxylation +O	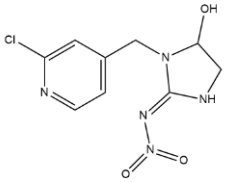 **NTN33893-5-hydroxy** Chemical Formula: $C_9H_{10}ClN_5O_3$ Exact Mass: 271.0472	Observed in both	Confidence level 3 1 experiment Higher levels in VH sludge	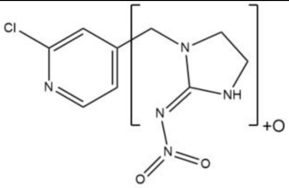 Chemical Formula: $C_9H_{10}ClN_5O_3$ Exact Mass: 271.0472

2nd step	Dehydration<br>-H2O	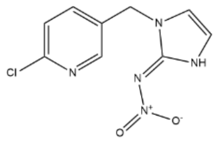<br>**NTN33893-olefine**<br>Chemical Formula: $C_9H_8ClN_5O_2$<br>Exact Mass: 253.0367	Not observed in sludge		
1st step	Reduction of nitro group<br>-O	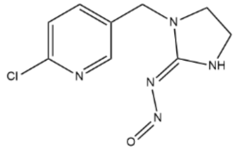<br>**NTN33893-nitrosimine**<br>Chemical Formula: $C_9H_{10}ClN_5O$<br>Exact Mass: 239.0574	Weak evidence in sludge	Confidence level 4 (no MS2)<br>1 experiment	
1st step	Cleavage of nitro group from nitroguanidine derivative<br>-NO2<br>+H	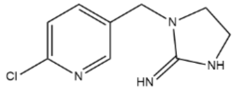<br>**NTN33893-desnitro**<br>Chemical Formula: $C_9H_{11}ClN_4$<br>Exact Mass: 210.0672	Observed in both	Confidence level 2b<br>2 experiments	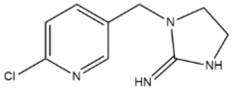<br>**NTN33893-desnitro**<br>Chemical Formula: $C_9H_{11}ClN_4$<br>Exact Mass: 210.0672
2nd step	Hydrolysis of imine group<br>-NH2<br>+O	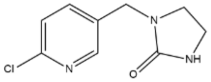<br>**NTN33893-urea**<br>Chemical Formula: $C_9H_{10}ClN_3O$<br>Exact Mass: 211.0512	Observed in both	Confidence level 2b<br>1 experiment	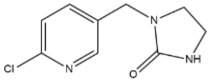<br>**NTN33893-urea**<br>Chemical Formula: $C_9H_{10}ClN_3O$<br>Exact Mass: 211.0512
2nd step	Desaturation of imidazol ring<br>-H2	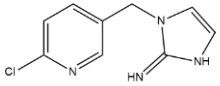<br>**NTN33893-_desnitro_olefine**<br>Chemical Formula: $C_9H_9ClN_4$<br>Exact Mass: 208.0516<br>★	Observed in both	Confidence level 3<br>2 experiments	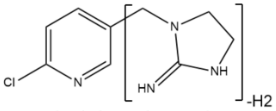<br>Chemical Formula: $C_9H_9ClN_4$<br>Exact Mass: 208.0516
3rd step	Oxidative cleavage of imidazol ring	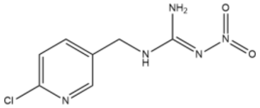<br>**NTN33893-ring-open-nitroguanidine**<br>Chemical Formula: $C_7H_8ClN_5O_2$<br>Exact Mass: 229.0367	Not observed in sludge		

Isoproturon<br>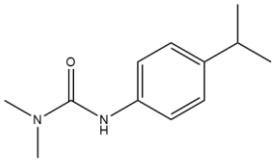<br>**Isoproturon**<br>Chemical Formula: $C_{12}H_{18}N_2O$<br>Exact Mass: 206.1419	1st step	C-Hydroxylation<br>+O	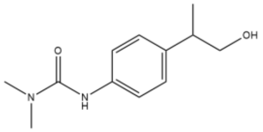<br>**1-OH-isoproturon**<br>Chemical Formula: $C_{12}H_{18}N_2O_2$<br>Exact Mass: 222.1368	Observed in both	Confidence level 3 (same as RPA_41060)<br>3 experiments	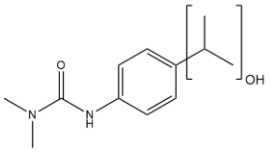<br>**hydroxy-isoproturon**<br>Chemical Formula: $C_{12}H_{18}N_2O_2$<br>Exact Mass: 222.1368
	2nd step	Oxidation of alcohol to carboxylic acid<br>-H2<br>+O2	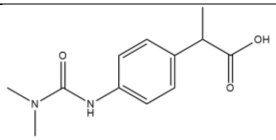<br>**2-{4-[(dimethylcarbamoyl) amino] phenyl} propanoic_acid**<br>Chemical Formula: $C_{12}H_{16}N_2O_3$<br>Exact Mass: 236.1161	Observed in both	Confidence level 3<br>4 experiments	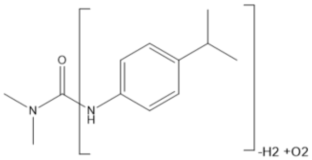<br>Chemical Formula: $C_{12}H_{16}N_2O_3$<br>Exact Mass: 236.1161
	1st step	C-Hydroxylation<br>+O	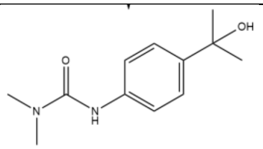<br>**RPA_410260**<br>Chemical Formula: $C_{12}H_{18}N_2O_2$<br>Exact Mass: 222.1368	Observed in both	Confidence level 3 (same as 1-OH isoproturon)<br>3 experiments	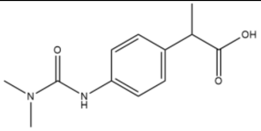<br>**2-{4-[(dimethylcarbamoyl) amino] phenyl} propanoic_acid**<br>Chemical Formula: $C_{12}H_{16}N_2O_3$<br>Exact Mass: 236.1161
	2nd step	Oxidative N-demethylation of urea<br>-CH2	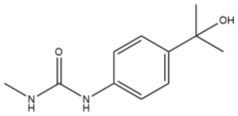<br>**RPA_415044**<br>Chemical Formula: $C_{11}H_{16}N_2O_2$<br>Exact Mass: 208.1212	Observed in both	Confidence level 3<br>3 experiments	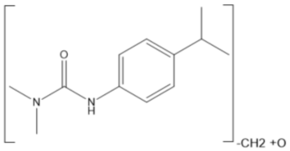<br>Chemical Formula: $C_{11}H_{16}N_2O_2$<br>Exact Mass: 208.1212
	1st step	Oxidative N-demethylation of urea<br>-CH2	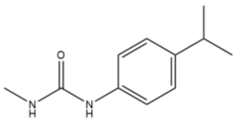<br>**Desmethyl-isoproturon**<br>Chemical Formula: $C_{11}H_{16}N_2O$<br>Exact Mass: 192.1263<br>★	Observed in both	Confidence level 2a<br>3 experiments	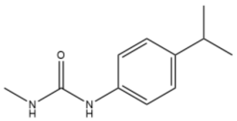<br>**Desmethyl-isoproturon**<br>Chemical Formula: $C_{11}H_{16}N_2O$<br>Exact Mass: 192.1263
	2nd step	Oxidative N-demethylation of urea<br>-CH2	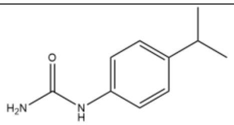<br>**[N-(4-isopropylphenyl)-urea_LS730334_didesmethyl-isoproturon**<br>Chemical Formula: $C_{10}H_{14}N_2O$<br>Exact Mass: 178.1106	Observed in both	Confidence level 2a<br>3 experiments	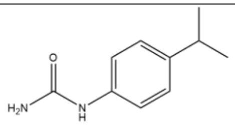<br>**[N-(4-isopropylphenyl)-urea_LS730334_didesmethyl-isoproturon**<br>Chemical Formula: $C_{10}H_{14}N_2O$<br>Exact Mass: 178.1106

	>3rd steps	Hydroxylation + oxidation of alcohol<br>-H2<br>+O2	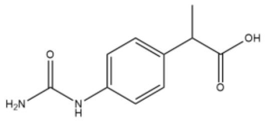<br>**RPA_409657**<br>Chemical Formula: $C_{10}H_{12}N_2O_3$<br>Exact Mass: 208.0848	maybe	Confidence level 4<br>1 experiment	
Kresoxim-methyl<br>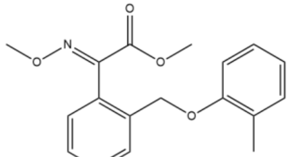<br>**Kresoxim-methyl**<br>Chemical Formula: $C_{18}H_{19}NO_4$<br>Exact Mass: 313.1314	1st step	Hydrolysis of ester<br>-CH2	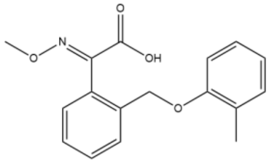<br>**BF_490-1**<br>Chemical Formula: $C_{17}H_{17}NO_4$<br>Exact Mass: 299.1158<br>★	Observed in both	Confidence level 2b<br>3 experiments	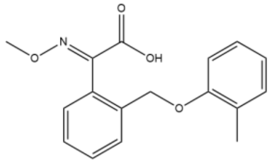<br>**BF_490-1**<br>Chemical Formula: $C_{17}H_{17}NO_4$<br>Exact Mass: 299.1158
	2nd step	Hydroxylation of aromatic methyl + oxidation of alcohol	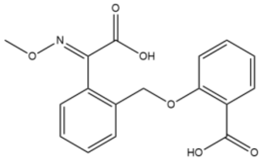<br>**490M4**<br>Chemical Formula: $C_{17}H_{15}NO_6$<br>Exact Mass: 329.0899	Not observed in sludge		
Mandipropamid<br>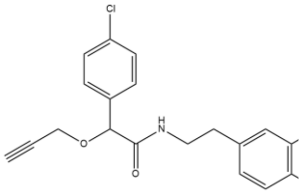<br>**Mandipropamid**<br>Chemical Formula: $C_{23}H_{22}ClNO_4$<br>Exact Mass: 411.1237	1st step	Reduction of propynyl group to propenyl<br>+H2	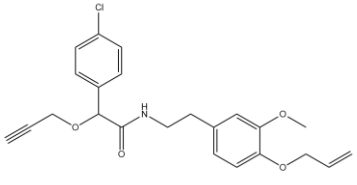<br>**SYN_536638**<br>Chemical Formula: $C_{23}H_{24}ClNO_4$<br>Exact Mass: 413.1394	Not observed in sludge		
	2nd step	Oxidative ether cleavage of aliphatic group<br>-C3H2	★<br>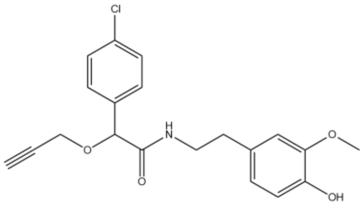<br>**NOA_458422**<br>Chemical Formula: $C_{20}H_{20}ClNO_4$<br>Exact Mass: 373.1081	Observed in both	Confidence level 3 (isomer to CGA-380778<br>2 experiments	

3rd step	Oxidative ether cleavage / O-demethylation	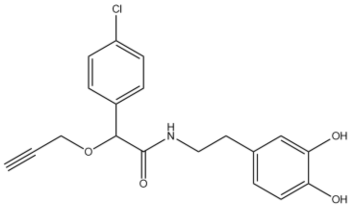 **SYN_505503** Chemical Formula: $C_{19}H_{18}ClNO_4$ Exact Mass: 359.0924	Observed in both	Confidence level 4<br>2 experiments	
2nd step	Hydroxylation	U7	Not observed in sludge		
>1st step	Hydroxylation (desaturation from U7)	U8	Not observed in sludge		
1st step	Oxidative ether cleavage of propynyl group<br>-C3H2	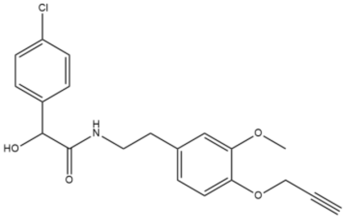 **CGA_380778** Chemical Formula: $C_{20}H_{20}ClNO_4$ Exact Mass: 373.1081	Observed in both	Confidence level 3 (isomer to NOA-458422)<br>2 experiments	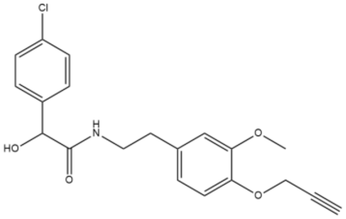 **CGA_380778** Chemical Formula: $C_{20}H_{20}ClNO_4$ Exact Mass: 373.1081
2nd step	Hydrolysis of secondary amide	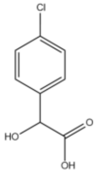 **NOA_459119** Chemical Formula: $C_8H_7ClO_3$ Exact Mass: 186.0084	Not observed in sludge		
>1st step			Observed only in sludge	Oxidative cleavage of secondary amide + oxidation of aldehyde to carboxylic acid<br>Confidence level 4<br>1 experiment	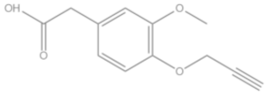 **Man_TP_180** Chemical Formula: $C_{12}H_{12}O_4$ Exact Mass: 219.0663

	1st step	Oxidative ether cleavage / O-demethylation -CH2	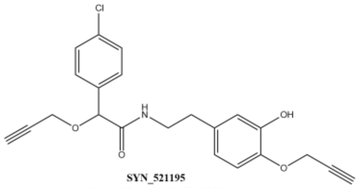 SYN_521195<br>Chemical Formula: $C_{22}H_{20}ClNO_4$<br>Exact Mass: 397.1081	Not observed in sludge		
	2nd step	Hydrolysis of secondary amide	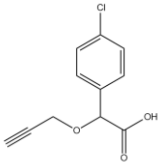 SYN_500003<br>Chemical Formula: $C_{11}H_9ClO_3$<br>Exact Mass: 224.0240	Not observed in sludge		
Mesotrione 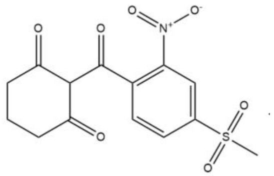 Mesotrione<br>Chemical Formula: $C_{14}H_{13}NO_7S$<br>Exact Mass: 339.0413	1st step	Oxidative cleavage α-position of triketone	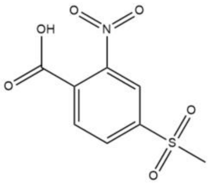 MNBA<br>Chemical Formula: $C_8H_7NO_6S$<br>Exact Mass: 244.9994<br>★	Not observed in sludge		
	2nd step	Reduction of nitro group to amine	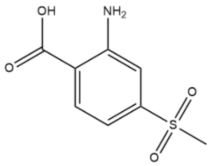 AMBA<br>Chemical Formula: $C_8H_9NO_4S$<br>Exact Mass: 215.0252	Observed in both	Confidence level 2b<br>1 experiment	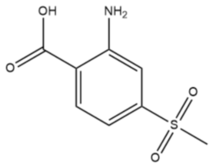 AMBA<br>Chemical Formula: $C_8H_9NO_4S$<br>Exact Mass: 215.0252
	1st step	Cyclization-tautomerism of nitro & ketone groups? -O3<br><br>(in sediment)	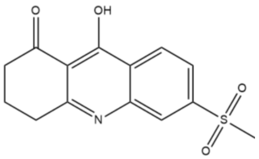 SYN546974<br>Chemical Formula: $C_{14}H_{13}NO_4S$<br>Exact Mass: 291.0565<br>(sediment)	Not observed in sludge		

Oxathiapiprolin 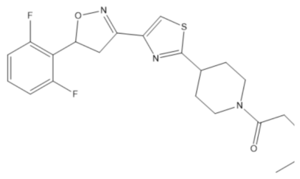 **Oxathiapiprolin** Chemical Formula: $C_{24}H_{22}F_5N_5O_2S$ Exact Mass: 539.1414	1st step	Hydroxylation of methyl on pyrazole ring + oxidation to carboxylic acid -H2 +O2	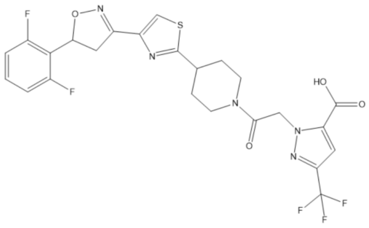 **IN-RAB06** Chemical Formula: $C_{24}H_{20}F_5N_5O_4S$ Exact Mass: 569.1156	Observed in both	Confidence level 3<br>1 experiment	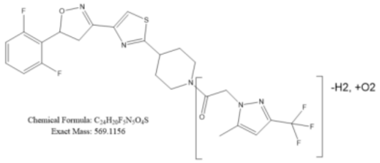
	2nd step	Oxidative N-dealkylation of tertiary amine	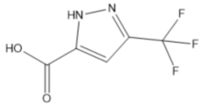 **IN-E8S72** Chemical Formula: $C_5H_3F_3N_2O_2$ Exact Mass: 180.0147	Observed in both	Confidence level 4<br>1 experiment (neg mode)	
	1st step	Cleavage of thiazole ring	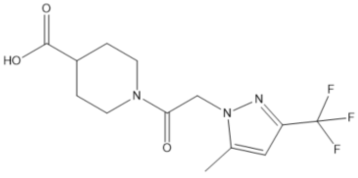 **IN-RLD51** Chemical Formula: $C_{13}H_{16}F_3N_3O_3$ Exact Mass: 319.1144	Observed in both	Confidence level 3<br>2 experiments	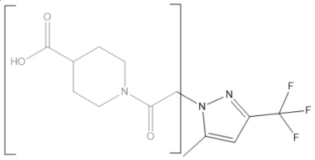 Chemical Formula: $C_{13}H_{16}F_3N_3O_3$ Exact Mass: 319.1144
	1st step	C-hydroxylation +O	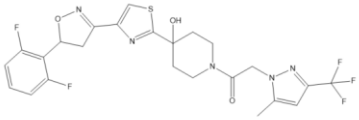 **IN-RDT31** Chemical Formula: $C_{24}H_{22}F_5N_5O_3S$ Exact Mass: 555.1364	Observed in both	Confidence level 3<br>1 experiment	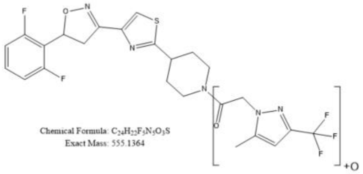

	>=1st step	Hydrolysis of tertiary amide	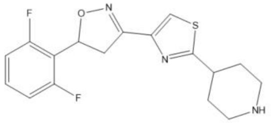 **IN-QPS10** Chemical Formula: $C_{17}H_{17}F_2N_3OS$ Exact Mass: 349.1060	Observed in both	Confidence level 2b 1 experiment	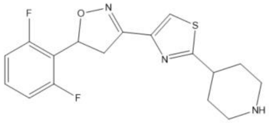 **IN-QPS10** Chemical Formula: $C_{17}H_{17}F_2N_3OS$ Exact Mass: 349.1060
			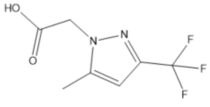 **IN-WR791** Chemical Formula: $C_7H_7F_3N_2O_2$ Exact Mass: 208.0460	Observed in both	Confidence level 2b 4 experiments	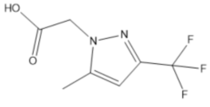 **IN-WR791** Chemical Formula: $C_7H_7F_3N_2O_2$ Exact Mass: 208.0460
Terbuthylazine 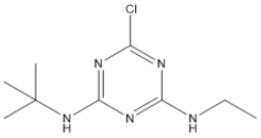 **Terbuthylazine** Chemical Formula: $C_9H_{16}ClN_5$ Exact Mass: 229.1094	1st step	Hydrolytic dechlorination of triazine ring -Cl +OH	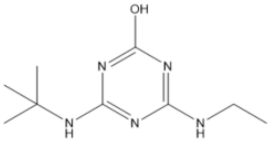 **MT13_GS_23158** Chemical Formula: $C_9H_{17}N_5O$ Exact Mass: 211.1433 ★	Observed in both	Confidence level 2b 3 experiments	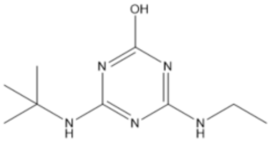 **MT13_GS_23158** Chemical Formula: $C_9H_{17}N_5O$ Exact Mass: 211.1433
	1st step	Oxidative N-deethylation of amine -C2H4	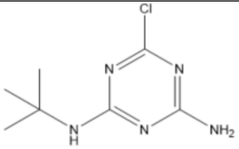 **MT1_GS_26379** Chemical Formula: $C_7H_{12}ClN_5$ Exact Mass: 201.0781 ★	Observed in both	Confidence level 2b 4 experiments	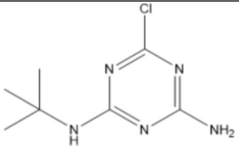 **MT1_GS_26379** Chemical Formula: $C_7H_{12}ClN_5$ Exact Mass: 201.0781
	2nd step	Hydrolytic dechlorination of triazine ring -Cl	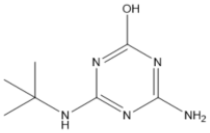 **MT14** Chemical Formula: $C_7H_{13}N_5O$ Exact Mass: 183.1120	Not observed in sludge		
	1st step	Hydroxylation of methyl + Oxidation of alcohol -H2 +O2	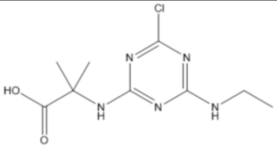 **GS31398** Chemical Formula: $C_9H_{14}ClN_5O_2$ Exact Mass: 259.0836	Observed in both	Confidence level 3 2 experiments	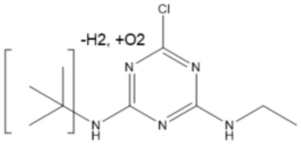 Chemical Formula: $C_9H_{14}ClN_5O_2$ Exact Mass: 259.0836

Topramezone<br>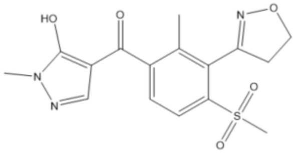<br>**Topramezone**<br>Chemical Formula: $C_{16}H_{17}N_3O_5S$<br>Exact Mass: 363.0889	1st step	Aldoxime to nitrile	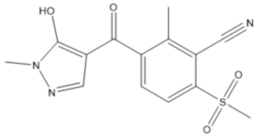<br>**M670H01**<br>Chemical Formula: $C_{14}H_{13}N_3O_4S$<br>Exact Mass: 319.0627	Not observed in sludge		
	1st step	Oxidative cleavage of pyrazole ring	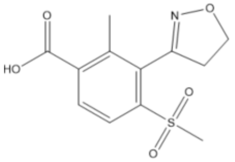<br>**M670H05**<br>Chemical Formula: $C_{12}H_{13}NO_5S$<br>★ Exact Mass: 283.0514	Observed in both	Confidence level 2b<br>1 experiment	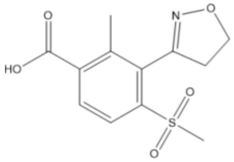<br>**M670H05**<br>Chemical Formula: $C_{12}H_{13}NO_5S$<br>Exact Mass: 283.0514
	> 2nd step	Decarboxylation of benzoic acid + C-Hydroxylation of methyl	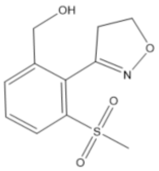<br>**M670H09**<br>Chemical Formula: $C_{11}H_{13}NO_4S$<br>Exact Mass: 255.0565	Not observed in sludge		

S8. References

(1) Lewis, K. A.; Tzilivakis, J.; Warner, D. J.; Green, A. An international database for pesticide risk assessments and management. *Human and Ecological Risk Assessment: An International Journal* **2016**, *22* (4), 1050-1064. DOI: 10.1080/10807039.2015.1133242.
(2) Fenner, K.; Screpanti, C.; Renold, P.; Rouchdi, M.; Vogler, B.; Rich, S. Comparison of Small Molecule Biotransformation Half-Lives between Activated Sludge and Soil: Opportunities for Read-Across? *Environ Sci Technol* **2020**, *54* (6), 3148-3158. DOI: 10.1021/acs.est.9b05104.
(3) Mansouri, K.; Williams, A. J. QMRF - KOC model for the soil adsorption coefficient prediction from OPERA models. **2017**, *RN: Q17-26-0017*. DOI: 10.13140/RG.2.2.27831.32163/1.
(4) Mansouri, K.; Grulke, C. M.; Judson, R. S.; Williams, A. J. OPERA models for predicting physicochemical properties and environmental fate endpoints. *Journal of Cheminformatics* **2018**, *10* (1), 10. DOI: 10.1186/s13321-018-0263-1.
(5) Azis, K.; Ntougias, S.; Melidis, P. NH4+-N versus pH and ORP versus NO3−-N sensors during online monitoring of an intermittently aerated and fed membrane bioreactor. *Environmental Science and Pollution Research* **2021**, *28* (26), 33837-33843. DOI: 10.1007/s11356-020-10534-4.
(6) Kokina, K.; Mezule, L.; Gruskevica, K.; Neilands, R.; Golovko, K.; Juhna, T. Impact of Rapid pH Changes on Activated Sludge Process. *Applied Sciences* **2022**, *12* (11), 5754.
(7) Liu, Y.; Ngo, H. H.; Guo, W.; Peng, L.; Wang, D.; Ni, B. The roles of free ammonia (FA) in biological wastewater treatment processes: A review. *Environment International* **2019**, *123*, 10-19. DOI: https://doi.org/10.1016/j.envint.2018.11.039.
(8) Zhou, Y.; Oehmen, A.; Lim, M.; Vadivelu, V.; Ng, W. J. The role of nitrite and free nitrous acid (FNA) in wastewater treatment plants. *Water Research* **2011**, *45* (15), 4672-4682. DOI: https://doi.org/10.1016/j.watres.2011.06.025.
(9) Fenner, K.; Men, Y. Comment on "Role of Ammonia Oxidation in Organic Micropollutant Transformation during Wastewater Treatment": Overlooked Evidence to the Contrary. *Environmental Science & Technology* **2021**, *55* (17), 12128-12129. DOI: 10.1021/acs.est.1c04178.
(10) Su, Q.; Schittich, A.-R.; Jensen, M. M.; Ng, H.; Smets, B. F. Role of Ammonia Oxidation in Organic Micropollutant Transformation during Wastewater Treatment: Insights from Molecular, Cellular, and Community Level Observations. *Environmental Science & Technology* **2021**, *55* (4), 2173-2188. DOI: 10.1021/acs.est.0c06466.
(11) Nsenga Kumwimba, M.; Meng, F. Roles of ammonia-oxidizing bacteria in improving metabolism and cometabolism of trace organic chemicals in biological wastewater treatment processes: A review. *Science of The Total Environment* **2019**, *659*, 419-441. DOI: https://doi.org/10.1016/j.scitotenv.2018.12.236.
(12) Baldwin, D. D.; Campbell, C. E. Short-Term Effects of Low pH on the Microfauna of an Activated Sludge Wastewater Treatment System. *Water Quality Research Journal* **2001**, *36* (3), 519-535. DOI: 10.2166/wqrj.2001.028 (acccessed 5/5/2023).
(13) Lombardo, A.; Manganaro, A.; Arning, J.; Benfenati, E. Development of new QSAR models for water, sediment, and soil half-life. *Science of The Total Environment* **2022**, *838*, 156004. DOI: https://doi.org/10.1016/j.scitotenv.2022.156004.

(14) *Estimation Programs Interface Suite™ for Microsoft® Windows, v 4.11*; United States Environmental Protection Agency: Washington, DC, USA, 2012.
(15) Arnot, J. A.; Gouin, T.; Mackay, D. *Practical Methods for Estimating Environmental Biodegradation Rates, Technical Report to Environment Canada*; Canadian Environmental Modelling Network, Trent, University, Peterborough, CEMN Report No 200503, 2005.
